# Is there evidence for the asymmetrical transfer of strength to an untrained limb?

**DOI:** 10.1007/s00421-024-05472-9

**Published:** 2024-04-03

**Authors:** Vickie Wong, Jun Seob Song, Yujiro Yamada, Ryo Kataoka, William B. Hammert, Robert W. Spitz, Jeremy P. Loenneke

**Affiliations:** 1 Department of Sport and Health, Solent University, Southampton, Hampshire SO14 0YN UK; 2 Kevser Ermin Applied Physiology Laboratory, The University of Mississippi, University, P.O. Box 1848, University, MS 38677 USA; 3 Department of Physiology and Biophysics, University of Mississippi Medical Center, Jackson, MS USA

**Keywords:** Asymmetry, Exercise, Crossover, Strength, Neural

## Abstract

**Purpose:**

The literature predominantly addresses cross-education of strength in the dominant limb rather than the non-dominant limb, guided by the hypothesis of an asymmetrical transfer of strength from unilateral training protocols. The purpose of the study was to review the literature and determine how much evidence was available to support this claim. A meta-analysis was performed to estimate the magnitude of this hypothesized asymmetrical transfer of strength.

**Methods:**

A literature search of all possible records was implemented using Cochrane Library, PubMed, and Scopus from February 2022 to May 2022. Comparison of randomized controlled trials was computed. The change scores and standard deviations of those change scores were extracted for each group. Only three studies met the criteria, from which a total of five effect sizes were extracted and further analyzed.

**Results:**

The overall effect of resistance training of the dominant limb on strength transfer to the non-dominant limb relative to the effects of resistance training the non-dominant limb on strength transfer to the dominant (non-training) limb was 0.46 (SE 0.42). The analysis from this study resulted in minimal support for the asymmetry hypothesis. Given the small number of studies available, we provide the effect but note that the estimate is unlikely to be stable.

**Conclusion:**

Although it is repeatedly stated that there is an asymmetrical transfer of strength, our results find little support for that claim. This is not to say that it does not exist, but additional research implementing a control group and a direct comparison between limbs is needed to better understand this question.

**Supplementary Information:**

The online version contains supplementary material available at 10.1007/s00421-024-05472-9.

## Introduction

Cross-education or the crossover effect has been described as an increase in skill or muscular abilities in an untrained limb from educating (i.e., training) an opposite limb (Scripture et al. 1894). Many of the initial studies, which observed a crossover effect, experimented with precise skills such as printing inverted and reversed letters (Hicks 1974; Parlow and Kinsbourne 1989) and finger mazes (Stoddard and Vaid 1996). The initial recording of cross-education of strength was by Scripture and colleagues (1894), where one participant observed an increase in strength in a limb that was not trained after unilaterally training the contralateral homologous limb by squeezing a rubber bulb. Since then, numerous studies have explored cross-education as a potential rehabilitation method, investigating its mechanisms and employing various types of resistance training (e.g., isometric, concentric, eccentric, etc.) to elucidate its effects (Farthing et al. 2007; Carroll et al. 2008; Hendy et al. 2012; Lepley and Palmieri-Smith 2014). Although research on this has been highly studied, the mechanisms are still not definitive; however, most would agree that there is a neural component consisting of cortical adaptations (Hortobágyi et al. 2003; Lagerquist et al. 2006; Farthing et al. 2007; Ruddy et al. 2017; Green and Gabriel 2018a).

The brain is partitioned into a seemingly symmetrical left and right hemisphere. In regard to motor control, the left hemisphere is generally responsible for the right side of the body and the right hemisphere is responsible for the left side of the body (Kimura 1973). The functions of the right and left hemispheres of the brain have been suggested to have stronger affiliations with specific activities (e.g., language on the left hemisphere and spatial orientation on the right hemisphere) (Gazzaniga et al. 1965; Kane and Kane 1979; Sperry 1982). Moreover, it has been suggested that the transfer of training shows an asymmetrical transfer, meaning that the transfer of strength from cross-education is greater in one direction over the other (e.g., right to left limb) (Parlow and Kinsbourne 1989; Hammond 2002; Farthing 2009). This idea is mainly supported by work from Farthing and colleagues (Farthing et al. 2005), which found that the cross-education of strength was greater when training the dominant limb compared to training the non-dominant limb (i.e., untrained limb resulted in greater changes when dominant limb trained). Based on that study, research on the cross-education of strength has continued to prioritize the training of the dominant limb for the greatest cross-education effect (Farthing et al. 2007; Lee et al. 2009; Dankel et al. 2020). Additionally, numerous review papers have suggested or mentioned that there is a greater cross-education of strength when training the dominant limb over that of the non-dominant limb (Farthing 2009; Farthing and Zehr 2014; Green and Gabriel 2018b). However, two other studies have failed to find this asymmetrical transfer of strength (Coombs et al. 2016; Othman et al. 2019). Therefore, we sought to review the available literature and determine how much evidence there was for this effect.

## Methods

Our search criteria were narrow to ensure that the research question was addressed. That is, a given study had to compare the strength change in an untrained limb when the homologous muscle was trained on the opposite limb. The unilateral training protocol did not have to follow a specific training manipulating frequency, duration, intensity, or type of training because the interest in the results (cross-education of strength) was a training protocol compared between a dominant and non-dominant limb in the same study sample. Additionally, studies were required to include separate training groups of the dominant and non-dominant limb along with a non-exercise control within the same study. The inclusion of a time-match, non-exercise control for each study would increase confidence that the cross-education of strength, if observed, was from the unilateral training. Studies were excluded if it was not written in English, did not include humans, and did not include resistance training. The small number of studies included for this analysis was not due to a lack of searching. As noted in Supplementary Table 1, we reviewed nearly 1,000 papers and provided reasons for their exclusion.

### Search strategy

**Fig. 1:**
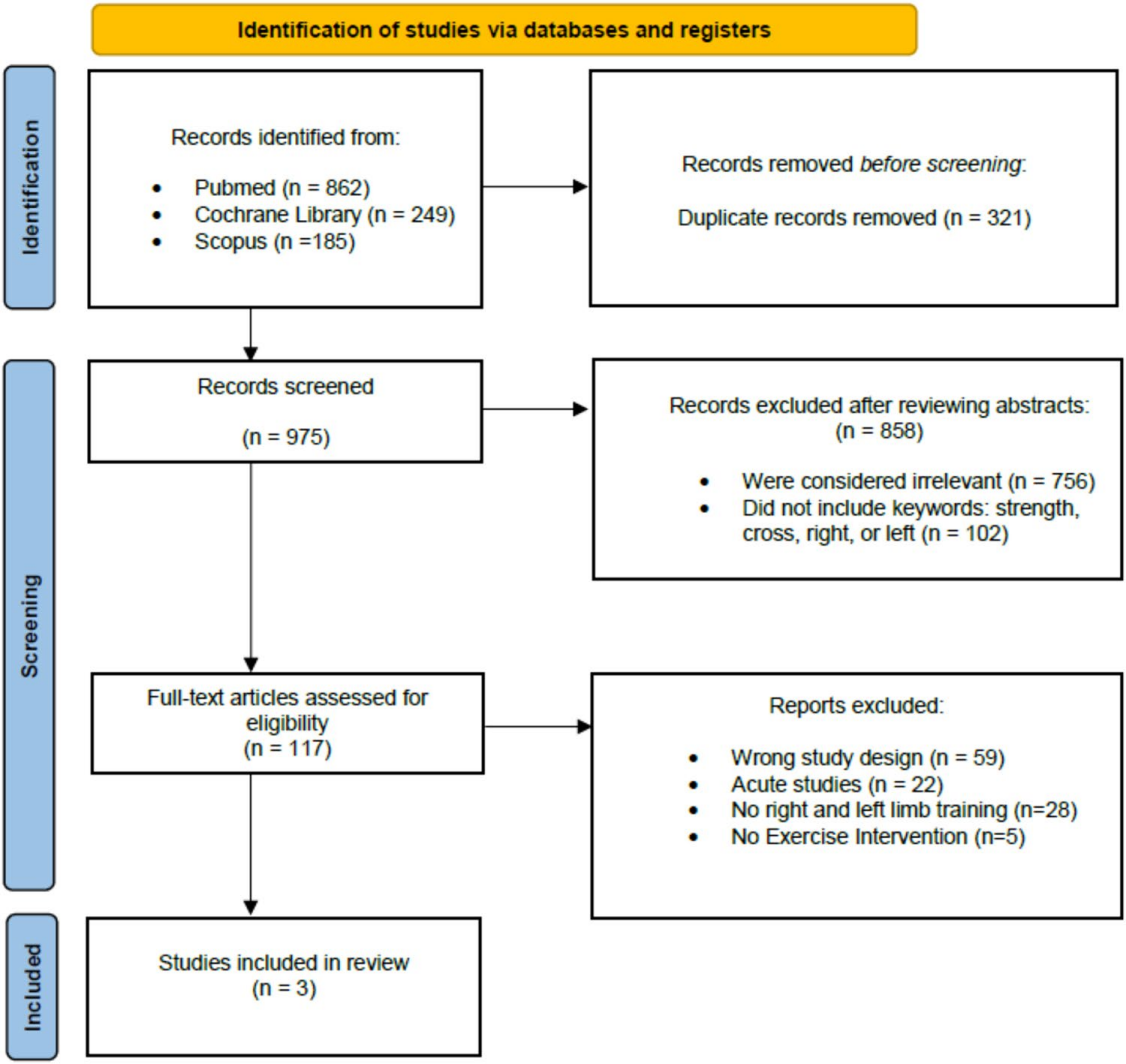
PRISMA flowchart of the studies included for the present meta-analysis

Our search was conducted in line with PRISMA guidelines (Page et al. 2021). The acquisition of studies was completed using Cochrane Library, PubMed, and Scopus from February 2022 to May 2022 with no limitations in publication dates (Fig. 1). Relevant studies were identified using the terms: “directionality cross-education strength training”; “right-hand strength training or left-hand strength training”; “right–left limb training cross-education”; and “dominant limb in cross-education of strength”. Other studies under the references of selected papers that met the inclusion criteria were additionally reviewed. Terms such as “dominant limb training crossover effect of strength” and “dominance in crossover effect strength” were also used, but zero relevant studies were found. The first author (V.W.) completed the search, and V.W., J.S.S., and J.L. independently extracted the data from the included papers. V.W. and J.L. independently conducted the meta-analysis. V.W. and J.S.S. independently evaluated the quality of the included studies using the risk of bias tool 2 (RoBII) (Sterne et al. 2019).

### Data extraction

An editable spreadsheet was prepared to capture the following: author name and year of publication, whether the study included a group training the dominant limb (with non-dominant untrained) and a group training the non-dominant limb (with dominant limb untrained) in the same study, whether the study included a time-matched non-exercise control group, sample size for each group, portion of the body trained, exercise completed, type of strength test utilized, the change scores for each group, and the standard deviation of the change score for each group. If data were reported as standard errors, they were converted to standard deviations by using the appropriate formula (i.e., multiplied by the square root of the sample size). The standard deviation of the difference score between measurements was used when reported directly but was estimated when not reported.

### Statistical analysis

All data were analyzed by multiple investigators as a quality control measure in an effort to maximize accuracy (V.W. and J.L.). Effect sizes were calculated for each study using the mean difference and the standard deviation of the difference (commonly known as Cohen’s dz) (Dankel and Loenneke 2018). If the standard deviation was not reported but an exact *p *value was, then the *t* value was calculated using the inverse of the cumulative distribution function. The* t* value was then used to calculate the change score standard deviation. We normalized the mean difference to the standard deviation of the difference, rather than using pretest and posttest standard deviations, because we were interested in capturing the magnitude of the variability within the intervention itself. If the variability of the change was not provided (and could not be calculated from the data provided), the standard deviation of the change was estimated using the following formula:

$${SD}_{\mathrm{of }change}=\sqrt{{[({\text{SDPretest}})}^{2}+ {({\text{SDPosttest}})}^{2}-(2r\times \mathrm{SDpretest }\times \mathrm{ SDPosttest})].}$$

*SD* represents the standard deviation and* r* represents the correlation coefficient between the pretest and the posttest scores. We used 0.9 as the pre–post correlation, since this correlation on strength tests would be expected to be large (Dankel et al. 2020). The standardized effect size and the standard error of this standardized effect size were computed as follows (Borenstein et al. 2021):

$$Standardized\,ES=\frac{{change}_{exercise}- {change}_{control}}{\sqrt{\frac{\left({N}_{1}-1\right){v}_{1}+\left({N}_{2}-1\right){v}_{2}}{{N}_{1}+{N}_{2}-2}}},$$

$$Standardized\,SE= \sqrt{\frac{{N}_{1}+ {N}_{2}}{{N}_{1}* {N}_{2}}+\frac{{ES}^{2}}{2({N}_{1}+ {N}_{2})}}.$$

*ES* represents the effect size, *N*_1_ represents the sample size of the exercise group, *N*_2_ represents the size of the control group, ${v}_{1}$ represents the variance of the exercise group, ${v}_{2}$ represents the variance of the control group, and *SE* represents the standard error.

All statistics were computed using the robumeta package (version 2.0) and metafor package (version 3.0–2) within R Studio (version 1.4.1717). We implemented these two packages to account for dependency between effect sizes. All studies were weighted using the inverse variance weight and effect sizes are reported in standardized units (Cohen’s *d*). Three separate comparisons were made and visually displayed as forest plots: (1) cross-education effect from dominant to non-dominant limb vs. non-exercise control; (2) cross-education effect from non-dominant to dominant limb vs. non-exercise control; and (3) cross-education from dominant to non-dominant limb vs. cross-education from non-dominant to dominant limb. Forest plots provide point estimates of the individual effect sizes in graphical form as boxes with 95% confidence intervals surrounding each block. The overall effect is included at the bottom of the plot as a diamond with a width equivalent to the confidence interval for the estimated effect (forest.robu function in the robumeta package).

In robumeta, we ran a correlated effects model with small sample corrections. The default correlation was 0.8; however, we also ran sensitivity analysis to determine the effect of rho on tau^2^. We also ran the analysis using the robust function of metafor (Restricted ML). To reduce problems associated with using a normal distribution, we implemented the argument tdist = TRUE with the rma.mv function, which applies the Knapp and Hartung adjustment to the analysis. We included estimates from metafor to include prediction intervals. Prediction intervals provide information of where the effect size of a new study would fall if this study was selected at random from the same population of the studies already included in the meta-analysis.

In the control groups, two limbs on each participant were not trained (i.e., these participants did not train; thus, both limbs were not trained) and, therefore, comparing the cross-education of strength to the intervention groups (dominant-limb vs non-dominant-limb training) was feasible with either limb. Of the three studies included in this review, two of them (Farthing et al. 2005; Othman et al. 2019) reported the cross-education data of the control group from both limbs. However, the Farthing and colleagues (2005) study, randomized the non-exercise control group limbs into a “trained” and “untrained” limb. In other words, it is not known which untrained limb (dominant or non-dominant) from the control group was used. One of the three studies (Coombs et al. 2016) only reported data for the control group without determining the side that the control data was from.

## Results

Our goal was to systematically review the literature with a meta-analysis to estimate the effect. Given the small number of studies available, we provide the effect but note that the estimate is unlikely to be stable. This indicated to us that there was currently not compelling evidence for the asymmetrical transfer in strength. In other words, there might be a greater cross-education effect when the dominant limb is trained, but the available evidence is not consistent. However, three forest plots are used to illustrate the effect from the available literature.

In the original search for this review, a total of 1296 papers were screened (duplicates removed left *n* = 975; listed in Supplementary Table 1). After screening the papers, 117 papers were accessed for eligibility. Exclusion reasons are documented in Fig. 1. Only three studies met the inclusion criteria (Farthing et al. 2005; Coombs et al. 2016; Othman et al. 2019). Two out of three studies were data from resistance training in the upper limb (Farthing et al. 2005; Coombs et al. 2016) and one was from resistance training in the lower limb (Othman et al. 2019). A total of five effect sizes for the changes of strength in the untrained limb were obtained from the collected studies. The number of participants obtained from the selected studies was a total of 104. Limb dominancy was determined based on the Waterloo Handedness Questionnaire for two studies (Farthing et al. 2005; Othman et al. 2019) whereas the other study did not describe how limb dominancy was determined (Coombs et al. 2016). The included studies incorporated wrist (Farthing et al. 2005; Coombs et al. 2016) and leg exercises (Othman et al. 2019) into their intervention. Separated accordingly, there were 35 participants that trained the dominant right side, 35 participants that trained the non-dominant left side, and 34 participants that were in the non-exercise control group. The pre and post data from each study are provided in Table 1. Based on the RoBII (Sterne et al. 2019), the methodological quality of the included studies was considered, overall, low risk (Supplementary Fig. 1). The domains that were evaluated included the randomization process, deviations from the intended interventions, missing outcome data, measurement of the outcome, and the selection of the reported results. No studies were rated with concerns or high risk of bias.

**Table 1:** Pre and post data from the studies included in the analysis

	Cross-education from dominant to non-dominant		Cross-education from non-dominant to dominant	
Farthing 2005 (Isometric ulnar deviation)	Pre	Post	Pre	Post
Training (*n=*12, *n=*13)	12.3 (2.07) Nm	17.1 (5.54) Nm	18.9 (3.6) Nm	20.3 (3.2) Nm
Control (*n=*14)	16.4 (5.6) Nm	18.1 (6.3) Nm		
Coombs 2016 (isotonic wrist flexion/extension)	Pre	Post	Pre	Post
Training (*n=*8, *n=*8)	7.9 (2.9) kg	8.74 (3.1) kg	8.8 (2.7) kg	10.2 (3.6) kg
Control (*n=*7)	8.4 (2.6) kg	8.5 (2.6) kg		
Othman 2019 (isotonic leg press)	Pre	Post	Pre	Post
Training (*n=*15, *n=*14)	54.6 (6.8) kg	87.1 (11.8) kg	50.8 (8.6) kg	81.1 (14.6) kg
Control (*n=*13)	51.9 (5.8) kg	52.1 (4.9) kg		
Othman 2019 (isometric knee extension)	Pre	Post	Pre	Post
Training (*n=*15, *n=*14)	329.3 (69.8) kg	370.3 (67.6) kg	341.7 (55.8) kg	371.4 (57.6) kg
Control (*n=*13)	369.4 (33.1) kg	372.9 (38.2) kg		
Othman 2019 (isometric knee flexion)	Pre	Post	Pre	Post
Training (*n=*15, *n=*14)	167.9 (20.7) kg	181.3 (20.1) kg	182.9 (39.6) kg	185.4 (41.2) kg
Control (*n=*13)	180.5 (19.4) kg	184.2 (14.3) kg		

The data is organized to demonstrate the directionality of this effect (top row), with the same control group data serving as the comparator for dominant to non-dominant and non-dominant to dominant comparisons. Mean values are presented alongside their standard deviations. Each study is listed on the left-hand side with the exercise test

The sample size is indicated in the following order (*n*: dominant to non-dominant, *n*: non-dominant to dominant) below the author’s name

### Cross-education from training the dominant limb

**Fig. 2:**
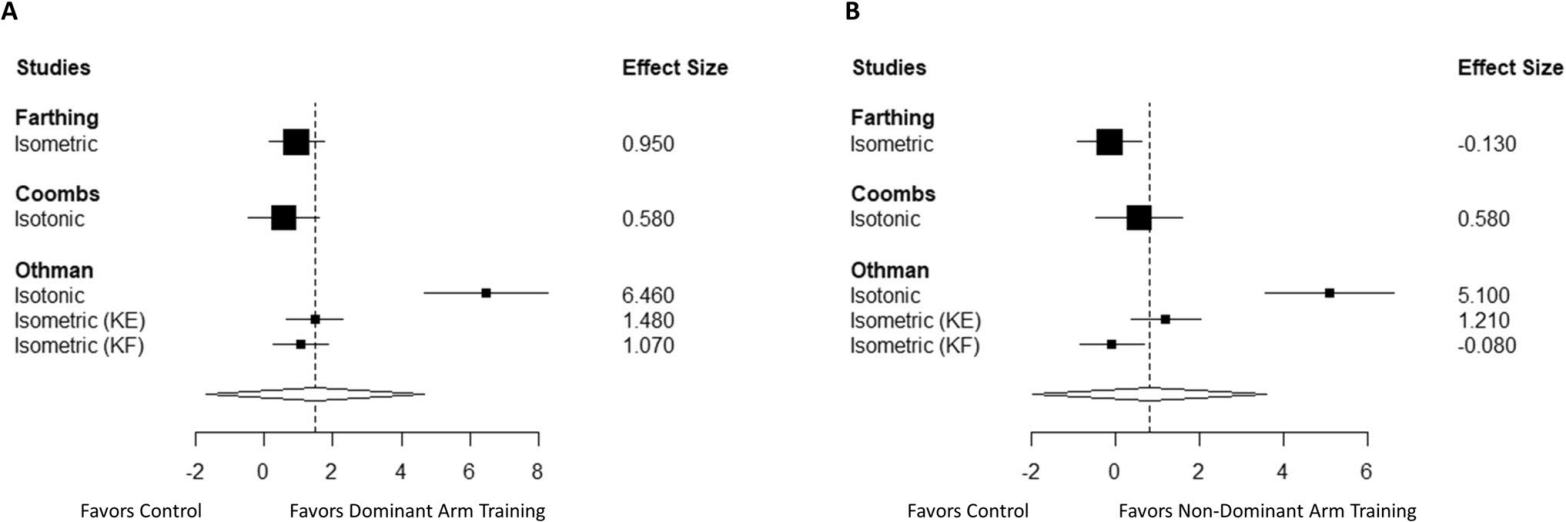
Forest plots of (**A)** resistance training of the dominant limb on strength transfer to the non-dominant (non-training) limb relative to a non-exercise control and (**B**) resistance training of the non-dominant limb on strength transfer to the dominant (non-training) limb relative to a non-exercise control. Values represent Cohen’s *d* (95% confidence interval). Each study is listed on the left side of the plot, with squares representing the effect size for each study surrounded by the 95% confidence interval. The overall effect is included at the bottom of the plot as a diamond with a width equivalent to the confidence interval for the estimated effect. The vertical dotted line denotes the overall point estimate. *KE*: knee extension and *KF*: knee flexion

The overall effect of resistance training of the dominant limb on strength transfer to the non-dominant (untrained) limb relative to a non-exercise control was 1.49 (Cohen’s *d*) with a standard error of 0.73, and a 95% confidence interval of − 1.6–4.6 (Fig. 2A, p = 0.18). The *I*^2^ was 91.93 and the tau^2^ was 3.07. Sensitivity analysis demonstrated that this effect was stable across different values of rho. However, because of the few studies available, this effect should be interpreted with caution. Results were similar when rerunning the analysis using the metafor package [Cohen’s *d* (95% confidence interval) of 2.01 (− 1.2, 5.2)]. The 95% prediction intervals ranged from − 8.0 to 12.0.

### Cross-education from training the non-dominant limb

The overall effect of resistance training the non-dominant limb on strength transfer to the dominant (untrained) limb relative to a non-exercise control was 0.82 (Cohen’s *d*) with a standard error of 0.65, and a 95% confidence interval of − 1.9–3.6 (Fig. 2B, p = 0.332). *I*^2^ was 92.18 and tau^2^ was 2.86. Sensitivity analysis demonstrated that this effect was stable across different values of rho. However, because of the few studies available, this effect should be interpreted with caution. Results were similar when rerunning the analysis using the metafor package [Cohen’s *d* (95% confidence interval) of 1.2 (− 1.5, 4.0)]. The 95% prediction intervals ranged from − 7.7 to 10.3.

### Cross-education: dominant vs. non-dominant

**Fig. 3:**
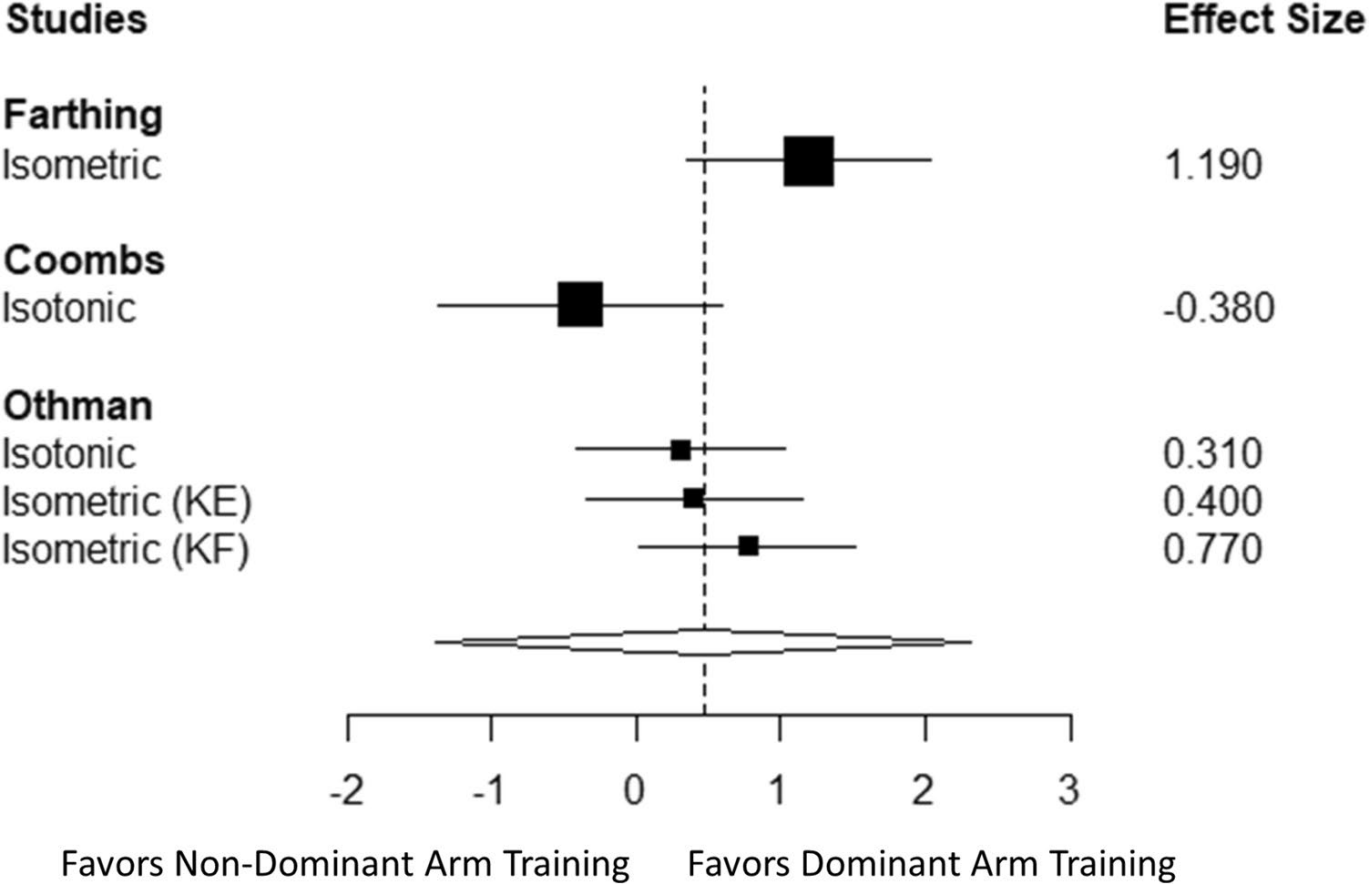
Forest plot of resistance training of the dominant limb on strength transfer to the non-dominant limb relative to the effects of resistance training the non-dominant limb on strength transfer to the dominant (non-training) limb. Values represent Cohen’s *d* (95% confidence interval). Each study is listed on the left side of the plot, with squares representing the effect size for each study surrounded by the 95% confidence interval. The overall effect is included at the bottom of the plot as a diamond with a width equivalent to the confidence interval for the estimated effect. *KE*: knee extension and *KF*: knee flexion

The overall effect of resistance training of the dominant limb on strength transfer to the non-dominant limb (not trained) relative to the effects of resistance training the non-dominant limb on strength transfer to the dominant (non-training) limb was 0.46 (Cohen’s *d*) with a standard error of 0.42, and a 95% confidence interval of − 1.3–2.3 (Fig. 3, *p* = 0.389). *I*^2^ was 65.42 and tau^2^ was 0.36. Sensitivity analysis demonstrated that this effect was stable across different values of rho. However, because of the few studies available, this effect should be interpreted with caution. Results were similar when rerunning the analysis using the metafor package [Cohen’s* d* (95% confidence interval) of 0.46 (-1.2, 2.1)]. The 95% prediction intervals ranged from − 2.5 to 3.4.

## Discussion

The cross-education of strength has been observed many times in the literature (Farthing 2009; Manca et al. 2017). However, contrary to what has been reported several times in review papers (Hammond 2002; Farthing 2009; Farthing and Zehr 2014; Green and Gabriel 2018b), there was little support for the asymmetrical transfer of strength in the literature. The suggestion of training the right-dominant limb for the best cross-educational effect is not currently supported by the available research (although lack of evidence is not evidence of absence). When quantifying the effect using the available literature, we found the estimated effect to include a large degree of uncertainty. Although it is tempting to suggest that the point estimate favors the thesis that greater transfer occurs when the dominant limb is trained, it is important to remember that the point estimate in a random-effect model is an estimation of the mean of the distribution of true effects (Borenstein et al. 2010). Whether or not there is an effect of limb dominancy on the cross-education of strength remains an open question and we provide suggestions for further addressing this question below. The lack of studies available highlights the need to be more cautious when suggesting the claim of asymmetrical transfer.

The only individual study that found an asymmetry of strength transfer was a study by Farthing and colleagues (2005). In that study, they found that there was a greater cross-education of strength when the dominant limb was trained compared with the non-dominant limb. Other investigations were unable to find this effect of limb dominancy (Coombs et al. 2016; Othman et al. 2019). Reasons for this discrepancy are not known but could be related to the type of training (e.g., leg press vs. ulnar deviation), the strength task used (isometric vs. dynamic), and the limbs (arms vs. legs) that are being trained. In addition, one of the papers included in this review was completed in a sample of children between the ages of 10–13 years (Othman et al. 2019). Due to the few numbers of studies found following our search, we opted to include this study in the analysis. However, we do not feel this is a limitation since the effect was compared with a time-matched non-exercise control to capture any effect from maturation. The only other work we found on this topic was a Master’s thesis which also found no impact of limb dominancy on the cross-education in strength (Wend 2017). That project included handgrip training in college-aged women (*n* = 12), but was not included in the analysis since it was not a peer-reviewed published paper.

### Considerations for future research

It is recommended that future work include both a dominant and non-dominant unilateral training program in the same study to directly test this question of asymmetry. Although that was a requirement for this paper, others have drawn conclusions largely from comparing percent changes from individual groups across different studies (Farthing 2009; Manca et al. 2017). In other words, they compare the transfer of strength of the dominant limb that is trained in one investigation to the transfer of strength when the non-dominant limb is trained in a completely separate investigation. This becomes problematic based on comparisons of different samples, exercises, and even different methods of strength measurements from these separate studies. Next, increasing the sample size should also be priority in order to provide more precise estimates for this possible effect. In the previous literature, sample sizes in each study include around 23–42 participants, ranging from 7 to 15 participants within each group. This means the existing literature is only able to detect very large effects. In addition, because much of the current literature prioritizes right limb-dominant individuals, research should also consider possible differences between right- and left-limb dominancy.

## Conclusions

Most of the literature on the cross-education of strength has prioritized training the dominant over the non-dominant limb because of the thought that it results in a greater cross-education for strength. This paper analyzed the currently available literature and was unable to find support for the asymmetry hypothesis. This is not to say that it does not exist; however, additional research with greater sample sizes is needed to better address this research question.

### Supplementary Information

Below is the link to the electronic supplementary material.

Supplementary file1 (PDF 843 KB)

Supplementary file2 (PDF 105 KB)

## Data Availability

The data used for the analysis is provided in text but is also available upon reasonable request.

## Abbreviations

ES: Effect size
Kg: Kilogram
*N*1: Sample size of the exercise group
*N*2: Sample size of the control group
Nm: Newton meter
RoBII: Risk of bias tool 2
SD: Standard deviation
*v*1: Variance of the exercise group
*v*2: Variance of the control group
