# Supplementary material for: Is there evidence for the asymmetrical transfer of strength to an untrained limb?
Source: Eur J Appl Physiol. 2024 Apr 3;124(8):2503–10. doi: 10.1007/s00421-024-05472-9 (PMC11322193; doi:10.1007/s00421-024-05472-9)
Supplement: Supplementary file 1 — Supplementary file1 (PDF 843 KB) [file 421_2024_5472_MOESM1_ESM.pdf]

key	title	year	volume	issue	pages	authors	notes
rayyan-283775620	Cross-education of muscular strength is facilitated by homeostatic plasticity	2017	117	4	665â€677	Frazer, AK and Williams, J and Spittle, M and Kidgell, DJ	RAYYAN-INCLUSION: {"Vickie"=>"Excluded", "jsong6"=>"Excluded"} | RAYYAN-EXCLUSION-REASONS: wrong study design,No dom vs. non-dom comparison
rayyan-283775621	Anodal Transcranial Direct Current Stimulation Prolongs the Cross-education of Strength and Corticomotor Plasticity	2015	47	9	1788â€1797	Hendy, AM and Teo, WP and Kidgell, DJ	RAYYAN-INCLUSION: {"Vickie"=>"Excluded", "jsong6"=>"Excluded"} | RAYYAN-EXCLUSION-REASONS: irrelevant,wrong study design
rayyan-283775622	Anodal tDCS prolongs the cross-education of strength and corticospinal plasticity	2015	10		63	Hendy, AM and Teo, W-P and Kidgell, DJ	RAYYAN-INCLUSION: {"Vickie"=>"Excluded", "jsong6"=>"Excluded"} | RAYYAN-EXCLUSION-REASONS: wrong study design,No dom vs. non-dom comparison
rayyan-283775623	Cross-education of arm muscular strength is unidirectional in right-handed individuals	2005	37	9	1594â€1600	Farthing, JP and Chilibeck, PD and Binsted, G	RAYYAN-INCLUSION: {"Vickie"=>"Included", "jsong6"=>"Included"}
rayyan-283775624	Cross-education of wrist extensor strength is not influenced by non-dominant training in right-handers	2016	116	9	1757â€1769	Coombs, TA and Frazer, AK and Horvath, DM and Pearce, AJ and Howatson, G and Kidgell, DJ	RAYYAN-INCLUSION: {"Vickie"=>"Included", "jsong6"=>"Included"}
rayyan-283775625	Effects of eccentric overload bout on change of direction and performance in soccer players	2015	36	4	308â€314	de Hoyo, M and de la Torre, A and Pradas, F and SaÃ±udo, B and Carrasco, L and Mateo-Cortes, J and DomÃ-nguez-Cobo, S and Fernandes, O and Gonzalo-Skok, O	RAYYAN-INCLUSION: {"Vickie"=>"Excluded", "jsong6"=>"Excluded"} | RAYYAN-EXCLUSION-REASONS: irrelevant
rayyan-283775626	Cross-education of muscle strength is greater with stimulated than voluntary contractions	1999	3	2	205â€219	HortobÃ¡gyi, T and Scott, K and Lambert, J and Hamilton, G and Tracy, J	RAYYAN-INCLUSION: {"Vickie"=>"Excluded", "jsong6"=>"Excluded"} | RAYYAN-EXCLUSION-REASONS: irrelevant,No dom vs. non-dom comparison
rayyan-283775627	Effects of enhanced cutaneous sensory input on interlimb strength transfer of the wrist extensors	2020	8	6		Barss, TS and Klarner, T and Sun, Y and Inouye, K and Zehr, EP	RAYYAN-INCLUSION: {"Vickie"=>"Excluded", "jsong6"=>"Excluded"} | RAYYAN-EXCLUSION-REASONS: wrong study design,No dom vs. non-dom comparison
rayyan-283775628	REFOCUS-PULSAR recovery-oriented practice training in specialist mental health care: a stepped-wedge cluster randomised controlled trial	2019	6	2	103â€114	Meadows, G and Brophy, L and Shawyer, F and Enticott, JC and Fossey, E and Thornton, CD and Weller, PJ and Wilson-Evered, E and Edan, V and Slade, M	RAYYAN-INCLUSION: {"Vickie"=>"Excluded", "jsong6"=>"Excluded"} | RAYYAN-EXCLUSION-REASONS: irrelevant
rayyan-283775629	No evidence of neural adaptations following chronic unilateral isometric training of the intrinsic muscles of the hand: a randomized controlled study	2016	116	10	1993â€2005	Manca, A and Ginatempo, F and Cabboi, MP and Mercante, B and Ortu, E and Dragone, D and De Natale, ER and Dvir, Z and Rothwell, JC and Deriu, F	RAYYAN-INCLUSION: {"Vickie"=>"Excluded", "jsong6"=>"Excluded"} | RAYYAN-EXCLUSION-REASONS: irrelevant,No dom vs. non-dom comparison
rayyan-283775630	Food Safety Knowledge, Attitude, and Practice of College Students, Ethiopia, 2019: a Cross-Sectional Study	2021	2021			Azanaw, J and Dagne, H and Andualem, Z and Adane, T	RAYYAN-INCLUSION: {"Vickie"=>"Excluded", "jsong6"=>"Excluded"} | RAYYAN-EXCLUSION-REASONS: irrelevant
rayyan-283775631	Influence of a training protocol with vibrating platform of different amplitudes on postural balance, gait speed, muscle strength and risk of falls in the elderly: randomized crossover trial	2019				RBR-2hpjjk,	RAYYAN-INCLUSION: {"Vickie"=>"Excluded", "jsong6"=>"Excluded"} | RAYYAN-EXCLUSION-REASONS: irrelevant
rayyan-283775632	Knowledge, attitudes and use of anabolic-androgenic steroids among male gym users: a community based survey in Riyadh, Saudi Arabia	2018				Alharbi, FF and Gamaleddin, I and Alharbi, SF and Almodayfer, O and Allohidan, F and Alghobain, M and Arafah, A and Al-Surimi, K	RAYYAN-INCLUSION: {"Vickie"=>"Excluded", "jsong6"=>"Excluded"} | RAYYAN-EXCLUSION-REASONS: irrelevant
rayyan-283775633	Menstrual health interventions to improve education and health outcomes among Ugandan students	2021				ISRCTN45461276,	RAYYAN-INCLUSION: {"Vickie"=>"Excluded", "jsong6"=>"Excluded"} | RAYYAN-EXCLUSION-REASONS: irrelevant
rayyan-283775634	Effects of Hamstring Training in Different Modes on Stabilizing Knee Joints With Anterior Drawer Laxity	2010				NCT01170546,	RAYYAN-INCLUSION: {"Vickie"=>"Excluded", "jsong6"=>"Excluded"} | RAYYAN-EXCLUSION-REASONS: irrelevant,wrong study design
rayyan-283775635	Comparison of a training videogame with conventional physiotherapy on postural control and quality of life of patients with Parkinson's disease	2016				RBR-27kqv5,	RAYYAN-INCLUSION: {"Vickie"=>"Excluded", "jsong6"=>"Excluded"} | RAYYAN-EXCLUSION-REASONS: irrelevant
rayyan-283775636	Minimal versus specialist equipment in the delivery of pulmonary rehabilitation: protocol for a non-inferiority randomised controlled trial	2021	11	10		Nolan, CM and Walsh, JA and Patel, S and Barker, RE and Polgar, O and Maddocks, M and Gao, W and Wilson, R and Fiorentino, F and Man, W	RAYYAN-INCLUSION: {"Vickie"=>"Excluded", "jsong6"=>"Excluded"} | RAYYAN-EXCLUSION-REASONS: irrelevant
rayyan-283775637	Quality of Life in Motion: A combined physical exercise and psychosocial training program to improve physical fitness in children with cancer	2008				NTR1531,	RAYYAN-INCLUSION: {"Vickie"=>"Excluded", "jsong6"=>"Excluded"} | RAYYAN-EXCLUSION-REASONS: irrelevant
rayyan-283775638	ACTIVE EARLY LEARNING (AEL): evaluation of a physical literacy professional development and curriculum support program in early learning centres	2019				ACTRN12619000638134,	RAYYAN-INCLUSION: {"Vickie"=>"Excluded", "jsong6"=>"Excluded"} | RAYYAN-EXCLUSION-REASONS: irrelevant
rayyan-283775639	Effects of Exercise During Pregnancy on Maternal and Child Health: a Randomized Clinical Trial	2014				NCT02148965,	RAYYAN-INCLUSION: {"Vickie"=>"Excluded", "jsong6"=>"Excluded"} | RAYYAN-EXCLUSION-REASONS: irrelevant
rayyan-283775640	Low Carbohydrate High Fat Ketogenic vs. Non-ketogenic Diet	2021				NCT04744558,	RAYYAN-INCLUSION: {"Vickie"=>"Excluded", "jsong6"=>"Excluded"} | RAYYAN-EXCLUSION-REASONS: nutrition,irrelevant
rayyan-283775641	The Effect of Pre-operative Pelvic Floor Muscle Exercise on Surgical Outcomes in Women With Stress Urinary Incontinence	2012				NCT01602107,	RAYYAN-INCLUSION: {"Vickie"=>"Excluded", "jsong6"=>"Excluded"} | RAYYAN-EXCLUSION-REASONS: irrelevant
rayyan-283775642	Utilizing Gaming Mechanics to Optimize Telerehabilitation Adherence in Persons With Stroke	2019				NCT03985761,	RAYYAN-INCLUSION: {"Vickie"=>"Excluded", "jsong6"=>"Excluded"} | RAYYAN-EXCLUSION-REASONS: irrelevant
rayyan-283775643	Interdisciplinary Pressure Management & Mobility Program as an Alternative to Usual Care: A Pilot Study	2008				NCT00796042,	RAYYAN-INCLUSION: {"Vickie"=>"Excluded", "jsong6"=>"Excluded"} | RAYYAN-EXCLUSION-REASONS: irrelevant
rayyan-283775644	Exercise and Nutritional Supplementation	2018				NCT03536871,	RAYYAN-INCLUSION: {"Vickie"=>"Excluded", "jsong6"=>"Excluded"} | RAYYAN-EXCLUSION-REASONS: nutrition,irrelevant
rayyan-283775645	Angles of Knee and Hip Joints for Optimization of Neuromuscular Electrical Stimulation of the Quadriceps Femoris Muscle	2019				NCT03822221,	RAYYAN-INCLUSION: {"Vickie"=>"Excluded", "jsong6"=>"Excluded"} | RAYYAN-EXCLUSION-REASONS: irrelevant
rayyan-283775646	Reducing Racism and Discrimination in Schools: Comprehensive Prevention Services	2022				NCT05189769,	RAYYAN-INCLUSION: {"Vickie"=>"Excluded", "jsong6"=>"Excluded"} | RAYYAN-EXCLUSION-REASONS: irrelevant
rayyan-283775647	Back Squat Exercise Treatment for Low Back Pain: Clinical Trial	2020				NCT04691258,	RAYYAN-INCLUSION: {"Vickie"=>"Excluded", "jsong6"=>"Excluded"} | RAYYAN-EXCLUSION-REASONS: irrelevant,wrong study design
rayyan-283775648	Evaluation of Tipping Point	2019				NCT03965273,	RAYYAN-INCLUSION: {"Vickie"=>"Excluded", "jsong6"=>"Excluded"} | RAYYAN-EXCLUSION-REASONS: irrelevant
rayyan-283775649	A High-PRotein Mediterranean Diet and Resistance Exercise for Cardiac Rehabilitation: a Pilot Randomised Controlled Trial	2020				NCT04272073,	RAYYAN-INCLUSION: {"Vickie"=>"Excluded", "jsong6"=>"Excluded"} | RAYYAN-EXCLUSION-REASONS: nutrition,irrelevant
rayyan-283775650	The Effectiveness of Health Facility-based and Community-based Care for Tuberculosis	2009				NCT00939419,	RAYYAN-INCLUSION: {"Vickie"=>"Excluded", "jsong6"=>"Excluded"} | RAYYAN-EXCLUSION-REASONS: irrelevant
rayyan-283775651	Pilot of a Prebiotic and Probiotic Trial in Young Infants With Severe Acute Malnutrition	2018				NCT03666572,	RAYYAN-INCLUSION: {"Vickie"=>"Excluded", "jsong6"=>"Excluded"} | RAYYAN-EXCLUSION-REASONS: irrelevant
rayyan-283775652	Grip Strength Is Associated with Cognitive Performance in Schizophrenia and the General Population: A UK Biobank Study of 476559 Participants	2018	44	4		Firth, J., Stubbs, B., Vancampfort, D., Firth, J.A., Large, M., Rosenbaum, S., Hallgren, M., Ward, P.B., Sarris, J., Yung, A.R.	RAYYAN-INCLUSION: {"Vickie"=>"Excluded", "jsong6"=>"Excluded"} | RAYYAN-EXCLUSION-REASONS: irrelevant
rayyan-283775654	Effect of Unilateral E-stim on strength in a contralateral untrained limb after bilateral strength training	2013	27			LoRusso, S and Barr, N and Hanik, W and Hazen, J and Strittmatter, A and Tocco, T	RAYYAN-INCLUSION: {"Vickie"=>"Excluded", "jsong6"=>"Excluded"} | RAYYAN-EXCLUSION-REASONS: wrong study design,no right and left limb training
rayyan-283775655	Cross Education in Patients With Sensorimotor Impairment	2016				NCT02795507,	RAYYAN-INCLUSION: {"Vickie"=>"Excluded", "jsong6"=>"Excluded"} | RAYYAN-EXCLUSION-REASONS: irrelevant,wrong study design
rayyan-283775656	Mirror Training Augments the Cross-education of Strength and Affects Inhibitory Paths	2016	48	6	1001â€1013	Zult, T and Goodall, S and Thomas, K and Solnik, S and HortobÃ¡gyi, T and Howatson, G	RAYYAN-INCLUSION: {"Vickie"=>"Excluded", "jsong6"=>"Excluded"} | RAYYAN-EXCLUSION-REASONS: wrong study design,No dom vs. non-dom comparison
rayyan-283775658	Interhemispheric plasticity in humans	2011	43	7	1188â€1199	HortobÃ¡gyi, T and Richardson, SP and Lomarev, M and Shamim, E and Meunier, S and Russman, H and Dang, N and Hallett, M	RAYYAN-INCLUSION: {"Vickie"=>"Excluded", "jsong6"=>"Excluded"} | RAYYAN-EXCLUSION-REASONS: No dom vs. non-dom comparison,no right and left limb training
rayyan-283775659	Effct of Rehabilitation in Stroke Recovery	2016				IRCT2016102430477N1,	RAYYAN-INCLUSION: {"Vickie"=>"Excluded", "jsong6"=>"Excluded"} | RAYYAN-EXCLUSION-REASONS: irrelevant
rayyan-283775660	28th Annual Meeting of the European Association of Cardiothoracic Anaesthesiologists, EACTA 2013	2014	17	2	233â€234		RAYYAN-INCLUSION: {"Vickie"=>"Excluded", "jsong6"=>"Excluded"} | RAYYAN-EXCLUSION-REASONS: irrelevant
rayyan-283775661	Using Modified Equipment in Field Hockey Leads to Positive Transfer of Learning Effect	2021	12			Brocken, J.E.A., van der Kamp, J., Lenior, M., Savelsbergh, G.J.P.	RAYYAN-INCLUSION: {"Vickie"=>"Excluded", "jsong6"=>"Excluded"} | RAYYAN-EXCLUSION-REASONS: irrelevant
rayyan-283775662	Risk of repetitive movement in the upper limbs of workers. Personal and labor factors [Riesgo por movimiento repetitivo en los miembros superiores de trabajadores. Factores personales y laborales]	2020	39	6		Mendinueta-MartÃ-nez, M., Herazo-BeltrÃ¡n, Y., Toro-GarcÃ-a, L., Cetares-Barrios, R., Ortiz-Berrio, K., Ricardo-Caiafa, Y.	RAYYAN-INCLUSION: {"Vickie"=>"Excluded", "jsong6"=>"Excluded"} | RAYYAN-EXCLUSION-REASONS: irrelevant,foreign language
rayyan-283775663	Interlimb transfer and generalisation of learning in the context of persistent failure to accomplish a visuomotor task	2019	237	4		Bolton, D.A.E., Buick, A.R., Carroll, T.J., Carson, R.G.	RAYYAN-INCLUSION: {"Vickie"=>"Excluded", "jsong6"=>"Excluded"} | RAYYAN-EXCLUSION-REASONS: irrelevant
rayyan-283775664	Ipsilateral corticomotor responses are confined to the homologous muscle following cross-education of muscular strength	2018	43	1		Mason, J., Frazer, A.K., Horvath, D.M., Pearce, A.J., Avela, J., Howatson, G., Kidgell, D.J.	RAYYAN-INCLUSION: {"Vickie"=>"Excluded", "jsong6"=>"Excluded"} | RAYYAN-EXCLUSION-REASONS: wrong study design,No dom vs. non-dom comparison
rayyan-283775665	Using virtual reality to transfer motor skill knowledge from one hand to another	2017	2017	127		Ossmy, O., Mukamel, R.	RAYYAN-INCLUSION: {"Vickie"=>"Excluded", "jsong6"=>"Excluded"} | RAYYAN-EXCLUSION-REASONS: irrelevant
rayyan-283775666	Cross-activation of the motor cortex during unilateral contractions of the quadriceps	2017	11			Hendy, A.M., Chye, L., Teo, W.-P.	RAYYAN-INCLUSION: {"Vickie"=>"Excluded", "jsong6"=>"Excluded"} | RAYYAN-EXCLUSION-REASONS: irrelevant,wrong study design
rayyan-283775667	Interlimb transfer of motor skill learning during walking: No evidence for asymmetric transfer	2017	56			Krishnan, C., Ranganathan, R., Tetarbe, M.	RAYYAN-INCLUSION: {"Vickie"=>"Excluded", "jsong6"=>"Excluded"} | RAYYAN-EXCLUSION-REASONS: irrelevant,wrong study design
rayyan-283775668	Structural and functional cortical connectivity mediating ross education of motor function	2017	37	10		Ruddy, K.L., Leemans, A., Woolley, D.G., Wenderoth, N., Carson, R.G.	RAYYAN-INCLUSION: {"Vickie"=>"Excluded", "jsong6"=>"Excluded"} | RAYYAN-EXCLUSION-REASONS: irrelevant,wrong study design
rayyan-283775670	Neural adaptations associated with interlimb transfer in a ballistic wrist flexion task	2016	10			Ruddy, K.L., Rudolf, A.K., Kalkman, B., King, M., Daffertshofer, A., Carroll, T.J., Carson, R.G.	RAYYAN-INCLUSION: {"Vickie"=>"Excluded", "jsong6"=>"Excluded"} | RAYYAN-EXCLUSION-REASONS: irrelevant,no right and left limb training
rayyan-283775671	Increased cross-education of muscle strength and reduced corticospinal inhibition following eccentric strength training	2015	300			Kidgell, D.J., Frazer, A.K., Rantalainen, T., Ruotsalainen, I., Ahtiainen, J., Avela, J., Howatson, G.	RAYYAN-INCLUSION: {"Vickie"=>"Excluded", "jsong6"=>"Excluded"} | RAYYAN-EXCLUSION-REASONS: No dom vs. non-dom comparison,no right and left limb training
rayyan-283775672	Surgical leg rotation: Cortical neuroplasticity assessed through brain mapping using transcranial magnetic stimulation	2014	37	4		Tesio, L., Benedetti, M.G., Rota, V., Manfrini, M., Perucca, L., Caronni, A.	RAYYAN-INCLUSION: {"Vickie"=>"Excluded", "jsong6"=>"Excluded"} | RAYYAN-EXCLUSION-REASONS: irrelevant
rayyan-283775673	Structural brain changes after 4 wk of unilateral strength training of the lower limb	2013	115	2		Palmer, H.S., HÃ¥berg, A.K., Fimland, M.S., Solstad, G.M., Moe Iversen, V., Hoff, J., Helgerud, J., Eikenes, L.	RAYYAN-INCLUSION: {"Vickie"=>"Excluded", "jsong6"=>"Excluded"} | RAYYAN-EXCLUSION-REASONS: No dom vs. non-dom comparison,no right and left limb training
rayyan-283775674	Neural pathways mediating cross education of motor function	2013				Ruddy, K.L., Carson, R.G.	RAYYAN-INCLUSION: {"Vickie"=>"Excluded", "jsong6"=>"Excluded"} | RAYYAN-EXCLUSION-REASONS: irrelevant,wrong study design
rayyan-283775676	Changes in functional magnetic resonance imaging cortical activation with cross education to an immobilized limb	2011	43	8		Farthing, J.P., Krentz, J.R., Magnus, C.R.A., Barss, T.S., Lanovaz, J.L., Cummine, J., Esopenko, C., Sarty, G.E., Borowsky, R.	RAYYAN-INCLUSION: {"Vickie"=>"Excluded", "jsong6"=>"Excluded"} | RAYYAN-EXCLUSION-REASONS: irrelevant,wrong study design
rayyan-283775677	Effects of cross-education on the muscle after a period of unilateral limb immobilization using a shoulder sling and swathe	2010	109	6		Magnus, C.R.A., Barss, T.S., Lanovaz, J.L., Farthing, J.P.	RAYYAN-INCLUSION: {"Vickie"=>"Excluded", "jsong6"=>"Excluded"} | RAYYAN-EXCLUSION-REASONS: No dom vs. non-dom comparison,no right and left limb training
rayyan-283775678	Cross-education of strength depends on limb dominance: Implications for theory and application	2009	37	4		Farthing, J.P.	RAYYAN-INCLUSION: {"Vickie"=>"Excluded", "jsong6"=>"Excluded"} | RAYYAN-EXCLUSION-REASONS: wrong study design,no right and left limb training
rayyan-283775679	Unilateral strength training increases voluntary activation of the opposite untrained limb	2009	120	4		Lee, M., Gandevia, S.C., Carroll, T.J.	RAYYAN-INCLUSION: {"Vickie"=>"Excluded", "jsong6"=>"Excluded"} | RAYYAN-EXCLUSION-REASONS: No dom vs. non-dom comparison,no right and left limb training
rayyan-283775680	Strength training the free limb attenuates strength loss during unilateral immobilization	2009	106	3		Farthing, J.P., Krentz, J.R., Magnus, C.R.A.	RAYYAN-INCLUSION: {"Vickie"=>"Excluded", "jsong6"=>"Excluded"} | RAYYAN-EXCLUSION-REASONS: wrong study design,No dom vs. non-dom comparison
rayyan-283775681	Bilateral effects of unilateral electromyostimulation or voluntary isometric training on dorsiflexion strength and muscle activation	2008	23	9		Yu, J., Zhou, S., Huang, L., Liu, J., Hu, X.	RAYYAN-INCLUSION: {"Vickie"=>"Excluded", "jsong6"=>"Excluded"} | RAYYAN-EXCLUSION-REASONS: irrelevant,No dom vs. non-dom comparison
rayyan-283775682	Neuro-physiological adaptations associated with cross-education of strength	2007	20	2		Farthing, J.P., Borowsky, R., Chilibeck, P.D., Binsted, G., Sarty, G.E.	RAYYAN-INCLUSION: {"Vickie"=>"Excluded", "jsong6"=>"Excluded"} | RAYYAN-EXCLUSION-REASONS: wrong study design,No dom vs. non-dom comparison
rayyan-283775684	Side-to-side comparisons of bone mineral density in upper and lower limbs of collegiate athletes	2002	16	4		McClanahan, B.S., Harmon-Clayton, K., Ward, K.D., Klesges, R.C., Vukadinovich, C.M., Cantler, E.D.	RAYYAN-INCLUSION: {"Vickie"=>"Excluded", "jsong6"=>"Excluded"} | RAYYAN-EXCLUSION-REASONS: irrelevant
rayyan-283775685	Age related neural adaptation following short term resistance training in women	2001	41	3		Bemben, M.G., Murphy, R.E.	RAYYAN-INCLUSION: {"Vickie"=>"Excluded", "jsong6"=>"Excluded"} | RAYYAN-EXCLUSION-REASONS: wrong study design,No dom vs. non-dom comparison
rayyan-283775686	Cross transfer effects of muscular training on blood flow in the ipsilateral and contralateral forearms	1983	51	3		Yasuda, Y., Miyamura, M.	RAYYAN-INCLUSION: {"Vickie"=>"Excluded", "jsong6"=>"Excluded"} | RAYYAN-EXCLUSION-REASONS: irrelevant,wrong study design
rayyan-283775687	Studies in cross education. III. Kinaesthetic learning of an irregular pattern	1934	17	5		Cook, T.W.	RAYYAN-INCLUSION: {"Vickie"=>"Excluded", "jsong6"=>"Excluded"} | RAYYAN-EXCLUSION-REASONS: irrelevant,wrong study design
rayyan-283775688	study comparing right and left hand laryngoscopy in manikin	2021				CTRI/2021/07/035021,	RAYYAN-INCLUSION: {"Vickie"=>"Excluded", "jsong6"=>"Excluded"} | RAYYAN-EXCLUSION-REASONS: irrelevant
rayyan-283775689	Asymmetric somatosensory activation with right- vs left-hand stimulation: a positron emisson tomographic study	1989	483	2	355â€360	Yoshii, F and Ginsberg, MD and Kelley, RE and Chang, JY and Barker, WW and Ingenito, G and Apicella, AM and Globus, MY-T and Duara, R and Boothe, TE	RAYYAN-INCLUSION: {"Vickie"=>"Excluded", "jsong6"=>"Excluded"} | RAYYAN-EXCLUSION-REASONS: irrelevant,no right and left limb training
rayyan-283775690	Sensory discrimination in right versus left hand in visually blind and its association to braille reading	2013	57	5	53â€54	Choraghe Rohan, P and Chitra, P	RAYYAN-INCLUSION: {"Vickie"=>"Excluded", "jsong6"=>"Excluded"} | RAYYAN-EXCLUSION-REASONS: irrelevant,no right and left limb training
rayyan-283775691	Variations of brain activities of acupuncture to TE5 of left hand in normal subjects	2011	39	4	673â€686	Hsu, SF and Chen, CY and Ke, MD and Huang, CH and Sun, YT and Lin, JG	RAYYAN-INCLUSION: {"Vickie"=>"Excluded", "jsong6"=>"Excluded"} | RAYYAN-EXCLUSION-REASONS: irrelevant
rayyan-283775692	Left-hand somatosensory stimulation combined with visual scanning training in rehabilitation for post-stroke hemineglect: a randomised, double-blind study	2009	19	3	364â€382	Polanowska, K and SeniÃ³w, J and Paprot, E and LeÅ›niak, M and CzÅ‚onkowska, A	RAYYAN-INCLUSION: {"Vickie"=>"Excluded", "jsong6"=>"Excluded"} | RAYYAN-EXCLUSION-REASONS: irrelevant,no right and left limb training
rayyan-283775693	TEACHING THE Ã¢â‚¬Å“FOUR-POSITION METHODÃ¢â‚¬ï¿½ MAKES BEGINNER ENDOSCOPISTS AWARE OF SPATIAL POSITIONING OF THE LEFT HAND TO MASTER UPPER GASTROINTESTINAL ENDOSCOPY	2019	156	6	Sâ€961â€[illegible]	Sugimoto, K and Osawa, S	RAYYAN-INCLUSION: {"Vickie"=>"Excluded", "jsong6"=>"Excluded"} | RAYYAN-EXCLUSION-REASONS: irrelevant
rayyan-283775694	One hand is better than two: motor extinction of left hand advantage in unilateral neglect	1994	32	1	1â€1	Robertson, IH and North, NT	RAYYAN-INCLUSION: {"Vickie"=>"Excluded", "jsong6"=>"Excluded"} | RAYYAN-EXCLUSION-REASONS: irrelevant
rayyan-283775695	The effects of left-hand exercises on beginning upper string performers' intonation and facility	2004			169 p	Mongeon, J	RAYYAN-INCLUSION: {"Vickie"=>"Excluded", "jsong6"=>"Excluded"} | RAYYAN-EXCLUSION-REASONS: irrelevant,no right and left limb training
rayyan-283775696	The effects of robot-assisted left-hand training on hemispatial neglect in older patients with chronic stroke: a pilot and randomized controlled trial	2021	100	9	e24781	Park, JH	RAYYAN-INCLUSION: {"Vickie"=>"Excluded", "jsong6"=>"Excluded"} | RAYYAN-EXCLUSION-REASONS: irrelevant
rayyan-283775697	Initiating word-finding trials with left-hand movement during anomia treatment remaps frontal language and executive mechanisms	2014	28	4	NP16	Towler, S and Benjamin, M and McGregor, K and Harnish, S and Garcia, A and Zlatar, Z	RAYYAN-INCLUSION: {"Vickie"=>"Excluded", "jsong6"=>"Excluded"} | RAYYAN-EXCLUSION-REASONS: irrelevant
rayyan-283775698	Noninvasive brain stimulation for treatment of right- and left-handed poststroke aphasics	2013	36	5	363â€372	Heiss, WD and Hartmann, A and Rubi-Fessen, I and Anglade, C and Kracht, L and Kessler, J and Weiduschat, N and Rommel, T and Thiel, A	RAYYAN-INCLUSION: {"Vickie"=>"Excluded", "jsong6"=>"Excluded"} | RAYYAN-EXCLUSION-REASONS: irrelevant,wrong study design
rayyan-283775699	Computer mouse operation: is the left-handed user disadvantaged?	1997	28	4	245â€248	Hoffmann, ER and Chang, WY and Yim, KY	RAYYAN-INCLUSION: {"Vickie"=>"Excluded", "jsong6"=>"Excluded"} | RAYYAN-EXCLUSION-REASONS: irrelevant
rayyan-283775700	Ipsilateral Sacroiliac dysfunction among (Right/Left-hand drive) taxi drivers and effect of sacroiliac belt on muscular activity during sit to unilateral stand task	2018				IRCT20090301001722N17,	RAYYAN-INCLUSION: {"Vickie"=>"Excluded", "jsong6"=>"Excluded"} | RAYYAN-EXCLUSION-REASONS: irrelevant
rayyan-283775701	Effects of repetitive handgrip training on endurance, specificity, and cross-education	1999	79	5	467â€475	Shields, RK and Leo, KC and Messaros, AJ and Somers, VK	RAYYAN-INCLUSION: {"Vickie"=>"Excluded", "jsong6"=>"Excluded"} | RAYYAN-EXCLUSION-REASONS: No dom vs. non-dom comparison,no right and left limb training
rayyan-283775702	Proprioceptive recalibration in the right and left hands following abrupt visuomotor adaptation	2012	217	2	187â€196	Salomonczyk, D and Henriques, DY and Cressman, EK	RAYYAN-INCLUSION: {"Vickie"=>"Excluded", "jsong6"=>"Excluded"} | RAYYAN-EXCLUSION-REASONS: irrelevant
rayyan-283775703	Surgical skills acquisition among left-handed trainees-true inferiority or unfair assessment: a preliminary study	2013	70	2	237â€242	Lee, JY and Mucksavage, P and McDougall, EM	RAYYAN-INCLUSION: {"Vickie"=>"Excluded", "jsong6"=>"Excluded"} | RAYYAN-EXCLUSION-REASONS: irrelevant
rayyan-283775704	Cued Dichotic Listening with Right-Handed, Left-Handed, Bilingual and Learning-Disabled Children	1987			10	Obrzut, and John, E and And, Others	RAYYAN-INCLUSION: {"Vickie"=>"Excluded", "jsong6"=>"Excluded"} | RAYYAN-EXCLUSION-REASONS: irrelevant
rayyan-283775705	Correlation between acupuncture and left-handed patients after acute stroke	2008			150	Mutasem, A and Xiao, B and Zi, X and Song, Z and Tu, Q	RAYYAN-INCLUSION: {"Vickie"=>"Excluded", "jsong6"=>"Excluded"} | RAYYAN-EXCLUSION-REASONS: irrelevant
rayyan-283775706	Movement related slow potentials. II. A contrast between finger and foot movements in left-handed subjects	1985	60	2	135â€145	Brunia, CH and Voorn, FJ and Berger, MP	RAYYAN-INCLUSION: {"Vickie"=>"Excluded", "jsong6"=>"Excluded"} | RAYYAN-EXCLUSION-REASONS: irrelevant
rayyan-283775708	Comparison of pressure pain threshold, grip strength,dexterity and touch pressure of dominant and non-dominant hands within and between right-and left-handed subjects	2004	19	6	874â€878	Ozcan, A and Tulum, Z and Pinar, L and BaÅŸkurt, F	RAYYAN-INCLUSION: {"Vickie"=>"Excluded", "jsong6"=>"Excluded"} | RAYYAN-EXCLUSION-REASONS: irrelevant,wrong study design
rayyan-283775709	Recognition and recall of dichotically presented verbal stimuli by right- and left-handed persons	1967	5		119â€126	Curry, FK and Rutherford, DR	RAYYAN-INCLUSION: {"Vickie"=>"Excluded", "jsong6"=>"Excluded"} | RAYYAN-EXCLUSION-REASONS: irrelevant
rayyan-283775710	The effect of left-handed rotation carnitine on serum lipid metabolism during short-term parenteral nutrition	1998	5	4	225â€226	Cheng, SZ and Lin, YK and Xu, DP	RAYYAN-INCLUSION: {"Vickie"=>"Excluded", "jsong6"=>"Excluded"} | RAYYAN-EXCLUSION-REASONS: irrelevant
rayyan-283775711	Are right handed patients more likely to benefit from acupuncture anti-emesis than left handed?	1988	157	5	172â€173	Fitzpatrick, KTJ and Dunde JWet, al	RAYYAN-INCLUSION: {"Vickie"=>"Excluded", "jsong6"=>"Excluded"} | RAYYAN-EXCLUSION-REASONS: irrelevant,wrong study design
rayyan-283775712	Effects of Combined Resistance Plus Aerobic Training on Body Composition, Muscle Strength, Aerobic Capacity, and Renal Function in Kidney Transplantation Subjects	2019				Lima, PS and de Campos, AS and de Faria Neto, O and Ferreira, TCA and Amorim, CEN and Stone, WJ and Prestes, J and Garcia, AMC and Urtado, CB	RAYYAN-INCLUSION: {"Vickie"=>"Excluded", "jsong6"=>"Excluded"} | RAYYAN-EXCLUSION-REASONS: irrelevant
rayyan-283775713	Vitamin D receptor polymorphisms are associated with baseline muscle strength and response to strength training	2017	35			Cohen, J and Attenasio, A and Swenson, S and Gu, A and Gordish-Dressman, H and Floor, M and Harmon, B and Hoffman, E and Hittel, D and Knoblach, S and Devaney, J and Tosi, LL	RAYYAN-INCLUSION: {"Vickie"=>"Excluded", "jsong6"=>"Excluded"} | RAYYAN-EXCLUSION-REASONS: wrong study design
rayyan-283775714	Telemedicine applied to kinesiotherapy for hand dysfunction in patients with systemic sclerosis and rheumatoid arthritis: recovery of movement and telemonitoring technology	2014	41	7	1324â€1333	Piga, M and Tradori, I and Pani, D and Barabino, G and DessÃ¬, A and Raffo, L and Mathieu, A	RAYYAN-INCLUSION: {"Vickie"=>"Excluded", "jsong6"=>"Excluded"} | RAYYAN-EXCLUSION-REASONS: irrelevant
rayyan-283775715	Effects of Respiratory Physiotherapy on Postural Control, Balance, Respiratory Functions and Respiratory Muscle Strength	2020				NCT04473872,	RAYYAN-INCLUSION: {"Vickie"=>"Excluded", "jsong6"=>"Excluded"} | RAYYAN-EXCLUSION-REASONS: irrelevant
rayyan-283775716	High-definition transcranial direct current stimulation to both primary motor cortices improves unimanual and bimanual dexterity	2017	643		84â€88	Pixa, NH and Steinberg, F and Doppelmayr, M	RAYYAN-INCLUSION: {"Vickie"=>"Excluded", "jsong6"=>"Excluded"} | RAYYAN-EXCLUSION-REASONS: irrelevant,wrong study design
rayyan-283775717	Effect of Kaempferia parviflora Extract on Physical Fitness of Soccer Players: a Randomized Double-Blind Placebo-Controlled Trial	2015	21		100â€108	Promthep, K and Eungpinichpong, W and Sripanidkulchai, B and Chatchawan, U	RAYYAN-INCLUSION: {"Vickie"=>"Excluded", "jsong6"=>"Excluded"} | RAYYAN-EXCLUSION-REASONS: irrelevant
rayyan-283775718	Eye and hand fatigue in minimal invasive surgery; 2D Vs. 3D: RCT	2015	29		S8	Youssef, A and Elbakbak, WS and Bouhelal, A and Patel, B	RAYYAN-INCLUSION: {"Vickie"=>"Excluded", "jsong6"=>"Excluded"} | RAYYAN-EXCLUSION-REASONS: irrelevant
rayyan-283775719	Dynamics of EEG coherence in right- and left-handed people when falling asleep. II. Analysis of interhemispheric relations	1998	24	1	32â€39	Zhavoronkova, LA and Trofimova, EV	RAYYAN-INCLUSION: {"Vickie"=>"Excluded", "jsong6"=>"Excluded"} | RAYYAN-EXCLUSION-REASONS: irrelevant
rayyan-283775720	Visual half-field differences in masking effects for sequential letter stimuli in the right and left handed	1972	10	1	111â€117	McKeever, WF and Gill, KM	RAYYAN-INCLUSION: {"Vickie"=>"Excluded", "jsong6"=>"Excluded"} | RAYYAN-EXCLUSION-REASONS: irrelevant
rayyan-283775721	Skeletal muscle electrostimulation in chronic pulmonary disease patients with subclinical peripheral atherosclerosis: effects on muscle status and exercise performance	2012	19	1	S131	Alexey Sumin, AN and Nedoseykina, EV and Arhipov, OG	RAYYAN-INCLUSION: {"Vickie"=>"Excluded", "jsong6"=>"Excluded"} | RAYYAN-EXCLUSION-REASONS: irrelevant,no right and left limb training
rayyan-283775722	Characterization of the types of hemispheric response and anxiety level in right-handed and left-handed individuals in various regions of Russia and Latvia	1998	24	1	134â€137	Arsavskis, VV	RAYYAN-INCLUSION: {"Vickie"=>"Excluded", "jsong6"=>"Excluded"} | RAYYAN-EXCLUSION-REASONS: irrelevant
rayyan-283775723	Coherence dynamics of EEG and motor reactions while falling asleep in right-handed and left-handed people. I. Analysis of intrahemispheric correlations	1997	23	6	18â€26	Zhavoronkova, LA and Trofimova, EV	RAYYAN-INCLUSION: {"Vickie"=>"Excluded", "jsong6"=>"Excluded"} | RAYYAN-EXCLUSION-REASONS: irrelevant
rayyan-283775724	Development of a novel supination strength testing method for the upper extremity	2017	35			Brown, BT and Schmidt, CC and Madonna, TJ and Kotsonis, T and Smolinski, PJ and Miller, MC	RAYYAN-INCLUSION: {"Vickie"=>"Excluded", "jsong6"=>"Excluded"} | RAYYAN-EXCLUSION-REASONS: irrelevant
rayyan-283775725	Left- versus right-hand tracking performance by right-handed boys and girls: examination of performance asymmetry	2003	97	3	779â€788	Carey, JR and Comnick, KT and Lojovich, JM and Lindgren, BR	RAYYAN-INCLUSION: {"Vickie"=>"Excluded", "jsong6"=>"Excluded"} | RAYYAN-EXCLUSION-REASONS: irrelevant
rayyan-283775726	Clinical Evaluation of the Performance of a Difluoroethane-based Cyto-selective Cryotherapy to Treat Dark Spots on the Hand in 30 Volunteers	2017				NCT03157427,	RAYYAN-INCLUSION: {"Vickie"=>"Excluded", "jsong6"=>"Excluded"} | RAYYAN-EXCLUSION-REASONS: irrelevant
rayyan-283775727	Hand, space and attentional asymmetries in goal-directed manual aiming	1997	33	2	251â€269	Hodges, NJ and Lyons, J and Cockell, D and Reed, A and Elliott, D	RAYYAN-INCLUSION: {"Vickie"=>"Excluded", "jsong6"=>"Excluded"} | RAYYAN-EXCLUSION-REASONS: irrelevant
rayyan-283775728	Haptic information differentially interferes with visual analysis in reaching-grasping control and in perceptual processes	1998	9	5	887â€891	Gentilucci, M and Daprati, E and Gangitano, M	RAYYAN-INCLUSION: {"Vickie"=>"Excluded", "jsong6"=>"Excluded"} | RAYYAN-EXCLUSION-REASONS: irrelevant
rayyan-283775729	A Comparison of the Effectiveness of 5% Lidocaine Medicated Plaster and EMLA Cream for Decreasing Pain on Venous Cannulation for Fluid Administration	2020				TCTR20200429002,	RAYYAN-INCLUSION: {"Vickie"=>"Excluded", "jsong6"=>"Excluded"} | RAYYAN-EXCLUSION-REASONS: irrelevant
rayyan-283775730	Energy resource depletion, ability perception, and cardiovascular response to behavioral challenge	2002	39	2	182â€187	Wright, RA and Penacerrada, DK	RAYYAN-INCLUSION: {"Vickie"=>"Excluded", "jsong6"=>"Excluded"} | RAYYAN-EXCLUSION-REASONS: irrelevant
rayyan-283775731	Comparison of the effects of high and low frequency repetitive transcranial magnetic stimulation on upper limb function in healthy subjects	2015	101		eS1611	Watanabe, M and Matsunaga, T and Okudera, Y and Sato, M and Hatakeyama, K and Chida, S and Shimada, Y	RAYYAN-INCLUSION: {"Vickie"=>"Excluded", "jsong6"=>"Excluded"} | RAYYAN-EXCLUSION-REASONS: irrelevant
rayyan-283775732	A behavioral manipulation engages right frontal cortex during aphasia therapy	2014	28	6	545â€553	Beniamin. ML and Towler. S and Garcia. A and Park. H and Sudhvadhom. A and Harnish. S and McGregor. KM and Zlatar. Z and Reillv. JJ and Rosenbek. JC and Gonzalez Rothi. LJ and Crosson.	RAYYAN-INCLUSION: {"Vickie"=>"Excluded", "jsong6"=>"Excluded"} | RAYYAN-EXCLUSION-REASONS: irrelevant
rayyan-283775733	The effect of acupressure on anxiety of patients candidate for coronary angiography	2014				IRCT2014062118178N1,	RAYYAN-INCLUSION: {"Vickie"=>"Excluded", "jsong6"=>"Excluded"} | RAYYAN-EXCLUSION-REASONS: irrelevant
rayyan-283775734	Hand posture. In writing with the nonpreferred hand. Education of the right hemisphere	1986	26	7	557â€566	Van Schaik, EC	RAYYAN-INCLUSION: {"Vickie"=>"Excluded", "jsong6"=>"Excluded"} | RAYYAN-EXCLUSION-REASONS: irrelevant
rayyan-283775735	Neglect as a deficit determined by an imbalance between multiple spatial representations	1997	116	3	493â€500	LÃ davas, E and Berti, A and Ruozzi, E and Barboni, F	RAYYAN-INCLUSION: {"Vickie"=>"Excluded", "jsong6"=>"Excluded"} | RAYYAN-EXCLUSION-REASONS: irrelevant
rayyan-283775736	Cricoid pressure: which hand?	2000	55	7	648â€653	Cook, TM and Godfrey, I and Rockett, M and Vanner, RG	RAYYAN-INCLUSION: {"Vickie"=>"Excluded", "jsong6"=>"Excluded"} | RAYYAN-EXCLUSION-REASONS: irrelevant
rayyan-283775737	Rehabilitation of allocentric negelct: A randomised clinical trial	2021				DRKS00023968,	RAYYAN-INCLUSION: {"Vickie"=>"Excluded", "jsong6"=>"Excluded"} | RAYYAN-EXCLUSION-REASONS: irrelevant
rayyan-283775738	Correlation between age, height and weightwith hand grip strength	2016					RAYYAN-INCLUSION: {"Vickie"=>"Excluded", "jsong6"=>"Excluded"} | RAYYAN-EXCLUSION-REASONS: irrelevant
rayyan-283775739	Bilateral transfer as a function of mental imagery	1980	12	3	197â€206	Kohl, RM and Roenker, DL	RAYYAN-INCLUSION: {"Vickie"=>"Excluded", "jsong6"=>"Excluded"} | RAYYAN-EXCLUSION-REASONS: irrelevant
rayyan-283775740	The embodied nature of motor imagery processes highlighted by short-term limb immobilization	2014	61	3	180â€186	Meugnot, A and Almecija, Y and Toussaint, L	RAYYAN-INCLUSION: {"Vickie"=>"Excluded", "jsong6"=>"Excluded"} | RAYYAN-EXCLUSION-REASONS: irrelevant
rayyan-283775741	Hyperbaric oxygen in multiple sclerosis: a double blind trial	1986	292	6517	367â€371	Wiles, CM and Clarke, CR and Irwin, HP and Edgar, EF and Swan, AV	RAYYAN-INCLUSION: {"Vickie"=>"Excluded", "jsong6"=>"Excluded"} | RAYYAN-EXCLUSION-REASONS: irrelevant
rayyan-283775742	Changes in resting-state functional connectivity after motor learning of chopstick manipulation with the non-dominant hand	2021				JPRN-UMIN000045453,	RAYYAN-INCLUSION: {"Vickie"=>"Excluded", "jsong6"=>"Excluded"} | RAYYAN-EXCLUSION-REASONS: irrelevant
rayyan-283775743	Quantitative EEG modifications during the Cold Water Pressor Test: hemispheric and hand differences	1994	17	3	261â€268	Ferracuti, S and Seri, S and Mattia, D and Cruccu, G	RAYYAN-INCLUSION: {"Vickie"=>"Excluded", "jsong6"=>"Excluded"} | RAYYAN-EXCLUSION-REASONS: irrelevant
rayyan-283775745	Study of the efficacy of topically applied cyclosporinsolution on psoriatic nails	2006				NTR715,	RAYYAN-INCLUSION: {"Vickie"=>"Excluded", "jsong6"=>"Excluded"} | RAYYAN-EXCLUSION-REASONS: irrelevant,wrong study design
rayyan-283775746	Erratum: idiopathic nephrotic syndrome in childre (The Lancet (2018) 392(10141) (61-74) (S0140673618305361) (10.1016/S0140-6736(18)30536-1))	2018	392	10144	282	Anonymous,	RAYYAN-INCLUSION: {"Vickie"=>"Excluded", "jsong6"=>"Excluded"} | RAYYAN-EXCLUSION-REASONS: irrelevant
rayyan-283775747	Explicit and implicit motor learning ic children with autism spectrum disorder	2012				IRCT2012091110816N1,	RAYYAN-INCLUSION: {"Vickie"=>"Excluded", "jsong6"=>"Excluded"} | RAYYAN-EXCLUSION-REASONS: irrelevant
rayyan-283775748	Vibration induced injuries in hands in longterm vibration exposed workers	2018	75		A515â€•	Gerhardsson, L and Hagberg, M	RAYYAN-INCLUSION: {"Vickie"=>"Excluded", "jsong6"=>"Excluded"} | RAYYAN-EXCLUSION-REASONS: irrelevant
rayyan-283775749	Orienting visuospatial attention generates manual reaction time asymmetries in target detection and pointing	2002	133	1	109â€116	BarthÃ©lÃ©my, S and Boulinguez, P	RAYYAN-INCLUSION: {"Vickie"=>"Excluded", "jsong6"=>"Excluded"} | RAYYAN-EXCLUSION-REASONS: irrelevant
rayyan-283775750	Movement observation-induced modulation of pain perception and motor cortex excitability	2015	126	6	1204â€1211	Volz, MS and Suarez-Contreras, V and Portilla, ALS and Illigens, B and Bermpohl, F and Fregni, F	RAYYAN-INCLUSION: {"Vickie"=>"Excluded", "jsong6"=>"Excluded"} | RAYYAN-EXCLUSION-REASONS: irrelevant
rayyan-283775751	Hemispheric processing of emotional stimuli: effect of gender, response hand, and attention	1993	6	4	209â€213	Freeman, FG and Traugott, J	RAYYAN-INCLUSION: {"Vickie"=>"Excluded", "jsong6"=>"Excluded"} | RAYYAN-EXCLUSION-REASONS: irrelevant
rayyan-283775752	Lightwand-guided endotracheal intubation performed by the nondominant hand is feasible	2007	23	10	504â€510	Kuo, YW and Yen, MK and Cheng, KI and Tang, CS and Chau, SW and Hou, MF and Wang, JJ and Lin, SF	RAYYAN-INCLUSION: {"Vickie"=>"Excluded", "jsong6"=>"Excluded"} | RAYYAN-EXCLUSION-REASONS: irrelevant
rayyan-283775753	The effect of hegu point massage with ice on pain intensity of patient post coronary artery bypass graft surgery	2016				IRCT2015111424047N2,	RAYYAN-INCLUSION: {"Vickie"=>"Excluded", "jsong6"=>"Excluded"} | RAYYAN-EXCLUSION-REASONS: irrelevant
rayyan-283775754	Effect acupressure and ice massage on pain intensity	2012				IRCT201102222515N2,	RAYYAN-INCLUSION: {"Vickie"=>"Excluded", "jsong6"=>"Excluded"} | RAYYAN-EXCLUSION-REASONS: irrelevant
rayyan-283775755	Lateralized interference of repetitive finger tapping: influence of familial handedness, cognitive load and verbal production	1983	21	6	617â€624	Kee, DW and Bathurst, K and Hellige, JB	RAYYAN-INCLUSION: {"Vickie"=>"Excluded", "jsong6"=>"Excluded"} | RAYYAN-EXCLUSION-REASONS: irrelevant
rayyan-283775756	Improving motor performance without training: the effect of combining mirror visual feedback with transcranial direct current stimulation	2015	113	7	2383â€2389	von Rein, E and Hoff, M and Kaminski, E and Sehm, B and Steele, CJ and Villringer, A and Ragert, P	RAYYAN-INCLUSION: {"Vickie"=>"Excluded", "jsong6"=>"Excluded"} | RAYYAN-EXCLUSION-REASONS: irrelevant
rayyan-283775757	Treating Intention In Aphasia: Neuroplastic Substrates	2007				NCT00567242,	RAYYAN-INCLUSION: {"Vickie"=>"Excluded", "jsong6"=>"Excluded"} | RAYYAN-EXCLUSION-REASONS: irrelevant
rayyan-283775758	Clinical Trial "Assessment of the effect of Diclofenac gel and EMLA" ointment"	2010				IRCT201008234618N1,	RAYYAN-INCLUSION: {"Vickie"=>"Excluded", "jsong6"=>"Excluded"} | RAYYAN-EXCLUSION-REASONS: irrelevant
rayyan-283775759	Comparison the Effect of between hand reflexology and acupressure on the level of anxiety & hemodynamic parameters on Patients with heart disease	2017				IRCT201703287529N13,	RAYYAN-INCLUSION: {"Vickie"=>"Excluded", "jsong6"=>"Excluded"} | RAYYAN-EXCLUSION-REASONS: irrelevant
rayyan-283775760	Sleep consolidates the effector-independent representation of a motor skill	2010	171	1	227â€234	Witt, K and Margraf, N and Bieber, C and Born, J and Deuschl, G	RAYYAN-INCLUSION: {"Vickie"=>"Excluded", "jsong6"=>"Excluded"} | RAYYAN-EXCLUSION-REASONS: irrelevant
rayyan-283775761	The Modified Otago Exercises Prevent Grip Strength Deterioration Among Older Fallers in the Malaysian Falls Assessment and Intervention Trial (MyFAIT)	2019	42	3	123â€129	Liew, LK and Tan, MP and Tan, PJ and Mat, S and Majid, LA and Hill, KD and Mazlan, M	RAYYAN-INCLUSION: {"Vickie"=>"Excluded", "jsong6"=>"Excluded"} | RAYYAN-EXCLUSION-REASONS: irrelevant
rayyan-283775762	Which Fingers Should We Perform Two-Finger Chest Compression Technique with When Performing Cardiopulmonary Resuscitation on an Infant in Cardiac Arrest?	2016	31	6	997â€1002	Kim, YS and Oh, JH and Kim, CW and Kim, SE and Lee, DH and Hong, JY	RAYYAN-INCLUSION: {"Vickie"=>"Excluded", "jsong6"=>"Excluded"} | RAYYAN-EXCLUSION-REASONS: irrelevant
rayyan-283775763	Effect of mindfulness meditation on brain-computer interface	2019	24	4	343â€353	Ramli, N and Kuok, SS and Tan, LK and Tan, YQ and Tan, LF and Goh, KJ and Kadir, KAA and Seow, PC and Goh, SY	RAYYAN-INCLUSION: {"Vickie"=>"Excluded", "jsong6"=>"Excluded"} | RAYYAN-EXCLUSION-REASONS: irrelevant
rayyan-283775764	Asymmetrical hemispheric activation and behavioral persistence: effects of unilateral muscle contractions	1998	12	4	526â€532	Schiff, BB and Guirguis, M and Kenwood, C and Herman, CP	RAYYAN-INCLUSION: {"Vickie"=>"Excluded", "jsong6"=>"Excluded"} | RAYYAN-EXCLUSION-REASONS: irrelevant,wrong study design
rayyan-283775765	Comparison of ipsilateral activation between right and left handers: a functional MR imaging study	1998	9	8	1861â€1866	Singh, LN and Higano, S and Takahashi, S and Kurihara, N and Furuta, S and Tamura, H and Shimanuki, Y and Mugikura, S and Fujii, T and Yamadori, A and Sakamoto, M and Yamada, S	RAYYAN-INCLUSION: {"Vickie"=>"Excluded", "jsong6"=>"Excluded"} | RAYYAN-EXCLUSION-REASONS: irrelevant,wrong study design
rayyan-283775766	Right hand superiority in serial recall of tactile stimuli	1982	18	4	595â€601	Riggio, L and Dellantonio, A and Barbato, M	RAYYAN-INCLUSION: {"Vickie"=>"Excluded", "jsong6"=>"Excluded"} | RAYYAN-EXCLUSION-REASONS: irrelevant
rayyan-283775767	Left hemisphere preponderance in trajectorial learning	1992	3	5	397â€400	Halsband, U	RAYYAN-INCLUSION: {"Vickie"=>"Excluded", "jsong6"=>"Excluded"} | RAYYAN-EXCLUSION-REASONS: irrelevant
rayyan-283775768	Controlled clinical trial of imidazole.2-hydroxybenzoate (ITF 182) versus sulindac in patients with rheumatoid arthritis	1986	8	3	292â€300	Fumagalli, M and Cunietti, E and Vaiani, G and Monti, M and Ferrari, F and Gandini, R	RAYYAN-INCLUSION: {"Vickie"=>"Excluded", "jsong6"=>"Excluded"} | RAYYAN-EXCLUSION-REASONS: irrelevant
rayyan-283775769	The effects of two matched memory tasks on concurrent finger tapping	1994	32	1	125â€129	Towell, N and Burton, A and Burton, E	RAYYAN-INCLUSION: {"Vickie"=>"Excluded", "jsong6"=>"Excluded"} | RAYYAN-EXCLUSION-REASONS: irrelevant
rayyan-283775770	RCT of Tactile touch for elderly people	2010				JPRN-UMIN000004186,	RAYYAN-INCLUSION: {"Vickie"=>"Excluded", "jsong6"=>"Excluded"} | RAYYAN-EXCLUSION-REASONS: irrelevant
rayyan-283775771	Effects of High and Low Frequency TENS on Sympathetic Skin Response and Skin Temperature	2012				NCT01696149,	RAYYAN-INCLUSION: {"Vickie"=>"Excluded", "jsong6"=>"Excluded"} | RAYYAN-EXCLUSION-REASONS: irrelevant
rayyan-283775772	Study on the Effect of Electroacupuncture at Both Zusanli and at Both Yanglingquan Acupoints on Cardiovascular Activity	2012				NCT01665105,	RAYYAN-INCLUSION: {"Vickie"=>"Excluded", "jsong6"=>"Excluded"} | RAYYAN-EXCLUSION-REASONS: irrelevant
rayyan-283775773	A functional magnetic imaging (fMRI) study of the effects of cannabis-based medicines (CMEs) on the neutral activity associated with noxious thermal stimulation in healthy human volunteers (CRI.FM024). Protocol V2	2004				ISRCTN58754671,	RAYYAN-INCLUSION: {"Vickie"=>"Excluded", "jsong6"=>"Excluded"} | RAYYAN-EXCLUSION-REASONS: irrelevant
rayyan-283775774	Psychophysical and electrophysiological approaches to the pain-relieving effects of heterotopic nociceptive stimuli	1984	107	4	1095â€1112	Willer, JC and Roby, A and Le, BD	RAYYAN-INCLUSION: {"Vickie"=>"Excluded", "jsong6"=>"Excluded"} | RAYYAN-EXCLUSION-REASONS: irrelevant
rayyan-283775775	Parathyroid gland autograft in the thyroidectomy: a prospective randomized controlled trial	2015				ChiCTR-IPR-15006777,	RAYYAN-INCLUSION: {"Vickie"=>"Excluded", "jsong6"=>"Excluded"} | RAYYAN-EXCLUSION-REASONS: irrelevant
rayyan-283775776	Hemispheric specialization for the visual control of action is independent of handedness	2006	95	6	3496â€3501	Gonzalez, CL and Ganel, T and Goodale, MA	RAYYAN-INCLUSION: {"Vickie"=>"Excluded", "jsong6"=>"Excluded"} | RAYYAN-EXCLUSION-REASONS: irrelevant,no right and left limb training
rayyan-283775777	A Closer Look at Motor Overflow in Dyslexic Children	1991			7	Parlow, and S, E	RAYYAN-INCLUSION: {"Vickie"=>"Excluded", "jsong6"=>"Excluded"} | RAYYAN-EXCLUSION-REASONS: irrelevant
rayyan-283775778	Functional MR imaging of the motor cortex in healthy volunteers and patients with brain tumours: qualitative and quantitative results	1999	52	1	3ï¿½ï¿½ï¿½14	Fellner, C and Schlaier, J and Fellner, F and Held, P and Blank, M and Schwerdtner, J and Brawanski, A and Kalender, WA	RAYYAN-INCLUSION: {"Vickie"=>"Excluded", "jsong6"=>"Excluded"} | RAYYAN-EXCLUSION-REASONS: irrelevant,no right and left limb training
rayyan-283775779	Unilateral hand contractions produce motivational biases in social economic decision making	2015	29	1	76â€81	HarlÃ©, KM and Sanfey, AG	RAYYAN-INCLUSION: {"Vickie"=>"Excluded", "jsong6"=>"Excluded"} | RAYYAN-EXCLUSION-REASONS: irrelevant
rayyan-283775780	Effects of acupoints TENS on heat pain threshold in normal subjects	2003	116	12	1864â€1868	Wang, N and Hui-Chan, C	RAYYAN-INCLUSION: {"Vickie"=>"Excluded", "jsong6"=>"Excluded"} | RAYYAN-EXCLUSION-REASONS: irrelevant
rayyan-283775781	Dissociating cognitive and motor interference effects on kinesthetic short-term memory	2009	73	3	380â€389	Kirsch, W and Hennighausen, E and RÃ¶sler, F	RAYYAN-INCLUSION: {"Vickie"=>"Excluded", "jsong6"=>"Excluded"} | RAYYAN-EXCLUSION-REASONS: irrelevant
rayyan-283775782	Tactile perceptual asymmetry: the influence of motor activity and imagery instructions	1989	80		517â€525	McFarland, K and Ashton, R and Heath, G	RAYYAN-INCLUSION: {"Vickie"=>"Excluded", "jsong6"=>"Excluded"} | RAYYAN-EXCLUSION-REASONS: irrelevant
rayyan-283775783	Intermanual transfer in a simple motor task	2002	38	5	805â€815	Schulze, K and LÃ¼ders, E and JÃ¤ncke, L	RAYYAN-INCLUSION: {"Vickie"=>"Excluded", "jsong6"=>"Excluded"} | RAYYAN-EXCLUSION-REASONS: irrelevant,wrong study design
rayyan-283775784	Methylphenidate alters brain connectivity after enhanced physical performance	2018	1679		26â€32	King, M and van Breda, K and Rauch, LH and Brooks, SJ and Stein, DJ and Ipser, J	RAYYAN-INCLUSION: {"Vickie"=>"Excluded", "jsong6"=>"Excluded"} | RAYYAN-EXCLUSION-REASONS: irrelevant
rayyan-283775785	Adding transcutaneous electrical nerve stimulation to visual scanning training does not enhance treatment effect on hemispatial neglect: a randomized, controlled, double-blind study	2016	23	6	377â€383	SeniÃ³w, J and Polanowska, K and LeÅ›niak, M and CzÅ‚onkowska, A	RAYYAN-INCLUSION: {"Vickie"=>"Excluded", "jsong6"=>"Excluded"} | RAYYAN-EXCLUSION-REASONS: irrelevant
rayyan-283775786	Cutaneous anesthesia of the forearm enhances sensorimotor function of the hand	2013	109	4	1091â€1096	Petoe, MA and Jaque, FA and Byblow, WD and Stinear, CM	RAYYAN-INCLUSION: {"Vickie"=>"Excluded", "jsong6"=>"Excluded"} | RAYYAN-EXCLUSION-REASONS: irrelevant
rayyan-283775787	The neural substrate of the ideomotor principle revisited: evidence for asymmetries in action-effect learning	2013	231		13â€27	Melcher, T and Winter, D and Hommel, B and Pfister, R and Dechent, P and Gruber, O	RAYYAN-INCLUSION: {"Vickie"=>"Excluded", "jsong6"=>"Excluded"} | RAYYAN-EXCLUSION-REASONS: irrelevant
rayyan-283775788	Functional magnetic resonance imaging during pantomiming tool-use gestures	2001	139	3	311â€317	Choi, SH and Na, DL and Kang, E and Lee, KM and Lee, SW and Na, DG	RAYYAN-INCLUSION: {"Vickie"=>"Excluded", "jsong6"=>"Excluded"} | RAYYAN-EXCLUSION-REASONS: irrelevant
rayyan-283775789	L-Menthol attenuates the magnitude of cold-induced vasodilation on the extremities of young females	2018	37	1	14	Kim, S and Lee, JY	RAYYAN-INCLUSION: {"Vickie"=>"Excluded", "jsong6"=>"Excluded"} | RAYYAN-EXCLUSION-REASONS: irrelevant
rayyan-283775790	Beneficial effects of an investigational wristband containing Synsepalum dulcificum (miracle fruit) seed oil on the performance of hand and finger motor skills in healthy subjects: a randomized controlled preliminary st	2018	32	2	321â€332	Gorin, S and Wakeford, C and Zhang, G and Sukamtoh, E and Matteliano, CJ and Finch, AE	RAYYAN-INCLUSION: {"Vickie"=>"Excluded", "jsong6"=>"Excluded"} | RAYYAN-EXCLUSION-REASONS: irrelevant
rayyan-283775791	Sensory dysfunction associated with repetitive strain injuries of tendinitis and focal hand dystonia: a comparative study	1996	23	4	234â€244	Byl, N and Wilson, F and Merzenich, M and Melnick, M and Scott, P and Oakes, A and McKenzie, A	RAYYAN-INCLUSION: {"Vickie"=>"Excluded", "jsong6"=>"Excluded"} | RAYYAN-EXCLUSION-REASONS: irrelevant
rayyan-283775793	Roles of the right dorsolateral prefrontal cortex during physical fatigue: a magnetoencephalographic study	2016					RAYYAN-INCLUSION: {"Vickie"=>"Excluded", "jsong6"=>"Excluded"} | RAYYAN-EXCLUSION-REASONS: irrelevant

rayyan-283775794	Effects of combining 2 weeks of passive sensory stimulation with active hand motor training in healthy adults	2013					DRKS00005118,	RAYYAN-INCLUSION: {"Vickie"=>"Excluded", "jsong6"=>"Excluded"} | RAYYAN-EXCLUSION-REASONS: wrong study design,No dom vs. non-dom comparison
rayyan-283775795	Digit ratio (2D: 4D), testosterone, cortisol, aggression, personality and hand-grip strength: evidence for prenatal effects on strength	2016	100		21â€25		Ribeiro, E and Neave, N and Morais, RN and Kilduff, L and Taylor, SR and Butovskaya, M and Fink, B and Manning, JT	RAYYAN-INCLUSION: {"Vickie"=>"Excluded", "jsong6"=>"Excluded"} | RAYYAN-EXCLUSION-REASONS: irrelevant
rayyan-283775796	The function of lidorcaine in alleviating propofol injection pain via bilateral symmetrical veins of hands or a single vein	2014					ChiCTR-IOR-15006006,	RAYYAN-INCLUSION: {"Vickie"=>"Excluded", "jsong6"=>"Excluded"} | RAYYAN-EXCLUSION-REASONS: irrelevant
rayyan-283775797	Adding transcutaneous electrical nerve stimulation to visual scanning training does not enhance treatment effect on hemispatial neglect: a randomized, controlled, double-blind study	2016	23	6	377â€383		Seniow, J and Polanowska, K and Lesniak, M and Czlonkowska, A	RAYYAN-INCLUSION: {"Vickie"=>"Excluded", "jsong6"=>"Excluded"} | RAYYAN-EXCLUSION-REASONS: irrelevant
rayyan-283775798	The influence of cardiac phase on reaction time depending on heart period length and on stimulus and response laterality	1996	24	2	169â€175		Weisz, J and Adam, G	RAYYAN-INCLUSION: {"Vickie"=>"Excluded", "jsong6"=>"Excluded"} | RAYYAN-EXCLUSION-REASONS: irrelevant
rayyan-283775799	Unilateral hand contractions produce motivational biases in social economic decision making	2015	29	1	76â€81		Harle, KM and Sanfey, AG	RAYYAN-INCLUSION: {"Vickie"=>"Excluded", "jsong6"=>"Excluded"} | RAYYAN-EXCLUSION-REASONS: irrelevant
rayyan-283775800	Comparison two methods of oxytocin use after delivery	2010					IRCT138812043413N1,	RAYYAN-INCLUSION: {"Vickie"=>"Excluded", "jsong6"=>"Excluded"} | RAYYAN-EXCLUSION-REASONS: irrelevant
rayyan-283775801	Incidence of radial artery occlusion in radial access angiography	2021					IRCT20201119049437N1,	RAYYAN-INCLUSION: {"Vickie"=>"Excluded", "jsong6"=>"Excluded"} | RAYYAN-EXCLUSION-REASONS: irrelevant
rayyan-283775802	Surgical gloves--a comparative study of the incidence and site of punctures	1992	1	11	16â€17		Bliss, S and Alexander-Williams, J	RAYYAN-INCLUSION: {"Vickie"=>"Excluded", "jsong6"=>"Excluded"} | RAYYAN-EXCLUSION-REASONS: irrelevant
rayyan-283775803	Psychophysical responses to experimentally induced heat and cold pain before, during, and after transcutaneous electrical nerve stimulation	2003	6	4	229â€236		Tulgar, M and Tulgar, O and Herken, H	RAYYAN-INCLUSION: {"Vickie"=>"Excluded", "jsong6"=>"Excluded"} | RAYYAN-EXCLUSION-REASONS: irrelevant
rayyan-283775804	How does the side of approach impact the force delivered during external chest compression?	2021	48		67â€72		Tsou, JY and Kao, CL and Hong, MY and Chang, CJ and Su, FC and Chi, CH	RAYYAN-INCLUSION: {"Vickie"=>"Excluded", "jsong6"=>"Excluded"} | RAYYAN-EXCLUSION-REASONS: irrelevant
rayyan-283775805	Cross-modal recognition of shape from hand to eyes and handedness in human newborns	2004	42	10	1365â€1369		Streri, A and Gentaz, E	RAYYAN-INCLUSION: {"Vickie"=>"Excluded", "jsong6"=>"Excluded"} | RAYYAN-EXCLUSION-REASONS: irrelevant
rayyan-283775806	Motor learning transfer from one's own virtual body (avatar) to the physical body using immersive virtual reality technology	2018					JPRN-UMIN000034357,	RAYYAN-INCLUSION: {"Vickie"=>"Excluded", "jsong6"=>"Excluded"} | RAYYAN-EXCLUSION-REASONS: irrelevant
rayyan-283775807	Right-left discrimination: effects of handedness and educational background	2002	43	3	213â€219		Ofte, SH	RAYYAN-INCLUSION: {"Vickie"=>"Excluded", "jsong6"=>"Excluded"} | RAYYAN-EXCLUSION-REASONS: irrelevant
rayyan-283775808	Methylphenidate alters brain connectivity after enhanced physical performance	2018	1679				King, M and van Breda, K and Rauch, LH and Brooks, SJ and Stein, DJ and Ipser, J	RAYYAN-INCLUSION: {"Vickie"=>"Excluded", "jsong6"=>"Excluded"} | RAYYAN-EXCLUSION-REASONS: irrelevant
rayyan-283775809	The effects of dry needling on the autonomic nervous system function in healthy subjects: a randomized single blind clinical trial	2016					ACTRN12616000881437,	RAYYAN-INCLUSION: {"Vickie"=>"Excluded", "jsong6"=>"Excluded"} | RAYYAN-EXCLUSION-REASONS: irrelevant
rayyan-283775810	Cardiopulmonary Function and Quality of Life in Pulmonary Tuberculosis	2021					NCT04844502,	RAYYAN-INCLUSION: {"Vickie"=>"Excluded", "jsong6"=>"Excluded"} | RAYYAN-EXCLUSION-REASONS: irrelevant
rayyan-283775811	Does the da vinci robot system eliminate hand dominance in performing surgical skills? a randomized evaluation of multispecialty surgical trainees' hand dominance in the open and robotic surgical settings	2013	189	4	e640		Bergman, A and Patel, T and Shapiro, E and RoyChoudhury, A and Badani, K	RAYYAN-INCLUSION: {"Vickie"=>"Excluded", "jsong6"=>"Excluded"} | RAYYAN-EXCLUSION-REASONS: irrelevant
rayyan-283775812	Bilateral finger temperature and the Law of Initial Values	1987	24	6	666â€669		Jamieson, J	RAYYAN-INCLUSION: {"Vickie"=>"Excluded", "jsong6"=>"Excluded"} | RAYYAN-EXCLUSION-REASONS: irrelevant
rayyan-283775813	Walking trajectory and hand movements in unilateral left neglect: a vestibular hypothesis	1994	32	12	1495â€1502		Robertson, IH and TegnÃ©r, R and Goodrich, SJ and Wilson, C	RAYYAN-INCLUSION: {"Vickie"=>"Excluded", "jsong6"=>"Excluded"} | RAYYAN-EXCLUSION-REASONS: irrelevant
rayyan-283775814	Psychopathy and electrodermal responses to nonsignal stimulation	1978	6	4	237â€246		Hare, RD	RAYYAN-INCLUSION: {"Vickie"=>"Excluded", "jsong6"=>"Excluded"} | RAYYAN-EXCLUSION-REASONS: irrelevant
rayyan-283775815	Functional asymmetries in the quality of handwriting movements: a kinematic analysis	1999	13	2	291â€297		Phillips, JG and Gallucci, RM and Bradshaw, JL	RAYYAN-INCLUSION: {"Vickie"=>"Excluded", "jsong6"=>"Excluded"} | RAYYAN-EXCLUSION-REASONS: irrelevant
rayyan-283775816	Using model hands for learning orthotic fabrication	2014	68	1	86â€94		Hagemann, E and Williams, CK and McKee, P and Stefanovich, A and Carnahan, H	RAYYAN-INCLUSION: {"Vickie"=>"Excluded", "jsong6"=>"Excluded"} | RAYYAN-EXCLUSION-REASONS: irrelevant
rayyan-283775817	How the motor system handles nouns: a behavioral study	2013	77	1	64â€73		Marino, BF and Gough, PM and Gallese, V and Riggio, L and Buccino, G	RAYYAN-INCLUSION: {"Vickie"=>"Excluded", "jsong6"=>"Excluded"} | RAYYAN-EXCLUSION-REASONS: irrelevant
rayyan-283775818	Recognizing the motion of a graspable object is guided by handedness	1997	8	12	2761â€2765		de'Sperati, C and Stucchi, N	RAYYAN-INCLUSION: {"Vickie"=>"Excluded", "jsong6"=>"Excluded"} | RAYYAN-EXCLUSION-REASONS: irrelevant
rayyan-283775819	Effects of exercise on fatigue, aerobic fitness, and disease activity measures in persons with rheumatoid arthritis	1997	20	3	195â€204		Neuberger, GB and Press, AN and Lindsley, HB and Hinton, R and Cagle, PE and Carlson, K and Scott, S and Dahl, J and Kramer, B	RAYYAN-INCLUSION: {"Vickie"=>"Excluded", "jsong6"=>"Excluded"} | RAYYAN-EXCLUSION-REASONS: irrelevant
rayyan-283775820	Effect on finger tremor of withdrawal of long-term treatment with propranolol or atenolol	1984	18	3	317â€324		Wharrad, HJ and Birmingham, AT and Wilson, CG and Williams, EJ and Roland, JM	RAYYAN-INCLUSION: {"Vickie"=>"Excluded", "jsong6"=>"Excluded"} | RAYYAN-EXCLUSION-REASONS: irrelevant
rayyan-283775821	Dynamic reaching in infants during binocular and monocular viewing	2013	229	1	1â€12		Ekberg, TL and Rosander, K and von Hofsten, C and Olsson, U and Soska, KC and Adolph, KE	RAYYAN-INCLUSION: {"Vickie"=>"Excluded", "jsong6"=>"Excluded"} | RAYYAN-EXCLUSION-REASONS: irrelevant
rayyan-283775822	Sensorimotor robotic measures of tDCS and HD-tDCS enhanced motor learning in children	2018			e120		Cole, L and Giuffre, A and Ciechanski, P and Metzler, M and Carlson, H and Zewdie, E and Kuo, H-C and Webber, A and Harris, A and Kirton, A	RAYYAN-INCLUSION: {"Vickie"=>"Excluded", "jsong6"=>"Excluded"} | RAYYAN-EXCLUSION-REASONS: irrelevant
rayyan-283775823	Motor planning in bimanual object manipulation: two plans for two hands?	2010	14	2	240â€254		Janssen, L and CrajÃ©, C and Weigelt, M and Steenbergen, B	RAYYAN-INCLUSION: {"Vickie"=>"Excluded", "jsong6"=>"Excluded"} | RAYYAN-EXCLUSION-REASONS: irrelevant
rayyan-283775824	The influence of target perturbation on manual aiming asymmetries in right-handers	1995	31	4	685â€697		Elliott, D and Lyons, J and Chua, R and Goodman, D and Carson, RG	RAYYAN-INCLUSION: {"Vickie"=>"Excluded", "jsong6"=>"Excluded"} | RAYYAN-EXCLUSION-REASONS: irrelevant
rayyan-283775825	Joint protection and home hand exercises improve hand function in patients with hand osteoarthritis: a randomized controlled trial	2002	47	1	44â€49		Stamm, TA and Machold, KP and Smolen, JS and Fischer, S and Redlich, K and Graninger, W and Ebner, W and Erlacher, L	RAYYAN-INCLUSION: {"Vickie"=>"Excluded", "jsong6"=>"Excluded"} | RAYYAN-EXCLUSION-REASONS: No dom vs. non-dom comparison
rayyan-283775826	Effects of external loads on postural sway during quiet stance in adults aged 20-80 years	2018	66		64â€69		Hill, MW and Duncan, MJ and Oxford, SW and Kay, AD and Price, MJ	RAYYAN-INCLUSION: {"Vickie"=>"Excluded", "jsong6"=>"Excluded"} | RAYYAN-EXCLUSION-REASONS: irrelevant
rayyan-283775827	Bimanual interference associated with the selection of target locations	2003	29	1	64â€77		Diedrichsen, J and Ivry, RB and Hazeltine, E and Kennerley, S and Cohen, A	RAYYAN-INCLUSION: {"Vickie"=>"Excluded", "jsong6"=>"Excluded"} | RAYYAN-EXCLUSION-REASONS: irrelevant
rayyan-283775828	Effect of keyboard keyswitch design on hand pain	1999	41	2	111â€119		Rempel, D and Tittiranonda, P and Burastero, S and Hudes, M and So, Y	RAYYAN-INCLUSION: {"Vickie"=>"Excluded", "jsong6"=>"Excluded"} | RAYYAN-EXCLUSION-REASONS: irrelevant
rayyan-283775829	Selected parameters of response organization in mildly mentally retarded children	1987	64	1	95â€100		Porretta, DL	RAYYAN-INCLUSION: {"Vickie"=>"Excluded", "jsong6"=>"Excluded"} | RAYYAN-EXCLUSION-REASONS: irrelevant
rayyan-283775830	Does transcutaneous nerve stimulation have effect on sympathetic skin response?	2017					Okuyucu, EE and Turhanoglu, AD and Guntel, M and Yilmazer, S and Savas, N and Mansuroglu, A	RAYYAN-INCLUSION: {"Vickie"=>"Excluded", "jsong6"=>"Excluded"} | RAYYAN-EXCLUSION-REASONS: irrelevant
rayyan-283775831	Right-left asymmetry in tonic pain perception and its modification by simultaneous contralateral noxious stimulation	1993	31	7	687â€694		Chandramouli, R and Kanchan, BR and Ambadevi, B	RAYYAN-INCLUSION: {"Vickie"=>"Excluded", "jsong6"=>"Excluded"} | RAYYAN-EXCLUSION-REASONS: irrelevant
rayyan-283775832	Early visual experience affects memorization and spatial representation of proprioceptive targets	1996	7	6	1219â€1223		Rossetti, Y and Gaunet, F and Thinus-Blanc, C	RAYYAN-INCLUSION: {"Vickie"=>"Excluded", "jsong6"=>"Excluded"} | RAYYAN-EXCLUSION-REASONS: irrelevant
rayyan-283775833	Clinical and laboratory studies of levamisole in patients with rheumatoid arthritis	1978	47	187	385â€400		El-Ghobarey, AF and Mavrikakis, ME and Macleod, M and Reynolds, PM and Capell, HA and Spencer, DG and Balint, G and Mathieu, JP and McAllister, T and Cooney, A and Dick, WC	RAYYAN-INCLUSION: {"Vickie"=>"Excluded", "jsong6"=>"Excluded"} | RAYYAN-EXCLUSION-REASONS: irrelevant
rayyan-283775834	Video Game Exercise Effectiveness of a Domiciliary Pulmonary Rehabilitation Program in Cystic Fibrosis Patients	2015					NCT02552043,	RAYYAN-INCLUSION: {"Vickie"=>"Excluded", "jsong6"=>"Excluded"} | RAYYAN-EXCLUSION-REASONS: irrelevant
rayyan-283775835	Influence of 10 Hz rTMS and noradrenergic modulation on motor learning in healthy humans	2013					DRKS00004653,	RAYYAN-INCLUSION: {"Vickie"=>"Excluded", "jsong6"=>"Excluded"} | RAYYAN-EXCLUSION-REASONS: irrelevant
rayyan-283775836	A randomized study of dynamic vs static external fixation of distal radial fractures	2018	12	2	825â€827		Mehmood, A and Ishaq, M and Muhammad, E	RAYYAN-INCLUSION: {"Vickie"=>"Excluded", "jsong6"=>"Excluded"} | RAYYAN-EXCLUSION-REASONS: irrelevant
rayyan-283775837	A randomized controlled trial of home exercise on the rheumatoid hand	1993	20	5	785â€789		Hoenig, H and Groff, G and Pratt, K and Goldberg, E and Franck, W	RAYYAN-INCLUSION: {"Vickie"=>"Excluded", "jsong6"=>"Excluded"} | RAYYAN-EXCLUSION-REASONS: No dom vs. non-dom comparison
rayyan-283775838	Crossing the midline by four to eight year old children	1988	26	2	221â€235		Bradshaw, JL and Spataro, JA and Harris, M and Nettleton, NC and Bradshaw, J	RAYYAN-INCLUSION: {"Vickie"=>"Excluded", "jsong6"=>"Excluded"} | RAYYAN-EXCLUSION-REASONS: irrelevant
rayyan-283775839	Efficiency of applying P6 acupressure on nausea , vomiting and comfort	2020					IRCT20160110025929N30,	RAYYAN-INCLUSION: {"Vickie"=>"Excluded", "jsong6"=>"Excluded"} | RAYYAN-EXCLUSION-REASONS: irrelevant
rayyan-283775840	Differences in face touching by Japanese and British people	1984	22	4	531â€534		Hatta, T and Dimond, SJ	RAYYAN-INCLUSION: {"Vickie"=>"Excluded", "jsong6"=>"Excluded"} | RAYYAN-EXCLUSION-REASONS: irrelevant
rayyan-283775841	Effect of using breastfeed observation aid on latching and breastfeeding	2014					TCTR20140829001,	RAYYAN-INCLUSION: {"Vickie"=>"Excluded", "jsong6"=>"Excluded"} | RAYYAN-EXCLUSION-REASONS: irrelevant
rayyan-283775842	Right hemisphere sensitivity to feedback	1990	28	12	1261â€1271		Derryberry, D	RAYYAN-INCLUSION: {"Vickie"=>"Excluded", "jsong6"=>"Excluded"} | RAYYAN-EXCLUSION-REASONS: irrelevant
rayyan-283775843	Somatosensory electrical stimulation improves skill acquisition, consolidation, and transfer by increasing sensorimotor activity and connectivity	2018	120	1	281â€290		Veldman, MP and Maurits, NM and Zijdewind, I and Maffiuletti, NA and van Middelkoop, S and Mizelle, JC and HortobÃ¡gyi, T	RAYYAN-INCLUSION: {"Vickie"=>"Excluded", "jsong6"=>"Excluded"} | RAYYAN-EXCLUSION-REASONS: irrelevant
rayyan-283775844	Intermanual difference in the Japanese Trail Making Test and its mirror version: intra-subject comparison of the task-completion time, cognitive time, and motor time	2003	25	24	1339â€1343		Toyokura, M and Ishida, A and Watanabe, F and Okada, N and Yamazaki, M	RAYYAN-INCLUSION: {"Vickie"=>"Excluded", "jsong6"=>"Excluded"} | RAYYAN-EXCLUSION-REASONS: irrelevant
rayyan-283775845	Effects of Fat Grip Training on Muscular Strength and Driving Performance in Division I Male Golfers	2018	32	1	205â€210		Cummings, PM and Waldman, HS and Krings, BM and Smith, JW and McAllister, MJ	RAYYAN-INCLUSION: {"Vickie"=>"Excluded", "jsong6"=>"Excluded"} | RAYYAN-EXCLUSION-REASONS: irrelevant
rayyan-283775846	Astigmatic neutrality in biaxial microincision cataract surgery	2009	35	9	1555â€1562		Kaufmann, C and Krishnan, A and Landers, J and Esterman, A and Thiel, MA and Goggin, M	RAYYAN-INCLUSION: {"Vickie"=>"Excluded", "jsong6"=>"Excluded"} | RAYYAN-EXCLUSION-REASONS: irrelevant
rayyan-283775847	Accelographic and mechanical post-tetanic count and train-of-four ratio assessed at the great toe	1998	15	6	649â€655		Saitoh, Y and Fujii, Y and Ueki, M and Makita, K and Amaha, K	RAYYAN-INCLUSION: {"Vickie"=>"Excluded", "jsong6"=>"Excluded"} | RAYYAN-EXCLUSION-REASONS: irrelevant
rayyan-283775848	The Transferability of Generic Minimally Invasive Surgical Skills: Is There Crossover of Core Skills Between Laparoscopy and Arthroscopy?	2016	73	2	329â€338		Akhtar, K and Sugand, K and Wijendra, A and Sarvesvaran, M and Sperrin, M and Standfield, N and Cobb, J and Gupte, C	RAYYAN-INCLUSION: {"Vickie"=>"Excluded", "jsong6"=>"Excluded"} | RAYYAN-EXCLUSION-REASONS: irrelevant
rayyan-283775849	Assessment of 2D: 4D ratio for the early diagnosis of caries and malocclusion in 7-15-year-old children	2021	20	1	154â€158		Issrani, R and Aljohani, F and Prabhu, N and Alam, MK	RAYYAN-INCLUSION: {"Vickie"=>"Excluded", "jsong6"=>"Excluded"} | RAYYAN-EXCLUSION-REASONS: irrelevant
rayyan-283775850	The effects of Hegu point ice massage on post-sternotomy pain in patients undergoing coronary artery bypass grafting: A singleâ€"blind, randomized, clinical trial	2020	7	2	73â€78		Taherian, T and Shorofi, SA and Zeydi, AE and Charati, JY and Pouresmail, Z and Jafari, H	RAYYAN-INCLUSION: {"Vickie"=>"Excluded", "jsong6"=>"Excluded"} | RAYYAN-EXCLUSION-REASONS: irrelevant
rayyan-283775851	Radial Artery Vasodilation Heat Study	2018					NCT03620383,	RAYYAN-INCLUSION: {"Vickie"=>"Excluded", "jsong6"=>"Excluded"} | RAYYAN-EXCLUSION-REASONS: irrelevant
rayyan-283775852	A comparison of two techniques of uterine closure in caesarean section	2016	29	10	1573â€1576		KÃ¶stÃ¼, B and Ercan, Ã– and Ã–zer, A and Bakacak, M and Ã–zdemir, Ã– and AvcÄ±, F	RAYYAN-INCLUSION: {"Vickie"=>"Excluded", "jsong6"=>"Excluded"} | RAYYAN-EXCLUSION-REASONS: irrelevant
rayyan-283775853	Paradoxically greater interhemispheric transfer deficits in partial than complete callosal agenesis	1998	36	10	1015â€1024		Aglioti, S and Beltramello, A and Tassinari, G and Berlucchi, G	RAYYAN-INCLUSION: {"Vickie"=>"Excluded", "jsong6"=>"Excluded"} | RAYYAN-EXCLUSION-REASONS: irrelevant
rayyan-283775854	Intravenous Lidocaine Alleviates the Pain of Propofol Injection by Local Anesthetic and Central Analgesic Effects	2018	19	3	598â€607		Xing, J and Liang, L and Zhou, S and Luo, C and Cai, J and Hei, Z	RAYYAN-INCLUSION: {"Vickie"=>"Excluded", "jsong6"=>"Excluded"} | RAYYAN-EXCLUSION-REASONS: irrelevant
rayyan-283775855	The effectiveness of Kinesio taping on playing-related pain, function and muscle strength in violin players: a randomized controlled clinical trial	2021	52		121â€131		Topdemir, E and Birinci, T and TaÅŸkÄ±ran, H and Mutlu, EK	RAYYAN-INCLUSION: {"Vickie"=>"Excluded", "jsong6"=>"Excluded"} | RAYYAN-EXCLUSION-REASONS: irrelevant
rayyan-283775856	The effect of preoperative oral carbohydrate loading on postoperative recovery after colorectal resections-a randomized controlled trial	2011	13		70â€•		Perakath, B and Bandyopadhyay, S and Jesudason, MR and Samuel, P and Nayak, S	RAYYAN-INCLUSION: {"Vickie"=>"Excluded", "jsong6"=>"Excluded"} | RAYYAN-EXCLUSION-REASONS: irrelevant
rayyan-283775857	Impairments of movement kinematics in patients with Huntington's disease: a comparison with and without a concurrent task	1997	12	3	386â€396		Georgiou, N and Phillips, JG and Bradshaw, JL and Cunnington, R and Chiu, E	RAYYAN-INCLUSION: {"Vickie"=>"Excluded", "jsong6"=>"Excluded"} | RAYYAN-EXCLUSION-REASONS: irrelevant
rayyan-283775858	The Effects of Mid-thoracic Manipulation on Heart Rate Variability	2015					NCT02379871,	RAYYAN-INCLUSION: {"Vickie"=>"Excluded", "jsong6"=>"Excluded"} | RAYYAN-EXCLUSION-REASONS: irrelevant
rayyan-283775859	Lateralized ultradian rhythms of the right and left brain: temporal variations of tactile discrimination tested in German subjects	1999	31	2	221â€231		Meier-Koll, A	RAYYAN-INCLUSION: {"Vickie"=>"Excluded", "jsong6"=>"Excluded"} | RAYYAN-EXCLUSION-REASONS: irrelevant
rayyan-283775860	Effect Of integration of cold And vibration On anxiety And pain during intravenous cannulation among children	2018					CTRI/2018/10/015950,	RAYYAN-INCLUSION: {"Vickie"=>"Excluded", "jsong6"=>"Excluded"} | RAYYAN-EXCLUSION-REASONS: irrelevant
rayyan-283775861	Normal and radiculopathic cutaneous pain tolerance levels evaluated by heat-beam dolorimetry	1990	72	6	883â€888		Lipman, JJ and Blumenkopf, B and Lawrence, PL	RAYYAN-INCLUSION: {"Vickie"=>"Excluded", "jsong6"=>"Excluded"} | RAYYAN-EXCLUSION-REASONS: irrelevant
rayyan-283775862	Somatic sensation of hand-object interactive movement is associated with activity in the left inferior parietal cortex	2006	26	14	3783â€3790		Naito, E and Ehrsson, HH	RAYYAN-INCLUSION: {"Vickie"=>"Excluded", "jsong6"=>"Excluded"} | RAYYAN-EXCLUSION-REASONS: irrelevant
rayyan-283775863	Strength training and shoulder proprioception	2015	50	3	277â€280		Salles, JI and Velasques, B and Cossich, V and Nicoliche, E and Ribeiro, P and Amaral, MV and Motta, G	RAYYAN-INCLUSION: {"Vickie"=>"Excluded", "jsong6"=>"Excluded"} | RAYYAN-EXCLUSION-REASONS: irrelevant
rayyan-283775864	Effects of Transcranial Direct Current Stimulation and High-Definition Transcranial Direct Current Stimulation Enhanced Motor Learning on Robotic Transcranial Magnetic Stimulation Motor Maps in Children	2021	15				Giuffre, A and Zewdie, E and Wrightson, JG and Cole, L and Carlson, HL and Kuo, H-C and Babwani, A and Kirton, A	RAYYAN-INCLUSION: {"Vickie"=>"Excluded", "jsong6"=>"Excluded"} | RAYYAN-EXCLUSION-REASONS: irrelevant
rayyan-283775865	Recent History of Effector Use Modulates Practice-Dependent Changes in Corticospinal Excitability but Not Motor Learning	2016	9	4	584â€593		Hussain, SJ and Darling, WG and Cole, KJ	RAYYAN-INCLUSION: {"Vickie"=>"Excluded", "jsong6"=>"Excluded"} | RAYYAN-EXCLUSION-REASONS: irrelevant
rayyan-283775866	Quantitative ultrasound at the hand phalanges in adolescent boys in relation to their pubertal development and physical efficiency	2013	64	5	353â€357		ChwaÅ‚czyÅ„ska, A and Pluskiewicz, W and Syrycka, J and Bolanowski, M	RAYYAN-INCLUSION: {"Vickie"=>"Excluded", "jsong6"=>"Excluded"} | RAYYAN-EXCLUSION-REASONS: irrelevant
rayyan-283775867	Investigating the Causal Role of preSMA in Levodopa-induced Dyskinesia in Parkinson's Disease	2017					NCT03354455,	RAYYAN-INCLUSION: {"Vickie"=>"Excluded", "jsong6"=>"Excluded"} | RAYYAN-EXCLUSION-REASONS: irrelevant,wrong drug
rayyan-283775868	Metaphor explanation attenuates the right-hand preference for depictive co-speech gestures that imitate actions	2007	101	3	185â€197		Kita, S and de Condappa, O and Mohr, C	RAYYAN-INCLUSION: {"Vickie"=>"Excluded", "jsong6"=>"Excluded"} | RAYYAN-EXCLUSION-REASONS: irrelevant
rayyan-283775869	Wrist postures while keyboarding: effects of a negative slope keyboard system and full motion forearm supports	1995	38	3	508â€517		Hedge, A and Powers, JR	RAYYAN-INCLUSION: {"Vickie"=>"Excluded", "jsong6"=>"Excluded"} | RAYYAN-EXCLUSION-REASONS: irrelevant
rayyan-283775870	Efficacy of a Nutritional Supplement on Skin Health in Generally Healthy Adults	2018					NCT03771807,	RAYYAN-INCLUSION: {"Vickie"=>"Excluded", "jsong6"=>"Excluded"} | RAYYAN-EXCLUSION-REASONS: irrelevant
rayyan-283775871	Training and reaching in premature infants	2011					RBR-2dgbbz,	RAYYAN-INCLUSION: {"Vickie"=>"Excluded", "jsong6"=>"Excluded"} | RAYYAN-EXCLUSION-REASONS: irrelevant
rayyan-283775872	Use of Muscle Strength Training at Heel Height	2020					RBR-4wjnm9,	RAYYAN-INCLUSION: {"Vickie"=>"Excluded", "jsong6"=>"Excluded"} | RAYYAN-EXCLUSION-REASONS: irrelevant
rayyan-283775873	Individual variation in hemispheric asymmetry: multitask study of effects related to handedness and sex	1994	123	3	235â€256		Hellige, JB and Bloch, MI and Cowin, EL and Eng, TL and Eviatar, Z and Sergent, V	RAYYAN-INCLUSION: {"Vickie"=>"Excluded", "jsong6"=>"Excluded"} | RAYYAN-EXCLUSION-REASONS: irrelevant
rayyan-283775874	Comparative study of the effectiveness of 2940-nm, 1340-nm laser and intense pulsed light use on global rejuvenation of hands	2015	72	5	AB267		Cignachi, S and Campos, V and Maluf, L and Grohs, L and Wancizinski, M and Costa, M	RAYYAN-INCLUSION: {"Vickie"=>"Excluded", "jsong6"=>"Excluded"} | RAYYAN-EXCLUSION-REASONS: irrelevant
rayyan-283775875	The effectiveness of galvanic current therapy and a conservative hand exercise program in a rheumatoid hand: a randomized controlled trial	2014	32	4	S22â€•		Dulgeroglu, D and Bal, A and Karaahmet, O and Umay, E and Noyan, S and Cakci, A	RAYYAN-INCLUSION: {"Vickie"=>"Excluded", "jsong6"=>"Excluded"} | RAYYAN-EXCLUSION-REASONS: irrelevant,wrong study design
rayyan-283775876	Hand and finger skin temperatures in convective and contact cold exposure	1996	72	4	372â€379		Chen, F and Liu, ZY and HolmÃ©r, I	RAYYAN-INCLUSION: {"Vickie"=>"Excluded", "jsong6"=>"Excluded"} | RAYYAN-EXCLUSION-REASONS: irrelevant
rayyan-283775877	Impaired motor skill acquisition using mirror visual feedback improved by transcranial direct current stimulation (tDCS) in patients with ParkinsonÃ¢â‚¬â„¢s disease	2019	13				Horiba, M and Ueki, Y and Nojima, I and Shimizu, Y and Sahashi, K and Itamoto, S and Suzuki, A and Yamada, G and Matsukawa, N and Wada, I	RAYYAN-INCLUSION: {"Vickie"=>"Excluded", "jsong6"=>"Excluded"} | RAYYAN-EXCLUSION-REASONS: irrelevant
rayyan-283775878	Role of nitric oxide in the visible light-induced rapid increase of human skin microcirculation at the local and systemic levels: II. healthy volunteers	2008	26	5	443â€449		Samoilova, KA and Zhevago, NA and Petrishchev, NN and Zimin, AA	RAYYAN-INCLUSION: {"Vickie"=>"Excluded", "jsong6"=>"Excluded"} | RAYYAN-EXCLUSION-REASONS: irrelevant
rayyan-283775879	The effectiveness of extracorporeal shockwave therapy on hypertrophic scar appearance and hand mobility in a severe burn patient	2020	4	2	72â€77		Chan, SYJ	RAYYAN-INCLUSION: {"Vickie"=>"Excluded", "jsong6"=>"Excluded"} | RAYYAN-EXCLUSION-REASONS: irrelevant
rayyan-283775880	Effects of thrust manipulation: cervical spine versus thoracic spine in Cervicogenic headache	2020					CTRI/2020/06/026092,	RAYYAN-INCLUSION: {"Vickie"=>"Excluded", "jsong6"=>"Excluded"} | RAYYAN-EXCLUSION-REASONS: irrelevant
rayyan-283775881	Refined task and proficiency criteria is required for optimal virtual reality minimally invasive surgery training	2010	158	2	250â€251		Thompson, J and Broderick, T and Doarn, C and Roesch, M and Henry, B and Stephan, Z and Davis, J and Weber, J and Boyden, M	RAYYAN-INCLUSION: {"Vickie"=>"Excluded", "jsong6"=>"Excluded"} | RAYYAN-EXCLUSION-REASONS: irrelevant
rayyan-283775882	Localized muscle fatigue in laparoendoscopic single-site surgery (LESS) and conventional laparoscopy	2016	30		S15		Sanchez Margallo, FM and Gianikellis, K and Skiadopoulos, A and Hermoso De Mendoza, J and Sanchez-Margallo, JA	RAYYAN-INCLUSION: {"Vickie"=>"Excluded", "jsong6"=>"Excluded"} | RAYYAN-EXCLUSION-REASONS: irrelevant
rayyan-283775883	Cortical Activation by Yamamoto New Scalp Acupuncture (YNSA) in the treatment of stroke patients a sham-controlled study aided by Functional Magnetic Resonance Imaging (fMRI)	2009	52	1	21â€29		Schockert, T and Schnitker, R and Boroojerdi, B and Vietzke, K and Smith, IQ and Yamamoto, T and Kastrau, F	RAYYAN-INCLUSION: {"Vickie"=>"Excluded", "jsong6"=>"Excluded"} | RAYYAN-EXCLUSION-REASONS: irrelevant
rayyan-283775884	The effect of Oketani massage on breastfeeding	2020					IRCT20201108049304N1,	RAYYAN-INCLUSION: {"Vickie"=>"Excluded", "jsong6"=>"Excluded"} | RAYYAN-EXCLUSION-REASONS: irrelevant
rayyan-283775885	Study comparing two ultrasound techniques for Radial artery cannulation in adult patients undergoing Cardiac Surgery	2021					CTRI/2021/07/034730,	RAYYAN-INCLUSION: {"Vickie"=>"Excluded", "jsong6"=>"Excluded"} | RAYYAN-EXCLUSION-REASONS: irrelevant
rayyan-283775886	The effect of reflexology massage on headache caused by intravenous nitroglycerin	2016					IRCT2015042922010N1,	RAYYAN-INCLUSION: {"Vickie"=>"Excluded", "jsong6"=>"Excluded"} | RAYYAN-EXCLUSION-REASONS: irrelevant
rayyan-283775887	The Effect of Straight-Line Long-Toss Versus Ultra-Long-Toss Throwing on Passive Glenohumeral Range of Motion Recovery After Pitching	2021	13	3	237â€244		Luo, TD and Sciascia, AD and Stone, AV and Gwam, CU and Grimes, CA and Strahm, JG and Mannava, S and Naugher, GL and Waterman, BR and Freehill, MT	RAYYAN-INCLUSION: {"Vickie"=>"Excluded", "jsong6"=>"Excluded"} | RAYYAN-EXCLUSION-REASONS: irrelevant
rayyan-283775888	Comparing effects of 4 months of two self-administered exercise training programs on physical performance in patients with chronic kidney disease: RENEXC - A randomized controlled trial	2018	13	12	e0207349		Hellberg, M and HÃ¶glund, P and Svensson, P and Clyne, N	RAYYAN-INCLUSION: {"Vickie"=>"Excluded", "jsong6"=>"Excluded"} | RAYYAN-EXCLUSION-REASONS: irrelevant
rayyan-283775889	Effect of age, handedness and use of compression wear on knee joint proprioceptive target matching asymmetries	2017					JPRN-UMIN000030318,	RAYYAN-INCLUSION: {"Vickie"=>"Excluded", "jsong6"=>"Excluded"} | RAYYAN-EXCLUSION-REASONS: irrelevant
rayyan-283775890	Effect of strength training in older depressed	2014					RBR-38nkj6,	RAYYAN-INCLUSION: {"Vickie"=>"Excluded", "jsong6"=>"Excluded"} | RAYYAN-EXCLUSION-REASONS: irrelevant
rayyan-283775891	The effects of chiropractic spinal manipulation on central processing of tonic pain - a pilot study using standardized low-resolution brain electromagnetic tomography (sLORETA)	2019	9	1	6925â€•		Navid, MS and Lelic, D and Niazi, IK and Holt, K and Mark, EB and Drewes, AM and Haavik, H	RAYYAN-INCLUSION: {"Vickie"=>"Excluded", "jsong6"=>"Excluded"} | RAYYAN-EXCLUSION-REASONS: irrelevant
rayyan-283775892	Sole and palm reflexology relevance in severity of fatigue in patients undergoing hemodialysis	2018	16		S76754â€•		Iranshahi, AM and Mohammadpourhodki, R and Siyasari, A and Abadi, HB and Sheikh, A	RAYYAN-INCLUSION: {"Vickie"=>"Excluded", "jsong6"=>"Excluded"} | RAYYAN-EXCLUSION-REASONS: irrelevant
rayyan-283775893	Chopstick operation training with the left non-dominant hand	2021	12	1	385â€395		Sawamura, D and Sakuraba, S and Yoshida, K and Hasegawa, N and Suzuki, Y and Yoshida, S and Honke, T and Sakai, S	RAYYAN-INCLUSION: {"Vickie"=>"Excluded", "jsong6"=>"Excluded"} | RAYYAN-EXCLUSION-REASONS: irrelevant,no right and left limb training
rayyan-283775894	Cortical modulation of nociception by galvanic vestibular stimulation: a potential clinical tool?	2020	13	1	60â€68		Hagiwara, K and Perchet, C and Frot, M and Bastuji, H and Garcia-Larrea, L	RAYYAN-INCLUSION: {"Vickie"=>"Excluded", "jsong6"=>"Excluded"} | RAYYAN-EXCLUSION-REASONS: irrelevant
rayyan-283775895	Pain and non-pain processing during hypnosis: a thulium-YAG event-related fMRI study	2009	47	3	1047â€1054		Vanhaudenhuvse. A and Bolv. M and Balteau. E and Schnakers. C and Moonen. G and Luxen. A and Lamv. M and Degueldre. C and Brichant. JF and Maquet. P and Laureys. S and Faymonville.	RAYYAN-INCLUSION: {"Vickie"=>"Excluded", "jsong6"=>"Excluded"} | RAYYAN-EXCLUSION-REASONS: irrelevant
rayyan-283775896	Effects of sensory behavioral tasks on pain threshold and cortical excitability	2013	8	1	e52968		Volz, MS and Suarez-Contreras, V and Mendonca, ME and Pinheiro, FS and Merabet, LB and Fregni, F	RAYYAN-INCLUSION: {"Vickie"=>"Excluded", "jsong6"=>"Excluded"} | RAYYAN-EXCLUSION-REASONS: irrelevant
rayyan-283775897	Sensorimotor representation and functional motor changes following short-term arm immobilization	2018	132	6	595â€603		Debarnot, U and Huber, C and Guillot, A and Schwartz, S	RAYYAN-INCLUSION: {"Vickie"=>"Excluded", "jsong6"=>"Excluded"} | RAYYAN-EXCLUSION-REASONS: irrelevant
rayyan-283775898	Enhanced Visual Feedback Using Immersive VR Affects Decision Making Regarding Hand Use With a Simulated Impaired Limb	2021	15				Sakabe, N and Altukhaim, S and Hayashi, Y and Sakurada, T and Yano, S and Kondo, T	RAYYAN-INCLUSION: {"Vickie"=>"Excluded", "jsong6"=>"Excluded"} | RAYYAN-EXCLUSION-REASONS: irrelevant
rayyan-283775899	Lateralized processing of centrally presented verbal and spatial tasks: effects of attention, gender, and hand used	1995	8	2	88â€98		Freeman, FG and Pearson, G and Evans, D	RAYYAN-INCLUSION: {"Vickie"=>"Excluded", "jsong6"=>"Excluded"} | RAYYAN-EXCLUSION-REASONS: irrelevant
rayyan-283775900	Loss of sensory attenuation in patients with functional (psychogenic) movement disorders	2014	137	11	2916â€2921		Parees, I and Brown, H and Nuruki, A and Adams, RA and Davare, M and Bhatia, KP and Friston, K and Edwards, MJ	RAYYAN-INCLUSION: {"Vickie"=>"Excluded", "jsong6"=>"Excluded"} | RAYYAN-EXCLUSION-REASONS: irrelevant
rayyan-283775901	Oxytocin enhances the pain-relieving effects of social support in romantic couples	2019	40	1	242â€251		Kreuder, AK and Wassermann, L and Wollseifer, M and Ditzen, B and Eckstein, M and Stoffel-Wagner, B and Hennig, J and Hurlemann, R and Scheele, D	RAYYAN-INCLUSION: {"Vickie"=>"Excluded", "jsong6"=>"Excluded"} | RAYYAN-EXCLUSION-REASONS: irrelevant
rayyan-283775902	Skill transfer from virtual reality to a real laparoscopic task	2001	15	10	1076â€1079		Torkington, J and Smith, SG and Rees, BI and Darzi, A	RAYYAN-INCLUSION: {"Vickie"=>"Excluded", "jsong6"=>"Excluded"} | RAYYAN-EXCLUSION-REASONS: irrelevant
rayyan-283775903	Simultaneous transcranial and transcutaneous spinal direct current stimulation to enhance athletic performance outcome in experienced boxers	2021	11	1		19722	Kamali, AM and Kazemiha, M and Keshtkarhesamabadi, B and Daneshvari, M and Zarifkar, A and Chakrabarti, P and Kateb, B and Nami, M	RAYYAN-INCLUSION: {"Vickie"=>"Excluded", "jsong6"=>"Excluded"} | RAYYAN-EXCLUSION-REASONS: irrelevant
rayyan-283775904	Comparison of 8-MOP aqueous bath and 8-MOP ethanolic lotion (Meladinine) in local PUVA therapy	1998	197	1	25â€30		Shephard, SE and Schregenberger, N and Dummer, R and Panizzon, RG	RAYYAN-INCLUSION: {"Vickie"=>"Excluded", "jsong6"=>"Excluded"} | RAYYAN-EXCLUSION-REASONS: irrelevant
rayyan-283775905	A novel neurocognitive rehabilitation tool in the recovery of hemiplegic hand grip after stroke: a case report	2019	34	1	53â€57		Paolucci, T and Capobianco, SV and Vinciguerra, SM and Cilli, M and Villani, C and Saraceni, VM	RAYYAN-INCLUSION: {"Vickie"=>"Excluded", "jsong6"=>"Excluded"} | RAYYAN-EXCLUSION-REASONS: irrelevant
rayyan-283775906	Repetitive transcranial magnetic stimulation of dorsolateral prefrontal cortex increases tolerance to human experimental pain	2005	25	1	153â€160		Graff-Guerrero, A and GonzÃ¡lez-Olvera, J and FresÃ¡n, A and GÃ³mez-MartÃn, D and MÃ©ndez-NÃºÃ±ez, JC and Pellicer, F	RAYYAN-INCLUSION: {"Vickie"=>"Excluded", "jsong6"=>"Excluded"} | RAYYAN-EXCLUSION-REASONS: irrelevant
rayyan-283775907	Effect of levodopa treatment for parkinsonism in welders: a double-blind study	2004	62	5	730â€733		Koller, WC and Lyons, KE and Truly, W	RAYYAN-INCLUSION: {"Vickie"=>"Excluded", "jsong6"=>"Excluded"} | RAYYAN-EXCLUSION-REASONS: irrelevant
rayyan-283775908	Six months of hormone replacement therapy does not influence muscle strength in postmenopausal women	2002	42	3	225â€231		Ribom, EL and Piehl-Aulin, K and Ljunghall, S and Ljunggren, O and NaessÃ©n, T	RAYYAN-INCLUSION: {"Vickie"=>"Excluded", "jsong6"=>"Excluded"} | RAYYAN-EXCLUSION-REASONS: irrelevant
rayyan-283775909	Interim results on the clinical effects of i.v. administered thymopentin in active rheumatoid arthritis	1984	4	6	451â€457		Malaise, M and Franchimont, P and Hauwaert, C and Danneskiold-Samsoe, B and Gross, D and Gerber, H and Gerschpacher, H and Bolla, K	RAYYAN-INCLUSION: {"Vickie"=>"Excluded", "jsong6"=>"Excluded"} | RAYYAN-EXCLUSION-REASONS: irrelevant
rayyan-283775910	The use of a radiolucent template to improve bone age X-ray quality (BASIC study)	2017	30	12	1277â€1280		Cockill, TG and Hewitt, A and Heafey, C and Wright, NP and Elder, CJ	RAYYAN-INCLUSION: {"Vickie"=>"Excluded", "jsong6"=>"Excluded"} | RAYYAN-EXCLUSION-REASONS: irrelevant
rayyan-283775911	An integrated experiential training programme with coaching to promote physical activity, and reduce fatigue among children with cancer: a randomised controlled trial	2018	101	11	1947â€1956		Lam, KKW and Li, WHC and Chung, OK and Ho, KY and Chiu, SY and Lam, HS and Chan, GCF	RAYYAN-INCLUSION: {"Vickie"=>"Excluded", "jsong6"=>"Excluded"} | RAYYAN-EXCLUSION-REASONS: irrelevant
rayyan-283775912	The action of aspirin on plasma kininogen and other plasma proteins in rheumatoid patients: relationship to disease activity	1980	7	4	347â€354		Sharma, JN and Zeitlin, IJ and Brooks, PM and Buchanan, WW and Dick, WC	RAYYAN-INCLUSION: {"Vickie"=>"Excluded", "jsong6"=>"Excluded"} | RAYYAN-EXCLUSION-REASONS: irrelevant
rayyan-283775913	Effect of yoga on musculoskeletal discomfort and motor functions in professional computer users	2009	33	3	297â€306		Telles, S and Dash, M and Naveen, KV	RAYYAN-INCLUSION: {"Vickie"=>"Excluded", "jsong6"=>"Excluded"} | RAYYAN-EXCLUSION-REASONS: irrelevant
rayyan-283775914	Adaptability to pain is associated with potency of local pain inhibition, but not conditioned pain modulation: a healthy human study	2014	155	5	968â€976		Zheng, Z and Wang, K and Yao, D and Xue, CCL and Arendt-Nielsen, L	RAYYAN-INCLUSION: {"Vickie"=>"Excluded", "jsong6"=>"Excluded"} | RAYYAN-EXCLUSION-REASONS: irrelevant
rayyan-283775915	Control of a trackball by the chin for communication applications, with and without neck movements	1997	42	3	213â€218		Jacobs, R and Hendrickx, E and Van Mele, I and Edwards, K and Verheust, M and Spaepen, A and van Steenberghe, D	RAYYAN-INCLUSION: {"Vickie"=>"Excluded", "jsong6"=>"Excluded"} | RAYYAN-EXCLUSION-REASONS: irrelevant
rayyan-283775916	Distance and location assimilation effects in rapid bimanual movement	1991	62	3	302â€308		Sherwood, DE	RAYYAN-INCLUSION: {"Vickie"=>"Excluded", "jsong6"=>"Excluded"} | RAYYAN-EXCLUSION-REASONS: irrelevant
rayyan-283775917	Hemispheric asymmetries of a motor memory in a recognition test after learning a movement sequence	2016	171					RAYYAN-INCLUSION: {"Vickie"=>"Excluded", "jsong6"=>"Excluded"} | RAYYAN-EXCLUSION-REASONS: irrelevant,wrong study design
rayyan-283775918	Effect of Verbal Empathy and Touch on Anxiety Relief in Patients Undergoing Flexible Bronchoscopy: can Empathy Reduce Patients' Anxiety?	2016	92	6	380â€388		Choi, SM and Lee, J and Park, YS and Lee, CH and Lee, SM and Yim, JJ	RAYYAN-INCLUSION: {"Vickie"=>"Excluded", "jsong6"=>"Excluded"} | RAYYAN-EXCLUSION-REASONS: irrelevant
rayyan-283775919	Immediate and delayed effects of treatment at the Dead Sea in patients with psoriatic arthritis	2000	19	3	77â€82		Elkayam, O and Ophir, J and Brener, S and Paran, D and Wigler, I and Efron, D and Even-Paz, Z and Politi, Y and Yaron, M	RAYYAN-INCLUSION: {"Vickie"=>"Excluded", "jsong6"=>"Excluded"} | RAYYAN-EXCLUSION-REASONS: acute,irrelevant
rayyan-283775920	Primary somatosensory cortex is actively involved in pain processing in human	2000	853	2	282â€289		Kanda, M and Nagamine, T and Ikeda, A and Ohara, S and Kunieda, T and Fujiwara, N and Yazawa, S and Sawamoto, N and Matsumoto, R and Taki, W and Shibasaki, H	RAYYAN-INCLUSION: {"Vickie"=>"Excluded", "jsong6"=>"Excluded"} | RAYYAN-EXCLUSION-REASONS: acute,irrelevant
rayyan-283775921	Effects of subthreshold transcranial magnetic stimulation on choice reaction time and correlation with motor cortical activation	1999	45	3	165â€179		Sawaki, L and Okita, T and Fujiwara, M and Mizuno, K	RAYYAN-INCLUSION: {"Vickie"=>"Excluded", "jsong6"=>"Excluded"} | RAYYAN-EXCLUSION-REASONS: acute,irrelevant
rayyan-283775922	The role of auditory and visual models in the production of bimanual tapping patterns	2013	224	4	507â€518		Kennedy, DM and Boyle, JB and Shea, CH	RAYYAN-INCLUSION: {"Vickie"=>"Excluded", "jsong6"=>"Excluded"} | RAYYAN-EXCLUSION-REASONS: acute,irrelevant
rayyan-283775923	Differential efficiency of transcutaneous electrical nerve stimulation in dominant versus nondominant hands in fibromyalgia: placebo-controlled functional near-infrared spectroscopy study	2018	5	1			Eken, A and Kara, M and Baskak, B and Baltaci, A and Gokcay, D	RAYYAN-INCLUSION: {"Vickie"=>"Excluded", "jsong6"=>"Excluded"} | RAYYAN-EXCLUSION-REASONS: irrelevant,no right and left limb training
rayyan-283775924	Confirmative study of the effectiveness of thymopentin in active rheumatoid arthritis	1985	4		87â€93		Malaise, M and Franchimont, P and Hauwaert, C and Danneskiold-Samsoe, B and Gross, D and Gerber, H and Gerschpacher, H and Bolla, K	RAYYAN-INCLUSION: {"Vickie"=>"Excluded", "jsong6"=>"Excluded"} | RAYYAN-EXCLUSION-REASONS: irrelevant
rayyan-283775925	Effects of flunitrazepam on responses to lateralized visual stimuli: evidence for cerebral asymmetry of execution of manual movements to targets in contralateral and ipsilateral visual space	1994	114	4	551â€558		Ingum, J and BjÃ¸rklund, R	RAYYAN-INCLUSION: {"Vickie"=>"Excluded", "jsong6"=>"Excluded"} | RAYYAN-EXCLUSION-REASONS: irrelevant
rayyan-283775926	Functional deterioration and selenium-vitamin E treatment in myotonic dystrophy. A placebo-controlled study	1994	235	3	205â€210		Orndahl, G and Grimby, G and Grimby, A and Johansson, G and Wilhelmsen, L	RAYYAN-INCLUSION: {"Vickie"=>"Excluded", "jsong6"=>"Excluded"} | RAYYAN-EXCLUSION-REASONS: irrelevant
rayyan-283775927	Radiological Features in Patients with Short Stature Homeobox-Containing (SHOX) Gene Deficiency and Turner Syndrome before and after 2 Years of GH Treatment	2015	84	1	14â€25		Child, CJ and Kalifa, G and Jones, C and Ross, JL and Rappold, GA and Quigley, CA and Zimmermann, AG and Garding, G and Cutler, GB and Blum, WF	RAYYAN-INCLUSION: {"Vickie"=>"Excluded", "jsong6"=>"Excluded"} | RAYYAN-EXCLUSION-REASONS: irrelevant
rayyan-283775928	The effects of transcutaneous electrical nerve stimulation on skin temperature in asymptomatic subjects	1995	75	7	621â€628		Scudds, RJ and Helewa, A and Scudds, RA	RAYYAN-INCLUSION: {"Vickie"=>"Excluded", "jsong6"=>"Excluded"} | RAYYAN-EXCLUSION-REASONS: irrelevant
rayyan-283775929	Effectiveness of a Group-Based Progressive Strength Training in Primary Care to Improve the Recurrence of Low Back Pain Exacerbations and Function: a Randomised Trial	2020	17	22			Calatayud, J and GuzmÃ¡n-GonzÃ¡lez, B and Andersen, LL and Cruz-Montecinos, C and Morell, MT and RoldÃ¡n, R and Ezzatvar, Y and CasaÃ±a, J	RAYYAN-INCLUSION: {"Vickie"=>"Excluded", "jsong6"=>"Excluded"} | RAYYAN-EXCLUSION-REASONS: irrelevant
rayyan-283775930	Intermittent Palm Cooling's Impact on Resistive Exercise Performance	2015	36	10	814â€821		Caruso, JF and Barbosa, A and Erickson, L and Edwards, R and Perry, R and Learmonth, L and Potter, WT	RAYYAN-INCLUSION: {"Vickie"=>"Excluded", "jsong6"=>"Excluded"} | RAYYAN-EXCLUSION-REASONS: irrelevant
rayyan-283775931	Two-Layer Wound Sealing before Surgical Hand Washing for Surgeons with a Minor Cut Injury on the Hand	2019	20	5	390â€394		Yoon, C and Gong, HS and Park, JS and Seok, HS and Park, JW and Baek, GH	RAYYAN-INCLUSION: {"Vickie"=>"Excluded", "jsong6"=>"Excluded"} | RAYYAN-EXCLUSION-REASONS: irrelevant
rayyan-283775932	Treatment of active rheumatoid arthritis with slow intravenous injections of thymopentin. A double-blind placebo-controlled randomised study	1985	1	8433	832â€836		Malaise, MG and Hauwaert, C and Franchimont, P and Danneskiold-Samsoe, B and Bach-Andersen, R and Gross, D and Gerber, H and Gerschpacher, H and Stocker, H and Bolla, K	RAYYAN-INCLUSION: {"Vickie"=>"Excluded", "jsong6"=>"Excluded"} | RAYYAN-EXCLUSION-REASONS: irrelevant
rayyan-283775933	Randomized double-blind controlled trial comparing room-temperature and heated lidocaine for digital nerve block	1995	26	6	677â€681		Waldbillig, DK and Quinn, JV and Stiell, IG and Wells, GA	RAYYAN-INCLUSION: {"Vickie"=>"Excluded", "jsong6"=>"Excluded"} | RAYYAN-EXCLUSION-REASONS: irrelevant
rayyan-283775934	Perception of pain after resistance exercise	1998	32	1	20â€24		Koltyn, KF and Arbogast, RW	RAYYAN-INCLUSION: {"Vickie"=>"Excluded", "jsong6"=>"Excluded"} | RAYYAN-EXCLUSION-REASONS: irrelevant
rayyan-283775935	Acquisition of chopstick-operation skills with the non-dominant hand and concomitant changes in brain activity	2019	9	1		20397	Sawamura, D and Sakuraba, S and Suzuki, Y and Asano, M and Yoshida, S and Honke, T and Kimura, M and Iwase, Y and Horimoto, Y and Yoshida, K and Sakai, S	RAYYAN-INCLUSION: {"Vickie"=>"Excluded", "jsong6"=>"Excluded"} | RAYYAN-EXCLUSION-REASONS: irrelevant
rayyan-283775936	Haemodynamic effects of eprosartan and valsartan in hypertensive patients during isometric and mental stress	2005	23	10	1923â€1927		Arosio, E and De Marchi, S and Prior, M and Rigoni, A and Lechi, A	RAYYAN-INCLUSION: {"Vickie"=>"Excluded", "jsong6"=>"Excluded"} | RAYYAN-EXCLUSION-REASONS: irrelevant
rayyan-283775937	Part and whole perceptual-motor practice of a polyrhythm	2003	338	3	205â€208		Kurtz, S and Lee, TD	RAYYAN-INCLUSION: {"Vickie"=>"Excluded", "jsong6"=>"Excluded"} | RAYYAN-EXCLUSION-REASONS: irrelevant
rayyan-283775938	A comparison of the anthropometric, strength, endurance and flexibility characteristics of female elite and recreational climbers and non-climbers	2001	19	7	499â€505		Grant, S and Hasler, T and Davies, C and Aitchison, TC and Wilson, J and Whittaker, A	RAYYAN-INCLUSION: {"Vickie"=>"Excluded", "jsong6"=>"Excluded"} | RAYYAN-EXCLUSION-REASONS: irrelevant
rayyan-283775939	The effect of unilateral somatosensory stimulation on hemispheric asymmetries during slow wave sleep	2004	27	1	63â€68		Cottone, LA and Adamo, D and Squires, NK	RAYYAN-INCLUSION: {"Vickie"=>"Excluded", "jsong6"=>"Excluded"} | RAYYAN-EXCLUSION-REASONS: irrelevant
rayyan-283775940	Allocation of attention to programming of movement sequences in Parkinson's disease	1994	16	1	117â€128		Jones, DL and Bradshaw, JL and Phillips, JG and Iansek, R and Mattingley, JB and Bradshaw, JA	RAYYAN-INCLUSION: {"Vickie"=>"Excluded", "jsong6"=>"Excluded"} | RAYYAN-EXCLUSION-REASONS: irrelevant
rayyan-283775941	Anosognosia for hemiplegia: test of the personal neglect hypothesis	1995	45	12	2195â€2199		Adair, JC and Na, DL and Schwartz, RL and Fennell, EM and Gilmore, RL and Heilman, KM	RAYYAN-INCLUSION: {"Vickie"=>"Excluded", "jsong6"=>"Excluded"} | RAYYAN-EXCLUSION-REASONS: irrelevant
rayyan-283775942	The effects of consecutive sessions of anodal transcranial direct current stimulation over the primary motor cortex on hand function in healthy older adults	2020	89			104063	Rostami, M and Mosallanezhad, Z and Ansari, S and Kidgell, D and Rezaeian, T and Bakhshi, E and Ghodrati, M and Jaberzadeh, S	RAYYAN-INCLUSION: {"Vickie"=>"Excluded", "jsong6"=>"Excluded"} | RAYYAN-EXCLUSION-REASONS: irrelevant
rayyan-283775943	Motor cortex inhibition with somatosensory and transcranial direct current stimulation: a metaplasticity study	2018	32	12	1068â€•		Cirillo, J and Trudgen, A and Byblow, W	RAYYAN-INCLUSION: {"Vickie"=>"Excluded", "jsong6"=>"Excluded"} | RAYYAN-EXCLUSION-REASONS: irrelevant
rayyan-283775944	Mental imagery-induced attention modulates pain perception and cortical excitability	2015	16			15	Volz, MS and Suarez-Contreras, V and Portilla, AL and Fregni, F	RAYYAN-INCLUSION: {"Vickie"=>"Excluded", "jsong6"=>"Excluded"} | RAYYAN-EXCLUSION-REASONS: irrelevant
rayyan-283775945	Haptic unilateral and bilateral discrimination of curved surfaces	1996	25	6	739â€749		Kappers, AM and Koenderink, JJ	RAYYAN-INCLUSION: {"Vickie"=>"Excluded", "jsong6"=>"Excluded"} | RAYYAN-EXCLUSION-REASONS: irrelevant
rayyan-283775946	Comparing the Efficacy of Triamcinolone Acetonide Iontophoresis versus Topical Calcipotriol/Betamethasone Dipropionate in Treating Nail Psoriasis: a Bilateral Controlled Clinical Trial	2018	2018				Saki, N and Hosseinpoor, S and Heiran, A and Mohammadi, A and Zeraatpishe, M	RAYYAN-INCLUSION: {"Vickie"=>"Excluded", "jsong6"=>"Excluded"} | RAYYAN-EXCLUSION-REASONS: irrelevant
rayyan-283775947	Radiation dose measurements for personnel performing 90Y-ibritumomab tiuxetan administration: a comparison between two injection methods for dose reduction	2009	82	978	491â€496		Law, M and Liu, R and Ng, S and Luk, MY and Leung, TW and Au, GK	RAYYAN-INCLUSION: {"Vickie"=>"Excluded", "jsong6"=>"Excluded"} | RAYYAN-EXCLUSION-REASONS: irrelevant
rayyan-283775948	Expectation to feel more pain disrupts the habituation of laser-pain rating and laser-evoked potential amplitudes	2016	333		244â€251		Pazzaglia, C and Testani, E and Giordano, R and Padua, L and Valeriani, M	RAYYAN-INCLUSION: {"Vickie"=>"Excluded", "jsong6"=>"Excluded"} | RAYYAN-EXCLUSION-REASONS: irrelevant,wrong study design
rayyan-283775949	The effect of fluoride containing toothpastes on sound human enamel in vivo during 3 weeks	1988	16	2	95â€99		Gelhard, TB and Arends, J	RAYYAN-INCLUSION: {"Vickie"=>"Excluded", "jsong6"=>"Excluded"} | RAYYAN-EXCLUSION-REASONS: irrelevant
rayyan-283775950	Effects of a person-centered nursing intervention for frailty among prefrail community-dwelling older adults	2020	17	18	1â€19		Ha, J and Park, Y-H	RAYYAN-INCLUSION: {"Vickie"=>"Excluded", "jsong6"=>"Excluded"} | RAYYAN-EXCLUSION-REASONS: irrelevant
rayyan-283775951	Effect of occupational exposure to rayon manufacturing chemicals on skin barrier to evaporative water loss	2004	46	5	410â€417		Chou, TC and Shih, TS and Tsai, JC and Wu, JD and Sheu, HM and Chang, HY	RAYYAN-INCLUSION: {"Vickie"=>"Excluded", "jsong6"=>"Excluded"} | RAYYAN-EXCLUSION-REASONS: irrelevant
rayyan-283775952	Efficacy of intravenous acetaminophen and lidocaine on propofol injection pain	2008	100	1	95â€98		Canbay, O and Celebi, N and Arun, O and KaragÃ¶z, AH and SaricaoÄŸlu, F and Ozgen, S	RAYYAN-INCLUSION: {"Vickie"=>"Excluded", "jsong6"=>"Excluded"} | RAYYAN-EXCLUSION-REASONS: irrelevant
rayyan-283775953	Changes in bite force and muscle forces in the upper extremities after counter irritation	2004	22	1	45â€49		Fukura, M and Kashima, K and Maeda, S and Shiba, R	RAYYAN-INCLUSION: {"Vickie"=>"Excluded", "jsong6"=>"Excluded"} | RAYYAN-EXCLUSION-REASONS: irrelevant
rayyan-283775954	Sympathetic nervous system effects in the hands following a grade III postero-anterior rotatory mobilisation technique applied to T4: a randomised, placebo-controlled trial	2010	15	3	248â€253		Jowsey, P and Perry, J	RAYYAN-INCLUSION: {"Vickie"=>"Excluded", "jsong6"=>"Excluded"} | RAYYAN-EXCLUSION-REASONS: irrelevant
rayyan-283775955	Assessment of the effectiveness of low-level laser therapy on the hands of patients with rheumatoid arthritis: a randomized double-blind controlled trial	2010	29	5	501â€509		Meireles, SM and Jones, A and Jennings, F and Suda, AL and Parizotto, NA and Natour, J	RAYYAN-INCLUSION: {"Vickie"=>"Excluded", "jsong6"=>"Excluded"} | RAYYAN-EXCLUSION-REASONS: irrelevant
rayyan-283775956	Finger cooling by contact with cold aluminium surfaces--effects of pressure, mass and whole body thermal balance	1994	69	1	55â€60		Chen, F and Nilsson, H and HolmÃ©r, I	RAYYAN-INCLUSION: {"Vickie"=>"Excluded", "jsong6"=>"Excluded"} | RAYYAN-EXCLUSION-REASONS: irrelevant
rayyan-283775957	Burn injury rehabilitation: hand dyskinesia and finger pain, treatment by orthokinetic orthoses	1985	6	6	495â€500		Neeman, RL	RAYYAN-INCLUSION: {"Vickie"=>"Excluded", "jsong6"=>"Excluded"} | RAYYAN-EXCLUSION-REASONS: irrelevant
rayyan-283775958	An intensive combined training program modulates physical, physiological, biomotoric, and technical parameters in women basketball players	2008	22	6	1769â€1778		KilinÃ§, F	RAYYAN-INCLUSION: {"Vickie"=>"Excluded", "jsong6"=>"Excluded"} | RAYYAN-EXCLUSION-REASONS: irrelevant
rayyan-283775959	Being Adaptive to Pain Enhances Sham Acupuncture Analgesia: A Crossover Healthy Human Study	2017	10	6	385â€395		Zheng, Z and Wong Lit Wan, D and Arendt-Nielsen, L and Yao, DY and Iversen, G and Xue, CC and Wang, K	RAYYAN-INCLUSION: {"Vickie"=>"Excluded", "jsong6"=>"Excluded"} | RAYYAN-EXCLUSION-REASONS: irrelevant
rayyan-283775960	Effect of L-citrulline on improving sensitivity of Cold - A randomized, placebo-controlled, double-blind, crossover study	2018	46	5	771â€780		Ueda, K and Kirimoto, T and Hasegawa, E and Ando, T and Hayashi, M and Nakamura, F and Sakano, K and Fujiwara, S and Ebihara, S	RAYYAN-INCLUSION: {"Vickie"=>"Excluded", "jsong6"=>"Excluded"} | RAYYAN-EXCLUSION-REASONS: irrelevant

rayyan-283775961	Post-operative pain management following arthroscopic rotator cuff repair. A comparison of inter-scalene block with subacromial bursal infusion	2007					ACTRN12607000150448,	RAYYAN-INCLUSION: {"Vickie"=>"Excluded", "jsong6"=>"Excluded"} | RAYYAN-EXCLUSION-REASONS: irrelevant
rayyan-283775962	An integrated experiential training programme with coaching to promote physical activity, and reduce fatigue among children with cancer: a randomised controlled trial	2018					Lam, KKW and Li, WHC and Chung, OK and Ho, KY and Chiu, SY and Lam, HS and Chan, GCF	RAYYAN-INCLUSION: {"Vickie"=>"Excluded", "jsong6"=>"Excluded"} | RAYYAN-EXCLUSION-REASONS: irrelevant
rayyan-283775963	Medium-dose-UVA-1 irradiation - and topical PUVA - Therapy in chronic dyshidrotic hand dermatitis - A prospective randomized study	2007	33	4	142â€“145		Adams, S and Bayerl, C	RAYYAN-INCLUSION: {"Vickie"=>"Excluded", "jsong6"=>"Excluded"} | RAYYAN-EXCLUSION-REASONS: irrelevant
rayyan-283775964	Successful placement of laryngeal mask in children	2020					IRCT20190325043107N17,	RAYYAN-INCLUSION: {"Vickie"=>"Excluded", "jsong6"=>"Excluded"} | RAYYAN-EXCLUSION-REASONS: irrelevant
rayyan-283775965	Effects of Combination Therapy of Baclofen & Trihexyphenidyl on Spasticity in Brain Injury Patients	2020					IRCT20200415047096N1,	RAYYAN-INCLUSION: {"Vickie"=>"Excluded", "jsong6"=>"Excluded"} | RAYYAN-EXCLUSION-REASONS: irrelevant
rayyan-283775966	Comparison of the effectiveness of DPI intubation procedure in comparison with conventional ITL method on improving the symptoms and overall outcome of intubation	2020					IRCT20200720048145N1,	RAYYAN-INCLUSION: {"Vickie"=>"Excluded", "jsong6"=>"Excluded"} | RAYYAN-EXCLUSION-REASONS: irrelevant
rayyan-283775967	Selective attention effects on somatosensory evoked potentials	1989	45	3	277â€“282		Papanicolaou, AC and Moore, BD and Gary, HE	RAYYAN-INCLUSION: {"Vickie"=>"Excluded", "jsong6"=>"Excluded"} | RAYYAN-EXCLUSION-REASONS: irrelevant
rayyan-283775968	Simple reaction time to lateralized visual stimuli	1980	50	3	193â€“211		Borutti, G and Cosi, V and Magrotti, E and Cerutti, G	RAYYAN-INCLUSION: {"Vickie"=>"Excluded", "jsong6"=>"Excluded"} | RAYYAN-EXCLUSION-REASONS: irrelevant
rayyan-283775969	DRA vs PRA for US-guided Radial Artery Catheterization in ICU	2021					NCT04878887,	RAYYAN-INCLUSION: {"Vickie"=>"Excluded", "jsong6"=>"Excluded"} | RAYYAN-EXCLUSION-REASONS: irrelevant
rayyan-283775970	The Effect Of Empowerment Program On Blood Pressure Of Patient With Type Two Diabetes	2014					IRCT2013080312493N2,	RAYYAN-INCLUSION: {"Vickie"=>"Excluded", "jsong6"=>"Excluded"} | RAYYAN-EXCLUSION-REASONS: acute,irrelevant
rayyan-283775971	"What" becoming "where": functional magnetic resonance imaging evidence for pragmatic relevance driving premotor cortex	2004	24	46	10431â€“10439		Wolfensteller, U and Schubotz, RI and Von Cramon, DY	RAYYAN-INCLUSION: {"Vickie"=>"Excluded", "jsong6"=>"Excluded"} | RAYYAN-EXCLUSION-REASONS: irrelevant
rayyan-283775972	Role of preemptive stellate ganglion blockade in prevention of radial artery spasm during off-pump CABG	2003	19	2	137â€“141		El Raouf, MA and El-Regal, EA and El-Halafawy, Y and Helmy, T and El-Henawy, A	RAYYAN-INCLUSION: {"Vickie"=>"Excluded", "jsong6"=>"Excluded"} | RAYYAN-EXCLUSION-REASONS: irrelevant
rayyan-283775973	A Randomized Comparison of Pediatric I-gel, Air-Q With Classic Laryngeal Mask Airway	2016					NCT02757820,	RAYYAN-INCLUSION: {"Vickie"=>"Excluded", "jsong6"=>"Excluded"} | RAYYAN-EXCLUSION-REASONS: irrelevant
rayyan-283775974	Pacing Activity Self-management for Patients With Chronic Fatigue Syndrome	2012					NCT01512342,	RAYYAN-INCLUSION: {"Vickie"=>"Excluded", "jsong6"=>"Excluded"} | RAYYAN-EXCLUSION-REASONS: irrelevant
rayyan-283775975	Fractional photothermolysis for photoaging of hands	2008	34	1	73â€“78		Jih, MH and Goldberg, LH and Kimyai-Asadi, A	RAYYAN-INCLUSION: {"Vickie"=>"Excluded", "jsong6"=>"Excluded"} | RAYYAN-EXCLUSION-REASONS: irrelevant
rayyan-283775976	Different activation of opercular and posterior cingulate cortex (PCC) in patients with complex regional pain syndrome (CRPS I) compared with healthy controls during perception of electrically induced pain: a functiona	2010	26	4	339â€“347		Freund, W and Wunderlich, AP and Stuber, G and Mayer, F and Steffen, P and Mentzel, M and Weber, F and Schmitz, B	RAYYAN-INCLUSION: {"Vickie"=>"Excluded", "jsong6"=>"Excluded"} | RAYYAN-EXCLUSION-REASONS: irrelevant
rayyan-283775977	Cumulative effects of anodal and priming cathodal tDCS on pegboard test performance and motor cortical excitability	2015	287		27â€“33		Christova, M and Rafolt, D and Gallasch, E	RAYYAN-INCLUSION: {"Vickie"=>"Excluded", "jsong6"=>"Excluded"} | RAYYAN-EXCLUSION-REASONS: irrelevant
rayyan-283775978	Assessment of Magnesium sulphate and ondansetronon on propofol 2% injection pain	2013					IRCT2013092613182N3,	RAYYAN-INCLUSION: {"Vickie"=>"Excluded", "jsong6"=>"Excluded"} | RAYYAN-EXCLUSION-REASONS: irrelevant
rayyan-283775979	Comparison of the effectiveness of painting onto the hand and immersing the hand in a bag, in pre-operative skin preparation of the hand	2009	34	3	371â€“373		Incoll, I and Saravanja, D and Thorvaldson, KT and Small, T	RAYYAN-INCLUSION: {"Vickie"=>"Excluded", "jsong6"=>"Excluded"} | RAYYAN-EXCLUSION-REASONS: irrelevant
rayyan-283775980	Effect of virtual reality training using leap motion controller on impairments and disability in patients with wrist and hand stiffness	2020	11	11	210â€“219		Dabholkar, TY and Shah, SS	RAYYAN-INCLUSION: {"Vickie"=>"Excluded", "jsong6"=>"Excluded"} | RAYYAN-EXCLUSION-REASONS: irrelevant
rayyan-283775981	Piroxicam gel, compared to EMLA cream is associated with less pain after venous cannulation in volunteers	2003	50	8	775â€“778		Dutta, A and Puri, GD and Wig, J	RAYYAN-INCLUSION: {"Vickie"=>"Excluded", "jsong6"=>"Excluded"} | RAYYAN-EXCLUSION-REASONS: irrelevant
rayyan-283775982	No-go dominant brain activity in human inferior prefrontal cortex revealed by functional magnetic resonance imaging	1998	10	3	1209â€“1213		Konishi, S and Nakajima, K and Uchida, I and Sekihara, K and Miyashita, Y	RAYYAN-INCLUSION: {"Vickie"=>"Excluded", "jsong6"=>"Excluded"} | RAYYAN-EXCLUSION-REASONS: irrelevant
rayyan-283775983	Effect of acupuncture on perception threshold: a randomised controlled trial	2012	30	1	32â€“36		Wu, S and Yamaguchi, H and Shibutani, K	RAYYAN-INCLUSION: {"Vickie"=>"Excluded", "jsong6"=>"Excluded"} | RAYYAN-EXCLUSION-REASONS: irrelevant
rayyan-283775984	The effect of combined functional training on BDNF, IGF-1, and their association with health-related fitness in the multiple sclerosis women	2020	52			101320	Abbaspoor, E and Zolfaghari, M and Ahmadi, B and Khodaei, K	RAYYAN-INCLUSION: {"Vickie"=>"Excluded", "jsong6"=>"Excluded"} | RAYYAN-EXCLUSION-REASONS: irrelevant
rayyan-283775985	Methylphenidate and motor organization in children with ADHD	1997					Renaud, J and Bourassa, M and Douglas, VI and Pelletier, G and Geoffroy, G and Robaey, P	RAYYAN-INCLUSION: {"Vickie"=>"Excluded", "jsong6"=>"Excluded"} | RAYYAN-EXCLUSION-REASONS: irrelevant
rayyan-283775986	Modulation effect of transcranial direct current stimulation on phase synchronization in motor imagery brain-computer interface	2014	2014		1270â€“1273		He, W and Wei, P and Zhou, Y and Wang, L	RAYYAN-INCLUSION: {"Vickie"=>"Excluded", "jsong6"=>"Excluded"} | RAYYAN-EXCLUSION-REASONS: irrelevant
rayyan-283775988	A Prospective Trial Comparing Q-Switched Ruby Laser and a Triple Combination Skin-Lightening Cream in the Treatment of Solar Lentigines	2016	42	7	853â€“857		Imhof, L and Dummer, R and Dreier, J and Kolm, I and Barysch, MJ	RAYYAN-INCLUSION: {"Vickie"=>"Excluded", "jsong6"=>"Excluded"} | RAYYAN-EXCLUSION-REASONS: irrelevant
rayyan-283775990	Effects of anodal high-definition transcranial direct current stimulation on bilateral sensorimotor cortex activation during sequential finger movements: an fnirs study	2016	876		351â€“359		Muthalib, M and Besson, P and Rothwell, J and Ward, T and Perrey, S	RAYYAN-INCLUSION: {"Vickie"=>"Excluded", "jsong6"=>"Excluded"} | RAYYAN-EXCLUSION-REASONS: irrelevant
rayyan-283775991	Somatotopic activation of opioid systems by target-directed expectations of analgesia	1999	19	9	3639â€“3648		Benedetti, F and Arduino, C and Amanzio, M	RAYYAN-INCLUSION: {"Vickie"=>"Excluded", "jsong6"=>"Excluded"} | RAYYAN-EXCLUSION-REASONS: irrelevant
rayyan-283775992	Stimulus-response compatibility in representational space	1998	36	8	731â€“735		Bachtold, D and Baumuller, M and Brugger, P	RAYYAN-INCLUSION: {"Vickie"=>"Excluded", "jsong6"=>"Excluded"} | RAYYAN-EXCLUSION-REASONS: irrelevant
rayyan-283775993	Masticatory Side Change and Masticatory Function	2021					NCT04714437,	RAYYAN-INCLUSION: {"Vickie"=>"Excluded", "jsong6"=>"Excluded"} | RAYYAN-EXCLUSION-REASONS: irrelevant
rayyan-283775994	Experimental studies on the effects of vibration and noise on sympathetic nerve activity in skin	1991	62	5	324â€“331		Okada, A and Naito, M and Ariizumi, M and Inaba, R	RAYYAN-INCLUSION: {"Vickie"=>"Excluded", "jsong6"=>"Excluded"} | RAYYAN-EXCLUSION-REASONS: irrelevant
rayyan-283775995	Is the somatosensory N250 related to deviance discrimination or conscious target detection?	1996	100	2	115â€“125		Kekoni, J and Hamalainen, H and McCloud, V and Reinikainen, K and Naatanen, R	RAYYAN-INCLUSION: {"Vickie"=>"Excluded", "jsong6"=>"Excluded"} | RAYYAN-EXCLUSION-REASONS: irrelevant
rayyan-283775996	Effect of yoga based lifestyle intervention on patients with knee osteoarthritis: a randomized controlled trial	2018	9				Deepeshwar, S and Tanwar, M and Kavuri, V and Budhi, RB	RAYYAN-INCLUSION: {"Vickie"=>"Excluded", "jsong6"=>"Excluded"} | RAYYAN-EXCLUSION-REASONS: irrelevant
rayyan-283775997	Short-term immobilization influences use-dependent cortical plasticity and fine motor performance	2016	330		247â€“256		Opie, GM and Evans, A and Ridding, MC and Semmler, JG	RAYYAN-INCLUSION: {"Vickie"=>"Excluded", "jsong6"=>"Excluded"} | RAYYAN-EXCLUSION-REASONS: irrelevant
rayyan-283775998	Association of Vitamin D Supplementation in Cardiorespiratory Fitness and Muscle Strength in Adult Twins: A Randomized Controlled Trial	2022	32	1	2â€“7		Medeiros, JFP and Borges, MVO and Soares, AA and de Sousa, EC and Costa, JRRD and Silva, WAC and de Sousa, MVB and Silbiger, VN and Dantas, PMS and Luchessi, AD	RAYYAN-INCLUSION: {"Vickie"=>"Excluded", "jsong6"=>"Excluded"} | RAYYAN-EXCLUSION-REASONS: nutrition,irrelevant
rayyan-283775999	Involuntary orientation of attention to unattended deviant nociceptive stimuli is modulated by concomitant visual task difficulty. Evidence from laser evoked potentials	2005	116	9	2165â€“2174		Legrain, V and Bruyer, R and GuÃ©rit, JM and Plaghki, L	RAYYAN-INCLUSION: {"Vickie"=>"Excluded", "jsong6"=>"Excluded"} | RAYYAN-EXCLUSION-REASONS: irrelevant
rayyan-283776000	Vibratory and tactile sense of the fingers after working with sanders	1985	56	3	217â€“223		Verberk, MM and Salle, HJA and Kempers, O	RAYYAN-INCLUSION: {"Vickie"=>"Excluded", "jsong6"=>"Excluded"} | RAYYAN-EXCLUSION-REASONS: irrelevant
rayyan-283776001	Efficacy comparison of two methods of r3 sympathicotomy for palmar hyperhidrosis	2014	94	47	3745â€“3747		Lin, M and Tu, Y and Chen, J and Li, X and Lai, F and Lin, J	RAYYAN-INCLUSION: {"Vickie"=>"Excluded", "jsong6"=>"Excluded"} | RAYYAN-EXCLUSION-REASONS: irrelevant
rayyan-283776002	Experimental pain sensitivity in women with temporomandibular disorders and pain-free controls: the relationship to orofacial muscular contraction and cardiovascular responses	2008	24	4	343â€“352		Mohn, C and Vassend, O and Knardahl, S	RAYYAN-INCLUSION: {"Vickie"=>"Excluded", "jsong6"=>"Excluded"} | RAYYAN-EXCLUSION-REASONS: acute,irrelevant
rayyan-283776003	Safety and Efficacy of Ingenol Mebutate 0.05% Gel When Used After Cryotherapy in the Hypertrophic Actinic Keratoses	2014					NCT02251652,	RAYYAN-INCLUSION: {"Vickie"=>"Excluded", "jsong6"=>"Excluded"} | RAYYAN-EXCLUSION-REASONS: irrelevant
rayyan-283776004	The effect of acupressure on anxiety and hemodynamic indices of elderly with hypertension	2021					IRCT20210807052097N1,	RAYYAN-INCLUSION: {"Vickie"=>"Excluded", "jsong6"=>"Excluded"} | RAYYAN-EXCLUSION-REASONS: irrelevant
rayyan-283776005	Sequence for estimating bone age using a combination of manual and automated methods: Application in forensic medicine	2021	12	3	116â€“125		Delage, C and Le Berre, T and Dumousset, E and Garcier, JM and Pereira, B and Boyer, L	RAYYAN-INCLUSION: {"Vickie"=>"Excluded", "jsong6"=>"Excluded"} | RAYYAN-EXCLUSION-REASONS: irrelevant
rayyan-283776006	Sensorimotor Representation and Functional Motor Changes Following Short-Term Arm Immobilization	2018					Debarnot, U and Huber, C and Guillot, A and Schwartz, S	RAYYAN-INCLUSION: {"Vickie"=>"Excluded", "jsong6"=>"Excluded"} | RAYYAN-EXCLUSION-REASONS: irrelevant
rayyan-283776007	Leak rate of latex gloves after tearing adhesive tape	1995	22	3	133â€“137		Cork, RC and Wood, D and Evans, B and deLanzac, K and Naraghi, M	RAYYAN-INCLUSION: {"Vickie"=>"Excluded", "jsong6"=>"Excluded"} | RAYYAN-EXCLUSION-REASONS: irrelevant
rayyan-283776008	Prevention of intra-thoracic recurrent laryngeal nerve injury with robot-assisted esophagectomy	2020					Hosoda, K and Niihara, M and Ushiku, H and Harada, H and Sakuraya, M and Washio, M and Yamashita, K and Hiki, N	RAYYAN-INCLUSION: {"Vickie"=>"Excluded", "jsong6"=>"Excluded"} | RAYYAN-EXCLUSION-REASONS: irrelevant
rayyan-283776009	Prevention of intra-thoracic recurrent laryngeal nerve injury with robot-assisted esophagectomy	2020	405	4	533â€“540		Hosoda, K and Niihara, M and Ushiku, H and Harada, H and Sakuraya, M and Washio, M and Yamashita, K and Hiki, N	RAYYAN-INCLUSION: {"Vickie"=>"Excluded", "jsong6"=>"Excluded"} | RAYYAN-EXCLUSION-REASONS: irrelevant
rayyan-283776010	Orienting attention in obsessive-compulsive disorder	2003	16	1	68â€“74		Georgiou-Karistianis, N and Howells, D and Bradshaw, J	RAYYAN-INCLUSION: {"Vickie"=>"Excluded", "jsong6"=>"Excluded"} | RAYYAN-EXCLUSION-REASONS: irrelevant
rayyan-283776011	Effect and persistency of botulinum toxin iontophoresis in the treatment of palmar hyperhidrosis	2008	49	2	75â€“79		Davarian, S and Kalantari, KK and Rezasoltani, A and Rahimi, A	RAYYAN-INCLUSION: {"Vickie"=>"Excluded", "jsong6"=>"Excluded"} | RAYYAN-EXCLUSION-REASONS: irrelevant
rayyan-283776012	Achieving coordination in prehension: joint freezing and postural contributions	1995	27	4	333â€“348		Steenbergen, B and Marteniuk, RG and Kalbfleisch, LE	RAYYAN-INCLUSION: {"Vickie"=>"Excluded", "jsong6"=>"Excluded"} | RAYYAN-EXCLUSION-REASONS: irrelevant
rayyan-283776013	Differences in cerebral activation during smooth pursuit and saccadic eye movements using positron-emission tomography	1998	44	8	685â€“689		O'Driscoll, GA and Strakowski, SM and Alpert, NM and Matthysse, SW and Rauch, SL and Levy, DL and Holzman, PS	RAYYAN-INCLUSION: {"Vickie"=>"Excluded", "jsong6"=>"Excluded"} | RAYYAN-EXCLUSION-REASONS: irrelevant
rayyan-283776015	Reliability of an instrumental assessment of tardive dyskinesia: results from VA Cooperative Study 394	1997	132	1	61â€“66		Caligiuri, MP and Lohr, JB and Rotrosen, J and Adler, L and Lavori, P and Edson, R	RAYYAN-INCLUSION: {"Vickie"=>"Excluded", "jsong6"=>"Excluded"} | RAYYAN-EXCLUSION-REASONS: irrelevant
rayyan-283776016	The effect of nail polish and acrylic nails on pulse oximetry reading using the Lifebox oximeter in Nigeria	2013	20	4	331â€“335		Desalu, I and Diakparomre, OI and Salami, AO and Abiola, AO	RAYYAN-INCLUSION: {"Vickie"=>"Excluded", "jsong6"=>"Excluded"} | RAYYAN-EXCLUSION-REASONS: irrelevant
rayyan-283776017	Normative data for modified box and blocks test measuring upper-limb function via motion capture	2014	51	6	919â€“931		Hebert, JS and Lewicke, J and Williams, TR and Vette, AH	RAYYAN-INCLUSION: {"Vickie"=>"Excluded", "jsong6"=>"Excluded"} | RAYYAN-EXCLUSION-REASONS: irrelevant
rayyan-283776018	Solar Lentigines Treatment With the Triple Combination Cream	2009					NCT00975312,	RAYYAN-INCLUSION: {"Vickie"=>"Excluded", "jsong6"=>"Excluded"} | RAYYAN-EXCLUSION-REASONS: irrelevant
rayyan-283776020	Brain mapping of bilateral interactions in attention deficit hyperactivity disorder and control boys	2000	111	7	1141â€“1156		Steger, J and Imhof, K and Steinhausen, H and Brandeis, D	RAYYAN-INCLUSION: {"Vickie"=>"Excluded", "jsong6"=>"Excluded"} | RAYYAN-EXCLUSION-REASONS: irrelevant
rayyan-283776021	Neural coupling of cooperative hand movements: a reflex and fMRI study	2013	25	4	948â€“958		Dietz, V and Macauda, G and Schrafl-Altermatt, M and Wirz, M and Kloter, E and Michels, L	RAYYAN-INCLUSION: {"Vickie"=>"Excluded", "jsong6"=>"Excluded"} | RAYYAN-EXCLUSION-REASONS: irrelevant,no right and left limb training
rayyan-283776022	Comparison Study with Protocol of Local Anesthesia in Open Carpal Tunnel Release - A Prospective, Randomized, Double Blind Study	2011					KCT0000186,	RAYYAN-INCLUSION: {"Vickie"=>"Excluded", "jsong6"=>"Excluded"} | RAYYAN-EXCLUSION-REASONS: irrelevant
rayyan-283776023	Effects of zero and 90 degree abduction of glenohumeral joint on the upper extremity nerve conduction velocity (NCS) in diabetic patients undergoing coronary artery bypass graft surgery	2016	34	373			Hosseinifar, M and Mojahed, Z and Mirmohammad-Sadeghi, M and Azadeh, H	RAYYAN-INCLUSION: {"Vickie"=>"Excluded", "jsong6"=>"Excluded"} | RAYYAN-EXCLUSION-REASONS: irrelevant
rayyan-283776024	Neonatal compartment syndrome: case report	2016	175	11		1831	Bradeanu, AM and Tomita, M and Iancu, M and Balaceanu, R	RAYYAN-INCLUSION: {"Vickie"=>"Excluded", "jsong6"=>"Excluded"} | RAYYAN-EXCLUSION-REASONS: irrelevant
rayyan-283776025	Habituation of the initial responses to cold water immersion in humans: a central or peripheral mechanism?	1998	512	2	621â€“628		Tipton, MJ and Eglin, CM and Golden, FC	RAYYAN-INCLUSION: {"Vickie"=>"Excluded", "jsong6"=>"Excluded"} | RAYYAN-EXCLUSION-REASONS: irrelevant
rayyan-283776026	Assessing synovitis in patients with rheumatoid arthritis by ultrasound-an agreement study exploring the most active side	2019	78		1009â€“1010		Terslev, L and Christensen, R and Aga, A-B and Sexton, J and Haavardsholm, EA and Hammer, HB	RAYYAN-INCLUSION: {"Vickie"=>"Excluded", "jsong6"=>"Excluded"} | RAYYAN-EXCLUSION-REASONS: irrelevant
rayyan-283776027	Comparision of efficacy of PRHgh (Daroopakhsh, Iran) and Norditropin (Novo -Nordisk, Denmark) on growth of short stature children with Growth Hormone deficiency	2012					IRCT201206054794N5,	RAYYAN-INCLUSION: {"Vickie"=>"Excluded", "jsong6"=>"Excluded"} | RAYYAN-EXCLUSION-REASONS: irrelevant
rayyan-283776028	Cervical Epidural Versus Cevical Facet Injection for Patients With Chronic Cervical Pain	2020					NCT04594876,	RAYYAN-INCLUSION: {"Vickie"=>"Excluded", "jsong6"=>"Excluded"} | RAYYAN-EXCLUSION-REASONS: irrelevant
rayyan-283776029	Improved bimanual control in elderly after motor cortex stimulation	2018	129		e197â€•		Jamil, A and Cuypers, K and Rand, MK and Nitsche, MA and Meesen, R	RAYYAN-INCLUSION: {"Vickie"=>"Excluded", "jsong6"=>"Excluded"} | RAYYAN-EXCLUSION-REASONS: irrelevant
rayyan-283776030	REUSABLE LARYNGEAL TUBE SUCTION(LTS-II); STANDARD INSERTION TECHNIQUE VERSUS TWO MODIFIED INSERTION TECHNIQUES USING JAW THRUST TECHNIQUE AND MACINTOSH LARYNGOSCOPY	2016					CTRI/2016/08/007128,	RAYYAN-INCLUSION: {"Vickie"=>"Excluded", "jsong6"=>"Excluded"} | RAYYAN-EXCLUSION-REASONS: irrelevant
rayyan-283776031	Prospective comparison between reduced port laparoscopic hiatal hernia repair and usual 5 port one	2014	28			356	Hattori, K and Tanaka, T and Nakano, K and Toura, I	RAYYAN-INCLUSION: {"Vickie"=>"Excluded", "jsong6"=>"Excluded"} | RAYYAN-EXCLUSION-REASONS: irrelevant
rayyan-283776032	Right-left asymmetry in corticospinal tract microstructure and dexterity are uncoupled in late adulthood	2021	240			118405	Demnitz, N and Madsen, KS and Johnsen, LK and Kjaer, M and Boraxbekk, CJ and Siebner, HR	RAYYAN-INCLUSION: {"Vickie"=>"Excluded", "jsong6"=>"Excluded"} | RAYYAN-EXCLUSION-REASONS: irrelevant
rayyan-283776033	Optimal stimulation site for rTMS to improve motor function: anatomical hand knob vs. hand motor hotspot	2021	740			135424	Kim, H and Kim, J and Lee, HJ and Lee, J and Na, Y and Chang, WH and Kim, YH	RAYYAN-INCLUSION: {"Vickie"=>"Excluded", "jsong6"=>"Excluded"} | RAYYAN-EXCLUSION-REASONS: irrelevant
rayyan-283776034	Effects of High-Definition and Conventional Transcranial Direct-Current Stimulation on Motor Learning in Children	2018	12				Cole, L and Giuffre, A and Ciechanski, P and Carlson, HL and Zewdie, E and Kuo, H-C and Kirton, A	RAYYAN-INCLUSION: {"Vickie"=>"Excluded", "jsong6"=>"Excluded"} | RAYYAN-EXCLUSION-REASONS: irrelevant
rayyan-283776035	Startle-induced reaction time shortening is not modified by prepulse inhibition	2005	165	4	541â€“548		Valls-SolÃ©, J and Kofler, M and Kumru, H and Castellote, JM and Sanegre, MT	RAYYAN-INCLUSION: {"Vickie"=>"Excluded", "jsong6"=>"Excluded"} | RAYYAN-EXCLUSION-REASONS: irrelevant
rayyan-283776036	Losing my hand. Body ownership attenuation after virtual lesion of the primary motor cortex	2018	48	6	2272â€“2287		Fossataro, C and Bruno, V and Giurgola, S and Bolognini, N and Garbarini, F	RAYYAN-INCLUSION: {"Vickie"=>"Excluded", "jsong6"=>"Excluded"} | RAYYAN-EXCLUSION-REASONS: irrelevant
rayyan-283776037	Losing my hand. Body ownership attenuation after virtual lesion of the primary motor cortex	2018	48	6	2272â€“2287		Fossataro, C and Bruno, V and Giurgola, S and Bolognini, N and Garbarini, F	RAYYAN-INCLUSION: {"Vickie"=>"Excluded", "jsong6"=>"Excluded"} | RAYYAN-EXCLUSION-REASONS: irrelevant
rayyan-283776038	Effects of dual-mode non-invasive brain stimulation on motor function	2014	567		24â€“29		Park, E and Kim, YH and Chang, WH and Kwon, TG and Shin, YI	RAYYAN-INCLUSION: {"Vickie"=>"Excluded", "jsong6"=>"Excluded"} | RAYYAN-EXCLUSION-REASONS: irrelevant
rayyan-283776039	Comparison of intradialytic versus home-based exercise programs on physical functioning, physical activity level, adherence, and health-related quality of life: pilot study	2020	10	1		8302	Ortega-PÃ©rez de Villar, L and MartÃ-nez-Olmos, FJ and PÃ©rez-DomÃ-nguez, FB and Benavent-Caballer, V and MontaÃ±ez-Aguilera, FJ and Mercer, T and Segura-OrtÃ-, E	RAYYAN-INCLUSION: {"Vickie"=>"Excluded", "jsong6"=>"Excluded"} | RAYYAN-EXCLUSION-REASONS: irrelevant
rayyan-283776040	The effect of folate and cobalamin on osteoarthritic hands	1994	13	4	351â€“356		Flynn, MA and Irvin, W and Krause, G	RAYYAN-INCLUSION: {"Vickie"=>"Excluded", "jsong6"=>"Excluded"} | RAYYAN-EXCLUSION-REASONS: irrelevant
rayyan-283776041	Altered cerebral pain processing of noxious stimuli from inflamed joints in rheumatoid arthritis: an event-related fMRI study	2019	81		272â€“279		SandstrÃ¶m, A and Ellerbrock, I and Jensen, KB and Martinsen, S and Altawil, R and Hakeberg, P and Fransson, P and Lampa, J and Kosek, E	RAYYAN-INCLUSION: {"Vickie"=>"Excluded", "jsong6"=>"Excluded"} | RAYYAN-EXCLUSION-REASONS: irrelevant
rayyan-283776042	Transcranial Direct-Current Stimulation Can Enhance Motor Learning in Children	2017	27	5	2758â€“2767		Ciechanski, P and Kirton, A	RAYYAN-INCLUSION: {"Vickie"=>"Excluded", "jsong6"=>"Excluded"} | RAYYAN-EXCLUSION-REASONS: irrelevant
rayyan-283776043	A pilot study of functional magnetic resonance imaging of the brain during manual and electroacupuncture stimulation of acupuncture point (LI-4 Hegu) in normal subjects reveals differential brain activation between	2002	8	4	411â€“419		Kong, J and Ma, L and Gollub, RL and Wei, J and Yang, X and Li, D and Weng, X and Jia, F and Wang, C and Li, F and Li, R and Zhuang, D	RAYYAN-INCLUSION: {"Vickie"=>"Excluded", "jsong6"=>"Excluded"} | RAYYAN-EXCLUSION-REASONS: irrelevant
rayyan-283776044	Effects of an energy and micronutrient supplement on skeletal maturation in undernourished children in Indonesia	2000	54		S74â€“9		Jahari, AB and Haas, J and Husaini, MA and Pollitt, E	RAYYAN-INCLUSION: {"Vickie"=>"Excluded", "jsong6"=>"Excluded"} | RAYYAN-EXCLUSION-REASONS: irrelevant
rayyan-283776045	The antipruritic effects of chlorpheniramine, cyproheptadine and sulphapyridine monitored with limb activity meters on chloroquine induced pruritus among patients with malaria	1995	24	1	67â€“73		Osifo, NG	RAYYAN-INCLUSION: {"Vickie"=>"Excluded", "jsong6"=>"Excluded"} | RAYYAN-EXCLUSION-REASONS: acute,irrelevant
rayyan-283776046	Improved detection of the pulse oximeter signal with a digital nerve block in patients in poor health status	2008	55	8	481â€“486		CordovÃ- de Armas, L and Espinaco ValdÃ©s, J and JimÃ©nez Paneque, RE and Costa Hidalgo, T and Vallongo MenÃ©ndez, MB	RAYYAN-INCLUSION: {"Vickie"=>"Excluded", "jsong6"=>"Excluded"} | RAYYAN-EXCLUSION-REASONS: irrelevant
rayyan-283776047	Comparative prospective randomized efficacy testing of different guiding catheters for coronary sinus cannulation in heart failure patients	2003	9	3	343â€“351		Butter, C and Gras, D and Ritter, P and Stellbrink, C and Fleck, E and Tockman, B and Schubert, B and Pochet, T and deVoogt, W	RAYYAN-INCLUSION: {"Vickie"=>"Excluded", "jsong6"=>"Excluded"} | RAYYAN-EXCLUSION-REASONS: irrelevant
rayyan-283776048	Frequency dependent effects of rTMS on motor and cognitive functions in Huntington's disease	2012	2	1	41â€“48		Groiss, SJ and Netz, J and Lange, HW and Buetefisch, CM	RAYYAN-INCLUSION: {"Vickie"=>"Excluded", "jsong6"=>"Excluded"} | RAYYAN-EXCLUSION-REASONS: irrelevant
rayyan-283776049	Somatosensory and transcranial direct current stimulation effects on manual dexterity and motor cortex function: A metaplasticity study	2019	12	4	938â€“947		Trudgen, A and Cirillo, J and Byblow, WD	RAYYAN-INCLUSION: {"Vickie"=>"Excluded", "jsong6"=>"Excluded"} | RAYYAN-EXCLUSION-REASONS: irrelevant
rayyan-283776050	A Randomised Ensemble Learning Approach for Multiclass Motor Imagery Classification Using Error Correcting Output Coding	2018	2018		5081â€“5084		Bera, S and Roy, R and Sikdar, D and Kar, A and Mukhopadhyay, R and Mahadevappal, M	RAYYAN-INCLUSION: {"Vickie"=>"Excluded", "jsong6"=>"Excluded"} | RAYYAN-EXCLUSION-REASONS: irrelevant
rayyan-283776051	Bihemispheric transcranial direct current stimulation enhances effector-independent representations of motor synergy and sequence learning	2014	34	3	1037â€“1050		Waters-Metenier, S and Husain, M and Wiestler, T and Diedrichsen, J	RAYYAN-INCLUSION: {"Vickie"=>"Excluded", "jsong6"=>"Excluded"} | RAYYAN-EXCLUSION-REASONS: irrelevant
rayyan-283776052	The effect of acetylcholine on finger capillary pressure and capillary flow in healthy volunteers	1996	494	1	307â€“313		Morris, SJ and Kunzek, S and Shore, AC	RAYYAN-INCLUSION: {"Vickie"=>"Excluded", "jsong6"=>"Excluded"} | RAYYAN-EXCLUSION-REASONS: irrelevant
rayyan-283776053	Enhanced motor imagery-based BCI performance via tactile stimulation on unilateral hand	2017	11				Shu, X and Yao, L and Sheng, X and Zhang, D and Zhu, X	RAYYAN-INCLUSION: {"Vickie"=>"Excluded", "jsong6"=>"Excluded"} | RAYYAN-EXCLUSION-REASONS: irrelevant
rayyan-283776054	Traditional care of the perineum during birth. A prospective, randomized, multicenter study of 1,076 women	2002	47	6	477â€“482		Mayerhofer, K and Bodner-Adler, B and Bodner, K and Rabl, M and Kaider, A and Wagenbichler, P and Joura, EA and Husslein, P	RAYYAN-INCLUSION: {"Vickie"=>"Excluded", "jsong6"=>"Excluded"} | RAYYAN-EXCLUSION-REASONS: irrelevant
rayyan-283776055	Effect of acupuncture 'dose' on modulation of the default mode network of the brain	2016	34	6	425â€“432		Lin, YJ and Kung, YY and Kuo, WJ and Niddam, DM and Chou, CC and Cheng, CM and Yeh, TC and Hsieh, JC and Chiu, JH	RAYYAN-INCLUSION: {"Vickie"=>"Excluded", "jsong6"=>"Excluded"} | RAYYAN-EXCLUSION-REASONS: irrelevant
rayyan-283776056	Interhemispheric Modulation of Dual-Mode, Noninvasive Brain Stimulation on Motor Function	2014	38	3	297â€“303		Park, E and Kim, YH and Chang, WH and Kwon, TG and Shin, YI	RAYYAN-INCLUSION: {"Vickie"=>"Excluded", "jsong6"=>"Excluded"} | RAYYAN-EXCLUSION-REASONS: acute,irrelevant
rayyan-283776057	Enhancement of motor learning with transcranial direct current stimulation in healthy children	2015	78		S157â€“S158		Ciechanski, P and Zewdie, E and Kirton, A	RAYYAN-INCLUSION: {"Vickie"=>"Excluded", "jsong6"=>"Excluded"} | RAYYAN-EXCLUSION-REASONS: irrelevant
rayyan-283776058	Effectiveness of transcranial direct current stimulation and mirror illusion on motor learning	2012	26	4	425â€•		Matsuo, A and Sahara, S and Nakahara, S and Hatta, Y and Hiyamizu, M and Maeoka, H and Morioka, S	RAYYAN-INCLUSION: {"Vickie"=>"Excluded", "jsong6"=>"Excluded"} | RAYYAN-EXCLUSION-REASONS: irrelevant
rayyan-283776059	Astigmatic change in biaxial microincisional cataract surgery with enlargement of one incision: a prospective controlled study	2009	37	3	254â€“261		Kaufmann, C and Thiel, MA and Esterman, A and Dougherty, PJ and Goggin, M	RAYYAN-INCLUSION: {"Vickie"=>"Excluded", "jsong6"=>"Excluded"} | RAYYAN-EXCLUSION-REASONS: irrelevant
rayyan-283776060	Effects of transcranial direct current stimulation on GABA and Glx in children: A pilot study	2020	15	1			Nwaroh, C and Giuffre, A and Cole, L and Bell, T and Carlson, HL and MacMaster, FP and Kirton, A and Harris, AD	RAYYAN-INCLUSION: {"Vickie"=>"Excluded", "jsong6"=>"Excluded"} | RAYYAN-EXCLUSION-REASONS: irrelevant
rayyan-283776061	Auriculotherapy in the treatment of Anxiety in people with HIV	2021					RBR-2jwy9xf,	RAYYAN-INCLUSION: {"Vickie"=>"Excluded", "jsong6"=>"Excluded"} | RAYYAN-EXCLUSION-REASONS: irrelevant
rayyan-283776062	Verbal dichotic listening in developmental stuttering: subgroups with atypical auditory processing	2004	17	4	224â€“232		Foundas, AL and Corey, DM and Hurley, MM and Heilman, KM	RAYYAN-INCLUSION: {"Vickie"=>"Excluded", "jsong6"=>"Excluded"} | RAYYAN-EXCLUSION-REASONS: irrelevant
rayyan-283776063	The efficacy of '0.05% Clobetasol + 2.5% zinc sulphate' cream vs. '0.05% Clobetasol alone' cream in the treatment of the chronic hand eczema: a double-blind study	2008	22	5	531â€“536		Faghihi, G and Iraji, F and Shahingohar, A and Saidat, A	RAYYAN-INCLUSION: {"Vickie"=>"Excluded", "jsong6"=>"Excluded"} | RAYYAN-EXCLUSION-REASONS: irrelevant
rayyan-283776065	Effect of physical activity on postpartum health	2021					IRCT20170501033743N3,	RAYYAN-INCLUSION: {"Vickie"=>"Excluded", "jsong6"=>"Excluded"} | RAYYAN-EXCLUSION-REASONS: irrelevant
rayyan-283776066	REUSABLE LARYNGEAL TUBE SUCTION(LTS-II); STANDARD INSERTION TECHNIQUE VERSUS TWO MODIFIED INSERTION TECHNIQUES FOR PATIENTS WITH A SIMULATED DIFFICULT AIRWAY	2016					CTRI/2016/08/007127,	RAYYAN-INCLUSION: {"Vickie"=>"Excluded", "jsong6"=>"Excluded"} | RAYYAN-EXCLUSION-REASONS: irrelevant
rayyan-283776067	The effect of counseling and foot reflexology on fatigue in women in the first pregnancy	2015					IRCT201508156888N11,	RAYYAN-INCLUSION: {"Vickie"=>"Excluded", "jsong6"=>"Excluded"} | RAYYAN-EXCLUSION-REASONS: irrelevant
rayyan-283776068	Effect of neuromuscular and Jones techniques on patients with bruxism and associated episodic and chronic tension headaches	2019					ACTRN12619001323112,	RAYYAN-INCLUSION: {"Vickie"=>"Excluded", "jsong6"=>"Excluded"} | RAYYAN-EXCLUSION-REASONS: irrelevant
rayyan-283776069	Prospective comparison between reduced port laparoscopic anterior resection and usual 5 port one for s. colon cancer and rectal cancer	2013	27		S313		Hattori, K and Ohta, K	RAYYAN-INCLUSION: {"Vickie"=>"Excluded", "jsong6"=>"Excluded"} | RAYYAN-EXCLUSION-REASONS: irrelevant
rayyan-283776070	Training and reaching in infants	2012					RBR-35rp8x,	RAYYAN-INCLUSION: {"Vickie"=>"Excluded", "jsong6"=>"Excluded"} | RAYYAN-EXCLUSION-REASONS: irrelevant
rayyan-283776071	Comparison of "Pick'n Roll" Single-incision Laparoscopic Cholecystectomy Technique, and Laparoscopic Cholecystectomy	2014					NCT02149797,	RAYYAN-INCLUSION: {"Vickie"=>"Excluded", "jsong6"=>"Excluded"} | RAYYAN-EXCLUSION-REASONS: irrelevant
rayyan-283776072	The effects of relaxation technique on infertility stress	2014					IRCT2014021516581N1,	RAYYAN-INCLUSION: {"Vickie"=>"Excluded", "jsong6"=>"Excluded"} | RAYYAN-EXCLUSION-REASONS: irrelevant
rayyan-283776075	Application of an advanced noise reduction algorithm for imaging of hands in rheumatic diseases-evaluation of image quality compared to standard dose images	2019	78		1012â€•		Ziegeler, K and Siepmann, S and Beck, A and Lembcke, A and Hamm, B and Hermann, KGA	RAYYAN-INCLUSION: {"Vickie"=>"Excluded", "jsong6"=>"Excluded"} | RAYYAN-EXCLUSION-REASONS: irrelevant
rayyan-283776077	Free Fatty Acids, Body Weight, and Growth Hormones Secretion in Children	2010					NCT01237041,	RAYYAN-INCLUSION: {"Vickie"=>"Excluded", "jsong6"=>"Excluded"} | RAYYAN-EXCLUSION-REASONS: irrelevant
rayyan-283776078	Comprehensive lifestyle change program in elderly patients with heart failure	2018	7		94â€•		Alhama-Belotto, M and Gray, J and Suhar, C	RAYYAN-INCLUSION: {"Vickie"=>"Excluded", "jsong6"=>"Excluded"} | RAYYAN-EXCLUSION-REASONS: irrelevant
rayyan-283776079	Construction and clinical preliminary validation of an automaticbone age assessment model based on deep learning	2019	53	11	974â€“978		Song, J and Gong, P and Gao, C and Han, Q and Li, X and Zhu, Z and Chen, H and Yu, Y and Fang, X	RAYYAN-INCLUSION: {"Vickie"=>"Excluded", "jsong6"=>"Excluded"} | RAYYAN-EXCLUSION-REASONS: irrelevant
rayyan-283776080	The mechanism of a human reaction to vibration stress by palmar sweating in relation to autonomic nerve tone	2000	73	1	41â€“46		Ando, H and Ishitake, T and Miyazaki, Y and Kano, M and Tsutsumi, A and Matoba, T	RAYYAN-INCLUSION: {"Vickie"=>"Excluded", "jsong6"=>"Excluded"} | RAYYAN-EXCLUSION-REASONS: irrelevant
rayyan-283776081	Interest of a three-dimensional vision system in laparoscopic suturing on pelvi-trainer: a prospective comparative study among naive medical students	2014	21	6	S90		Rabischong, B and Compan, C and Botchorishvili, R and Bourdel, N and Canis, M	RAYYAN-INCLUSION: {"Vickie"=>"Excluded", "jsong6"=>"Excluded"} | RAYYAN-EXCLUSION-REASONS: irrelevant
rayyan-283776082	Factors responsible for fistula failure in hemodialysis patients	2006	22	4	451â€“453		Talaiezadeh, AH and Askarpour, S and Paziar, F	RAYYAN-INCLUSION: {"Vickie"=>"Excluded", "jsong6"=>"Excluded"} | RAYYAN-EXCLUSION-REASONS: irrelevant
rayyan-283776084	On the acute effect of some drugs on the heart rate in man during the establishment of artificial conditioned speech connections. I	1974	68		45â€“58		Hrbek, J and Komenda, S and Siroka, A and Macakova, J	RAYYAN-INCLUSION: {"Vickie"=>"Excluded", "jsong6"=>"Excluded"} | RAYYAN-EXCLUSION-REASONS: acute,irrelevant
rayyan-283776085	A double-blind randomized placebo controlled parallel group study evaluating the effects of ibuprofen and glucosamine sulfate on exercise induced muscle soreness	2007	15	1	21â€“28		Arendt-Nielsen, L and Weidner, M and Bartholin, D and Rosetzsky, A	RAYYAN-INCLUSION: {"Vickie"=>"Excluded", "jsong6"=>"Excluded"} | RAYYAN-EXCLUSION-REASONS: irrelevant
rayyan-283776086	Effect of tactile stimulation on hand mental rotation among young healthy adults: a randomized controlled trial	2020	7	2			Rezaeinasab, M and Estahbanati, MF and Chermahini, SA and Shamsizadeh, A and Assadollahi, Z and Hasanshahi, A and Azin, M	RAYYAN-INCLUSION: {"Vickie"=>"Excluded", "jsong6"=>"Excluded"} | RAYYAN-EXCLUSION-REASONS: irrelevant
rayyan-283776087	Anodal transcranial direct current stimulation over the posterior parietal cortex reduces the onset time to the rubber hand illusion and increases the body ownership	2018					Lira, M and Pantaleao, FN and de Souza Ramos, CG and Boggio, PS	RAYYAN-INCLUSION: {"Vickie"=>"Excluded", "jsong6"=>"Excluded"} | RAYYAN-EXCLUSION-REASONS: irrelevant
rayyan-283776088	Glove perforation rate in vascular surgery--a comparison between single and double gloving	2001	30	2	122â€“124		Aarnio, P and Laine, T	RAYYAN-INCLUSION: {"Vickie"=>"Excluded", "jsong6"=>"Excluded"} | RAYYAN-EXCLUSION-REASONS: irrelevant
rayyan-283776089	Writer's cramp: a single-center experience	2021	190		S11â€•		Cakar, A and Tufekcioglu, Z and Hanagasi, H and Parman, Y	RAYYAN-INCLUSION: {"Vickie"=>"Excluded", "jsong6"=>"Excluded"} | RAYYAN-EXCLUSION-REASONS: irrelevant
rayyan-283776090	A double-blind randomized placebo controlled parallel group study demonstrates analgesic effects of sheanut oil extract in exercise induced muscle tenderness	2009	17	1	8â€“14		Arendt-Nielsen, L and Rosetzsky, A and Weidner, MS	RAYYAN-INCLUSION: {"Vickie"=>"Excluded", "jsong6"=>"Excluded"} | RAYYAN-EXCLUSION-REASONS: irrelevant
rayyan-283776091	Multimodal Assessment of Precentral Anodal TDCS: individual Rise in Supplementary Motor Activity Scales With Increase in Corticospinal Excitability	2021	15				Karabanov, AN and Shindo, K and Shindo, Y and Raffin, E and Siebner, HR	RAYYAN-INCLUSION: {"Vickie"=>"Excluded", "jsong6"=>"Excluded"} | RAYYAN-EXCLUSION-REASONS: irrelevant
rayyan-283776092	Enhancing mirror visual feedback with intermittent theta burst stimulation in healthy adults	2019					Jiaqi Zhang, J and Fong, KNK	RAYYAN-INCLUSION: {"Vickie"=>"Excluded", "jsong6"=>"Excluded"} | RAYYAN-EXCLUSION-REASONS: irrelevant
rayyan-283776093	Some possible causes of associations between HL A antigens and disease	1975	2	5	485â€“489		Boettcher, B	RAYYAN-INCLUSION: {"Vickie"=>"Excluded", "jsong6"=>"Excluded"} | RAYYAN-EXCLUSION-REASONS: irrelevant
rayyan-283776094	MI Practice and tDCS With Aging	2016					NCT02813291,	RAYYAN-INCLUSION: {"Vickie"=>"Excluded", "jsong6"=>"Excluded"} | RAYYAN-EXCLUSION-REASONS: irrelevant
rayyan-283776095	Representation of wrist joint kinematics by the ensemble of muscle spindles from synergistic muscles	1998	79	5	2265â€“2276		Verschueren, SM and Cordo, PJ and Swinnen, SP	RAYYAN-INCLUSION: {"Vickie"=>"Excluded", "jsong6"=>"Excluded"} | RAYYAN-EXCLUSION-REASONS: irrelevant
rayyan-283776096	A retrospective analysis of hand tapping as a longitudinal marker of disease progression in Huntington's disease	2014	14			35	Collins, LM and Lazic, SE and Barker, RA	RAYYAN-INCLUSION: {"Vickie"=>"Excluded", "jsong6"=>"Excluded"} | RAYYAN-EXCLUSION-REASONS: irrelevant
rayyan-283776097	Reactive rise in blood pressure upon cuff inflation: cuff inflation at the arm causes a greater rise in pressure than at the wrist in hypertensive patients	2007	12	5	275â€“280		Charmoy, A and WÃ¼rzner, G and Ruffieux, C and Hasler, C and Cachat, F and Waeber, B and Burnier, M	RAYYAN-INCLUSION: {"Vickie"=>"Excluded", "jsong6"=>"Excluded"} | RAYYAN-EXCLUSION-REASONS: irrelevant
rayyan-283776098	Mode and site of acupuncture modulation in the human brain: 3D (124-ch) EEG power spectrum mapping and source imaging	2006	29	4	1080â€“1091		Chen, AC and Liu, FJ and Wang, L and Arendt-Nielsen, L	RAYYAN-INCLUSION: {"Vickie"=>"Excluded", "jsong6"=>"Excluded"} | RAYYAN-EXCLUSION-REASONS: irrelevant
rayyan-283776099	The effects of venlafaxine on cortical motor area activity in healthy subjects: a pilot study	2014	34	1	93â€“98		Li, CY and Song, XZ and Han, LX and Xie, Q and Wang, J and Li, YK and Liu, FD and Liu, Y	RAYYAN-INCLUSION: {"Vickie"=>"Excluded", "jsong6"=>"Excluded"} | RAYYAN-EXCLUSION-REASONS: irrelevant
rayyan-283776100	Mapping proprioception across a 2D horizontal workspace	2010	5	7	e11851		Wilson, ET and Wong, J and Gribble, PL	RAYYAN-INCLUSION: {"Vickie"=>"Excluded", "jsong6"=>"Excluded"} | RAYYAN-EXCLUSION-REASONS: irrelevant
rayyan-283776101	Effect of modified jaw thrust maneuver on tracheal intubation using a lightwand	2015				45	Hwang, J-Y and Chang, J-E and Kim, H and Kim, C-S and Min, S-W	RAYYAN-INCLUSION: {"Vickie"=>"Excluded", "jsong6"=>"Excluded"} | RAYYAN-EXCLUSION-REASONS: irrelevant
rayyan-283776102	Enhancement of motor learning by focal intermittent theta burst stimulation (iTBS) of either the primary motor (M1) or somatosensory area (S1) in healthy human subjects	2018	36	1	117â€“130		Platz, T and Adler-Wiebe, M and Roschka, S and Lotze, M	RAYYAN-INCLUSION: {"Vickie"=>"Excluded", "jsong6"=>"Excluded"} | RAYYAN-EXCLUSION-REASONS: irrelevant
rayyan-283776103	Cortical representation of different motor rhythms during bimanual movements	2012	223	4	489â€“504		Muthuraman, M and Arning, K and Govindan, RB and Heute, U and Deuschl, G and Raethjen, J	RAYYAN-INCLUSION: {"Vickie"=>"Excluded", "jsong6"=>"Excluded"} | RAYYAN-EXCLUSION-REASONS: irrelevant
rayyan-283776104	Acute effects of vibration on digital circulatory function in healthy men	1995	52	12	834â€“841		Bovenzi, M and Griffin, MJ and Ruffell, CM	RAYYAN-INCLUSION: {"Vickie"=>"Excluded", "jsong6"=>"Excluded"} | RAYYAN-EXCLUSION-REASONS: acute,irrelevant
rayyan-283776105	Is performance of intermittent intense exercise enhanced by use of a commercial palm cooling device?	2009	23	9	2666â€“2672		Walker, TB and Zupan, MF and McGregor, JN and Cantwell, AR and Norris, TD	RAYYAN-INCLUSION: {"Vickie"=>"Excluded", "jsong6"=>"Excluded"} | RAYYAN-EXCLUSION-REASONS: irrelevant
rayyan-283776106	Reaching to proprioceptively defined targets in Parkinson's disease: effects of deep brain stimulation therapy	2013	244		99â€“112		Lee, D and Henriques, DY and Snider, J and Song, D and Poizner, H	RAYYAN-INCLUSION: {"Vickie"=>"Excluded", "jsong6"=>"Excluded"} | RAYYAN-EXCLUSION-REASONS: irrelevant
rayyan-283776107	The effects of immediate vision on implicit hand maps	2014	232	4	1241â€“1247		Longo, MR	RAYYAN-INCLUSION: {"Vickie"=>"Excluded", "jsong6"=>"Excluded"} | RAYYAN-EXCLUSION-REASONS: irrelevant
rayyan-283776108	Videolaryngoscopic endotracheal intubation (GlideScope) of morbidly obese patients in semi-erect position: a comparison with rapid sequence induction in supine position	2012	21	6	843â€“850		Gupta, D and Rusin, K	RAYYAN-INCLUSION: {"Vickie"=>"Excluded", "jsong6"=>"Excluded"} | RAYYAN-EXCLUSION-REASONS: irrelevant
rayyan-283776109	Psychomotor performance in relation to acute oral administration of Delta9-tetrahydrocannabinol and standardized cannabis extract in healthy human subjects	2009	259	5	284â€“292		Roser, P and Gallinat, J and Weinberg, G and Juckel, G and Gorynia, I and Stadelmann, AM	RAYYAN-INCLUSION: {"Vickie"=>"Excluded", "jsong6"=>"Excluded"} | RAYYAN-EXCLUSION-REASONS: acute,irrelevant
rayyan-283776110	Increasing response rates to postal questionnaires: a randomised trial of variations in design	2004	9	4	213â€“217		Puffer, S and Porthouse, J and Birks, Y and Morton, V and Torgerson, D	RAYYAN-INCLUSION: {"Vickie"=>"Excluded", "jsong6"=>"Excluded"} | RAYYAN-EXCLUSION-REASONS: irrelevant
rayyan-283776111	Decline in measured glomerular filtration rate is associated with a decrease in endurance, strength, balance and fine motor skills	2017	22	7	513â€“519		Hellberg, M and HÃ¶glund, P and Svensson, P and Abdulahi, H and Clyne, N	RAYYAN-INCLUSION: {"Vickie"=>"Excluded", "jsong6"=>"Excluded"} | RAYYAN-EXCLUSION-REASONS: irrelevant
rayyan-283776112	Motor imagery-induced EEG patterns in individuals with spinal cord injury and their impact on brain-computer interface accuracy	2014	11	3		35011	MÃ¼ller-Putz, GR and Daly, I and Kaiser, V	RAYYAN-INCLUSION: {"Vickie"=>"Excluded", "jsong6"=>"Excluded"} | RAYYAN-EXCLUSION-REASONS: irrelevant
rayyan-283776113	Clinical evaluation of a finger oscillometric blood pressure device	1991	25	12	1310â€“1314		Sesler, JM and Munroe, WP and McKenney, JM	RAYYAN-INCLUSION: {"Vickie"=>"Excluded", "jsong6"=>"Excluded"} | RAYYAN-EXCLUSION-REASONS: irrelevant
rayyan-283776114	Effect of Yoga asanas on nerve conduction in type 2 diabetes	2002	46	3	298â€“306		Malhotra, V and Singh, S and Tandon, OP and Madhu, SV and Prasad, A and Sharma, SB	RAYYAN-INCLUSION: {"Vickie"=>"Excluded", "jsong6"=>"Excluded"} | RAYYAN-EXCLUSION-REASONS: irrelevant
rayyan-283776115	Objective assessment of training surgical skills using simulated tissue interface with real-time feedback	2008	65	4	270â€“274		Rafiq, A and Tamariz, F and Boanca, C and Lavrentyev, V and Merrell, RC	RAYYAN-INCLUSION: {"Vickie"=>"Excluded", "jsong6"=>"Excluded"} | RAYYAN-EXCLUSION-REASONS: irrelevant
rayyan-283776116	The influence of Emla cream on cutaneous microcirculation	2003	28	3	121â€“128		HÃ¤fner, HM and Thomma, SR and Eichner, M and Steins, A and JÃ¼nger, M	RAYYAN-INCLUSION: {"Vickie"=>"Excluded", "jsong6"=>"Excluded"} | RAYYAN-EXCLUSION-REASONS: irrelevant
rayyan-283776117	Treatment of the alcoholic organic brain syndrome with EMD 21657. A derivative of a pyritinolmetabolite: double-blind clinical, quantitative EEG and psychometric studies	1978	13	3	177â€“192		Saletu, B and GrÃ¼nberger, J and Saletu, M and Mader, R and Volavka, J	RAYYAN-INCLUSION: {"Vickie"=>"Excluded", "jsong6"=>"Excluded"} | RAYYAN-EXCLUSION-REASONS: irrelevant
rayyan-283776118	Combining fMRI with a pharmacokinetic model to determine which brain areas activated by painful stimulation are specifically modulated by remifentanil	2002	16	4	999â€“1014		Wise, RG and Rogers, R and Painter, D and Bantick, S and Ploghaus, A and Williams, P and Rapeport, G and Tracey, I	RAYYAN-INCLUSION: {"Vickie"=>"Excluded", "jsong6"=>"Excluded"} | RAYYAN-EXCLUSION-REASONS: irrelevant
rayyan-283776119	Biofield detection: role of bioenergy awareness training and individual differences in absorption	2004	10	1	167â€“169		Schwartz, GE and Swanick, S and Sibert, W and Lewis, DA and Lewis, SE and Nelson, L and Jain, S and Mallory, L and Foust, L and Moore, K and Tussing, D and Bell, IR	RAYYAN-INCLUSION: {"Vickie"=>"Excluded", "jsong6"=>"Excluded"} | RAYYAN-EXCLUSION-REASONS: irrelevant
rayyan-283776120	Comparison of the effect of reflexology and body massage on physiological parameters and bilirubin levels in neonates under photo therapy	2020					IRCT20161210031328N11,	RAYYAN-INCLUSION: {"Vickie"=>"Excluded", "jsong6"=>"Excluded"} | RAYYAN-EXCLUSION-REASONS: irrelevant
rayyan-283776121	The Milch vs. the double traction method for shoulder relocation	2018					IRCT20131226015941N5,	RAYYAN-INCLUSION: {"Vickie"=>"Excluded", "jsong6"=>"Excluded"} | RAYYAN-EXCLUSION-REASONS: irrelevant
rayyan-283776122	The effect of accupressure on anxiety and pain of dressing in patient with burns	2019					IRCT20180902040926N1,	RAYYAN-INCLUSION: {"Vickie"=>"Excluded", "jsong6"=>"Excluded"} | RAYYAN-EXCLUSION-REASONS: irrelevant
rayyan-283776123	Long-term progressive motor skill training enhances corticospinal excitability for the ipsilateral hemisphere and motor performance of the untrained hand	2017	45	12	1490â€“1500		Christiansen, L and Larsen, MN and Grey, MJ and Nielsen, JB and Lundbye-Jensen, J	RAYYAN-INCLUSION: {"Vickie"=>"Excluded", "jsong6"=>"Excluded"} | RAYYAN-EXCLUSION-REASONS: irrelevant,no right and left limb training
rayyan-283776124	The influence of antihypertensive therapy on the structural arteriolar changes in essential hypertension: different effects of enalapril and hydrochlorothiazide	1993	234	3	271â€“279		DahlÃ¶f, B and Hansson, L	RAYYAN-INCLUSION: {"Vickie"=>"Excluded", "jsong6"=>"Excluded"} | RAYYAN-EXCLUSION-REASONS: irrelevant
rayyan-283776125	The acquisition of bimanual coordination is mediated by anisotropic coupling between the hands	2002	21	5	699â€“721		Summers, JJ and Davis, AS and Byblow, WD	RAYYAN-INCLUSION: {"Vickie"=>"Excluded", "jsong6"=>"Excluded"} | RAYYAN-EXCLUSION-REASONS: irrelevant
rayyan-283776126	Resource allocation and somatosensory P300 amplitude during dual task: effects of tracking speed and predictability of tracking direction	2004	115	11	2616â€“2628		Kida, T and Nishihira, Y and Hatta, A and Wasaka, T and Tazoe, T and Sakajiri, Y and Nakata, H and Kaneda, T and Kuroiwa, K and Akiyama, S and Sakamoto, M and Kamijo, K and Higashiura, T	RAYYAN-INCLUSION: {"Vickie"=>"Excluded", "jsong6"=>"Excluded"} | RAYYAN-EXCLUSION-REASONS: irrelevant
rayyan-283776127	Effects of training with a dynamic moment of inertia bat on swing performance	2011	25	11	2999â€“3005		Liu, C and Liu, YC and Kao, YC and Shiang, TY	RAYYAN-INCLUSION: {"Vickie"=>"Excluded", "jsong6"=>"Excluded"} | RAYYAN-EXCLUSION-REASONS: irrelevant
rayyan-283776128	Rapid opening and closing of the hand as a measure of early neurologic recovery in the upper extremity after surgery for cervical spondylotic myelopathy	2005	86	1	105â€“108		Prabhu, K and Babu, KS and Samuel, S and Chacko, AG	RAYYAN-INCLUSION: {"Vickie"=>"Excluded", "jsong6"=>"Excluded"} | RAYYAN-EXCLUSION-REASONS: irrelevant
rayyan-283776129	A comparison of single-handed chin lift and two-handed jaw thrust for tracheal intubation using a lightwand	2017	31	1	5â€“10		Yang, SM and Kim, H and Chang, JE and Min, SW and Lee, JM and Hwang, JY	RAYYAN-INCLUSION: {"Vickie"=>"Excluded", "jsong6"=>"Excluded"} | RAYYAN-EXCLUSION-REASONS: irrelevant
rayyan-283776130	Comparison of nail lacquer clobetasol efficacy at 0.05%, 1% and 8% in nail psoriasis treatment: prospective, controlled and randomized pilot study	2012	87	2	203â€“211		Nakamura, RC and Abreu, Ld and Duque-Estrada, B and Tamler, C and Leverone, AP	RAYYAN-INCLUSION: {"Vickie"=>"Excluded", "jsong6"=>"Excluded"} | RAYYAN-EXCLUSION-REASONS: irrelevant
rayyan-283776131	Palmar Warming for Radial Artery Vasodilation to Facilitate Transradial Access: a Randomized Controlled Trial	2019	30	3	421â€“424		Al-Hakim, R and Hedge, JC and Jahangiri, Y and Kaufman, JA and Galuppo, R and Farsad, K	RAYYAN-INCLUSION: {"Vickie"=>"Excluded", "jsong6"=>"Excluded"} | RAYYAN-EXCLUSION-REASONS: irrelevant
rayyan-283776132	Conditioned pain modulation dampens the thermal grill illusion	2017	21	9	1591â€“1601		Harper, DE and Hollins, M	RAYYAN-INCLUSION: {"Vickie"=>"Excluded", "jsong6"=>"Excluded"} | RAYYAN-EXCLUSION-REASONS: irrelevant
rayyan-283776133	Intramuscular clodronate in erosive osteoarthritis of the hand is effective on pain and reduces serum COMP: a randomized pilot trial-The ER.O.D.E. study (ERosive Osteoarthritis and Disodium-clodronate Evaluation)	2017	36	10	2343â€“2350		Saviola, G and Abdi-Ali, L and Povino, MR and Campostrini, L and Sacco, S and Dalle Carbonare, null and Carbonare, LD	RAYYAN-INCLUSION: {"Vickie"=>"Excluded", "jsong6"=>"Excluded"} | RAYYAN-EXCLUSION-REASONS: irrelevant
rayyan-283776135	The clinical benefit of topical moisturizers in xerosis patient	2015					KCT0001508,	RAYYAN-INCLUSION: {"Vickie"=>"Excluded", "jsong6"=>"Excluded"} | RAYYAN-EXCLUSION-REASONS: irrelevant
rayyan-283776136	Test models for hygienic handrub and hygienic handwash: the effects of two different contamination and sampling techniques	1992	20	3	163â€“171		Rotter, ML and Koller, W	RAYYAN-INCLUSION: {"Vickie"=>"Excluded", "jsong6"=>"Excluded"} | RAYYAN-EXCLUSION-REASONS: irrelevant
rayyan-283776137	Randomized trial of Nordic walking in patients with moderate to severe heart failure	2013	29	11	1470â€“1476		Keast, ML and Slovinec D'Angelo, ME and Nelson, CR and Turcotte, SE and McDonnell, LA and Nadler, RE and Reed, JL and Pipe, AL and Reid, RD	RAYYAN-INCLUSION: {"Vickie"=>"Excluded", "jsong6"=>"Excluded"} | RAYYAN-EXCLUSION-REASONS: irrelevant

rayyan-283776138	Spinal anesthesia in cesarean section: 1% versus 0.5% hyperbaric bupivacaine	1996	62	1	9â€“15		Gallo, F and Alberti, A and Fongaro, A and Negri, MG and Carlot, A and Altafini, L and Valenti, S	RAYYAN-INCLUSION: {"Vickie"=>"Excluded", "jsong6"=>"Excluded"} | RAYYAN-EXCLUSION-REASONS: irrelevant
rayyan-283776139	Daytime sleep has no effect on the time course of motor sequence and visuomotor adaptation learning	2016	131		147â€“154		Backhaus, W and Braass, H and Renne, T and Kruger, C and Gerloff, C and Hummel, FC	RAYYAN-INCLUSION: {"Vickie"=>"Excluded", "jsong6"=>"Excluded"} | RAYYAN-EXCLUSION-REASONS: irrelevant
rayyan-283776140	Efficacy of Hand Exoskeleton Controlled by BCI in Post Stroke Patients	2014					NCT02325947,	RAYYAN-INCLUSION: {"Vickie"=>"Excluded", "jsong6"=>"Excluded"} | RAYYAN-EXCLUSION-REASONS: irrelevant
rayyan-283776141	The effect of propranolol on post-traumatic stress and clinical outcomes in burn patients	2020					IRCT20190919044819N2,	RAYYAN-INCLUSION: {"Vickie"=>"Excluded", "jsong6"=>"Excluded"} | RAYYAN-EXCLUSION-REASONS: irrelevant
rayyan-283776142	Sensory Stimulation Training for BCI System Based on Somatosensory Attentional Orientation	2019	66	3	640â€“646		Yao, L and Sheng, X and Mrachacz-Kersting, N and Zhu, X and Farina, D and Jiang, N	RAYYAN-INCLUSION: {"Vickie"=>"Excluded", "jsong6"=>"Excluded"} | RAYYAN-EXCLUSION-REASONS: irrelevant
rayyan-283776143	Study of Spirit Pass in Newborn and Patient	2015					NCT02489136,	RAYYAN-INCLUSION: {"Vickie"=>"Excluded", "jsong6"=>"Excluded"} | RAYYAN-EXCLUSION-REASONS: irrelevant
rayyan-283776144	Music combined touch therapy to decrease pain response in preterm infants	2016					ISRCTN14131492,	RAYYAN-INCLUSION: {"Vickie"=>"Excluded", "jsong6"=>"Excluded"} | RAYYAN-EXCLUSION-REASONS: irrelevant
rayyan-283776145	Control of Pain From Braces With Patient Advice Sheets	2017					NCT03159520,	RAYYAN-INCLUSION: {"Vickie"=>"Excluded", "jsong6"=>"Excluded"} | RAYYAN-EXCLUSION-REASONS: irrelevant
rayyan-283776146	Motivation to learn: can incentives improve laparoscopic training?	2017	24	7	S194â€“S195		Abittan, BS and Grant, A and Stauber, M and Ricardo, A and Nimaroff, M	RAYYAN-INCLUSION: {"Vickie"=>"Excluded", "jsong6"=>"Excluded"} | RAYYAN-EXCLUSION-REASONS: irrelevant
rayyan-283776147	Effect of paracetamol pretreatment on rocuronium-induced injection pain: a randomized, double-blind, placebo-controlled comparison with lidocaine	2014	5	6			Ates, G and Kose, EA and Oz, G and Apan, A	RAYYAN-INCLUSION: {"Vickie"=>"Excluded", "jsong6"=>"Excluded"} | RAYYAN-EXCLUSION-REASONS: irrelevant
rayyan-283776148	Randomized clinical trial of the influence of intraperitoneal local anesthesia on pain after laparoscopic colectomy	2009					ACTRN12609000948291,	RAYYAN-INCLUSION: {"Vickie"=>"Excluded", "jsong6"=>"Excluded"} | RAYYAN-EXCLUSION-REASONS: irrelevant
rayyan-283776149	Influence of swaddling on tactile manual learning in preterm infants	2021	153				Lejeune, F and Delacroix, E and Gentaz, E and Berne-Audeoud, F and Marcus, L and Debillon, T	RAYYAN-INCLUSION: {"Vickie"=>"Excluded", "jsong6"=>"Excluded"} | RAYYAN-EXCLUSION-REASONS: irrelevant
rayyan-283776150	Abdominal Compression Administered Early by the Colonoscopist During Water Exchange Colonoscopy	2019					NCT03954561,	RAYYAN-INCLUSION: {"Vickie"=>"Excluded", "jsong6"=>"Excluded"} | RAYYAN-EXCLUSION-REASONS: irrelevant
rayyan-283776152	A Smartphone Application as an Exploratory Endpoint in a Phase 3 Parkinson's Disease Clinical Trial: A Pilot Study	2022					Page, A and Yung, N and Auinger, P and Venuto, C and Glidden, A and Macklin, E and Omberg, L and Schwarzschild, MA and Dorsey, ER	RAYYAN-INCLUSION: {"Vickie"=>"Excluded", "jsong6"=>"Excluded"} | RAYYAN-EXCLUSION-REASONS: irrelevant
rayyan-283776153	Reducing arm muscle activation by using an ablation catheter torque tool: Electromyographic analysis and user perception	2021	144				Mass, P and Kumthekar, R and Opfermann, J and Sherwin, ED and Berul, CI	RAYYAN-INCLUSION: {"Vickie"=>"Excluded", "jsong6"=>"Excluded"} | RAYYAN-EXCLUSION-REASONS: irrelevant
rayyan-283776154	Pain on Injection of Propofol: the Effect of Injectate Temperature	1996	30	4	456â€“460		Lee, SK and Lee, SJ and Kil, HY and Yoon, YJ	RAYYAN-INCLUSION: {"Vickie"=>"Excluded", "jsong6"=>"Excluded"} | RAYYAN-EXCLUSION-REASONS: irrelevant
rayyan-283776155	To compare the current practice of assistance provided by other doctors and paramedics to anaesthesia doctors during visualisation of opening of breathing pipe under general anaesthesia - a randomised , open label.	2017					CTRI/2017/12/010806,	RAYYAN-INCLUSION: {"Vickie"=>"Excluded", "jsong6"=>"Excluded"} | RAYYAN-EXCLUSION-REASONS: irrelevant
rayyan-283776156	Dynamic organization of the somatosensory cortex induced by motor activity	2001	124		2259â€“2267		Braun, C and Heinz, U and Schweizer, R and Wiech, K and Birbaumer, N and Topka, H	RAYYAN-INCLUSION: {"Vickie"=>"Excluded", "jsong6"=>"Excluded"} | RAYYAN-EXCLUSION-REASONS: irrelevant
rayyan-283776157	The effect of blindfold, earmuff and quiet time protocol on sleep and physiological indices in CCU patients	2017					IRCT20170130032293N3,	RAYYAN-INCLUSION: {"Vickie"=>"Excluded", "jsong6"=>"Excluded"} | RAYYAN-EXCLUSION-REASONS: irrelevant
rayyan-283776158	A cane reduces loss of balance in patients with peripheral neuropathy: results from a challenging unipedal balance test	1996	77	5	446â€“452		Ashton-Miller, JA and Yeh, MWL and Richardson, JK and Galloway, T	RAYYAN-INCLUSION: {"Vickie"=>"Excluded", "jsong6"=>"Excluded"} | RAYYAN-EXCLUSION-REASONS: irrelevant
rayyan-283776159	Comparison of the analgesic effects of dronabinol and smoked marijuana in daily marijuana smokers	2013	38	10	1984â€“1992		Cooper, ZD and Comer, SD and Haney, M	RAYYAN-INCLUSION: {"Vickie"=>"Excluded", "jsong6"=>"Excluded"} | RAYYAN-EXCLUSION-REASONS: irrelevant
rayyan-283776160	Randomized comparison of long and short vascular sheaths in reduction of the operator radiation exposure during uterine artery embolization	2016	89	1062		20140448	Cao, MQ and Zhang, XB and Zhuang, ZG and Zhou, W and Xu, JR and Zhong, YC	RAYYAN-INCLUSION: {"Vickie"=>"Excluded", "jsong6"=>"Excluded"} | RAYYAN-EXCLUSION-REASONS: irrelevant
rayyan-283776161	Detection of submaximal effort by use of the rapid exchange grip	1989	14	4	742â€“745		Hildreth, DH and Breidenbach, WC and Lister, GD and Hodges, AD	RAYYAN-INCLUSION: {"Vickie"=>"Excluded", "jsong6"=>"Excluded"} | RAYYAN-EXCLUSION-REASONS: irrelevant
rayyan-283776162	effect of different methods of multimedia distraction on pain and anxiety	2019					IRCT20190224042820N1,	RAYYAN-INCLUSION: {"Vickie"=>"Excluded", "jsong6"=>"Excluded"} | RAYYAN-EXCLUSION-REASONS: irrelevant
rayyan-283776163	Cortical motor circuits after piano training in adulthood: neurophysiologic evidence	2016	11	6			Houdayer, E and Cursi, M and Nuara, A and Zanini, S and Gatti, R and Comi, G and Leocani, L	RAYYAN-INCLUSION: {"Vickie"=>"Excluded", "jsong6"=>"Excluded"} | RAYYAN-EXCLUSION-REASONS: irrelevant
rayyan-283776164	Total Time Spent Training Might Be More Important Than Number of Individual Training Sessions While Building Laparoscopic Skills and Improving Performance	2018	25	7	S280â€“		Abittan, BS and Ricardo, A and Grant, A and Nimaroff, M	RAYYAN-INCLUSION: {"Vickie"=>"Excluded", "jsong6"=>"Excluded"} | RAYYAN-EXCLUSION-REASONS: irrelevant
rayyan-283776165	Sumatriptan (5-HT1B/1D-agonist) causes a transient allodynia	2004	24	12	1057â€“1066		Linde, M and Elam, M and Lundblad, L and Olausson, H and DahlÃ¶f, CG	RAYYAN-INCLUSION: {"Vickie"=>"Excluded", "jsong6"=>"Excluded"} | RAYYAN-EXCLUSION-REASONS: acute,irrelevant
rayyan-283776166	Determination of sex using hand dimensions	2016	6	1	23â€“28		Varu, PR and Gajera, CN and Mangal, HM and Modi, PM	RAYYAN-INCLUSION: {"Vickie"=>"Excluded", "jsong6"=>"Excluded"} | RAYYAN-EXCLUSION-REASONS: irrelevant
rayyan-283776167	A clinical evaluation of a glycolic acid hand treatment with retinyl palmitate and an SPF 15 on photoaged hand skin	2000	11	3	165â€“172		Barkovic, SA and Appa, Y and Payonk, G and Pote, JS and Rizer, RL and Stephens, TJ	RAYYAN-INCLUSION: {"Vickie"=>"Excluded", "jsong6"=>"Excluded"} | RAYYAN-EXCLUSION-REASONS: irrelevant
rayyan-283776168	Clinical evaluation of a radio-protective cream for the hands of the pediatric interventional cardiologist	2017	89	4	709â€“716		Subramanian, S and Waller, BR and Winders, N and Bird, LE and Agrawal, V and Zurakowski, D and Kuhls-Gilcrist, A and Khandkar, A and Sathanandam, SK	RAYYAN-INCLUSION: {"Vickie"=>"Excluded", "jsong6"=>"Excluded"} | RAYYAN-EXCLUSION-REASONS: irrelevant
rayyan-283776169	Evaluation of interference of a hand care cream with alcohol-based hand disinfection	2001	49	1	81â€“84		Dharan, S and Hugonnet, S and Sax, H and Pittet, D	RAYYAN-INCLUSION: {"Vickie"=>"Excluded", "jsong6"=>"Excluded"} | RAYYAN-EXCLUSION-REASONS: irrelevant
rayyan-283776170	Successful burst spinal cord stimulation in a CRPS patient with failed conventional stimulation	2013	16	5	e87â€“		Kriek, N and Groeneweg, G and Van Looy, P and Vancamp, T and De Ridder, D and Huygen, F	RAYYAN-INCLUSION: {"Vickie"=>"Excluded", "jsong6"=>"Excluded"} | RAYYAN-EXCLUSION-REASONS: irrelevant
rayyan-283776172	Hemispheric specialization in normal man studied by bilateral measurements of the regional cerebral blood flow. A study with the 133-Xe inhalation technique	1975	98	3	511â€“524		Risberg, J and Halsey, JH and Wills, EL and Wilson, EM	RAYYAN-INCLUSION: {"Vickie"=>"Excluded", "jsong6"=>"Excluded"} | RAYYAN-EXCLUSION-REASONS: irrelevant
rayyan-283776173	Comparison of clinical efficacy of 0.1% intralesional bleomycin solution and 0.05% intralesional bleomycin solution in patients with warts	2021	15	7	1680â€“1684		Bukhsh, K and Ashraf, A and Kalhoro, HBA and Khalid, Z and Chachar, AZK and Pirzado, MS and Ansari, MA	RAYYAN-INCLUSION: {"Vickie"=>"Excluded", "jsong6"=>"Excluded"} | RAYYAN-EXCLUSION-REASONS: irrelevant
rayyan-283776174	Treatment of Palmar Plantar Erythrodysesthesia (PPE) With Topical Sildenafil	2010					NCT01219023,	RAYYAN-INCLUSION: {"Vickie"=>"Excluded", "jsong6"=>"Excluded"} | RAYYAN-EXCLUSION-REASONS: irrelevant
rayyan-283776175	Effects of movement representation techniques on motor learning of thumb-opposition tasks	2020	10	1	12267â€“		Cuenca-Martinez, F and Suso-Marti, L and Leon-Hernandez, JV and La Touche, R	RAYYAN-INCLUSION: {"Vickie"=>"Excluded", "jsong6"=>"Excluded"} | RAYYAN-EXCLUSION-REASONS: irrelevant
rayyan-283776176	Effect of nattokinase on the blood flow improvement in healthy subjects-a randomized, placebo-controlled, double-blind, cross-over study	2018	46	10	1739â€“1748		Watanabe, N and Takaoka, S and Uehara, S and Ohta, M	RAYYAN-INCLUSION: {"Vickie"=>"Excluded", "jsong6"=>"Excluded"} | RAYYAN-EXCLUSION-REASONS: irrelevant
rayyan-283776177	Activation amplitude patterns do not change for back muscles but are altered for abdominal muscles between dominant and non-dominant hands during one-handed lifts	2009	106	1	95â€“104		Butler, HL and Hubley-Kozey, CL and Kozey, JW	RAYYAN-INCLUSION: {"Vickie"=>"Excluded", "jsong6"=>"Excluded"} | RAYYAN-EXCLUSION-REASONS: irrelevant
rayyan-283776178	Intentional switches between bimanual coordination patterns are primarily effectuated by the nondominant hand	2006	10	1	7â€“23		de Poel, HJ and Peper, CL and Beek, PJ	RAYYAN-INCLUSION: {"Vickie"=>"Excluded", "jsong6"=>"Excluded"} | RAYYAN-EXCLUSION-REASONS: irrelevant
rayyan-283776179	Some problems with reaction time as a measure of memory search	1974	4	5	489â€“492		Durham, RL and Sestokas, D	RAYYAN-INCLUSION: {"Vickie"=>"Excluded", "jsong6"=>"Excluded"} | RAYYAN-EXCLUSION-REASONS: irrelevant
rayyan-283776180	Cross transfer effects of conditioning and deconditioning on muscular strength	1975	18	1	9â€“16		Shaver, LG	RAYYAN-INCLUSION: {"Vickie"=>"Excluded", "jsong6"=>"Excluded"} | RAYYAN-EXCLUSION-REASONS: irrelevant,wrong study design
rayyan-283776181	Anodal tDCS applied during strength training enhances motor cortical plasticity	2013	45	9	1721â€“1729		Hendy, AM and Kidgell, DJ	RAYYAN-INCLUSION: {"Vickie"=>"Excluded", "jsong6"=>"Excluded"} | RAYYAN-EXCLUSION-REASONS: irrelevant
rayyan-283776182	Visual Adaptation Study	2015					NCT02380859,	RAYYAN-INCLUSION: {"Vickie"=>"Excluded", "jsong6"=>"Excluded"} | RAYYAN-EXCLUSION-REASONS: irrelevant
rayyan-283776183	Spatial lateralization: the relationship among sex, handedness and familial sinistrality	1983	21	6	683â€“686		Tinkcom, M and Obrzut, JE and Poston, CS	RAYYAN-INCLUSION: {"Vickie"=>"Excluded", "jsong6"=>"Excluded"} | RAYYAN-EXCLUSION-REASONS: irrelevant
rayyan-283776184	Cerebral lateralization and cognitive deficits after congenital hemiparesis	2002	27	5	356â€“362		Kolk, A and Talvik, T	RAYYAN-INCLUSION: {"Vickie"=>"Excluded", "jsong6"=>"Excluded"} | RAYYAN-EXCLUSION-REASONS: irrelevant
rayyan-283776185	An electrophysiological contribution to the study of language lateralization and prognosis of aphasia	2010	33	2	134â€“141		Cobianchi, A and Dall'Armi, V and Giaquinto, S	RAYYAN-INCLUSION: {"Vickie"=>"Excluded", "jsong6"=>"Excluded"} | RAYYAN-EXCLUSION-REASONS: irrelevant
rayyan-283776186	Effects of an 18-week strength training program on low-handicap golfers' performance	2012	26	4	1110â€“1121		Alvarez, M and Sedano, S and Cuadrado, G and Redondo, JC	RAYYAN-INCLUSION: {"Vickie"=>"Excluded", "jsong6"=>"Excluded"} | RAYYAN-EXCLUSION-REASONS: irrelevant
rayyan-283776187	Assessment of a sixteen-week training program on strength, pain, and function in rheumatoid arthritis patients	2009	15	4	165â€“171		Flint-Wagner, HG and Lisse, J and Lohman, TG and Going, SB and Guido, T and Cussler, E and Gates, D and Yocum, DE	RAYYAN-INCLUSION: {"Vickie"=>"Excluded", "jsong6"=>"Excluded"} | RAYYAN-EXCLUSION-REASONS: irrelevant
rayyan-283776189	Left-right motor cortex asymmetry in the pathophysiology of essential tremor	2020	35		S521â€“		Ammann. C and Urso. D and Pagge. C and Dileone. M and Pineda-Pardo. JA and Fernandez-Rodriguez. B and Monie. MHG and Hernandez-Fernandez. F and Manez-Miro. JU and Martinez-Fer	RAYYAN-INCLUSION: {"Vickie"=>"Excluded", "jsong6"=>"Excluded"} | RAYYAN-EXCLUSION-REASONS: irrelevant,wrong study design
rayyan-283776190	Pediatric transcranial static magnetic field stimulation to improve motor learning: the PSTIM trial	2019	12	2	526â€“		Hollis, A and Zewdie, E and Kuo, H and Hilderley, A and Kirton, A	RAYYAN-INCLUSION: {"Vickie"=>"Excluded", "jsong6"=>"Excluded"} | RAYYAN-EXCLUSION-REASONS: irrelevant,wrong study design
rayyan-283776191	Comparison of hemodynamic changes after repetitive transcranial magnetic stimulation over the anatomical hand knob and hand motor hotspot: a functional near-infrared spectroscopy study	2020	38	6	407â€“417		Kim, J and Kim, H and Lee, J and Lee, HJ and Na, Y and Chang, WH and Kim, YH	RAYYAN-INCLUSION: {"Vickie"=>"Excluded", "jsong6"=>"Excluded"} | RAYYAN-EXCLUSION-REASONS: irrelevant
rayyan-283776192	Cortical potentials related to assessment of pain intensity with visual analogue scale (VAS)	2002	113	7	1013â€“1024		Kanda, M and Matsuhashi, M and Sawamoto, N and Oga, T and Mima, T and Nagamine, T and Shibasaki, H	RAYYAN-INCLUSION: {"Vickie"=>"Excluded", "jsong6"=>"Excluded"} | RAYYAN-EXCLUSION-REASONS: irrelevant
rayyan-283776193	Hematopoietic stem cell transplantation for chronic inflammatory demyelinating polyradiculoneuropathy	2020					Burt, RK and Balabanov, R and Tavee, J and Han, X and Sufit, R and Ajroud-Driss, S and Jovanovic, B and Quigley, K and Arnautovic, I and Helenowski, I and Sharrack, B	RAYYAN-INCLUSION: {"Vickie"=>"Excluded", "jsong6"=>"Excluded"} | RAYYAN-EXCLUSION-REASONS: irrelevant
rayyan-283776194	Effect of pranayama and meditation on psychological well-being of healthcare workers during COVID-19 pandemic	2020					CTRI/2020/05/025162,	RAYYAN-INCLUSION: {"Vickie"=>"Excluded", "jsong6"=>"Excluded"} | RAYYAN-EXCLUSION-REASONS: irrelevant
rayyan-283776195	Randomized Controlled Trial of Exercise in CKDÃ¢â‚¬â€The RENEXC Study	2019	4	7	963â€“976		Hellberg, M and Hoglund, P and Svensson, P and Clyne, N	RAYYAN-INCLUSION: {"Vickie"=>"Excluded", "jsong6"=>"Excluded"} | RAYYAN-EXCLUSION-REASONS: irrelevant
rayyan-283776196	The effects of mirror and non-mirror visual feedback conditions during unilateral exercise training on contralateral strength and passive joint position sense	2018	3	1	52â€“		Denizoglu Kulli, H and Durgut, E and Alpay, K	RAYYAN-INCLUSION: {"Vickie"=>"Excluded", "jsong6"=>"Excluded"} | RAYYAN-EXCLUSION-REASONS: irrelevant
rayyan-283776197	Effects of nutritional supplementation and resistance training on muscle strength in free living elders. Results of one year follow	2004	8	2	68â€“75		Bunout, B and Barrera, G and de la Maza, P and Avendano, M and Gattas, V and Petermann, M and Hirsh, S	RAYYAN-INCLUSION: {"Vickie"=>"Excluded", "jsong6"=>"Excluded"} | RAYYAN-EXCLUSION-REASONS: irrelevant
rayyan-283776198	"Knuckle Cracking": can Blinded Observers Detect Changes with Physical Examination and Sonography?	2017			1â€“7		Boutin, RD and Netto, AP and Nakamura, D and Bateni, C and Szabo, RM and Cronan, M and Foster, B and Barfield, WR and Seibert, JA and Chaudhari, AJ	RAYYAN-INCLUSION: {"Vickie"=>"Excluded", "jsong6"=>"Excluded"} | RAYYAN-EXCLUSION-REASONS: irrelevant
rayyan-283776199	Action observation training modifies the function and structure of the mirror neuron system in multiple sclerosis patients with right upper limb motor deficits	2017	88	16			Rocca, M and Fumagalli, S and Ravetto, A and Dirito, AM and Preziosa, P and Gatti, R and Boneschi, FM and Comola, M and Comi, G and Filippi, M	RAYYAN-INCLUSION: {"Vickie"=>"Excluded", "jsong6"=>"Excluded"} | RAYYAN-EXCLUSION-REASONS: irrelevant
rayyan-283776202	Implementation of specific strength training among industrial laboratory technicians: long-term effects on back, neck and upper extremity pain	2013	14				Pedersen, MT and Andersen, CH and Zebis, MK and Sjogaard, G and Andersen, LL	RAYYAN-INCLUSION: {"Vickie"=>"Excluded", "jsong6"=>"Excluded"} | RAYYAN-EXCLUSION-REASONS: irrelevant
rayyan-283776203	Total sleep deprivation and pain perception during cold noxious stimuli in older adults	2019	42		A91â€“A92		Cunningham, HA and Greenlund, IM and Fonkoue, IT and Smoot, CA and Carter, JR	RAYYAN-INCLUSION: {"Vickie"=>"Excluded", "jsong6"=>"Excluded"} | RAYYAN-EXCLUSION-REASONS: irrelevant
rayyan-283776204	Compare the effectiveness of hematoma block versus intravenously sedation for reduction of colles fracture	2021	15	8	2519â€“2521		Hussain, S and Khan, Z and Khan, MH and Khan, MG and Bahadar, J and Badshah, J	RAYYAN-INCLUSION: {"Vickie"=>"Excluded", "jsong6"=>"Excluded"} | RAYYAN-EXCLUSION-REASONS: irrelevant
rayyan-283776205	Anemia prevalence, severity and associated factors among children aged 6-71â€‰months in rural Hunan Province, China: a community-based cross-sectional study	2020	20	1	989â€“		Li, H and Xiao, J and Liao, M and Huang, G and Zheng, J and Wang, H and Huang, Q and Wang, A	RAYYAN-INCLUSION: {"Vickie"=>"Excluded", "jsong6"=>"Excluded"} | RAYYAN-EXCLUSION-REASONS: irrelevant
rayyan-283776206	Altered perceptual hand maps in focal hand dystonia	2020	35		S60â€“		Mainka, T and Ossmann, B and Zeuner, K and Baumann, A and Baumer, T and Kuhn, A and Azanon Gracia, E and Zittel, S and Longo, M and Ganos, C	RAYYAN-INCLUSION: {"Vickie"=>"Excluded", "jsong6"=>"Excluded"} | RAYYAN-EXCLUSION-REASONS: irrelevant
rayyan-283776207	Changes in aquagenic wrinkling of the palms in patients with cystic fibrosis treated with ivacaftor	2021	203	9			Jacobi, E and Avolio, J and Shaw, M and Gonska, T and Ratjen, FA and Solomon, M and Grasemann, H	RAYYAN-INCLUSION: {"Vickie"=>"Excluded", "jsong6"=>"Excluded"} | RAYYAN-EXCLUSION-REASONS: irrelevant
rayyan-283776208	PARK-BAND training program for Parkinson's disease in clinical settings: a feasibility study	2021	36		S183â€“		Lima. D and Bonfadini. J and Almeida. S and Sobreira. E and Damasceno. P and Alencar. M and Viana-Junior. A and Luna. J and Rodrigues. P and Pereira. I and Gadelha. A and Oliveira. P and C	RAYYAN-INCLUSION: {"Vickie"=>"Excluded", "jsong6"=>"Excluded"} | RAYYAN-EXCLUSION-REASONS: irrelevant
rayyan-283776209	Transcranial alternating current stimulation does not change the common input to the motor neuron pool	2020	131	4	e158â€“		Ibanez, J and Spampinato, D and Del Vecchio, A and Vollette, CA and Casolo, A and Rothwell, JC and Farina, D	RAYYAN-INCLUSION: {"Vickie"=>"Excluded", "jsong6"=>"Excluded"} | RAYYAN-EXCLUSION-REASONS: irrelevant
rayyan-283776210	Strengthening inhibitory system through SICI neurofeedback training suppresses motor learning	2020	131	4	e155â€“		Gyoda, T and Nojima, I and Huang, YZ	RAYYAN-INCLUSION: {"Vickie"=>"Excluded", "jsong6"=>"Excluded"} | RAYYAN-EXCLUSION-REASONS: irrelevant
rayyan-283776211	Microbiological evaluation of the efficacy of soapy water to clean hands	2012	87	5		429	Amin, MN and Pickering, AJ and Ram, PK and Unicomb, L and Najnin, N and Homaira, N and Ashraf, S and Abedin, J and Islam, MS and Luby, SP	RAYYAN-INCLUSION: {"Vickie"=>"Excluded", "jsong6"=>"Excluded"} | RAYYAN-EXCLUSION-REASONS: irrelevant
rayyan-283776212	Comparison of laser Doppler imaging, fingertip lacticemy test, and nailfold capillaroscopy for assessment of digital microcirculation in systemic sclerosis	2010	12	4	R157		Correa, MJ and Andrade, LE and Kayser, C	RAYYAN-INCLUSION: {"Vickie"=>"Excluded", "jsong6"=>"Excluded"} | RAYYAN-EXCLUSION-REASONS: irrelevant
rayyan-283776213	Proof of principle: traumatic brain injury speech rehabilitation under direct current stimulation (BISCUIT)	2019	130	8	e149â€“		Rackoll, T and Darkow, R and Wusten, K and Floel, A and Gorsler, A	RAYYAN-INCLUSION: {"Vickie"=>"Excluded", "jsong6"=>"Excluded"} | RAYYAN-EXCLUSION-REASONS: irrelevant
rayyan-283776214	Effects of anodal transcranial direct current stimulation combined with motor imagery on motor sequence learning in healthy older adults	2020	131	4	e93â€“e94		Metais, A and Muller, C and Breuil, C and Boublay, N and Di Rienzo, F and Guillot, A and Collet, C and Krolak-Salmon, P and Saimpont, A	RAYYAN-INCLUSION: {"Vickie"=>"Excluded", "jsong6"=>"Excluded"} | RAYYAN-EXCLUSION-REASONS: irrelevant
rayyan-283776215	Improvement of psoriatic onychodystrophy by a water-soluble nail lacquer	2009	23	7	832â€“834		Cantoresi, F and Sorgi, P and Arcese, A and Bidoli, A and Bruni, F and Carnevale, C and Calvieri, S	RAYYAN-INCLUSION: {"Vickie"=>"Excluded", "jsong6"=>"Excluded"} | RAYYAN-EXCLUSION-REASONS: irrelevant
rayyan-283776216	The effect of high-definition transcranial direct current stimulation intensity on motor performance in healthy adults: a randomized controlled trial	2021	18	1		103	Lerner, O and Friedman, J and Frenkel-Toledo, S	RAYYAN-INCLUSION: {"Vickie"=>"Excluded", "jsong6"=>"Excluded"} | RAYYAN-EXCLUSION-REASONS: irrelevant
rayyan-283776217	The effect of acute sleep deprivation on skin conductance response and its correlation to fmri data during fear conditioning	2015	38		A116		McNamee, R and Conrad, T and Pace-Schott, E and Milad, M and Germain, A	RAYYAN-INCLUSION: {"Vickie"=>"Excluded", "jsong6"=>"Excluded"} | RAYYAN-EXCLUSION-REASONS: acute,irrelevant
rayyan-283776218	Effect of Needle Gauge on Pain and Anxiety During Dental Anesthesia	2020					IRCT20190702044078N1,	RAYYAN-INCLUSION: {"Vickie"=>"Excluded", "jsong6"=>"Excluded"} | RAYYAN-EXCLUSION-REASONS: irrelevant
rayyan-283776219	An international. multicenter. randomized. open-label. safety and efficacy trial of intravenous zoledronic acid administered either once or twice yearly in children with severe osteogenesis imperfecta. a 1-year extensio	2004					EUCTR2004-001666-40-HU,	RAYYAN-INCLUSION: {"Vickie"=>"Excluded", "jsong6"=>"Excluded"} | RAYYAN-EXCLUSION-REASONS: irrelevant
rayyan-283776220	Incidence of microperforation for surgical gloves depends on duration of wear	2009	30	5	409â€“414		Partecke, LI and Goerdt, AM and Langner, I and Jaeger, B and Assadian, O and Heidecke, CD and Kramer, A and Huebner, NO	RAYYAN-INCLUSION: {"Vickie"=>"Excluded", "jsong6"=>"Excluded"} | RAYYAN-EXCLUSION-REASONS: irrelevant
rayyan-283776221	Optimal chest compression technique for paediatric cardiac arrest victims	2015	23			36	Kim, MJ and Lee, HS and Kim, S and Park, YS	RAYYAN-INCLUSION: {"Vickie"=>"Excluded", "jsong6"=>"Excluded"} | RAYYAN-EXCLUSION-REASONS: irrelevant
rayyan-283776222	Effect of audio distraction methods on pain and anxiety during dental treatment	2019					IRCT20190702044077N1,	RAYYAN-INCLUSION: {"Vickie"=>"Excluded", "jsong6"=>"Excluded"} | RAYYAN-EXCLUSION-REASONS: irrelevant
rayyan-283776223	Treatment of systemic sclerosis with extracorporeal photochemotherapy. Results of a multicenter trial	1992	128	3	337â€“346		Rook, AH and Freundlich, B and Jegasothy, BV and Perez, MI and Barr, WG and Jimenez, SA and Rietschel, RL and Wintroub, B and Kahaleh, MB and Varga, J	RAYYAN-INCLUSION: {"Vickie"=>"Excluded", "jsong6"=>"Excluded"} | RAYYAN-EXCLUSION-REASONS: irrelevant
rayyan-283776225	Effect of sensory stimulation (acupuncture) on sympathetic and parasympathetic activities in healthy subjects	2000	79	1	52â€“59		Haker, E and Egekvist, H and Bjerring, P	RAYYAN-INCLUSION: {"Vickie"=>"Excluded", "jsong6"=>"Excluded"} | RAYYAN-EXCLUSION-REASONS: irrelevant
rayyan-283776226	Assessment of Vasomotion of People With Spinal Cord Injury	2020					NCT04215939,	RAYYAN-INCLUSION: {"Vickie"=>"Excluded", "jsong6"=>"Excluded"} | RAYYAN-EXCLUSION-REASONS: irrelevant
rayyan-283776227	Results of the second interim assessment of rEECur, an international randomized controlled trial of chemotherapy for the treatment of recurrent and primary refractory Ewing sarcoma (RR-ES)	2020	38	15			McCabe. MG and Kirton. L and Khan. M and Fenwick. N and Dirksen. U and Gaspar. N and Kanerva. J and Kuehne. T and Longhi. A and Luksch. R and Mata. C and Phillips. M and Safwat. A and	RAYYAN-INCLUSION: {"Vickie"=>"Excluded", "jsong6"=>"Excluded"} | RAYYAN-EXCLUSION-REASONS: irrelevant
rayyan-283776228	Istradefylline effects on tremor dominant (TD) and postural instability and gait difficulty (PIGD) motor subtypes	2021	96	15			Pagan, F and Parno, J and Ristuccia, R and Mori, A and Torres-Yaghi, Y	RAYYAN-INCLUSION: {"Vickie"=>"Excluded", "jsong6"=>"Excluded"} | RAYYAN-EXCLUSION-REASONS: irrelevant
rayyan-283776229	Ergonomic evaluation of an endoscope support stand during simulated colonoscopies	2018	87	6	AB506â€“		Shergill, AK and Barr, A and Harris-Adamson, C	RAYYAN-INCLUSION: {"Vickie"=>"Excluded", "jsong6"=>"Excluded"} | RAYYAN-EXCLUSION-REASONS: irrelevant
rayyan-283776230	A clinical trail to study the effectiveness of balance exercise on risk of fall prevention among older people in older people in selected old age homes at Bhubaneswar, orissa.(Pilot study)	2020					CTRI/2020/09/027845,	RAYYAN-INCLUSION: {"Vickie"=>"Excluded", "jsong6"=>"Excluded"} | RAYYAN-EXCLUSION-REASONS: irrelevant
rayyan-283776231	The 'LASOO' approach to mask-ventilation: a pilot randomised controlled trial vs the traditional 'EC' method	2017	72		46â€“		Cunningham, J and Shelton, C and Dobson, A and Delis, E and Alexander, P and Findlay, D and Talbot, L	RAYYAN-INCLUSION: {"Vickie"=>"Excluded", "jsong6"=>"Excluded"} | RAYYAN-EXCLUSION-REASONS: irrelevant
rayyan-283776233	TMS and Attentional Bias in Functional Motor Disorder	2014					NCT02102906,	RAYYAN-INCLUSION: {"Vickie"=>"Excluded", "jsong6"=>"Excluded"} | RAYYAN-EXCLUSION-REASONS: irrelevant
rayyan-283776234	Closed-circuit anesthesia preserves skin blood flow during surgery	2006	44	1	25â€“30		Ke, YC and Ho, ST and Yi, HD and Li, CY and Wang, JJ and Lin, CY and Lu, CC	RAYYAN-INCLUSION: {"Vickie"=>"Excluded", "jsong6"=>"Excluded"} | RAYYAN-EXCLUSION-REASONS: irrelevant
rayyan-283776235	Clinical Evaluation of Intravenous SG-75 in Treatment of Unstable Angina: a Multicenter Double-Blind Comparative Study with Intravenous ISDN	1991	7	9	2031â€“2054		Kato. K and Iinuma. H and Hirosawa. K and Sugimoto. T and Uchida. Y and Hosoda. S and Havakawa. H and Kishida. H and Takano. T and Katagiri. T and Osada. H and Hiramori. K and Fukami.	RAYYAN-INCLUSION: {"Vickie"=>"Excluded", "jsong6"=>"Excluded"} | RAYYAN-EXCLUSION-REASONS: irrelevant
rayyan-283776236	The Effect Of Ice Massage On PerceÃ±ved Pain	2020					NCT04288388,	RAYYAN-INCLUSION: {"Vickie"=>"Excluded", "jsong6"=>"Excluded"} | RAYYAN-EXCLUSION-REASONS: irrelevant
rayyan-283776237	Prospective Nail Polish Study	2022					NCT05181761,	RAYYAN-INCLUSION: {"Vickie"=>"Excluded", "jsong6"=>"Excluded"} | RAYYAN-EXCLUSION-REASONS: irrelevant
rayyan-283776238	Comparison Between Rigid Video Assisted Laryngoscopy vs Flexible Laryngoscopy in Anticipated Difficult Intubation	2020					NCT04628611,	RAYYAN-INCLUSION: {"Vickie"=>"Excluded", "jsong6"=>"Excluded"} | RAYYAN-EXCLUSION-REASONS: irrelevant
rayyan-283776239	The effect of topical application of lavender essential oil on physiological indexes and pain intensity during venous cannulation	2018					IRCT20171226038078N2,	RAYYAN-INCLUSION: {"Vickie"=>"Excluded", "jsong6"=>"Excluded"} | RAYYAN-EXCLUSION-REASONS: irrelevant
rayyan-283776240	Upskilling physiotherapists in evidence-based assessment and management of patients with rheumatoid arthritis: a single-blind randomised controlled trial	2013					ACTRN12613000659707,	RAYYAN-INCLUSION: {"Vickie"=>"Excluded", "jsong6"=>"Excluded"} | RAYYAN-EXCLUSION-REASONS: irrelevant
rayyan-283776241	The Effect of Acupressure and Deep Breathing on Anxiety, Fatigue and Sleep Quality	2020					IRCT20200305046701N1,	RAYYAN-INCLUSION: {"Vickie"=>"Excluded", "jsong6"=>"Excluded"} | RAYYAN-EXCLUSION-REASONS: irrelevant
rayyan-283776242	To compare two techniques of laparoscopic rectal prolapse surgery	2020					CTRI/2020/12/030034,	RAYYAN-INCLUSION: {"Vickie"=>"Excluded", "jsong6"=>"Excluded"} | RAYYAN-EXCLUSION-REASONS: irrelevant
rayyan-283776243	The Comparison of the Effect of Needle Gauge on Pain and Anxiety of Children During Articaine Mandibular Buccal Infiltration Anesthesia	2020					IRCT20200509047369N1,	RAYYAN-INCLUSION: {"Vickie"=>"Excluded", "jsong6"=>"Excluded"} | RAYYAN-EXCLUSION-REASONS: irrelevant
rayyan-283776244	The effect of foot reflexology on patients undergoing renal transplantation surgery	2018					IRCT20170116031972N6,	RAYYAN-INCLUSION: {"Vickie"=>"Excluded", "jsong6"=>"Excluded"} | RAYYAN-EXCLUSION-REASONS: acute,irrelevant
rayyan-283776246	Ultrasound-guided venous access for PMK and ICD. randomised trial	2016	18			378	Mattia Liccardo, M and Liccardo, ML and Nocerino, PN and Borrino, AB and Carbone, CC and Di Meo, EDM and Salzano, GS	RAYYAN-INCLUSION: {"Vickie"=>"Excluded", "jsong6"=>"Excluded"} | RAYYAN-EXCLUSION-REASONS: irrelevant
rayyan-283776247	A Long-Acting Growth Hormone (Somavaratan, VRS-317) in Children compared to Daily rhGH	2015					EUCTR2014-004525-41-BE,	RAYYAN-INCLUSION: {"Vickie"=>"Excluded", "jsong6"=>"Excluded"} | RAYYAN-EXCLUSION-REASONS: irrelevant
rayyan-283776248	The effect of jacobson relaxation onfamily Caregivers	2021					IRCT20210808052114N1,	RAYYAN-INCLUSION: {"Vickie"=>"Excluded", "jsong6"=>"Excluded"} | RAYYAN-EXCLUSION-REASONS: irrelevant
rayyan-283776249	The effectiveness and efficacy of splints for thumb base osteoarthritis: a pilot randomized controlled trial	2014	53		i41â€“i42		Adams, J and BoucÂ¸as, SB and Hislop, K and Dziedzic, K and Arden, N and Graham, C and McKenna, H and Jansen, V and Shaylor, B and Macleod, C and Bancroft, K and Bradley, S	RAYYAN-INCLUSION: {"Vickie"=>"Excluded", "jsong6"=>"Excluded"} | RAYYAN-EXCLUSION-REASONS: irrelevant
rayyan-283776250	Intracorporeal laparoscopic suturing training: computers versus the instructor	2021	128		258â€“259		Lazaridis, A and Navaratnarajah, R and Iliodromiti, S and Odejinmi, F	RAYYAN-INCLUSION: {"Vickie"=>"Excluded", "jsong6"=>"Excluded"} | RAYYAN-EXCLUSION-REASONS: irrelevant
rayyan-283776251	No overt effects of a 6-week exergame training on sensorimotor and cognitive function in older adults. A preliminary investigation	2017	11				Ordnung, M and Hoff, M and Kaminski, E and Villringer, A and Ragert, P	RAYYAN-INCLUSION: {"Vickie"=>"Excluded", "jsong6"=>"Excluded"} | RAYYAN-EXCLUSION-REASONS: irrelevant
rayyan-283776252	03: 18 PM Abstract No. 65 Palmar warming (PaW) technique for radial artery vasodilation to facilitate transradial access: a randomized controlled trial	2019	30	3	S31â€“S32		Hedge, J and Jahangiri, Y and Farsad, K and Kaufman, J and Galuppo Monticelli, R and Al-Hakim, R	RAYYAN-INCLUSION: {"Vickie"=>"Excluded", "jsong6"=>"Excluded"} | RAYYAN-EXCLUSION-REASONS: irrelevant
rayyan-283776253	Ipsilesional Mu Rhythm Desynchronization Correlates With Improvements in Affected Hand Grip Strength and Functional Connectivity in Sensorimotor Cortices Following BCI-FES Intervention for Upper Extremity in St	2021	15				Remsik. AB and Gjini. K and Williams. L and van Kan. PLE and Gloe. S and Biorklund. E and Rivera. CA and Romero. S and Young. BM and Nair. VA and Caldera. KE and Williams. JC and Prabhak	RAYYAN-INCLUSION: {"Vickie"=>"Excluded", "jsong6"=>"Excluded"} | RAYYAN-EXCLUSION-REASONS: irrelevant
rayyan-283776254	The effect of two methods of resistance training (Rest-Pause, FST7) on anthropometric indices, muscle strength and thyroid hormones in young bodybuilders	2021					IRCT20180503039517N8,	RAYYAN-INCLUSION: {"Vickie"=>"Excluded", "jsong6"=>"Excluded"} | RAYYAN-EXCLUSION-REASONS: irrelevant
rayyan-283776255	A single-center. prospective. randomized. double-blind. intra-patient (left-to-right) placebo-controlled. pilot study to assess the efficacy and safety of a betamethasone valerate 0.1% medicated plaster in the treatment	2014					EUCTR2014-000291-25-IT,	RAYYAN-INCLUSION: {"Vickie"=>"Excluded", "jsong6"=>"Excluded"} | RAYYAN-EXCLUSION-REASONS: irrelevant
rayyan-283776256	Movement visualisation in virtual reality-based systems for motor rehabilitation: effect of virtual body shape on embodiment	2016	22		S59â€“		Dos Santos, LF and Epple, S and Gierig, S and Schmidt, H and Kruger, J and Dohle, C	RAYYAN-INCLUSION: {"Vickie"=>"Excluded", "jsong6"=>"Excluded"} | RAYYAN-EXCLUSION-REASONS: irrelevant
rayyan-283776257	Wireless Sensing of Lower Lip and Thumb-Index Finger 'Ramp-and-Hold' Isometric Force Dynamics in a Small Cohort of Unilateral MCA Stroke: Discussion of Preliminary Findings	2020	20	4			Barlow, S and Custead, R and Lee, J and Hozan, M and Greenwood, J	RAYYAN-INCLUSION: {"Vickie"=>"Excluded", "jsong6"=>"Excluded"} | RAYYAN-EXCLUSION-REASONS: irrelevant
rayyan-283776259	The Effect of Sonophoresis on Topical Anesthesia: A Pilot Study	2011					NCT01283490,	RAYYAN-INCLUSION: {"Vickie"=>"Excluded", "jsong6"=>"Excluded"} | RAYYAN-EXCLUSION-REASONS: irrelevant
rayyan-283776260	Ultrasound guided venous access for pacemaker and defibbrillators. Randomized trial	2015	4			46	Liccardo, M and Nocerino, P and Urso, AC and Borrino, A and Carbone, C and Follero, P and Ciardiello, C and Prato, L and Salzano, G	RAYYAN-INCLUSION: {"Vickie"=>"Excluded", "jsong6"=>"Excluded"} | RAYYAN-EXCLUSION-REASONS: irrelevant
rayyan-283776261	Phase II Clinical Study of Protection of Nail Change and Skin Toxicity by Using a Frozen Glove in Japanese Patients with Early Breast Cancer Treated by Docetaxel and Cyclophosphamide (TC)	2010	69	24			Hayashi, T and Fujita, T and Mase, T and Nakano, S and Wada, M and Kashizuka, T	RAYYAN-INCLUSION: {"Vickie"=>"Excluded", "jsong6"=>"Excluded"} | RAYYAN-EXCLUSION-REASONS: irrelevant
rayyan-283776262	Intra-operative nasal compression after lateral osteotomy to minimize post-operative Peri-orbital ecchymosis and edema	2019	48	1		50	Sowerby, L and Kim, LM and Chow, W and Moore, C	RAYYAN-INCLUSION: {"Vickie"=>"Excluded", "jsong6"=>"Excluded"} | RAYYAN-EXCLUSION-REASONS: irrelevant
rayyan-283776263	Similarity between carotid and coronary artery responses to sympathetic stimulation and the role of ÃŽÂ±1-receptors in humans	2018	125	2	409â€“418		van Mil, ACCM and Tymko, MM and Kerstens, TP and Stembridge, M and Green, DJ and Ainslie, PN and Thijssen, DHJ	RAYYAN-INCLUSION: {"Vickie"=>"Excluded", "jsong6"=>"Excluded"} | RAYYAN-EXCLUSION-REASONS: irrelevant
rayyan-283776264	Selection of Patients' Recumbent Position Laterality According to Physician Handedness Bias Increases the Success Rate of Lumbar Puncture: a Multicenter Study	2019	31	3	318â€“322		Zhao, JL and Sun, YR and Shan, Q and Xiao, ZP and Hou, BC and Hu, J	RAYYAN-INCLUSION: {"Vickie"=>"Excluded", "jsong6"=>"Excluded"} | RAYYAN-EXCLUSION-REASONS: irrelevant
rayyan-283776265	Improved nondominant hand performance on a laparoscopic virtual reality simulator after playing the Nintendo Wii	2013	27	11	4224â€“4231		Middleton, KK and Hamilton, T and Tsai, PC and Middleton, DB and Falcone, JL and Hamad, G	RAYYAN-INCLUSION: {"Vickie"=>"Excluded", "jsong6"=>"Excluded"} | RAYYAN-EXCLUSION-REASONS: irrelevant,wrong study design
rayyan-283776267	The influence of training with heavy rugby balls on selected spin pass variables in youth rugby union players	2008	11	2	209â€“213		Hooper, JJ and James, SD and Jones, DC and Lee, DM and GÃ¡l, JM	RAYYAN-INCLUSION: {"Vickie"=>"Excluded", "jsong6"=>"Excluded"} | RAYYAN-EXCLUSION-REASONS: irrelevant
rayyan-283776268	Effect of Tactile Sensory Substitution on the Proprioceptive Error Map of the Arm	2021	15				Tanner, J and Orthlieb, G and Shumate, D and Helms Tillery, S	RAYYAN-INCLUSION: {"Vickie"=>"Excluded", "jsong6"=>"Excluded"} | RAYYAN-EXCLUSION-REASONS: irrelevant
rayyan-283776269	Clinical study on the treatment of knee osteoarthritis with massage combined with warm acupuncture	2019	27		S221â€“		Zhang, H and Dou, Q and Zhu, T	RAYYAN-INCLUSION: {"Vickie"=>"Excluded", "jsong6"=>"Excluded"} | RAYYAN-EXCLUSION-REASONS: irrelevant
rayyan-283776270	Intravenous Iron Supplement for Iron Deficiency in Patients With Severe Aortic Stenosis	2019					NCT04206228,	RAYYAN-INCLUSION: {"Vickie"=>"Excluded", "jsong6"=>"Excluded"} | RAYYAN-EXCLUSION-REASONS: irrelevant
rayyan-283776271	Compare the effect of pranayama breathing and hand reflexology on anxiety in patients undergoing coronary angiography	2016					IRCT201308024655N6,	RAYYAN-INCLUSION: {"Vickie"=>"Excluded", "jsong6"=>"Excluded"} | RAYYAN-EXCLUSION-REASONS: irrelevant
rayyan-283776272	An Integrative Approach to Managing Rheumatoid Arthritis (RA): healing the Body, Mind & Spirit	2020	72		4153â€“4155		Abeson, E	RAYYAN-INCLUSION: {"Vickie"=>"Excluded", "jsong6"=>"Excluded"} | RAYYAN-EXCLUSION-REASONS: irrelevant
rayyan-283776273	Association Between Maximal Bench Press Strength and Isometric Handgrip Strength Among Breast Cancer Survivors	2017	98	2	264â€“269		Rogers, BH and Brown, JC and Gater, DR and Schmitz, KH	RAYYAN-INCLUSION: {"Vickie"=>"Excluded", "jsong6"=>"Excluded"} | RAYYAN-EXCLUSION-REASONS: irrelevant
rayyan-283776274	Physical exercise versus exercise program using electrical stimulation devices for home use	2008	42	1	117â€“122		Santos, FM and Rodrigues, RG and Trindade-Filho, EM	RAYYAN-INCLUSION: {"Vickie"=>"Excluded", "jsong6"=>"Excluded"} | RAYYAN-EXCLUSION-REASONS: irrelevant
rayyan-283776275	Reduction of the bilateral deficit following unilateral strength training: a TMS study	2010	13		e88		Pearce, A and Kidgell, D	RAYYAN-INCLUSION: {"Vickie"=>"Excluded", "jsong6"=>"Excluded"} | RAYYAN-EXCLUSION-REASONS: irrelevant,no right and left limb training
rayyan-283776276	Upper limb motor training based on task-oriented exercises induces functional brain reorganization in patients with multiple sclerosis	2019	410		150â€“159		Bonzano, L and PedullÃ , L and Tacchino, A and Brichetto, G and Battaglia, MA and Mancardi, GL and Bove, M	RAYYAN-INCLUSION: {"Vickie"=>"Excluded", "jsong6"=>"Excluded"} | RAYYAN-EXCLUSION-REASONS: irrelevant
rayyan-283776277	Cross-transfer effects in the upper extremity during an occupationally embedded exercise	2001	55	3	317â€“323		Nagel, MJ and Rice, MS	RAYYAN-INCLUSION: {"Vickie"=>"Excluded", "jsong6"=>"Excluded"} | RAYYAN-EXCLUSION-REASONS: irrelevant,wrong study design
rayyan-283776278	Methylphenidate improves visual-spatial memory in children with attention-deficit/hyperactivity disorder	2004	43	3	260â€“268		Bedard, AC and Martinussen, R and Ickowicz, A and Tannock, R	RAYYAN-INCLUSION: {"Vickie"=>"Excluded", "jsong6"=>"Excluded"} | RAYYAN-EXCLUSION-REASONS: irrelevant
rayyan-283776279	Lateralized ultradian rhythms: evidence from tactile discrimination of either hand	1998	87	3	1015â€“1034		Meier-Koll, A	RAYYAN-INCLUSION: {"Vickie"=>"Excluded", "jsong6"=>"Excluded"} | RAYYAN-EXCLUSION-REASONS: irrelevant
rayyan-283776280	Step aerobic vs. cycle ergometer training: effects on aerobic capacity, coordinative tasks, and pleasure in untrained adults--a randomized controlled trial	2002	114	23	992â€“998		Kerschan-Schindl, K and Wiesinger, G and Zauner-Dungl, A and Kollmitzer, J and Fialka-Moser, V and Quittan, M	RAYYAN-INCLUSION: {"Vickie"=>"Excluded", "jsong6"=>"Excluded"} | RAYYAN-EXCLUSION-REASONS: irrelevant
rayyan-283776281	Deep Brain Stimulation for Spasmodic Dysphonia: A Blinded Comparison of Unilateral and Bilateral Stimulation in Two Patients	2020	98	3	200â€“205		Kruger, MT and Kruger, MT and Kruger, MT and Kruger, MT and Hu, A and Honey, CR	RAYYAN-INCLUSION: {"Vickie"=>"Excluded", "jsong6"=>"Excluded"} | RAYYAN-EXCLUSION-REASONS: irrelevant
rayyan-283776282	Compensatory changes accompanying chronic forced use of the nondominant hand by unilateral amputees	2014	34	10	3622â€“3631		Philip, BA and Frey, SH	RAYYAN-INCLUSION: {"Vickie"=>"Excluded", "jsong6"=>"Excluded"} | RAYYAN-EXCLUSION-REASONS: irrelevant
rayyan-283776283	Effects of tDCS and HD-tDCS on motor learning and motor maps in children	2018	129		e119â€“e120		Giuffre, A and Cole, L and Ciechanski, P and Carlson, H and Zewdie, E and Kuo, H-C and Kahl, C and Webber, A and Metzler, M and Harris, A and Kirton, A	RAYYAN-INCLUSION: {"Vickie"=>"Excluded", "jsong6"=>"Excluded"} | RAYYAN-EXCLUSION-REASONS: irrelevant
rayyan-283776284	Pain processing in four regions of human cingulate cortex localized with co-registered PET and MR imaging	1996	8	7	1461â€“1473		Vogt, BA and Derbyshire, S and Jones, AKP	RAYYAN-INCLUSION: {"Vickie"=>"Excluded", "jsong6"=>"Excluded"} | RAYYAN-EXCLUSION-REASONS: irrelevant
rayyan-283776285	Stimulation of reaching behavior using sticky mittens in preterm infants	2016					RBR-386vfr,	RAYYAN-INCLUSION: {"Vickie"=>"Excluded", "jsong6"=>"Excluded"} | RAYYAN-EXCLUSION-REASONS: irrelevant
rayyan-283776286	COMPARISON OF ULTRASOUND GUIDED RECTUS SHEATH BLOCK AND OBLIQUE SUBCOSTAL TRANSVERSUS ABDOMINIS PLANE BLOCK FOR PERIOPERATIVE ANALGESIA FOR MIDLINE INCISION ABDOMINAL SURGERII	2021					CTRI/2021/07/034703,	RAYYAN-INCLUSION: {"Vickie"=>"Excluded", "jsong6"=>"Excluded"} | RAYYAN-EXCLUSION-REASONS: irrelevant
rayyan-283776287	Protective effect of otago exercise programme on fall prevention in elderly patients discharged after total hip replacement	2020	127		224â€“225		Bu, D and Wang, G	RAYYAN-INCLUSION: {"Vickie"=>"Excluded", "jsong6"=>"Excluded"} | RAYYAN-EXCLUSION-REASONS: irrelevant
rayyan-283776288	Effect of physical exercise interventions on musculoskeletal pain in all body regions among office workers: a one-year randomized controlled trial	2010	15	1	100â€“104		Andersen, LL and Christensen, KB and Holtermann, A and Poulsen, OM and SjÃ¸gaard, G and Pedersen, MT and Hansen, EA	RAYYAN-INCLUSION: {"Vickie"=>"Excluded", "jsong6"=>"Excluded"} | RAYYAN-EXCLUSION-REASONS: irrelevant
rayyan-283776289	Synchronized Pedaling with Martial Arts Improves Quality of Life of Women with Breast Cancer	2018	39	13	978â€“983		Navarro-Sanz, A and Espejo-Reina, A and Cerezo-Guzman, MV and Fernandez-Ortega, JF and Meza-Leiva, H and Conejo-Tirado, I and Santiago-Sanchez, C	RAYYAN-INCLUSION: {"Vickie"=>"Excluded", "jsong6"=>"Excluded"} | RAYYAN-EXCLUSION-REASONS: irrelevant
rayyan-283776290	Two different sets of handexercises improved grip strength after eight weeks in patients with arthritis	2014	73					RAYYAN-INCLUSION: {"Vickie"=>"Excluded", "jsong6"=>"Excluded"} | RAYYAN-EXCLUSION-REASONS: irrelevant,wrong study design
rayyan-283776292	Utility of a 3D navigation system to reduce ionising radiation exposure in common flutter ablation. Study of operator and patient dosimetry	2020	75	6	585â€“		Sanchez Ruiz, Y and Scavee, C and Marchandise, S and Belda Cuesta, E and Garnir, Q and Le Polain de Waroux, J-B and Lichtherte, S	RAYYAN-INCLUSION: {"Vickie"=>"Excluded", "jsong6"=>"Excluded"} | RAYYAN-EXCLUSION-REASONS: irrelevant
rayyan-283776293	Evaluating an exercise game session in the ExerCube	2020					ISRCTN43067716,	RAYYAN-INCLUSION: {"Vickie"=>"Excluded", "jsong6"=>"Excluded"} | RAYYAN-EXCLUSION-REASONS: acute,irrelevant
rayyan-283776294	A prospective. multicenter. randomized. evaluator-blinded. split-hand study to evaluate the effectiveness and safety of largegel particle hyaluronic acid with lidocaine for the correction of volume deficits in the dorsal h	2019	12	5	S13â€“		Moradi, A and Allen, S and Bank, D and Marmu, E and Fagien, S and Glaser, DA and Maguire, C and Cohen, J and Arlati, L	RAYYAN-INCLUSION: {"Vickie"=>"Excluded", "jsong6"=>"Excluded"} | RAYYAN-EXCLUSION-REASONS: irrelevant
rayyan-283776295	Comparison of human cerebral activation pattern during cutaneous warmth, heat pain, and deep cold pain	1996	76	1	571â€“581		Casey, KL and Minoshima, S and Morrow, TJ and Koeppe, RA	RAYYAN-INCLUSION: {"Vickie"=>"Excluded", "jsong6"=>"Excluded"} | RAYYAN-EXCLUSION-REASONS: irrelevant
rayyan-283776296	The time-related information about a nocebo treatment can modify its onset of action but not the heart rate variability	2020					ISRCTN96623027,	RAYYAN-INCLUSION: {"Vickie"=>"Excluded", "jsong6"=>"Excluded"} | RAYYAN-EXCLUSION-REASONS: irrelevant
rayyan-283776297	Cervical radiculopathy effectively managed by a physical therapy student utilizing a cervical lateral glide manipulation in a radial nerve neurodynamic position: a case report	2016	25		e127â€“		Rot, J and Lempert, C	RAYYAN-INCLUSION: {"Vickie"=>"Excluded", "jsong6"=>"Excluded"} | RAYYAN-EXCLUSION-REASONS: irrelevant
rayyan-283776298	Efficacy of Lumbar Stabilization and Graded Activity Exercises on Biochemical Mediators of Pain in Patients with Non-Specific Chronic Low Back Pain	2019					PACTR201904487417085,	RAYYAN-INCLUSION: {"Vickie"=>"Excluded", "jsong6"=>"Excluded"} | RAYYAN-EXCLUSION-REASONS: irrelevant
rayyan-283776299	exercise-induced muscle damage; prophylactic and therapeutic countermeasures	2019					IRCT20181228042154N1,	RAYYAN-INCLUSION: {"Vickie"=>"Excluded", "jsong6"=>"Excluded"} | RAYYAN-EXCLUSION-REASONS: irrelevant
rayyan-283776300	Efficiency of Thoracolumbar Interfascial Plane Block in Lumbar Disk Surgery	2017					NCT03180099,	RAYYAN-INCLUSION: {"Vickie"=>"Excluded", "jsong6"=>"Excluded"} | RAYYAN-EXCLUSION-REASONS: irrelevant
rayyan-283776301	Safety and tolerability of arimoclomol in patients with sporadic inclusion body myositis: a randomised, double-blind, placebo-controlled, phase IIa proof-of-concept trial	2013	72				Machado. P and Miller. A and Herbelin. L and He. J and Noel. J and Wang. Y and McVey. AL and Pasnoor. M and Gallagher. P and Statland. J and Bradv. S and Lu. C-H and Kalmar. B and Sethi. I	RAYYAN-INCLUSION: {"Vickie"=>"Excluded", "jsong6"=>"Excluded"} | RAYYAN-EXCLUSION-REASONS: irrelevant
rayyan-283776302	Neuromuscular Electrical Stimulation and Isokinetic Training	2007					NCT00512018,	RAYYAN-INCLUSION: {"Vickie"=>"Excluded", "jsong6"=>"Excluded"} | RAYYAN-EXCLUSION-REASONS: irrelevant
rayyan-283776303	Comparing Buccal Infiltration Technique to Inferior Alveolar Nerve Block Technique for Alleviation of Pain During Pulpal Treatment of Second Mandibular Deciduous Molars: (Randomized Control Clinical Trial)	2021					NCT04812639,	RAYYAN-INCLUSION: {"Vickie"=>"Excluded", "jsong6"=>"Excluded"} | RAYYAN-EXCLUSION-REASONS: irrelevant
rayyan-283776304	Deep transcranial magnetic stimulation promotes weight loss and thermoregulation in obesity through neuroendocrine modulation	2018	39	2			Luzi, L and Ferrulli, A and Redaelli, V and Adamo, M and Costa, E and Luzi, F	RAYYAN-INCLUSION: {"Vickie"=>"Excluded", "jsong6"=>"Excluded"} | RAYYAN-EXCLUSION-REASONS: acute,irrelevant
rayyan-283776305	A prospective study of glove perforation in posterior lumbar interbody spinal fusion: what are the factors to watch out?	2016	25		S331		Kang, MS and Kim, TH and Lee, SH and Hwang, JH	RAYYAN-INCLUSION: {"Vickie"=>"Excluded", "jsong6"=>"Excluded"} | RAYYAN-EXCLUSION-REASONS: irrelevant
rayyan-283776306	The effect of remote ischemic preconditioning in prevention of delirium in patient with cardiopulmonary bypass	2017					IRCT2017041712830N21,	RAYYAN-INCLUSION: {"Vickie"=>"Excluded", "jsong6"=>"Excluded"} | RAYYAN-EXCLUSION-REASONS: acute,irrelevant
rayyan-283776307	The Effect of Scapulothoracic Mobilization on Cervical Pain and Range of Motion in Patients With Neck Pain and Scapular Dyskinesia	2017					NCT03046160,	RAYYAN-INCLUSION: {"Vickie"=>"Excluded", "jsong6"=>"Excluded"} | RAYYAN-EXCLUSION-REASONS: irrelevant
rayyan-283776308	The Effect of Transcranial Direct Current Stimulation of the Somatosensory Area on Upper Limb Motor Performance	2020					NCT04618614,	RAYYAN-INCLUSION: {"Vickie"=>"Excluded", "jsong6"=>"Excluded"} | RAYYAN-EXCLUSION-REASONS: irrelevant
rayyan-283776309	Using transcranial direct current stimulation to boost the hypoalgesic effects of exercise:: A randomised controlled experimental study	2019					ACTRN12619000667112,	RAYYAN-INCLUSION: {"Vickie"=>"Excluded", "jsong6"=>"Excluded"} | RAYYAN-EXCLUSION-REASONS: irrelevant
rayyan-283776310	The Effect of Foot Reflexology on Stress, Anxiety, Depression and Serum Cortisol levels in Hemodialysis Patients	2018					IRCT20180304038936N2,	RAYYAN-INCLUSION: {"Vickie"=>"Excluded", "jsong6"=>"Excluded"} | RAYYAN-EXCLUSION-REASONS: irrelevant
rayyan-283776311	Repetitive transcranial magnetic stimulation in preventing the transition from acute to sustained pain: A randomised controlled trial	2018					ACTRN12618001366246,	RAYYAN-INCLUSION: {"Vickie"=>"Excluded", "jsong6"=>"Excluded"} | RAYYAN-EXCLUSION-REASONS: acute,irrelevant
rayyan-283776312	The effect of rhythmic breathing exercise on nausea and vomiting due to chemotherapy in breast cancer patients	2020					IRCT20200521047526N1,	RAYYAN-INCLUSION: {"Vickie"=>"Excluded", "jsong6"=>"Excluded"} | RAYYAN-EXCLUSION-REASONS: irrelevant
rayyan-283776313	Assessing the effectiveness of touch intervention for pain control during examination for retinopathy of prematurity	2020					ISRCTN10976481,	RAYYAN-INCLUSION: {"Vickie"=>"Excluded", "jsong6"=>"Excluded"} | RAYYAN-EXCLUSION-REASONS: irrelevant
rayyan-283776314	SOS Versus Awake Fiberoptic Intubation	2017					NCT03075033,	RAYYAN-INCLUSION: {"Vickie"=>"Excluded", "jsong6"=>"Excluded"} | RAYYAN-EXCLUSION-REASONS: irrelevant
rayyan-283776315	Survey the Effect of Foot Reflexology on Physiological Indices, Pain Level and Crying of Infants under Vaccination	2019					IRCT20130412012990N10,	RAYYAN-INCLUSION: {"Vickie"=>"Excluded", "jsong6"=>"Excluded"} | RAYYAN-EXCLUSION-REASONS: wrong study design

rayyan-283776316	Effect of thoracic spine manipulation on pulmonary function in swimmers with asthma â€“ a study protocol of a randomized controlled crossover trial	2021				ACTRN12621001636842,	RAYYAN-INCLUSION: {"Vickie"=>"Excluded", "jsong6"=>"Excluded"} | RAYYAN-EXCLUSION-REASONS: irrelevant
rayyan-283776317	Efficacy of Ambrisentan in Limited Scleroderma Patients in Improving Blood Flow to Hands or Feet	2010				NCT01072669,	RAYYAN-INCLUSION: {"Vickie"=>"Excluded", "jsong6"=>"Excluded"} | RAYYAN-EXCLUSION-REASONS: irrelevant
rayyan-283776318	Transcranial Direct Current Stimulation (tDCS) and Spaced Aphasia Treatment	2019				NCT04142866,	RAYYAN-INCLUSION: {"Vickie"=>"Excluded", "jsong6"=>"Excluded"} | RAYYAN-EXCLUSION-REASONS: irrelevant
rayyan-283776319	The effect of cervical manual traction on the length of the cervical spine in asymptomatic individuals	2010				ACTRN12610000305022,	RAYYAN-INCLUSION: {"Vickie"=>"Excluded", "jsong6"=>"Excluded"} | RAYYAN-EXCLUSION-REASONS: irrelevant
rayyan-283776320	Efficacy and Safety of Somatropin in Combination with Leuprorelin Compared to Somatropin Alone and to an Untreated Control Group in Pubertal Children with Idiopathic Short Stature - PHOENIX	2006				EUCTR2005-001750-25-NL,	RAYYAN-INCLUSION: {"Vickie"=>"Excluded", "jsong6"=>"Excluded"} | RAYYAN-EXCLUSION-REASONS: irrelevant
rayyan-283776321	The effect of abdominal massage applied on term newborns receiving phototherapy on transcutaneous bilirubin level	2019				ISRCTN27769096,	RAYYAN-INCLUSION: {"Vickie"=>"Excluded", "jsong6"=>"Excluded"} | RAYYAN-EXCLUSION-REASONS: irrelevant
rayyan-283776322	Naps show no enhancing effects on movement performance in healthy young adults	2015	101		eS99	Backhaus, W and Gerloff, C and Hummel, F	RAYYAN-INCLUSION: {"Vickie"=>"Excluded", "jsong6"=>"Excluded"} | RAYYAN-EXCLUSION-REASONS: irrelevant
rayyan-283776323	A clinical trial for Botox as treatment for provoked vestibulodynia	2016				EUCTR2016-000375-25-SE,	RAYYAN-INCLUSION: {"Vickie"=>"Excluded", "jsong6"=>"Excluded"} | RAYYAN-EXCLUSION-REASONS: irrelevant
rayyan-283776324	Do ankle braces affect functional performance?	2020				ACTRN12620000128998,	RAYYAN-INCLUSION: {"Vickie"=>"Excluded", "jsong6"=>"Excluded"} | RAYYAN-EXCLUSION-REASONS: acute,irrelevant
rayyan-283776325	Lateralized cortical/cognitive processing and bilateral electrodermal activity: effects of sensory mode and sex	1982	15	3	191â€“201	Smith, BD and Ketterer, MW	RAYYAN-INCLUSION: {"Vickie"=>"Excluded", "jsong6"=>"Excluded"} | RAYYAN-EXCLUSION-REASONS: irrelevant
rayyan-283776326	Lateralized ultradian rhythms in time and space: a chronobiological field study in Kenyan Maasai	1999	31	2	233â€“243	Meier-Koll, A	RAYYAN-INCLUSION: {"Vickie"=>"Excluded", "jsong6"=>"Excluded"} | RAYYAN-EXCLUSION-REASONS: irrelevant
rayyan-283776327	Flanker interference effects in a line bisection task	2014	232	4	1327â€“1334	Chieffi, S and Iachini, T and Iavarone, A and Messina, G and Viggiano, A and Monda, M	RAYYAN-INCLUSION: {"Vickie"=>"Excluded", "jsong6"=>"Excluded"} | RAYYAN-EXCLUSION-REASONS: irrelevant
rayyan-283776328	Maximizing performance feedback effectiveness through videotape replay and a self-controlled learning environment	1997	68	4	269â€“279	Janelle, CM and Barba, DA and Frehlich, SG and Tennant, LK and Cauraugh, JH	RAYYAN-INCLUSION: {"Vickie"=>"Excluded", "jsong6"=>"Excluded"} | RAYYAN-EXCLUSION-REASONS: irrelevant
rayyan-283776329	Amantadine hydrochloride treatment in heredodegenerative ataxias: a double blind study	1996	61	3	259â€“264	Botez, MI and Botez-Marquard, T and Elie, R and Pedraza, OL and Goyette, K and Lalonde, R	RAYYAN-INCLUSION: {"Vickie"=>"Excluded", "jsong6"=>"Excluded"} | RAYYAN-EXCLUSION-REASONS: irrelevant
rayyan-283776330	Which factors affect hand selection in children's grasping in hemispace? Combined effects of task demand and motor dominance	2006	60	1	88â€“93	Leconte, P and Fagard, J	RAYYAN-INCLUSION: {"Vickie"=>"Excluded", "jsong6"=>"Excluded"} | RAYYAN-EXCLUSION-REASONS: irrelevant
rayyan-283776331	Capsaicin-induced impairment of tactile spatial discrimination ability in man: indirect evidence for increased receptive fields in human nervous system	1998	797	2	361â€“367	Kauppila, T and Mohammadian, P and Nielsen, J and Andersen, OK and Arendt-Nielsen, L	RAYYAN-INCLUSION: {"Vickie"=>"Excluded", "jsong6"=>"Excluded"} | RAYYAN-EXCLUSION-REASONS: irrelevant
rayyan-283776332	Finding if there is any similarity between the personality and handwriting such as open people have a large size of letter or reserved person has a small size of letter this is to find is there are other features that can be c	2020				CTRI/2020/08/027150,	RAYYAN-INCLUSION: {"Vickie"=>"Excluded", "jsong6"=>"Excluded"} | RAYYAN-EXCLUSION-REASONS: irrelevant,wrong study design
rayyan-283776333	Ultrasound biomicroscopy of sclerotomy sites: the effect of vitreous shaving around sclerotomy sites during pars plana vitrectomy	2001	21	5	464â€“468	Sabti, K and Kapusta, M and Mansour, M and Overbury, O and Chow, D	RAYYAN-INCLUSION: {"Vickie"=>"Excluded", "jsong6"=>"Excluded"} | RAYYAN-EXCLUSION-REASONS: irrelevant
rayyan-283776334	The effects of emotion and ocular dominance on lateral eye movement	1988	26	2	213â€“220	Borod, JC and Vingiano, W and Cytryn, F	RAYYAN-INCLUSION: {"Vickie"=>"Excluded", "jsong6"=>"Excluded"} | RAYYAN-EXCLUSION-REASONS: irrelevant
rayyan-283776335	Judgements about numerosity by a commissurotomized subject	1992	30	10	865â€“876	Corballis, MC and Sergentt, J	RAYYAN-INCLUSION: {"Vickie"=>"Excluded", "jsong6"=>"Excluded"} | RAYYAN-EXCLUSION-REASONS: irrelevant,wrong study design
rayyan-283776336	The effect of magnetic therapy on the incidence of post-operative acute urinary retention in patients undergoing surgery in hospital	2021				IRCT20210415050985N1,	RAYYAN-INCLUSION: {"Vickie"=>"Excluded", "jsong6"=>"Excluded"} | RAYYAN-EXCLUSION-REASONS: acute,irrelevant
rayyan-283776337	Clinical utility of extracorporeal shock wave therapy on hypertrophic scars of the hand caused by burn injury: a prospective, randomized, double-blinded study	2020	9	5		Joo, SY and Lee, SY and Cho, YS and Seo, CH	RAYYAN-INCLUSION: {"Vickie"=>"Excluded", "jsong6"=>"Excluded"} | RAYYAN-EXCLUSION-REASONS: irrelevant
rayyan-283776338	Improving accuracy and knot-strength in a robotic surgical task in surgical residents Ã¢â‚¬â€œ A single session tDCS study	2020	131	4	e67â€“e68	Patel, R and Suwa, Y and Kinross, J and von Roon, A and Woods, A and Darzi, A and Singh, H and Leff, D	RAYYAN-INCLUSION: {"Vickie"=>"Excluded", "jsong6"=>"Excluded"} | RAYYAN-EXCLUSION-REASONS: irrelevant
rayyan-283776339	Investigating the feasibility of inducing a pointless habit using mobile app technology, as a novel interventional component of habit reversal therapy (HRT) for treating obsessive compulsive disorder (OCD)	2021				ISRCTN69935306,	RAYYAN-INCLUSION: {"Vickie"=>"Excluded", "jsong6"=>"Excluded"} | RAYYAN-EXCLUSION-REASONS: irrelevant
rayyan-283776340	A Phase 3, Randomized, Double-blind, Placebo and Adalimumab-controlled Study to Evaluate the Efficacy and Safety of Filgotinib in Subjects with Active Psoriatic Arthritis Who Are NaÃƒÂ¯ve to Biologic DMARD Therap	2020				EUCTR2019-001996-35-HU,	RAYYAN-INCLUSION: {"Vickie"=>"Excluded", "jsong6"=>"Excluded"} | RAYYAN-EXCLUSION-REASONS: irrelevant
rayyan-283776341	Endocrine and metabolic effects of long-term administration of [Nle27]growth hormone-releasing hormone-(1-29)-NH2 in age-advanced men and women	1997	82	5	1472â€“1479	Khorram, O and Laughlin, GA and Yen, SS	RAYYAN-INCLUSION: {"Vickie"=>"Excluded", "jsong6"=>"Excluded"} | RAYYAN-EXCLUSION-REASONS: acute,irrelevant
rayyan-283776343	Symptom Improvement Evaluation and Metabolic control among Adult Subjects with Symptomatic Hypoparathyroidism Treated with Recombinant Human Parathyroid hormone [rhPTH(1-84)]	2017				EUCTR2017-000284-32-DK,	RAYYAN-INCLUSION: {"Vickie"=>"Excluded", "jsong6"=>"Excluded"} | RAYYAN-EXCLUSION-REASONS: irrelevant
rayyan-283776344	Characteristics of improved needle-scalpel therapy versus conventional therapy for lumbar disc herniation	2005	9	26	15â€“17	Liu, Q-G and Dai, A-L and He, H-Q and Li, Z-X and Yang, K and Huang, M-H	RAYYAN-INCLUSION: {"Vickie"=>"Excluded", "jsong6"=>"Excluded"} | RAYYAN-EXCLUSION-REASONS: irrelevant
rayyan-283776345	Effects of a single session of action-observation-execution and accelerated skill acquisition program on corticospinal excitability and reach to grasp in individuals with subacute stroke	2019				TCTR20190904004,	RAYYAN-INCLUSION: {"Vickie"=>"Excluded", "jsong6"=>"Excluded"} | RAYYAN-EXCLUSION-REASONS: irrelevant
rayyan-283776346	Effect of upper extremity proprioceptive neuromuscular facilitation combined with elastic resistance bands on respiratory muscle strength: a randomized controlled trial	2013	17	6	541â€“546	Areas, GP and Borghi-Silva, A and Lobato, AN and Silva, AA and Freire, RC and Areas, FZ	RAYYAN-INCLUSION: {"Vickie"=>"Excluded", "jsong6"=>"Excluded"} | RAYYAN-EXCLUSION-REASONS: irrelevant
rayyan-283776347	Virtual reality and haptics as a training device for movement rehabilitation after stroke: a single-case study	2004	85	8	1247â€“1250	Broeren, J and Rydmark, M and Sunnerhagen, KS	RAYYAN-INCLUSION: {"Vickie"=>"Excluded", "jsong6"=>"Excluded"} | RAYYAN-EXCLUSION-REASONS: irrelevant
rayyan-283776348	Supplementation of Kaempferia parviflora Extract Enhances Physical Fitness and Modulates Parameters of Heart Rate Variability in Adolescent Student-Athletes: a Randomized, Double-Blind, Placebo-Controlled Clinica	2020			1â€“8	Sripanidkulchai, B and Promthep, K and Tuntiyasawasdikul, S and Tabboon, P and Areemit, R	RAYYAN-INCLUSION: {"Vickie"=>"Excluded", "jsong6"=>"Excluded"} | RAYYAN-EXCLUSION-REASONS: irrelevant
rayyan-283776349	Effects of mental practice on muscle strength in task-oriented movement: a randomized controlled trial	2011	97		eS616â€•	Kitaura, M and Fukui, S and Matsuo, A and Shomoto, K	RAYYAN-INCLUSION: {"Vickie"=>"Excluded", "jsong6"=>"Excluded"} | RAYYAN-EXCLUSION-REASONS: irrelevant
rayyan-283776350	Electroacupuncture-induced plasticity between hand and face representations in the motor cortex in healthy subjects	2020	45	10	829â€“834	Zhong, J-M and Huang, J-P and Jiang, L and Liu, J-H	RAYYAN-INCLUSION: {"Vickie"=>"Excluded", "jsong6"=>"Excluded"} | RAYYAN-EXCLUSION-REASONS: irrelevant,wrong study design
rayyan-283776351	A Virtual, Randomized, Control Trial of a Digital Therapeutic for Speech, Language, and Cognitive Intervention in Post-stroke Persons With Aphasia	2021	12			Braley, M and Pierce, JS and Saxena, S and De Oliveira, E and Taraboanta, L and Anantha, V and Lakhan, SE and Kiran, S	RAYYAN-INCLUSION: {"Vickie"=>"Excluded", "jsong6"=>"Excluded"} | RAYYAN-EXCLUSION-REASONS: irrelevant
rayyan-283776352	Muscle force during a steering response driving task	2012	45		S497â€•	Pandis, P and Prinold, J and Southgate, DFL and Hill, AM and Bull, AMJ	RAYYAN-INCLUSION: {"Vickie"=>"Excluded", "jsong6"=>"Excluded"} | RAYYAN-EXCLUSION-REASONS: irrelevant
rayyan-283776353	Effects of fatigue on forearm supination	2016	34			Brown, BT and Schmidt, CC and Madonna, TJ and Sarmast, Z and Miller, MC	RAYYAN-INCLUSION: {"Vickie"=>"Excluded", "jsong6"=>"Excluded"} | RAYYAN-EXCLUSION-REASONS: irrelevant
rayyan-283776354	Tool usage in open surgery video data	2020	15	1	S66â€“S67	Goldbraikh, A and D'Angelo, AL and Pugh, C and Laufer, S	RAYYAN-INCLUSION: {"Vickie"=>"Excluded", "jsong6"=>"Excluded"} | RAYYAN-EXCLUSION-REASONS: irrelevant
rayyan-283776355	Map-hand-a reliable and valid tool measuring activity performance in patients with hand osteoarthritis	2018	26		S242â€•	Tveter, A and Nossum, R and Mehl Eide, R and Klokkeide, A and Matre, K and Olsen, M and Andreassen, O and Osteras, N and Kjeken, I	RAYYAN-INCLUSION: {"Vickie"=>"Excluded", "jsong6"=>"Excluded"} | RAYYAN-EXCLUSION-REASONS: irrelevant
rayyan-283776356	Clinical study of an anthroposophic treatment strategy for early rheumatoid arthritis, compared to conventional long-term therapy	2016				DRKS00011083,	RAYYAN-INCLUSION: {"Vickie"=>"Excluded", "jsong6"=>"Excluded"} | RAYYAN-EXCLUSION-REASONS: irrelevant
rayyan-283776357	Effects of Exercise on Thoracic Aneurysms	2019				NCT04197648,	RAYYAN-INCLUSION: {"Vickie"=>"Excluded", "jsong6"=>"Excluded"} | RAYYAN-EXCLUSION-REASONS: irrelevant
rayyan-283776358	Measurement of the Hippocampal Theta Rhythm From the Outer Ear Canal	2019				NCT03954483,	RAYYAN-INCLUSION: {"Vickie"=>"Excluded", "jsong6"=>"Excluded"} | RAYYAN-EXCLUSION-REASONS: nutrition,irrelevant
rayyan-283776359	Preventive HIPEC in combination with perioperative FLOT versus FLOT alone for resectable diffuse type gastric and gastroesophageal junction Type II/III adenocarcinoma â€“ The phase III â€œPREVENTâ€•trial of the /	2020				EUCTR2017-003832-35-DE,	RAYYAN-INCLUSION: {"Vickie"=>"Excluded", "jsong6"=>"Excluded"} | RAYYAN-EXCLUSION-REASONS: irrelevant
rayyan-283776360	Remifentanil on Hemodynamic Response to Anesthesia Induction	2016				NCT02763098,	RAYYAN-INCLUSION: {"Vickie"=>"Excluded", "jsong6"=>"Excluded"} | RAYYAN-EXCLUSION-REASONS: irrelevant
rayyan-283776361	Accelerating Motor Learning in Pediatrics	2017				NCT03193580,	RAYYAN-INCLUSION: {"Vickie"=>"Excluded", "jsong6"=>"Excluded"} | RAYYAN-EXCLUSION-REASONS: irrelevant
rayyan-283776362	Conventional open versus laparoscopic surgery for peritoneal dialysis (PD) peritonitis	2009				ISRCTN55802603,	RAYYAN-INCLUSION: {"Vickie"=>"Excluded", "jsong6"=>"Excluded"} | RAYYAN-EXCLUSION-REASONS: irrelevant
rayyan-283776363	Aerobic Exercise on Prenatal Sleep Quality and Maternal-fetal Attachment	2020				NCT04364919,	RAYYAN-INCLUSION: {"Vickie"=>"Excluded", "jsong6"=>"Excluded"} | RAYYAN-EXCLUSION-REASONS: irrelevant
rayyan-283776364	Internet-based study of a supplement combination in hand osteoarthritis	2019				ACTRN12619000835145,	RAYYAN-INCLUSION: {"Vickie"=>"Excluded", "jsong6"=>"Excluded"} | RAYYAN-EXCLUSION-REASONS: irrelevant
rayyan-283776365	The effect of supplementation with a blackcurrant drink containing L-theanine and pine bark extract in supporting cognitive performance in healthy older adults	2021				ACTRN12621001590853,	RAYYAN-INCLUSION: {"Vickie"=>"Excluded", "jsong6"=>"Excluded"} | RAYYAN-EXCLUSION-REASONS: acute,irrelevant
rayyan-283776366	Self Assessment of Fetal Ultrasound Images	2014				NCT02074592,	RAYYAN-INCLUSION: {"Vickie"=>"Excluded", "jsong6"=>"Excluded"} | RAYYAN-EXCLUSION-REASONS: irrelevant
rayyan-283776367	The analgesic efficacy of ultrasound guided transversus abdominis plane (TAP) block after laparoscopic appendicectomy in children: a prospective randomised trial	2008				ACTRN12608000314325,	RAYYAN-INCLUSION: {"Vickie"=>"Excluded", "jsong6"=>"Excluded"} | RAYYAN-EXCLUSION-REASONS: acute,irrelevant
rayyan-283776368	Dental Anxiety in Children and Salivary Cortisol Levels	2020				NCT04338854,	RAYYAN-INCLUSION: {"Vickie"=>"Excluded", "jsong6"=>"Excluded"} | RAYYAN-EXCLUSION-REASONS: irrelevant
rayyan-283776369	Comparison of Two Dosage Strengths of Transdermal Fentanyl Versus Transdermal Buprenorphine and Placebo in Acute Pain Models in Healthy Volunteers	2009				NCT00886002,	RAYYAN-INCLUSION: {"Vickie"=>"Excluded", "jsong6"=>"Excluded"} | RAYYAN-EXCLUSION-REASONS: acute,irrelevant
rayyan-283776370	Using Classification and Regression Tree Analysis to Assess the Construct Validity of the Automated Neuropsychological Assessment Metrics in the Assessment of Cognitive Impairment in SLE Compared to the ACR Neu	2020	72		2564â€“2566	Yuen, K and Beaton, D and Bingham, K and Su, J and Kakvan, M and Diaz-Martinez, JP and Tartaglia, C and Ruttan, L and Wither, J and Anderson, N and Bonilla, D and Choi, M and Fritzler, M a	RAYYAN-INCLUSION: {"Vickie"=>"Excluded", "jsong6"=>"Excluded"} | RAYYAN-EXCLUSION-REASONS: irrelevant,wrong study design
rayyan-283776371	Kinect-based system in occupational therapy for hand motor functional recovery after stroke	2019	4		150â€•	Adomaviciene, A and Grigonyte, A and Juocevicius, A	RAYYAN-INCLUSION: {"Vickie"=>"Excluded", "jsong6"=>"Excluded"} | RAYYAN-EXCLUSION-REASONS: irrelevant
rayyan-283776372	Graded motor imagery based on mirror neuron on rehabilitative training for stroke patients: a BOLD-fMRI study	2016				ChiCTR-IOR-16008137,	RAYYAN-INCLUSION: {"Vickie"=>"Excluded", "jsong6"=>"Excluded"} | RAYYAN-EXCLUSION-REASONS: irrelevant
rayyan-283776373	The impact of gel fingernail polish application on the reduction of bacterial viability following a surgical hand scrub	2021				Anderson, SL and Wisnieski, L and Achilles, SL and Wooton, KE and Shaffer, CL and Hunt, JA	RAYYAN-INCLUSION: {"Vickie"=>"Excluded", "jsong6"=>"Excluded"} | RAYYAN-EXCLUSION-REASONS: irrelevant
rayyan-283776374	Microwave propagation on acupuncture channels	2006	31	1	1â€“2	Krevsky, MA and Zinina, ES and Koshurinov, Y and Ovechkin, AM and Tkachenko, YA and Han, W and Lee, SM and Yoon, G	RAYYAN-INCLUSION: {"Vickie"=>"Excluded", "jsong6"=>"Excluded"} | RAYYAN-EXCLUSION-REASONS: irrelevant
rayyan-283776375	Mental walking through a complex maze influences lateralized ultradian rhythms	1998	87	3	1059â€“1074	Meier-Koll, A and Albrecht, U and Neuschwander, D	RAYYAN-INCLUSION: {"Vickie"=>"Excluded", "jsong6"=>"Excluded"} | RAYYAN-EXCLUSION-REASONS: irrelevant
rayyan-283776376	Acute action of aminophylline in patients with amyotrophic lateral sclerosis	2007	115	5	301â€“305	Berto, MC and Filha, SC and Camelier, A and Rosa, FW and de Souza Bulle Oliveira, A and Jardim, JR	RAYYAN-INCLUSION: {"Vickie"=>"Excluded", "jsong6"=>"Excluded"} | RAYYAN-EXCLUSION-REASONS: irrelevant
rayyan-283776377	Trunk muscle responses following unpredictable loading of an abducted arm	2009	30	2	181â€“186	Mullington, CJ and Klungarvuth, L and Catley, M and McGregor, AH and Strutton, PH	RAYYAN-INCLUSION: {"Vickie"=>"Excluded", "jsong6"=>"Excluded"} | RAYYAN-EXCLUSION-REASONS: irrelevant
rayyan-283776378	Effect of electrical stimulation applied to the skull on response time and cognition of soccer players	2020				RBR-9bjttc,	RAYYAN-INCLUSION: {"Vickie"=>"Excluded", "jsong6"=>"Excluded"} | RAYYAN-EXCLUSION-REASONS: irrelevant
rayyan-283776379	3D or 2D Upper Extremity Exergaming for Persons With Parkinson's Disease	2018				NCT03515746,	RAYYAN-INCLUSION: {"Vickie"=>"Excluded", "jsong6"=>"Excluded"} | RAYYAN-EXCLUSION-REASONS: irrelevant
rayyan-283776380	Global topological dominance in the left hemisphere	2007	104	52	21014â€“21019	Wang, B and Zhou, TG and Zhuo, Y and Chen, L	RAYYAN-INCLUSION: {"Vickie"=>"Excluded", "jsong6"=>"Excluded"} | RAYYAN-EXCLUSION-REASONS: irrelevant,no right and left limb training
rayyan-283776381	Cerebral lateralization for the processing of spatial coordinates and categories in left-and right-handers	1995	33	4	421â€“439	LAENG, B and PETERS, M	RAYYAN-INCLUSION: {"Vickie"=>"Excluded", "jsong6"=>"Excluded"} | RAYYAN-EXCLUSION-REASONS: irrelevant
rayyan-283776382	Brain stimulation, aging and cognition: investigating neuroplasticity and cognitive enhancement following brain stimulation in amnestic mild cognitive impairment	2015				ACTRN12615000886583,	RAYYAN-INCLUSION: {"Vickie"=>"Excluded", "jsong6"=>"Excluded"} | RAYYAN-EXCLUSION-REASONS: irrelevant
rayyan-283776383	Compare effect of two exercises on hand grip strength in old age	2020				IRCT20200720048148N1,	RAYYAN-INCLUSION: {"Vickie"=>"Excluded", "jsong6"=>"Excluded"} | RAYYAN-EXCLUSION-REASONS: irrelevant
rayyan-283776384	Dichotic listening using Mandarin CV-words of six plosives and vowel /a/	2020	59	12	941â€“947	Hu, X-J and Lau, C-C	RAYYAN-INCLUSION: {"Vickie"=>"Excluded", "jsong6"=>"Excluded"} | RAYYAN-EXCLUSION-REASONS: irrelevant
rayyan-283776385	Speech-induced changes in corticospinal excitability	1996	40	4	628â€“634	Tokimura, H and Tokimura, Y and Oliviero, A and Asakura, T and Rothwell, JC	RAYYAN-INCLUSION: {"Vickie"=>"Excluded", "jsong6"=>"Excluded"} | RAYYAN-EXCLUSION-REASONS: irrelevant
rayyan-283776386	3D or 2D Upper Extremity Exergaming for Persons With Parkinson's Disease	2018				NCT03515746,	RAYYAN-INCLUSION: {"Vickie"=>"Excluded", "jsong6"=>"Excluded"} | RAYYAN-EXCLUSION-REASONS: irrelevant
rayyan-283776387	The distribution of plaque and gingivitis and the influence of toothbrushing hand in a group of South Wales 11-12 year-old children	1987	14	10	564â€“572	Addy, M and Griffiths, G and Dummer, P and Kingdom, A and Shaw, WC	RAYYAN-INCLUSION: {"Vickie"=>"Excluded", "jsong6"=>"Excluded"} | RAYYAN-EXCLUSION-REASONS: irrelevant
rayyan-283776388	Faulty proprioceptive information disrupts motor imagery: an experimental study	2007	53	1	41â€“45	McCormick, K and Zalucki, N and Hudson, M and Moseley, GL	RAYYAN-INCLUSION: {"Vickie"=>"Excluded", "jsong6"=>"Excluded"} | RAYYAN-EXCLUSION-REASONS: irrelevant
rayyan-283776389	Effect of side and rate of stimulation on cerebral blood flow changes in motor areas during finger movements in humans	1993	13	4	639â€“645	Sabatini, U and Chollet, F and Rascol, O and Celsis, P and Rascol, A and Lenzi, GL and Marc- Vergnes, J-P	RAYYAN-INCLUSION: {"Vickie"=>"Excluded", "jsong6"=>"Excluded"} | RAYYAN-EXCLUSION-REASONS: irrelevant
rayyan-283776390	Effect of the vibration board on the strength of ankle dorsal and plantar flexor muscles: a preliminary randomized controlled study	2006	77	1	10â€“16	Costantino, C and Pogliacomi, F and Soncini, G	RAYYAN-INCLUSION: {"Vickie"=>"Excluded", "jsong6"=>"Excluded"} | RAYYAN-EXCLUSION-REASONS: irrelevant
rayyan-283776391	Modulation of executive motor network after neurofeedback training in Parkinson's disease	2017	32		1054â€•	Smulders, N and Mehler, D and Subramanian, L and Linden, D and Whittaker, J	RAYYAN-INCLUSION: {"Vickie"=>"Excluded", "jsong6"=>"Excluded"} | RAYYAN-EXCLUSION-REASONS: irrelevant
rayyan-283776392	Effects of motion feedback combined with body weight support treadmill training for gait recovery in hemiplegic patients after stroke	2016				ChiCTR-IOR-16008411,	RAYYAN-INCLUSION: {"Vickie"=>"Excluded", "jsong6"=>"Excluded"} | RAYYAN-EXCLUSION-REASONS: irrelevant
rayyan-283776393	Thickness and stiffness changes of the transverse carpal ligament in carpal tunnel syndrome patients	2016	34			Marquardt, TL and Gabra, JN and Evans, PJ and Seitz, WH and Li, Z-M	RAYYAN-INCLUSION: {"Vickie"=>"Excluded", "jsong6"=>"Excluded"} | RAYYAN-EXCLUSION-REASONS: irrelevant
rayyan-283776394	Influence of mirror therapy and motor imagery on intermanual transfer effects in upper-limb prosthesis training of healthy participants: a randomized pre-posttest study	2018	13	10	e0204839	Romkema, S and Bongers, RM and van der Sluis, CK	RAYYAN-INCLUSION: {"Vickie"=>"Excluded", "jsong6"=>"Excluded"} | RAYYAN-EXCLUSION-REASONS: irrelevant
rayyan-283776395	Transcranial direct current stimulation over multiple days enhances motor performance of a grip task	2017	60	5	329â€“333	Fan, J and Voisin, J and Milot, MH and Higgins, J and Boudrias, MH	RAYYAN-INCLUSION: {"Vickie"=>"Excluded", "jsong6"=>"Excluded"} | RAYYAN-EXCLUSION-REASONS: No dom vs. non-dom comparison
rayyan-283776396	Mental practice has influence on limitation of motion and muscle atrophy following immobilisation of the radiocarpal joint - a prospective randomised experimental study	2011	149	3	288â€“295	Einsiedel, T and Herzig, D and GrÃ¶n, G and Mayer, J and Becker, C and Gebhard, F	RAYYAN-INCLUSION: {"Vickie"=>"Excluded", "jsong6"=>"Excluded"} | RAYYAN-EXCLUSION-REASONS: irrelevant
rayyan-283776397	Effects of Bilateral Asymmetrical Limbs Proprioceptive Neuromuscular Facilitation on Multifidus Muscle in Chronic Low Back Pain	2019				NCT04206137,	RAYYAN-INCLUSION: {"Vickie"=>"Excluded", "jsong6"=>"Excluded"} | RAYYAN-EXCLUSION-REASONS: irrelevant
rayyan-283776398	The Effects of Pre-Loaded Betaine Supplementation on Thermoregulation and Exercise Metabolism in the Heat	2020				NCT04585295,	RAYYAN-INCLUSION: {"Vickie"=>"Excluded", "jsong6"=>"Excluded"} | RAYYAN-EXCLUSION-REASONS: irrelevant
rayyan-283776399	Comparative Study Between ICSI Results in Transvaginal Ultrasound Guided Embryo Transfer and Transabdominal Ultrasound Guided Embryo Transfer	2018				NCT03683043,	RAYYAN-INCLUSION: {"Vickie"=>"Excluded", "jsong6"=>"Excluded"} | RAYYAN-EXCLUSION-REASONS: irrelevant
rayyan-283776400	Assessment in Healthy Volunteers of the Analgesic Effect Monitored by the NOL Index During Hypnotherapy Session	2021				NCT04802239,	RAYYAN-INCLUSION: {"Vickie"=>"Excluded", "jsong6"=>"Excluded"} | RAYYAN-EXCLUSION-REASONS: irrelevant
rayyan-283776401	Success Rate of 4 Injection Protocols for Mandibular First Molars With Symptomatic Irreversible Pulpitis	2021				NCT04929522,	RAYYAN-INCLUSION: {"Vickie"=>"Excluded", "jsong6"=>"Excluded"} | RAYYAN-EXCLUSION-REASONS: irrelevant
rayyan-283776402	The Effect of Intermittent Fasting on Acute Subconcussive Head Impacts	2022				NCT05236127,	RAYYAN-INCLUSION: {"Vickie"=>"Excluded", "jsong6"=>"Excluded"} | RAYYAN-EXCLUSION-REASONS: irrelevant
rayyan-283776403	Analgesic Effect of Peripheral Dexmedetomidine	2009				NCT00971178,	RAYYAN-INCLUSION: {"Vickie"=>"Excluded", "jsong6"=>"Excluded"} | RAYYAN-EXCLUSION-REASONS: irrelevant
rayyan-283776404	Cause-effect Relationships Between Brain Networks and Bimanual Coordination in Older Adults	2020				NCT04349137,	RAYYAN-INCLUSION: {"Vickie"=>"Excluded", "jsong6"=>"Excluded"} | RAYYAN-EXCLUSION-REASONS: irrelevant
rayyan-283776405	Effect of Sea Grapes-antioxidants Extract in Obese Men : 4 Weeks Randomized-Double Blind Controlled Trial	2021				NCT05037591,	RAYYAN-INCLUSION: {"Vickie"=>"Excluded", "jsong6"=>"Excluded"} | RAYYAN-EXCLUSION-REASONS: acute,irrelevant
rayyan-283776406	A case of improved psychotic depression and motor symptoms in Parkinson's disease following electroconvulsive therapy (ECT)	2020	35		S474â€•	Ramesh, S and Ratliff, J	RAYYAN-INCLUSION: {"Vickie"=>"Excluded", "jsong6"=>"Excluded"} | RAYYAN-EXCLUSION-REASONS: irrelevant
rayyan-283776407	The effects of 12-week plyometric training program on lower and upper extremity performance in pre-pubertal female volleyball players	2015	26	3	S50â€“S51	Cinar Medeni, O and Er, F and Suveren Erdogan, C and Ipekoglu, UG and Colakoglu, FF and Baltaci, G	RAYYAN-INCLUSION: {"Vickie"=>"Excluded", "jsong6"=>"Excluded"} | RAYYAN-EXCLUSION-REASONS: irrelevant
rayyan-283776408	Both Unopposed and Opposed Judo Tasks are Suitable for Analyzing Changes in Lateral Preference	2019	18	2	295â€“300	Mayo, X and Iglesias-Soler, E and Dopico-Calvo, X	RAYYAN-INCLUSION: {"Vickie"=>"Excluded", "jsong6"=>"Excluded"} | RAYYAN-EXCLUSION-REASONS: irrelevant,wrong study design
rayyan-283776409	Influence of linear and undulating strength periodization on physical fitness, physiological, and performance responses to simulated judo matches	2015	29	2	358â€“367	Franchini, E and Branco, BM and Agostinho, MF and Calmet, M and Candau, R	RAYYAN-INCLUSION: {"Vickie"=>"Excluded", "jsong6"=>"Excluded"} | RAYYAN-EXCLUSION-REASONS: irrelevant
rayyan-283776410	Circadian and seasonal changes in ACTH-induced effects in healthy young men	1983	25	5	657â€“665	Reinberg, A and Touitou, Y and Levi, F and Nicolai, A	RAYYAN-INCLUSION: {"Vickie"=>"Excluded", "jsong6"=>"Excluded"} | RAYYAN-EXCLUSION-REASONS: irrelevant
rayyan-283776411	A randomized paired comparison of photodynamic therapy and topical 5-fluorouracil in the treatment of actinic keratoses	1999	41	3	414â€“418	Kurwa, HA and Yong-Gee, SA and Seed, PT and Markey, AC and Barlow, RJ	RAYYAN-INCLUSION: {"Vickie"=>"Excluded", "jsong6"=>"Excluded"} | RAYYAN-EXCLUSION-REASONS: irrelevant
rayyan-283776412	Non-dominant hand quicker to insert peripheral venous catheters under echographic guidance: a randomised trial	2017	36	5	291â€“296	Durand-Bailloud, L and Aho, LS and Savoldelli, G and Ecarnot, F and Girard, C and Benkhadra, M	RAYYAN-INCLUSION: {"Vickie"=>"Excluded", "jsong6"=>"Excluded"} | RAYYAN-EXCLUSION-REASONS: irrelevant,wrong study design
rayyan-283776413	Recitation Effect of Holy Quran on Intensity of Anxiety and Depression in Patients Undergoing Hemodialysis	2014				IRCT201312108665N2,	RAYYAN-INCLUSION: {"Vickie"=>"Excluded", "jsong6"=>"Excluded"} | RAYYAN-EXCLUSION-REASONS: irrelevant
rayyan-283776414	Topical anesthetics for intravenous insertion in children: a randomized equivalency study	2002	110	4	758â€“761	Kleiber, C and Sorenson, M and Whiteside, K and Gronstal, BA and Tannous, R	RAYYAN-INCLUSION: {"Vickie"=>"Excluded", "jsong6"=>"Excluded"} | RAYYAN-EXCLUSION-REASONS: irrelevant
rayyan-283776415	Oral anabolic steroid increases muscle products in hemodialysis patients	2012				ISRCTN41591818,	RAYYAN-INCLUSION: {"Vickie"=>"Excluded", "jsong6"=>"Excluded"} | RAYYAN-EXCLUSION-REASONS: irrelevant,wrong study design
rayyan-283776416	Hypothalamic digoxin, hemispheric chemical dominance, and chronic bronchitis emphysema	2003	113	9	1241â€“1258	Kurup, RK and Kurup, PA	RAYYAN-INCLUSION: {"Vickie"=>"Excluded", "jsong6"=>"Excluded"} | RAYYAN-EXCLUSION-REASONS: irrelevant
rayyan-283776417	Hypothalamic digoxin, hemispheric chemical dominance, and inflammatory bowel disease	2003	113	9	1221â€“1240	Kurup, RK and Kurup, PA	RAYYAN-INCLUSION: {"Vickie"=>"Excluded", "jsong6"=>"Excluded"} | RAYYAN-EXCLUSION-REASONS: irrelevant
rayyan-283776418	Learning by chronic stroke survivors of 2 sequentially linked actions is preserved in dominant hemisphere strokes but may be impaired in non-dominant hemisphere strokes	2016	11	4	17â€•	Shalabi, K and Graziado, S and Davidson, R and Morgan, G and Eyre, J	RAYYAN-INCLUSION: {"Vickie"=>"Excluded", "jsong6"=>"Excluded"} | RAYYAN-EXCLUSION-REASONS: irrelevant
rayyan-283776419	Improvement in Cognitive functioning in Patients with Schizophrenia after cognitive retraining	2018				CTRI/2018/05/014095,	RAYYAN-INCLUSION: {"Vickie"=>"Excluded", "jsong6"=>"Excluded"} | RAYYAN-EXCLUSION-REASONS: irrelevant
rayyan-283776420	2-parallel groups, Phase 3 study to compare efficacy and safety of masitinib versus placebo in the treatment of primary progressive or relapse-free secondary progressive multiple sclerosis	2016				CTRI/2016/09/007268,	RAYYAN-INCLUSION: {"Vickie"=>"Excluded", "jsong6"=>"Excluded"} | RAYYAN-EXCLUSION-REASONS: irrelevant
rayyan-283776422	Laparoscopic Vaginal Cuff Suturing Proficiency for OBGYN Residents	2017				NCT03360357,	RAYYAN-INCLUSION: {"Vickie"=>"Excluded", "jsong6"=>"Excluded"} | RAYYAN-EXCLUSION-REASONS: irrelevant
rayyan-283776423	The treatment of joint degeneration of the thumb with two different operation techniques	2009				NTR1674,	RAYYAN-INCLUSION: {"Vickie"=>"Excluded", "jsong6"=>"Excluded"} | RAYYAN-EXCLUSION-REASONS: irrelevant
rayyan-283776424	The effect of pressure on DNA deposition by touch	2017				Tobias, SHA and Jacques, GS and Morgan, RM and Meakin, GE	RAYYAN-INCLUSION: {"Vickie"=>"Excluded", "jsong6"=>"Excluded"} | RAYYAN-EXCLUSION-REASONS: irrelevant
rayyan-283776426	Did My Hand Move in a Mirror? Body Ownership Induced by the Mirror Hand Illusion	2021	15			Iida, A and Saito, H and Ota, H	RAYYAN-INCLUSION: {"Vickie"=>"Excluded", "jsong6"=>"Excluded"} | RAYYAN-EXCLUSION-REASONS: irrelevant
rayyan-283776427	Post-operative high intensity interval training in patients undergoing major foregut cancer surgery: A randomised controlled trial	2020				ACTRN12620000315910,	RAYYAN-INCLUSION: {"Vickie"=>"Excluded", "jsong6"=>"Excluded"} | RAYYAN-EXCLUSION-REASONS: acute,irrelevant
rayyan-283776428	Effects of two nights partial sleep deprivation on an evening submaximal weightlifting performance; are 1 h powernaps useful on the day of competition?	2019	36	3	407â€“426	Brotherton, EJ and Moseley, SE and Langan-Evans, C and Pullinger, SA and Robertson, CM and Burniston, JG and Edwards, BJ	RAYYAN-INCLUSION: {"Vickie"=>"Excluded", "jsong6"=>"Excluded"} | RAYYAN-EXCLUSION-REASONS: irrelevant
rayyan-283776429	Effects of multicomponent training on functional capacity, muscle performance and body composition of institutionalized older adults	2021				RBR-5ydpb34,	RAYYAN-INCLUSION: {"Vickie"=>"Excluded", "jsong6"=>"Excluded"} | RAYYAN-EXCLUSION-REASONS: irrelevant
rayyan-283776430	Anodal transcranial direct current stimulation enhances the effects of motor imagery training in a finger tapping task	2016	43	1	113â€“119	Saimpont, A and Mercier, C and Malouin, F and Guillot, A and Collet, C and Doyon, J and Jackson, PL	RAYYAN-INCLUSION: {"Vickie"=>"Excluded", "jsong6"=>"Excluded"} | RAYYAN-EXCLUSION-REASONS: irrelevant
rayyan-283776431	A Trial Comparing Pain During Local Tumesent Injection In Fingers Using Different Syringe-Needle Combinations	2018				NCT03736915,	RAYYAN-INCLUSION: {"Vickie"=>"Excluded", "jsong6"=>"Excluded"} | RAYYAN-EXCLUSION-REASONS: irrelevant
rayyan-283776432	Transcranial Magnetic Stimulation (TMS) in Multiple Sclerosis	2019				NCT04062331,	RAYYAN-INCLUSION: {"Vickie"=>"Excluded", "jsong6"=>"Excluded"} | RAYYAN-EXCLUSION-REASONS: acute,irrelevant
rayyan-283776433	The Canadian Underage Substance Use Prevention Trial	2019				NCT04198974,	RAYYAN-INCLUSION: {"Vickie"=>"Excluded", "jsong6"=>"Excluded"} | RAYYAN-EXCLUSION-REASONS: irrelevant
rayyan-283776434	The Effects of Low-intensity Blood-flow Restricted Exercise on Upper Limb Function Following Spinal Cord Injury	2018				NCT03690700,	RAYYAN-INCLUSION: {"Vickie"=>"Excluded", "jsong6"=>"Excluded"} | RAYYAN-EXCLUSION-REASONS: irrelevant
rayyan-283776435	The Effect of High-flow Nasal Oxygenation to the Saturation During Analgo-sedation in Different ASA Risk Class Patients	2018				NCT03687385,	RAYYAN-INCLUSION: {"Vickie"=>"Excluded", "jsong6"=>"Excluded"} | RAYYAN-EXCLUSION-REASONS: acute,irrelevant
rayyan-283776436	Can High-flow Nasal Oxygenation Improve Oxygen Saturation During Analgo-sedation in Obese Adults?	2018				NCT03687424,	RAYYAN-INCLUSION: {"Vickie"=>"Excluded", "jsong6"=>"Excluded"} | RAYYAN-EXCLUSION-REASONS: irrelevant
rayyan-283776437	Influence of HFNO on Spontaneous Ventilation in Patients of Different ASA Risk During Analgo-sedation for Vitrectomy	2019				NCT04055077,	RAYYAN-INCLUSION: {"Vickie"=>"Excluded", "jsong6"=>"Excluded"} | RAYYAN-EXCLUSION-REASONS: irrelevant
rayyan-283776438	Effect of Tool Design on Hand Pain in Dental Practitioners	2011				NCT01332760,	RAYYAN-INCLUSION: {"Vickie"=>"Excluded", "jsong6"=>"Excluded"} | RAYYAN-EXCLUSION-REASONS: irrelevant
rayyan-283776439	Effect of HFNO on Spontaneous Ventilation in Obese Patients During Analgo-sedation for Vitrectomy	2019				NCT04049240,	RAYYAN-INCLUSION: {"Vickie"=>"Excluded", "jsong6"=>"Excluded"} | RAYYAN-EXCLUSION-REASONS: irrelevant
rayyan-283776440	Meditation and Yoga for Irritable Bowel Syndrome	2020				NCT04302623,	RAYYAN-INCLUSION: {"Vickie"=>"Excluded", "jsong6"=>"Excluded"} | RAYYAN-EXCLUSION-REASONS: irrelevant
rayyan-283776441	The influence that physical exercise Tai-Geiko provides in people with multiple sclerosis regarding muscle strength, balance and performing two tasks at the same time	2018				RBR-4sty47,	RAYYAN-INCLUSION: {"Vickie"=>"Excluded", "jsong6"=>"Excluded"} | RAYYAN-EXCLUSION-REASONS: irrelevant
rayyan-283776442	Control Network Neuromodulation to Enhance Cognitive Training in Complex Traumatic Brain Injury	2022				NCT05236010,	RAYYAN-INCLUSION: {"Vickie"=>"Excluded", "jsong6"=>"Excluded"} | RAYYAN-EXCLUSION-REASONS: irrelevant
rayyan-283776443	Left passive foot movement increases respiratory rate in awake congenital central hypoventilation syndrome patients and controls	2011	183	1		Woo, MS and Woo, MA and Macey, PM and Harper, RM	RAYYAN-INCLUSION: {"Vickie"=>"Excluded", "jsong6"=>"Excluded"} | RAYYAN-EXCLUSION-REASONS: irrelevant
rayyan-283776444	Dietary Nitrate and Nitrite to Increase Nitric Oxide in Patients With Coronary Artery Disease	2003				NCT00069654,	RAYYAN-INCLUSION: {"Vickie"=>"Excluded", "jsong6"=>"Excluded"} | RAYYAN-EXCLUSION-REASONS: irrelevant
rayyan-283776445	Validation of the withings ScanWatch as a wrist-worn reflective pulse oximeter: prospective interventional clinical study	2021	23	4		Kirszenblat, R and Edouard, P	RAYYAN-INCLUSION: {"Vickie"=>"Excluded", "jsong6"=>"Excluded"} | RAYYAN-EXCLUSION-REASONS: irrelevant
rayyan-283776446	Surgical hand disinfection with alcohols at various concentrations: parallel experiments using the new proposed European standards method	1998	19	10	778â€“781	Rotter, ML and Simpson, RA and Koller, W	RAYYAN-INCLUSION: {"Vickie"=>"Excluded", "jsong6"=>"Excluded"} | RAYYAN-EXCLUSION-REASONS: irrelevant
rayyan-283776447	Assessment of the effect of dextromethorphan and ketamine on the acute nociceptive threshold and wind-up of the second pain response in healthy male volunteers	2002	53	6	604â€“612	Hughes, AM and Rhodes, J and Fisher, G and Sellers, M and Growcott, JW	RAYYAN-INCLUSION: {"Vickie"=>"Excluded", "jsong6"=>"Excluded"} | RAYYAN-EXCLUSION-REASONS: irrelevant
rayyan-283776448	The dynamics of the m. extensor digitorum communis cortical representation after motor imagery training sessions using brain-computer interface: controlled study	2018	129		e210â€•	Poydasheva, A and Lyukmanov, R and Chernyavsky, A and Aziatskaya, G and Suponeva, N and Piradov, M	RAYYAN-INCLUSION: {"Vickie"=>"Excluded", "jsong6"=>"Excluded"} | RAYYAN-EXCLUSION-REASONS: irrelevant
rayyan-283776449	Wrist postures in video display terminal operators (VDT) using different keyboards	1997	9	3	255â€“266	Ro, J and Jacobs, K	RAYYAN-INCLUSION: {"Vickie"=>"Excluded", "jsong6"=>"Excluded"} | RAYYAN-EXCLUSION-REASONS: irrelevant
rayyan-283776450	Placement of the left side aed pad is poor: training on the left compared to the right side of a manikin does not improve pad placement	2015	132			Staerk, M and Bodtker, H and Rahbek, S and Vammen, L and Lofgren, B	RAYYAN-INCLUSION: {"Vickie"=>"Excluded", "jsong6"=>"Excluded"} | RAYYAN-EXCLUSION-REASONS: irrelevant
rayyan-283776451	Conformationally selective multidimensional chemical shift ranges in proteins from a PACSY database purged using intrinsic quality criteria	2016	64	2	115â€“130	Fritzsching, KJ and Hong, M and Schmidt-Rohr, K	RAYYAN-INCLUSION: {"Vickie"=>"Excluded", "jsong6"=>"Excluded"} | RAYYAN-EXCLUSION-REASONS: irrelevant
rayyan-283776452	Digitizing tablet and Fahn-Tolosa-Marin tremor ratings have comparable minimum detectable change	2016	31		S315	Elble, RJ	RAYYAN-INCLUSION: {"Vickie"=>"Excluded", "jsong6"=>"Excluded"} | RAYYAN-EXCLUSION-REASONS: irrelevant
rayyan-283776453	The effect of whole body vibration, mental workload and backrest angles on heart rate variability	2017				IRCT2016051527901N1,	RAYYAN-INCLUSION: {"Vickie"=>"Excluded", "jsong6"=>"Excluded"} | RAYYAN-EXCLUSION-REASONS: irrelevant
rayyan-283776454	Assessment of anti-stress, behavioural, and neurophysiological effects of L-theanine: A randomised, double-blind, placebo-controlled, crossover trial	2014				ACTRN12614000826640,	RAYYAN-INCLUSION: {"Vickie"=>"Excluded", "jsong6"=>"Excluded"} | RAYYAN-EXCLUSION-REASONS: irrelevant
rayyan-283776455	A 96-week, prospective, multicenter, randomised, double-blind, placebo-controlled, 2-parallel groups, Phase 3 study to compare efficacy and safety of masitinib 4.5 mg/kg/day versus placebo in the treatment of patie	2011				EUCTR2010-021219-17-SK,	RAYYAN-INCLUSION: {"Vickie"=>"Excluded", "jsong6"=>"Excluded"} | RAYYAN-EXCLUSION-REASONS: irrelevant
rayyan-283776456	Roflumilast TMS-EEG Plasticity	2020				NCT04369547,	RAYYAN-INCLUSION: {"Vickie"=>"Excluded", "jsong6"=>"Excluded"} | RAYYAN-EXCLUSION-REASONS: irrelevant
rayyan-283776457	Resistance and Aerobic Training, Relationship With Birth Weight in Colombian Children	2018				NCT03779737,	RAYYAN-INCLUSION: {"Vickie"=>"Excluded", "jsong6"=>"Excluded"} | RAYYAN-EXCLUSION-REASONS: irrelevant
rayyan-283776458	Cardiovascular Effects of Heated Tobacco Products (HTP)	2020				NCT04550026,	RAYYAN-INCLUSION: {"Vickie"=>"Excluded", "jsong6"=>"Excluded"} | RAYYAN-EXCLUSION-REASONS: irrelevant
rayyan-283776459	UVA-1 for Treatment of Skin Tightening and Improvement of Hand Function in Scleroderma	2021				NCT04875078,	RAYYAN-INCLUSION: {"Vickie"=>"Excluded", "jsong6"=>"Excluded"} | RAYYAN-EXCLUSION-REASONS: irrelevant
rayyan-283776460	Conventional or High Definition tDCS to Enhance Implicit Motor Sequence Learning in Healthy Young Adults?	2020				NCT04600531,	RAYYAN-INCLUSION: {"Vickie"=>"Excluded", "jsong6"=>"Excluded"} | RAYYAN-EXCLUSION-REASONS: irrelevant
rayyan-283776461	Abstract #67: effect of Anodal Transcranial Direct Current Stimulation on Motor Learning in Healthy Individuals	2019	12	2	e24â€•	Kunaratnam, N and Saumur, TM and Swarbrick, D and Kiss, A and Mochizuki, G and Chen, JL	RAYYAN-INCLUSION: {"Vickie"=>"Excluded", "jsong6"=>"Excluded"} | RAYYAN-EXCLUSION-REASONS: irrelevant
rayyan-283776462	Ageing affects accuracy in hand laterality judgement	2016	102		e30â€•	Sapsford, F and Dewhurst, S and Donovan, T	RAYYAN-INCLUSION: {"Vickie"=>"Excluded", "jsong6"=>"Excluded"} | RAYYAN-EXCLUSION-REASONS: irrelevant
rayyan-283776463	Population kinetics of the skin flora on gloved hands following surgical hand disinfection with 3 propanol-based hand rubs: a prospective, randomized, double-blind trial	2007	28	3	346â€“350	Rotter, ML and Kampf, G and Suchomel, M and Kundi, M	RAYYAN-INCLUSION: {"Vickie"=>"Excluded", "jsong6"=>"Excluded"} | RAYYAN-EXCLUSION-REASONS: irrelevant
rayyan-283776464	An 8-week, multi-center, randomized, double-blind, placebo-controlled, parallel group trial of Diclofenac Sodium Gel 1% in patients with primary osteoarthritis of the hand - N/A	2005				EUCTR2004-004776-37-DE,	RAYYAN-INCLUSION: {"Vickie"=>"Excluded", "jsong6"=>"Excluded"} | RAYYAN-EXCLUSION-REASONS: irrelevant
rayyan-283776465	The use of an elastomeric fabric orthosis to optimise upper limb recovery following stroke	2011	97		eS770â€“eS771	Mayston, M and Barron, L	RAYYAN-INCLUSION: {"Vickie"=>"Excluded", "jsong6"=>"Excluded"} | RAYYAN-EXCLUSION-REASONS: acute,irrelevant
rayyan-283776466	Comparative double-blind, placebo-controlled sleep laboratory studies of a combination of lorazepam and diphenhydramine (SM-1014) and its single components	1987	42	6	1037â€“1058	Saletu, B and Grunberger, J and Krupka, M and Schuster, P	RAYYAN-INCLUSION: {"Vickie"=>"Excluded", "jsong6"=>"Excluded"} | RAYYAN-EXCLUSION-REASONS: irrelevant
rayyan-283776467	Can finger pinch strength be used as an alternative to hand grip strength for assessing muscle weakness in haemodialysis patients	2015	30		iii337â€“iii338	El-Kateb, S and Omichi, Y and Srivareerat, M and Davenport, A	RAYYAN-INCLUSION: {"Vickie"=>"Excluded", "jsong6"=>"Excluded"} | RAYYAN-EXCLUSION-REASONS: irrelevant
rayyan-283776468	Using drugs to treat pain in hand arthritis	2012				EUCTR2011-003803-39-GB,	RAYYAN-INCLUSION: {"Vickie"=>"Excluded", "jsong6"=>"Excluded"} | RAYYAN-EXCLUSION-REASONS: irrelevant
rayyan-283776469	CONTRALATERAL EFFECTS OF ECCENTRIC TRAINING ON NEUROMUSCULAR FUNCTION OF THE ELBOW FLEXORS DURING FOUR WEEKS OF IMMOBILISATION	2019				DRKS00017169,	RAYYAN-INCLUSION: {"Vickie"=>"Excluded", "jsong6"=>"Excluded"} | RAYYAN-EXCLUSION-REASONS: wrong study design,no right and left limb training
rayyan-283776470	The Effect of the application of Mustard Seeds on specific points of the ear of patients with back pain	2019				RBR-744pfs,	RAYYAN-INCLUSION: {"Vickie"=>"Excluded", "jsong6"=>"Excluded"} | RAYYAN-EXCLUSION-REASONS: irrelevant
rayyan-283776471	To find out the cognitive changes in obese PCOS women	2021				CTRI/2021/07/034581,	RAYYAN-INCLUSION: {"Vickie"=>"Excluded", "jsong6"=>"Excluded"} | RAYYAN-EXCLUSION-REASONS: irrelevant
rayyan-283776472	Radial access	2016	39	3	S99â€•	Binkert, CA	RAYYAN-INCLUSION: {"Vickie"=>"Excluded", "jsong6"=>"Excluded"} | RAYYAN-EXCLUSION-REASONS: irrelevant
rayyan-283776473	Effects of 4 weeks supplementation with a multi-vitamin/mineral preparation on neurocognitive function in healthy adults	2017				ACTRN12617000305325,	RAYYAN-INCLUSION: {"Vickie"=>"Excluded", "jsong6"=>"Excluded"} | RAYYAN-EXCLUSION-REASONS: irrelevant
rayyan-283776474	Effects of a school-based intervention programme on growth, health and well-being of schoolchildren in three African countires: The KaziAfya project	2018				ISRCTN29534081,	RAYYAN-INCLUSION: {"Vickie"=>"Excluded", "jsong6"=>"Excluded"} | RAYYAN-EXCLUSION-REASONS: irrelevant
rayyan-283776475	The effect of a specific balance-strategy training program in women aged 40-60 years	2007				ACTRN12607000625471,	RAYYAN-INCLUSION: {"Vickie"=>"Excluded", "jsong6"=>"Excluded"} | RAYYAN-EXCLUSION-REASONS: irrelevant
rayyan-283776476	Catheter-associated bloodstream infections (CA-BSI) in adults with cancer: a prospective randomized controlled trial	2017	130			Mollee, P and Okano, S and Looke, D and Kennedy, G and Harper, J and Clouston, J and Van Kuilenburg, R and Geary, A and Joubert, W and Eastgate, M and Jones, M	RAYYAN-INCLUSION: {"Vickie"=>"Excluded", "jsong6"=>"Excluded"} | RAYYAN-EXCLUSION-REASONS: irrelevant
rayyan-283776477	A pilot study of cognitive training with or without tDCS for cognitive deficits in older persons with HIV	2017	10	4	e50â€“e52	Ownby, RL and Rivera, J and Mejia, E and Acevedo, A	RAYYAN-INCLUSION: {"Vickie"=>"Excluded", "jsong6"=>"Excluded"} | RAYYAN-EXCLUSION-REASONS: irrelevant
rayyan-283776478	Assessing subclinical tactual deficits in the hand function of diabetic blind persons at risk for peripheral neuropathy	2007	88	12	1662â€“1672	Travieso, D and Lederman, SJ	RAYYAN-INCLUSION: {"Vickie"=>"Excluded", "jsong6"=>"Excluded"} | RAYYAN-EXCLUSION-REASONS: irrelevant
rayyan-283776479	Efficacy of a Computerized Program of Cognitive Rehabilitation of Attention in People With Acquired Brain Injury (ABI)	2020				NCT04214314,	RAYYAN-INCLUSION: {"Vickie"=>"Excluded", "jsong6"=>"Excluded"} | RAYYAN-EXCLUSION-REASONS: irrelevant
rayyan-283776480	The effects of Muscle Stimulation of the whole body on the activities of walking, exercising and the risk of falling in elderly people who do not exercise	2020				RBR-422x64,	RAYYAN-INCLUSION: {"Vickie"=>"Excluded", "jsong6"=>"Excluded"} | RAYYAN-EXCLUSION-REASONS: irrelevant
rayyan-283776481	Acupressure for Fatigue in Elderly Cancer Patients	2020				NCT04362904,	RAYYAN-INCLUSION: {"Vickie"=>"Excluded", "jsong6"=>"Excluded"} | RAYYAN-EXCLUSION-REASONS: irrelevant
rayyan-283776482	The effect of the FÃ©dÃ©ration Internationale de Football Association (FIFA) 11+ Kids injury prevention program on reducing injury rates in childrenâ€™s soccer	2018				ACTRN12618001117202,	RAYYAN-INCLUSION: {"Vickie"=>"Excluded", "jsong6"=>"Excluded"} | RAYYAN-EXCLUSION-REASONS: irrelevant
rayyan-283776483	Comparation of Physiotherapu Approach Using Weights and Elastic Bands on Respiratory Function	2013				RBR-3ss6zt,	RAYYAN-INCLUSION: {"Vickie"=>"Excluded", "jsong6"=>"Excluded"} | RAYYAN-EXCLUSION-REASONS: irrelevant
rayyan-283776484	Optimising clinical and functional outcomes in older adults with Heart Failure (HF) using the Peripheral Remodeling through Intermittent Muscular Exercise (PRIME) approach: A pilot study	2019				ACTRN12619000253101,	RAYYAN-INCLUSION: {"Vickie"=>"Excluded", "jsong6"=>"Excluded"} | RAYYAN-EXCLUSION-REASONS: irrelevant
rayyan-283776485	Influence of the Autonomic Nervous System in Response to Exercise in Hypertensive Individuals	2020				NCT04371757,	RAYYAN-INCLUSION: {"Vickie"=>"Excluded", "jsong6"=>"Excluded"} | RAYYAN-EXCLUSION-REASONS: irrelevant

rayyan-283776486	Repetitive training of contralateral limb through reconsolidation strengthens motor skills	2022	766				Gyoda, T., Ishida, K., Watanabe, T., Nojima, I.	RAYYAN-INCLUSION: {"Vickie"=>"Excluded", "jsong6"=>"Excluded"} | RAYYAN-EXCLUSION-REASONS: irrelevant,wrong study design
rayyan-283776488	Evaluation of the effectiveness of outdoor fitness equipment intervention in achieving fitness goals for seniors	2021	18	23			Chow, H.-W., Chang, K.-T., Fang, I.-Y.	RAYYAN-INCLUSION: {"Vickie"=>"Excluded", "jsong6"=>"Excluded"} | RAYYAN-EXCLUSION-REASONS: irrelevant
rayyan-283776489	Occupational Performance and Hand Function in People With Parkinson's Disease After Participation in Lee Silverman Voice Treatment (LSVT) BIGÂ®	2021	75	6			Doucet, B.M., Blanchard, M., Bienvenu, F.	RAYYAN-INCLUSION: {"Vickie"=>"Excluded", "jsong6"=>"Excluded"} | RAYYAN-EXCLUSION-REASONS: irrelevant
rayyan-283776490	Determination by priority weight of motoric function in children by age groups	2021	23	3			KeleÅŸ, A., DeliceoÄŸlu, G.	RAYYAN-INCLUSION: {"Vickie"=>"Excluded", "jsong6"=>"Excluded"} | RAYYAN-EXCLUSION-REASONS: irrelevant
rayyan-283776491	What Are Practical Surgical Anatomic Landmarks and Distances from Relevant Neurologic Landmarks in Cadavers for the Posterior Approach in Shoulder Arthroplasty?	2021	479	10			Bahk, M.S., Greiwe, R.M.	RAYYAN-INCLUSION: {"Vickie"=>"Excluded", "jsong6"=>"Excluded"} | RAYYAN-EXCLUSION-REASONS: irrelevant
rayyan-283776492	The effect of therapeutic instrumental music performance method on upper extremity functions in adolescent cerebral palsy	2021	121	5			Dogruoz Karatekin, B., Icagasioglu, A.	RAYYAN-INCLUSION: {"Vickie"=>"Excluded", "jsong6"=>"Excluded"} | RAYYAN-EXCLUSION-REASONS: irrelevant
rayyan-283776493	Case Report: Good Prognosis of Mixed Alien Hand Syndrome by Verbal-Cue Rehabilitation Exercise	2021	12				Qu, K., Gan, L., Jiang, W., Yu, P., Dong, M.	RAYYAN-INCLUSION: {"Vickie"=>"Excluded", "jsong6"=>"Excluded"} | RAYYAN-EXCLUSION-REASONS: irrelevant
rayyan-283776494	Determination of physical profile in elite athletes for the detection and selection of talents in Jiujitsu [DeterminaciÃ³n del perfil fÃsico en deportistas Ã©lite para la detecciÃ³n y selecciÃ³n de talentos en Jiu-Jitsu]	2021	13	3			MejÃa, C., Palacios, J.	RAYYAN-INCLUSION: {"Vickie"=>"Excluded", "jsong6"=>"Excluded"} | RAYYAN-EXCLUSION-REASONS: irrelevant
rayyan-283776495	Preventing a loss of accuracy of the tennis serve under pressure	2021	16				Beckmanni, J., Fimpel, L., Wergin, V.V.	RAYYAN-INCLUSION: {"Vickie"=>"Excluded", "jsong6"=>"Excluded"} | RAYYAN-EXCLUSION-REASONS: irrelevant
rayyan-283776496	Effect of handgrip training in extreme heat on the development of handgrip maximal isometric strength among young males	2021	18	10			BartolomÃ©, I., Siquier-Coll, J., PÃ©rez-Quintero, M., Robles-Gil, M.C., MuÃ±oz, D., Maynar-MariÃ±o, M.	RAYYAN-INCLUSION: {"Vickie"=>"Excluded", "jsong6"=>"Excluded"} | RAYYAN-EXCLUSION-REASONS: No dom vs. non-dom comparison
rayyan-283776497	Dynamics of Ñ[illegible]anges in the power characteristics of the Biceps Brachii Muscle under the influence of different vibration effects [illegible]	2021	20	2			Trepova, A.S., Eremushkin, M.A.	RAYYAN-INCLUSION: {"Vickie"=>"Excluded", "jsong6"=>"Excluded"} | RAYYAN-EXCLUSION-REASONS: irrelevant
rayyan-283776498	Quantitative measurement of upper extremity muscles strength among badminton players through dynamometer	2021	46	2			Younas, A.S., Afzal, W., Mahmood, T., Sharif, F., Mubashir, M.	RAYYAN-INCLUSION: {"Vickie"=>"Excluded", "jsong6"=>"Excluded"} | RAYYAN-EXCLUSION-REASONS: irrelevant
rayyan-283776499	Effectiveness of using active games for strength development in 10-year-old boys at the initial training stage in kyokushin karate [illegible]	2021	21	1			Marchenko, S., Satdyiev, B.	RAYYAN-INCLUSION: {"Vickie"=>"Excluded", "jsong6"=>"Excluded"} | RAYYAN-EXCLUSION-REASONS: irrelevant
rayyan-283776500	Using dual-polarization GPS antenna with optimized adaptive neuro-fuzzy inference system to improve single point positioning accuracy in urban canyons	2021	68	1			Sun, R., Fu, L., Wang, G., Cheng, Q., Hsu, L.-T., Ochieng, W.Y.	RAYYAN-INCLUSION: {"Vickie"=>"Excluded", "jsong6"=>"Excluded"} | RAYYAN-EXCLUSION-REASONS: irrelevant
rayyan-283776501	'Prime time of life', a 12-week home-based online multimodal exercise training and health education programme for middle-aged and older adults in laois	2021	5	1			Cooper, D., Kavanagh, R., Bolton, J., Myers, C., O'Connor, S.	RAYYAN-INCLUSION: {"Vickie"=>"Excluded", "jsong6"=>"Excluded"} | RAYYAN-EXCLUSION-REASONS: irrelevant
rayyan-283776502	University physical education department studentsâ€™ psychomotor functionality and physical activity analysis	2021	2021	8			Pravdov, D.M., Pravdov, M.A., Khromtsov, N.E., Matsko, A.I.	RAYYAN-INCLUSION: {"Vickie"=>"Excluded", "jsong6"=>"Excluded"} | RAYYAN-EXCLUSION-REASONS: irrelevant
rayyan-283776503	Novel insights on the bottomâ€"up rise strength transfer: investigating massed vs. distributed exercise training	2021					Aman, M.S., Hosseinzadeh, M., Nokhodchi, N., Bondi, D., Pietrangelo, T., Sardroodian, M.	RAYYAN-INCLUSION: {"Vickie"=>"Excluded", "jsong6"=>"Excluded"} | RAYYAN-EXCLUSION-REASONS: irrelevant
rayyan-283776504	2D:4D digital length index in ball sports [Ã•dice de longitud digital 2D:4D en deportes de pelota]	2021		39			BermÃºdez, S.R., GarcÃa, A.M., Ayala, C.F., Aguirre Loaiza, H.H.	RAYYAN-INCLUSION: {"Vickie"=>"Excluded", "jsong6"=>"Excluded"} | RAYYAN-EXCLUSION-REASONS: irrelevant
rayyan-283776505	Grip strength-endurance in ambitious and recreational climbers: Does the strength decrement index serve as a feasible measure?	2020	17	24			Labott, B.K., Held, S., Donath, L.	RAYYAN-INCLUSION: {"Vickie"=>"Excluded", "jsong6"=>"Excluded"} | RAYYAN-EXCLUSION-REASONS: irrelevant
rayyan-283776506	Anthropometric and Physical Fitness Components Comparison between High- and Low-Performance Archers	2020	8	2			Lau, J.S., Ghafar, R., Hashim, H.A., Zulkifli, E.Z.	RAYYAN-INCLUSION: {"Vickie"=>"Excluded", "jsong6"=>"Excluded"} | RAYYAN-EXCLUSION-REASONS: irrelevant
rayyan-283776507	Intralobular and Interlobular Parietal Functional Network Correlated to MI-BCI Performance	2020	28	12			Phang, C.-R., Ko, L.-W.	RAYYAN-INCLUSION: {"Vickie"=>"Excluded", "jsong6"=>"Excluded"} | RAYYAN-EXCLUSION-REASONS: irrelevant
rayyan-283776508	Preoperative Transcranial Direct Current Stimulation in Glioma Patients: A Proof of Concept Pilot Study	2020	11				Lang, S., Gan, L.S., McLennan, C., Kirton, A., Monchi, O., Kelly, J.J.P.	RAYYAN-INCLUSION: {"Vickie"=>"Excluded", "jsong6"=>"Excluded"} | RAYYAN-EXCLUSION-REASONS: irrelevant
rayyan-283776509	Pisiformâ€"Hamate Coalition With Entrapment Neuropathy of the Deep Palmar Branch of the Ulnar Nerve	2020	2	5			Endo, F., Tajika, T., Kuboi, T., Chikuda, H.	RAYYAN-INCLUSION: {"Vickie"=>"Excluded", "jsong6"=>"Excluded"} | RAYYAN-EXCLUSION-REASONS: irrelevant
rayyan-283776510	Comparing the hand grip power and creatine kinase levels of U-17 judo national team athletes before and after a 6-week strength training	2020	24	4			Ã‡elik, N.M., Soyal, M.	RAYYAN-INCLUSION: {"Vickie"=>"Excluded", "jsong6"=>"Excluded"} | RAYYAN-EXCLUSION-REASONS: irrelevant,wrong study design
rayyan-283776511	Effects of Atorvastatin on Bone Mass and Muscle Strength in Elderly Patients with Hypercholesterolemia Combined with Low Bone Mass [illegible]	2020	23	21			Wang, X., Du, Y., Xiao, F., Zhang, M., Yu, A., Wu, R., Chen, Y., Chen, H., Cheng, Q.	RAYYAN-INCLUSION: {"Vickie"=>"Excluded", "jsong6"=>"Excluded"} | RAYYAN-EXCLUSION-REASONS: irrelevant
rayyan-283776512	What is the impact of fitness on injury risk during police academy training? A retrospective cohort study	2020	12	1			Tomes, C., Schram, B., Pope, R., Orr, R.	RAYYAN-INCLUSION: {"Vickie"=>"Excluded", "jsong6"=>"Excluded"} | RAYYAN-EXCLUSION-REASONS: irrelevant
rayyan-283776514	Factors affecting compliance with weight-bearing restriction and the amount of weight-bearing in the elderly with femur or pelvic fractures	2020	44	2			Seo, H., Lee, G.J., Shon, H.-C., Kong, H.H., Oh, M., Cho, H., Lee, C.J.	RAYYAN-INCLUSION: {"Vickie"=>"Excluded", "jsong6"=>"Excluded"} | RAYYAN-EXCLUSION-REASONS: irrelevant
rayyan-283776515	Effect of a 16-month exercise training program on functional capacities in a centenarian male master athlete: A case study	2020	15	4			FEDERICI, A., FERRI-MARINI, C., BRANDONI, G., GRASSI, E., VALENTINI, M., LUCERTINI, F.	RAYYAN-INCLUSION: {"Vickie"=>"Excluded", "jsong6"=>"Excluded"} | RAYYAN-EXCLUSION-REASONS: irrelevant
rayyan-283776516	Theoretical and methodological foundations for the use of innovative simulators of locally directed impact during the training process of highly qualified armwrestling athletes	2020	20	6			Kamayev, O., Bezkorovainyi, D., Mazurenko, I., Vlasko, S., Zvyagintseva, I.	RAYYAN-INCLUSION: {"Vickie"=>"Excluded", "jsong6"=>"Excluded"} | RAYYAN-EXCLUSION-REASONS: irrelevant
rayyan-283776517	A proposed training program and its effect on muscle strength responses and some physiological variables for volleyball beginners	2020	11	12			Shalaby, M.N., Fadl, M.A.	RAYYAN-INCLUSION: {"Vickie"=>"Excluded", "jsong6"=>"Excluded"} | RAYYAN-EXCLUSION-REASONS: irrelevant
rayyan-283776518	Cerebral laterality and body composition in judo athletes	2020	22				Akalin, T.C.	RAYYAN-INCLUSION: {"Vickie"=>"Excluded", "jsong6"=>"Excluded"} | RAYYAN-EXCLUSION-REASONS: irrelevant
rayyan-283776519	Topographic Somatosensory Imagery for Real-Time fMRI Brain-Computer Interfacing	2019	13				Kaas, A., Goebel, R., Valente, G., Sorger, B.	RAYYAN-INCLUSION: {"Vickie"=>"Excluded", "jsong6"=>"Excluded"} | RAYYAN-EXCLUSION-REASONS: irrelevant
rayyan-283776520	Restoration of hand function with long-term paired associative stimulation after chronic incomplete tetraplegia: a case study	2019	5	1			Rodionov, A., Savolainen, S., Kirveskari, E., MÃ¤kelÃ¤, J.P., Shulga, A.	RAYYAN-INCLUSION: {"Vickie"=>"Excluded", "jsong6"=>"Excluded"} | RAYYAN-EXCLUSION-REASONS: irrelevant
rayyan-283776521	Effect of telemonitoring versus conventional physiotherapy techniques in improving grip strength among postmenopausal home makers	2019	10	8			Kamalnathan, P., Aishwarya, A.	RAYYAN-INCLUSION: {"Vickie"=>"Excluded", "jsong6"=>"Excluded"} | RAYYAN-EXCLUSION-REASONS: irrelevant
rayyan-283776523	Anatomical shoulder movement strength imbalance among water polo overhead athletes	2019	7	2			Nekooei, P., Tengku-Fadilah, T.K., Amri, S., Baki, R.B., Majlesi, S., Nekouei, P.	RAYYAN-INCLUSION: {"Vickie"=>"Excluded", "jsong6"=>"Excluded"} | RAYYAN-EXCLUSION-REASONS: irrelevant
rayyan-283776524	Motor abilities: Peculiarities of strength effort assessment in boys aged 11-13	2019	19	1			Ivashchenko, O.V., MirosÅ‚awa, C., Nosko, M.O., Malyshev, D.A.	RAYYAN-INCLUSION: {"Vickie"=>"Excluded", "jsong6"=>"Excluded"} | RAYYAN-EXCLUSION-REASONS: irrelevant
rayyan-283776525	Yoga practices as an alternative training for physical fitness in children with visual impairment	2019	36	4			Mohanty, S., Pradhan, B., Hankey, A.	RAYYAN-INCLUSION: {"Vickie"=>"Excluded", "jsong6"=>"Excluded"} | RAYYAN-EXCLUSION-REASONS: irrelevant
rayyan-283776526	Comparative analysis of the physical development of schoolchildren of 7-10 years in urban and rural areas	2019	98	3			Saldan, I.P., Pashkov, A.P., Zhukova, O.V.	RAYYAN-INCLUSION: {"Vickie"=>"Excluded", "jsong6"=>"Excluded"} | RAYYAN-EXCLUSION-REASONS: irrelevant
rayyan-283776527	Comparative analysis of strength preparation indicators of 12-13-year-old junior football players	2018	18	4			Sergey, L., Boris, B., Tatiana, S., Svetlana, S., Alexey, B., Khudyakova, V.	RAYYAN-INCLUSION: {"Vickie"=>"Excluded", "jsong6"=>"Excluded"} | RAYYAN-EXCLUSION-REASONS: irrelevant
rayyan-283776528	Effect of neuromuscular electrical stimulation training on the finger extensor muscles for the contralateral corticospinal tract in normal subjects: A diffusion tensor tractography study	2018	12				Jang, S.H., Seo, Y.S.	RAYYAN-INCLUSION: {"Vickie"=>"Excluded", "jsong6"=>"Excluded"} | RAYYAN-EXCLUSION-REASONS: No dom vs. non-dom comparison
rayyan-283776529	Hand strength and dexterity in patients with prader-willi syndrome: A pilot intervention study	2018	46	11			Hsu, W.-L., Chiu, V.J.-Y., Chang, W.-H., Lin, M.-C., Wei, J.-T., Tzeng, I.-S.	RAYYAN-INCLUSION: {"Vickie"=>"Excluded", "jsong6"=>"Excluded"} | RAYYAN-EXCLUSION-REASONS: irrelevant
rayyan-283776530	Comparative study of handgrip strength in judo and jiu-jitsu athletes [Estudo comparativo da forÃ§a de preensÃ£o manual em atletas de judÃ´ e jiu-jitsu]	2018	14				Silva, V.F., Chaves, L.T., Ferreira, W.C., Fialho, J.V.A.P.	RAYYAN-INCLUSION: {"Vickie"=>"Excluded", "jsong6"=>"Excluded"} | RAYYAN-EXCLUSION-REASONS: irrelevant
rayyan-283776531	Effects of physical, virtual reality-based, and brain exercise on physical, cognition, and preference in older persons: a randomized controlled trial	2018	15	1			Htut, T.Z.C., Hiengkaew, V., Jalayondeja, C., Vongsirinavarat, M.	RAYYAN-INCLUSION: {"Vickie"=>"Excluded", "jsong6"=>"Excluded"} | RAYYAN-EXCLUSION-REASONS: irrelevant
rayyan-283776532	Non-invasive activation of cervical spinal networks after severe paralysis	2018	35	18			Gad, P., Lee, S., Terrafranca, N., Zhong, H., Turner, A., Gerasimenko, Y., Edgerton, V.R.	RAYYAN-INCLUSION: {"Vickie"=>"Excluded", "jsong6"=>"Excluded"} | RAYYAN-EXCLUSION-REASONS: irrelevant
rayyan-283776533	Effects of modern fitness technologies on physical qualities in students with locomotor disorders	2018	69	3			Cherepov, E., Epishev, V., Terekhina, E.	RAYYAN-INCLUSION: {"Vickie"=>"Excluded", "jsong6"=>"Excluded"} | RAYYAN-EXCLUSION-REASONS: irrelevant
rayyan-283776534	Exercise for rheumatoid arthritis of the hand	2018	2018	7			Williams, M.A., Srikesavan, C., Heine, P.J., Bruce, J., Brosseau, L., Hoxey-Thomas, N., Lamb, S.E.	RAYYAN-INCLUSION: {"Vickie"=>"Excluded", "jsong6"=>"Excluded"} | RAYYAN-EXCLUSION-REASONS: irrelevant
rayyan-283776535	BDNF Val66Met polymorphism is associated with altered activity-dependent modulation of short-interval intracortical inhibition in bilateral M1	2018	13	6			Morin-Moncet, O., Latulipe-Loiselle, A., Therrien-Blanchet, J.-M., Theoret, H.	RAYYAN-INCLUSION: {"Vickie"=>"Excluded", "jsong6"=>"Excluded"} | RAYYAN-EXCLUSION-REASONS: irrelevant
rayyan-283776536	Transcutaneous electrical spinal stimulation promotes long-term recovery of upper extremity function in chronic tetraplegia	2018	26	6			Inanici, F., Samejima, S., Gad, P., Edgerton, V.R., Hofstetter, C.P., Moritz, C.T.	RAYYAN-INCLUSION: {"Vickie"=>"Excluded", "jsong6"=>"Excluded"} | RAYYAN-EXCLUSION-REASONS: irrelevant
rayyan-283776538	Effects of Head-Neck Positions on the Hand Grip Strength in Healthy Young Adults: A Cross-Sectional Study	2018	2018				Zafar, H., Alghadir, A., Anwer, S.	RAYYAN-INCLUSION: {"Vickie"=>"Excluded", "jsong6"=>"Excluded"} | RAYYAN-EXCLUSION-REASONS: irrelevant,wrong study design
rayyan-283776539	Physical and strength variables as a predictor of 2000m rowing ergometer performance in elite rowers	2017	17	4			Majumdar, P., Das, A., Mandal, M.	RAYYAN-INCLUSION: {"Vickie"=>"Excluded", "jsong6"=>"Excluded"} | RAYYAN-EXCLUSION-REASONS: irrelevant
rayyan-283776540	Robotic Hand-Assisted Training for Spinal Cord Injury Driven by Myoelectric Pattern Recognition: A Case Report	2017	96	10			Lu, Z., Tong, K.-Y., Shin, H., Stampas, A., Zhou, P.	RAYYAN-INCLUSION: {"Vickie"=>"Excluded", "jsong6"=>"Excluded"} | RAYYAN-EXCLUSION-REASONS: irrelevant
rayyan-283776541	Effects of exercise training experience on hand grip strength, body composition and postural stability in fitness pole dancers	2017	57	9			Nawrocka, A., Mynarski, A., Powerska, A., Rozpara, M., Garbaciak, W.	RAYYAN-INCLUSION: {"Vickie"=>"Excluded", "jsong6"=>"Excluded"} | RAYYAN-EXCLUSION-REASONS: irrelevant
rayyan-283776542	Grip strength and its relationship to police recruit task performance and injury risk: A retrospective cohort study	2017	14	8			Orr, R., Pope, R., Stierli, M., Hinton, B.	RAYYAN-INCLUSION: {"Vickie"=>"Excluded", "jsong6"=>"Excluded"} | RAYYAN-EXCLUSION-REASONS: irrelevant
rayyan-283776543	Advanced myoelectric control for robotic hand-assisted training: Outcome from a stroke patient	2017	8				Lu, Z., Tong, K., Shin, H., Li, S., Zhou, P.	RAYYAN-INCLUSION: {"Vickie"=>"Excluded", "jsong6"=>"Excluded"} | RAYYAN-EXCLUSION-REASONS: irrelevant
rayyan-283776545	Shoulder functional electrical stimulation during wheelchair propulsion in spinal cord injury subjects	2017	23	2			Freixes, O., Fernandez, S.A., Gatti, M.A., Crespo, M.J., Olmos, L.E., Russo, M.J.	RAYYAN-INCLUSION: {"Vickie"=>"Excluded", "jsong6"=>"Excluded"} | RAYYAN-EXCLUSION-REASONS: irrelevant
rayyan-283776546	Anthropometric basis of vertical jump performance: A study in young Indian national players	2017	11	2			Sharma, H.B., Gandhi, S., Meitei, K.K., Dvivedi, J., Dvivedi, S.	RAYYAN-INCLUSION: {"Vickie"=>"Excluded", "jsong6"=>"Excluded"} | RAYYAN-EXCLUSION-REASONS: irrelevant
rayyan-283776548	Effects of hand grip exercise on shoulder joint internal rotation and external rotation peak torque	2016	29	3			Lee, D.-R., Kim, L.J.-S.	RAYYAN-INCLUSION: {"Vickie"=>"Excluded", "jsong6"=>"Excluded"} | RAYYAN-EXCLUSION-REASONS: irrelevant,wrong study design
rayyan-283776549	Formation of professionally important qualities of students with weakened motor fitness using a health related and sport-oriented training program	2016	16	1			Kuzmin, V.A., Kopylov, Y.A., Kudryavtsev, M.D., Tolstopyatov, I.A., Galimov, G.Y., Ionova, O.M.	RAYYAN-INCLUSION: {"Vickie"=>"Excluded", "jsong6"=>"Excluded"} | RAYYAN-EXCLUSION-REASONS: irrelevant
rayyan-283776550	Bilateral asymmetry in left handers increased concerning morphological laterality in a recent sample of young adults	2016	73	4			Sander, M.M., Scheffler, C.	RAYYAN-INCLUSION: {"Vickie"=>"Excluded", "jsong6"=>"Excluded"} | RAYYAN-EXCLUSION-REASONS: irrelevant,no right and left limb training
rayyan-283776551	Preliminary Investigation of an Innovative Digital Motion Analysis Device for Badminton Athlete Performance Evaluation	2016	147				Taha, Z., Hassan, M.S.S., Yap, H.J., Yeo, W.K.	RAYYAN-INCLUSION: {"Vickie"=>"Excluded", "jsong6"=>"Excluded"} | RAYYAN-EXCLUSION-REASONS: irrelevant
rayyan-283776552	Dermatoglyphic profile and hand grip strength of the finalists athletes in the Brazilian paracanoe championship	2016	19	1			De Sousa, A.P.S., Ferreira, H.R., Filho, J.F.	RAYYAN-INCLUSION: {"Vickie"=>"Excluded", "jsong6"=>"Excluded"} | RAYYAN-EXCLUSION-REASONS: irrelevant
rayyan-283776553	Investigating the effects of maximal strength training on wrestlersâ€™ physical, physiological and selected motor skills	2015	20	3			Kaya, I.	RAYYAN-INCLUSION: {"Vickie"=>"Excluded", "jsong6"=>"Excluded"} | RAYYAN-EXCLUSION-REASONS: irrelevant
rayyan-283776554	Mirror illusion reduces motor cortical inhibition in the ipsilateral primary motor cortex during forceful unilateral muscle contractions	2015	113	7			Zult, T., Goodall, S., Thomas, K., HortobÃ¡gyi, T., Howatson, G.	RAYYAN-INCLUSION: {"Vickie"=>"Excluded", "jsong6"=>"Excluded"} | RAYYAN-EXCLUSION-REASONS: irrelevant
rayyan-283776555	Differences in anthropometry, somatotype, body composition and physiological characteristics of female volleyball players by competition level	2015	11	1			Nikolaidis, P.T., Afonso, J., Busko, K.	RAYYAN-INCLUSION: {"Vickie"=>"Excluded", "jsong6"=>"Excluded"} | RAYYAN-EXCLUSION-REASONS: irrelevant
rayyan-283776556	Intensive specific maximal judo drills improve psycho-motor ability but may impair hand grip isometric strength	2015	15	2			Obminski, Z., Litwiniuk, A., Staniak, Z., Zdanowicz, R., Weimo, Z.	RAYYAN-INCLUSION: {"Vickie"=>"Excluded", "jsong6"=>"Excluded"} | RAYYAN-EXCLUSION-REASONS: irrelevant
rayyan-283776557	Hybrid brain-Computer interface and functional electrical stimulation for sensorimotor training in participants with tetraplegia: A Proof-of-Concept Study	2015	39	1			VÇ"ckovÃc, A., Wallace, L., Allan, D.B.	RAYYAN-INCLUSION: {"Vickie"=>"Excluded", "jsong6"=>"Excluded"} | RAYYAN-EXCLUSION-REASONS: irrelevant
rayyan-283776558	Clinical effectiveness of grip strength in predicting ambulation of elderly inpatients	2014	9				Beseler, M.R., Rubio, C., Duarte, E., HervÃ¡s, D., Guevara, M.C., Giner-Pascual, M., Viosca, E.	RAYYAN-INCLUSION: {"Vickie"=>"Excluded", "jsong6"=>"Excluded"} | RAYYAN-EXCLUSION-REASONS: irrelevant
rayyan-283776559	Hamate body and capitate fracture in punch injury	2014	32	10			Goliver, J.A., Adamow, J.S., Goliver, J.	RAYYAN-INCLUSION: {"Vickie"=>"Excluded", "jsong6"=>"Excluded"} | RAYYAN-EXCLUSION-REASONS: irrelevant
rayyan-283776560	From specific training to global shift of manual preference in Kung Fu experts	2014	118	1			Maeda, R.S., Souza, R.M., Teixeira, L.A.	RAYYAN-INCLUSION: {"Vickie"=>"Excluded", "jsong6"=>"Excluded"} | RAYYAN-EXCLUSION-REASONS: irrelevant
rayyan-283776562	Elbow joint fatigue and bench-press training	2014	49	3			Huang, Y.-P., Chou, Y.-L., Chen, F.-C., Wang, R.-T., Huang, M.-J., Chou, P.P.-H.	RAYYAN-INCLUSION: {"Vickie"=>"Excluded", "jsong6"=>"Excluded"} | RAYYAN-EXCLUSION-REASONS: irrelevant
rayyan-283776563	Experimental research on relationship between attention and cortex-muscle interaction	2014	48	6			Zheng, Y., Wang, G., Xu, J., Qin, Y., Wang, J.	RAYYAN-INCLUSION: {"Vickie"=>"Excluded", "jsong6"=>"Excluded"} | RAYYAN-EXCLUSION-REASONS: irrelevant
rayyan-283776565	Physiological characteristics of adaptation at multi-modal approach in organization of physical education process	2014		6			Dyakova, E.Yu., Zakharova, A.N.	RAYYAN-INCLUSION: {"Vickie"=>"Excluded", "jsong6"=>"Excluded"} | RAYYAN-EXCLUSION-REASONS: irrelevant
rayyan-283776567	Contrasting effects of fatigue on multifinger coordination in young and older adults	2013	115	4			Singh, T., Zatsiorsky, V.M., Latash, M.L.	RAYYAN-INCLUSION: {"Vickie"=>"Excluded", "jsong6"=>"Excluded"} | RAYYAN-EXCLUSION-REASONS: irrelevant
rayyan-283776568	Transfer of learning between hands to handle a novel object in old age	2013	227	1			Parikh, P.J., Cole, K.J.	RAYYAN-INCLUSION: {"Vickie"=>"Excluded", "jsong6"=>"Excluded"} | RAYYAN-EXCLUSION-REASONS: irrelevant
rayyan-283776569	Inter-limb transfer of ballistic motor skill following non-dominant limb training in young and older adults	2013	227	1			Hinder, M.R., Carroll, T.J., Summers, J.J.	RAYYAN-INCLUSION: {"Vickie"=>"Excluded", "jsong6"=>"Excluded"} | RAYYAN-EXCLUSION-REASONS: irrelevant,wrong study design,no right and left limb training
rayyan-283776570	Changes in brain activation patterns according to cross-training effect in serial reaction time task: An functional MRI study	2013	8	7			Kwon, Y.H., Kwon, J.W., Park, J.W.	RAYYAN-INCLUSION: {"Vickie"=>"Excluded", "jsong6"=>"Excluded"} | RAYYAN-EXCLUSION-REASONS: irrelevant
rayyan-283776571	Laterality of handgrip strength: Age- and physical training-related changes in Lithuanian schoolchildren and conscripts	2013	1288	1			Tutkuviene, J., SchiefenhÃ¶vel, W.	RAYYAN-INCLUSION: {"Vickie"=>"Excluded", "jsong6"=>"Excluded"} | RAYYAN-EXCLUSION-REASONS: irrelevant
rayyan-283776572	Physiological and technical-tactical analysis in brazilian jiu-jitsu competition	2013	4	2			Andreato, L.V., Franchini, E., De Moraes, S.M.F., PastÃ³rio, J.J., da Silva, D.F., Esteves, J.V.D.C., Branco, B.H.M., Romero, P.V.S., Machado, F.A.	RAYYAN-INCLUSION: {"Vickie"=>"Excluded", "jsong6"=>"Excluded"} | RAYYAN-EXCLUSION-REASONS: irrelevant
rayyan-283776573	Channel selection and classification of electroencephalogram signals: An artificial neural network and genetic algorithm-based approach	2012	55	2			Yang, J., Singh, H., Hines, E.L., Schlaghecken, F., Iliescu, D.D., Leeson, M.S., Stocks, N.G.	RAYYAN-INCLUSION: {"Vickie"=>"Excluded", "jsong6"=>"Excluded"} | RAYYAN-EXCLUSION-REASONS: irrelevant
rayyan-283776574	Isometric force development of some muscle groups in athletes	2012	26	1			Doder, D.V., Babiak, J.J., JanjiÄ‡, N.J., Doder, R.Å½.	RAYYAN-INCLUSION: {"Vickie"=>"Excluded", "jsong6"=>"Excluded"} | RAYYAN-EXCLUSION-REASONS: irrelevant
rayyan-283776575	Saccadic eye movements in a high-speed bimanual stacking task: Changes of attentional control during learning and automatization	2011	11	7			Foerster, R.M., Carbone, E., Koesling, H., Schneider, W.X.	RAYYAN-INCLUSION: {"Vickie"=>"Excluded", "jsong6"=>"Excluded"} | RAYYAN-EXCLUSION-REASONS: irrelevant
rayyan-283776576	Physical and psychological benefits of a 24-week traditional dance program in breast cancer survivors	2011	15	2			Kaltsatou, A., Mameletzi, D., Douka, S.	RAYYAN-INCLUSION: {"Vickie"=>"Excluded", "jsong6"=>"Excluded"} | RAYYAN-EXCLUSION-REASONS: irrelevant
rayyan-283776577	Absence of cross-limb transfer of performance gains following ballistic motor practice in older adults	2011	110	1			Hinder, M.R., Schmidt, M.W., Garry, M.I., Carroll, T.J., Summers, J.J.	RAYYAN-INCLUSION: {"Vickie"=>"Excluded", "jsong6"=>"Excluded"} | RAYYAN-EXCLUSION-REASONS: irrelevant,no right and left limb training
rayyan-283776578	Isolated rupture of the teres major tendon	2010	33	11			Lester, J.D., Boselli, K.J., Kim, P.D., Ahmad, C.S.	RAYYAN-INCLUSION: {"Vickie"=>"Excluded", "jsong6"=>"Excluded"} | RAYYAN-EXCLUSION-REASONS: irrelevant
rayyan-283776579	Assessment of hand grip strength in mexican boxers by training phase	2010	6				GarciÃ¡, C.M.R., Harasymowicz, J., Viramontes, J.A., Ã"rdenes, I.A., VÃ¡zquez, F.B.	RAYYAN-INCLUSION: {"Vickie"=>"Excluded", "jsong6"=>"Excluded"} | RAYYAN-EXCLUSION-REASONS: irrelevant
rayyan-283776581	Comparison of trunk muscle strength of soccer players with and without low back pain [Vergleich der Rumpfmuskelkraft von Fuballspielern mit und ohne RÃ¼ckenschmerzen]	2010	148	4			Maus, U., Kieffer, O., Siebert, C.H., MÃ¼ller-Rath, R., Andereya, S., Eisenhardt, D., Miltner, O.	RAYYAN-INCLUSION: {"Vickie"=>"Excluded", "jsong6"=>"Excluded"} | RAYYAN-EXCLUSION-REASONS: irrelevant
rayyan-283776582	Brain activity preceding a 2D manual catching task	2009	47	4			Tombini, M., Zappasodi, F., Zollo, L., Pellegrino, G., Cavallo, G., Tecchio, F., Guglielmelli, E., Rossini, P.M.	RAYYAN-INCLUSION: {"Vickie"=>"Excluded", "jsong6"=>"Excluded"} | RAYYAN-EXCLUSION-REASONS: irrelevant
rayyan-283776583	Single-trial reconstruction of finger-pinch forces from human motor-cortical activation measured by near-infrared spectroscopy (NIRS)	2009	47	2			Nambu, I., Osu, R., Sato, M.-a., Ando, S., Kawato, M., Naito, E.	RAYYAN-INCLUSION: {"Vickie"=>"Excluded", "jsong6"=>"Excluded"} | RAYYAN-EXCLUSION-REASONS: irrelevant
rayyan-283776585	Motor imagery and action observation: Modulation of sensorimotor brain rhythms during mental control of a brain-computer interface	2009	120	2			Neuper, C., Scherer, R., Wriessnegger, S., Pfurtscheller, G.	RAYYAN-INCLUSION: {"Vickie"=>"Excluded", "jsong6"=>"Excluded"} | RAYYAN-EXCLUSION-REASONS: irrelevant
rayyan-283776586	Ergonomic risk associated with assisting in minimally invasive surgery	2009	23	1			Lee, G., Lee, T., Dexter, D., Godinez, C., Meenaghan, N., Catania, R., Park, A.	RAYYAN-INCLUSION: {"Vickie"=>"Excluded", "jsong6"=>"Excluded"} | RAYYAN-EXCLUSION-REASONS: irrelevant
rayyan-283776587	Indentation in shape memory alloys	2008					Cheng, Y.-T., Grummon, D.S.	RAYYAN-INCLUSION: {"Vickie"=>"Excluded", "jsong6"=>"Excluded"} | RAYYAN-EXCLUSION-REASONS: irrelevant
rayyan-283776588	Effect of vibration training on the glucose kinetics, arterial pressure, and grip strength dynamometry [Efecto de un entrenamiento vibratorio sobre la cinemÃ¡tica de la glucosa, presiÃ³n arterial, y dinamometrÃa manu	2008	25	126			Pardo, E.M., Alcaraz, P.E., Mesa, F., Carrasco, L.	RAYYAN-INCLUSION: {"Vickie"=>"Excluded", "jsong6"=>"Excluded"} | RAYYAN-EXCLUSION-REASONS: irrelevant
rayyan-283776589	Unilateral practice of a ballistic movement causes bilateral increases in performance and corticospinal excitability	2008	104	6			Carroll, T.J., Lee, M., Hsu, M., Sayde, J.	RAYYAN-INCLUSION: {"Vickie"=>"Excluded", "jsong6"=>"Excluded"} | RAYYAN-EXCLUSION-REASONS: irrelevant
rayyan-283776590	The effects of a Simplified Tai-Chi Exercise Program (STEP) on the physical health of older adults living in long-term care facilities: A single group design with multiple time points	2008	45	4			Chen, K.M., Lin, J.-N., Lin, H.-S., Wu, H.-C., Chen, W.-T., Li, C.-H., Kai Lo, S.	RAYYAN-INCLUSION: {"Vickie"=>"Excluded", "jsong6"=>"Excluded"} | RAYYAN-EXCLUSION-REASONS: irrelevant
rayyan-283776591	The effect of resistance training on handgrip strength in young adults	2008	16	2			Thomas, E.M., Sahlberg, M., Svantesson, U.	RAYYAN-INCLUSION: {"Vickie"=>"Excluded", "jsong6"=>"Excluded"} | RAYYAN-EXCLUSION-REASONS: irrelevant
rayyan-283776592	Revisiting an old question with a new technology: Gender differences on a neuro-cognitive temporal tracking task	2007	2				Rice, V.J., Butler, J.	RAYYAN-INCLUSION: {"Vickie"=>"Excluded", "jsong6"=>"Excluded"} | RAYYAN-EXCLUSION-REASONS: irrelevant
rayyan-283776593	Diurnal variation in temperature, mental and physical performance, and tasks specifically related to football (soccer)	2007	24	3			Reilly, T., Atkinson, G., Edwards, B., Waterhouse, J., Farrelly, K., Fairhurst, E.	RAYYAN-INCLUSION: {"Vickie"=>"Excluded", "jsong6"=>"Excluded"} | RAYYAN-EXCLUSION-REASONS: irrelevant
rayyan-283776594	Generalization of motor adaptation skills from bimanual-grasp to individual limbs	2006					Hitchens, J., Patton, J.	RAYYAN-INCLUSION: {"Vickie"=>"Excluded", "jsong6"=>"Excluded"} | RAYYAN-EXCLUSION-REASONS: irrelevant
rayyan-283776595	The Berlin brain-computer interface: EEG-based communication without subject training	2006	14	2			Blankertz, B., Dornhege, G., Krauledat, M., MÃ¼ller, K.-R., Kunzmann, V., Losch, F., Curio, G.	RAYYAN-INCLUSION: {"Vickie"=>"Excluded", "jsong6"=>"Excluded"} | RAYYAN-EXCLUSION-REASONS: irrelevant
rayyan-283776596	Quantifying muscle patterns and spine load during various forms of the push-up	2006	38	3			Freeman, S., Karpowicz, A., Gray, J., McGill, S.	RAYYAN-INCLUSION: {"Vickie"=>"Excluded", "jsong6"=>"Excluded"} | RAYYAN-EXCLUSION-REASONS: irrelevant
rayyan-283776597	Combined functional task practice and dynamic high intensity resistance training promotes recovery of upper-extremity motor function in post-stroke hemiparesis: A case study	2006	30	3			Patten, C., Dozono, J., Schmidt, S.G., Jue, M.E., Lum, P.S.	RAYYAN-INCLUSION: {"Vickie"=>"Excluded", "jsong6"=>"Excluded"} | RAYYAN-EXCLUSION-REASONS: irrelevant
rayyan-283776598	Motor-unit coherence and its relation with synchrony are influenced by training	2004	92	6			Semmler, J.G., Sale, M.V., Meyer, F.G., Nordstrom, M.A.	RAYYAN-INCLUSION: {"Vickie"=>"Excluded", "jsong6"=>"Excluded"} | RAYYAN-EXCLUSION-REASONS: irrelevant
rayyan-283776599	MEG study of short-term plasticity following multiple digit frequency discrimination training in humans	2004	16	4			Liu, L., Ioannides, A.A.	RAYYAN-INCLUSION: {"Vickie"=>"Excluded", "jsong6"=>"Excluded"} | RAYYAN-EXCLUSION-REASONS: irrelevant
rayyan-283776600	Is there training-dependent reorganization of digit representations in area 3b of string players?	2004	115	2			Hashimoto, I., Suzuki, A., Kimura, T., Iguchi, Y., Tanosaki, M., Takino, R., Haruta, Y., Taira, M.	RAYYAN-INCLUSION: {"Vickie"=>"Excluded", "jsong6"=>"Excluded"} | RAYYAN-EXCLUSION-REASONS: irrelevant
rayyan-283776602	Evidence for fusion and segregation induced by 21 Hz multiple-digit stimulation in humans	2000	11	10			Liu, L.C., Gaetz, W.C., Bosnyak, D.J., Roberts, L.E.	RAYYAN-INCLUSION: {"Vickie"=>"Excluded", "jsong6"=>"Excluded"} | RAYYAN-EXCLUSION-REASONS: irrelevant
rayyan-283776603	Yoga training and motor speed based on a finger tapping task	1999	43	4			Dash, M., Telles, S.	RAYYAN-INCLUSION: {"Vickie"=>"Excluded", "jsong6"=>"Excluded"} | RAYYAN-EXCLUSION-REASONS: irrelevant
rayyan-283776604	A case of conversion disorder during nitrous oxide psychosedation	1999	27	3			Satoh, Y., Niwa, H., Hashiguti, K., Tanimoto, A., Nisimura, N., Matsuura, H.	RAYYAN-INCLUSION: {"Vickie"=>"Excluded", "jsong6"=>"Excluded"} | RAYYAN-EXCLUSION-REASONS: irrelevant
rayyan-283776605	Outcome of Functional Electrical Stimulation in the Rehabilitation of a Child with C-5 Tetraplegia	1999	22	2			Davis, S.E., Mulcahey, M.J., Smith, B.T., Betz, R.R.	RAYYAN-INCLUSION: {"Vickie"=>"Excluded", "jsong6"=>"Excluded"} | RAYYAN-EXCLUSION-REASONS: irrelevant
rayyan-283776606	WMSDs: Results of assessment of exposure to biomechanical overloads and clinical testing among female workers manually producing plywood panels [Movimenti ripetitivi degli arti superiori: Risultati della valutazion	1998	89	5			Zecchi, G., Venturi, G.	RAYYAN-INCLUSION: {"Vickie"=>"Excluded", "jsong6"=>"Excluded"} | RAYYAN-EXCLUSION-REASONS: irrelevant
rayyan-283776607	Muscle power, dexterity skill and visual perception in community home girls trained in yoga or sports and in regular school girls	1997	41	4			Raghuraj, P., Telles, S.	RAYYAN-INCLUSION: {"Vickie"=>"Excluded", "jsong6"=>"Excluded"} | RAYYAN-EXCLUSION-REASONS: irrelevant
rayyan-283776609	Motor unit recruitment in a distributed model of extraocular muscle	1996	76	2			Dean, P.	RAYYAN-INCLUSION: {"Vickie"=>"Excluded", "jsong6"=>"Excluded"} | RAYYAN-EXCLUSION-REASONS: irrelevant
rayyan-283776610	Anthropometric, strength, endurance and flexibility characteristics of elite and recreational climbers	1996	14	4			Grant, S., Hynes, V., Whittaker, A., Aitchison, T.	RAYYAN-INCLUSION: {"Vickie"=>"Excluded", "jsong6"=>"Excluded"} | RAYYAN-EXCLUSION-REASONS: irrelevant
rayyan-283776611	Effect of regular training on the anthropometric parameters and urine steroids in childhood	1994	68	4			Juricskay, Z., Mezey, B.	RAYYAN-INCLUSION: {"Vickie"=>"Excluded", "jsong6"=>"Excluded"} | RAYYAN-EXCLUSION-REASONS: irrelevant
rayyan-283776612	Effects of 10 and 20 days bed rest on leg muscle mass and strength in young subjects	1994	150	616			Suzuki, Y., Murakami, T., Haruna, Y., Kawakubo, K., Goto, S., Makita, Y., Ikawa, S., Gunji, A.	RAYYAN-INCLUSION: {"Vickie"=>"Excluded", "jsong6"=>"Excluded"} | RAYYAN-EXCLUSION-REASONS: irrelevant
rayyan-283776613	Effects of sustained manual work and partial sleep deprivation on muscular strength and endurance	1987	56	1			Legg, S.J., Patton, J.F.	RAYYAN-INCLUSION: {"Vickie"=>"Excluded", "jsong6"=>"Excluded"} | RAYYAN-EXCLUSION-REASONS: irrelevant
rayyan-283776614	Biofeedback training for the treatment of chronic pain in an elderly arthritic female	1983	2	3			Boczkowski, J.A.	RAYYAN-INCLUSION: {"Vickie"=>"Excluded", "jsong6"=>"Excluded"} | RAYYAN-EXCLUSION-REASONS: irrelevant
rayyan-293004650	Cross-education strength and activation after eccentric exercise	2014	49	5	582â€589		Lepley, LK and Palmieri-Smith, RM	RAYYAN-INCLUSION: {"Vickie"=>"Excluded", "jsong6"=>"Excluded"} | RAYYAN-EXCLUSION-REASONS: wrong study design,No dom vs. non-dom comparison
rayyan-293004651	The cross education of strength and skill following unilateral strength training in the upper and lower limbs	2018	120	2	468â€479		Green, LA and Gabriel, DA	RAYYAN-INCLUSION: {"Vickie"=>"Excluded", "jsong6"=>"Excluded"} | RAYYAN-EXCLUSION-REASONS: wrong study design,No dom vs. non-dom comparison
rayyan-293004652	Corticospinal adaptations and strength maintenance in the immobilized arm following 3 weeks unilateral strength training	2013	23	6	740â€748		Pearce, AJ and Hendy, A and Bowen, WA and Kidgell, DJ	RAYYAN-INCLUSION: {"Vickie"=>"Excluded", "jsong6"=>"Excluded"} | RAYYAN-EXCLUSION-REASONS: No dom vs. non-dom comparison,no right and left limb training
rayyan-293004653	Cross-Education Of Contralateral Antagonists	2010					NCT01103232,	RAYYAN-INCLUSION: {"Vickie"=>"Excluded", "jsong6"=>"Excluded"} | RAYYAN-EXCLUSION-REASONS: irrelevant
rayyan-293004654	Dominant and nondominant leg press training induce similar contralateral and ipsilateral limb training adaptations with children	2019	44	9	973â€984		Ben Othman, A and Chaouachi, A and Chaouachi, M and Makhlouf, I and Farthing, JP and Granacher, U and Behm, DG	RAYYAN-INCLUSION: {"Vickie"=>"Included", "jsong6"=>"Included"}
rayyan-293004655	Set Configuration in Strength Training Programs Modulates the Cross Education Phenomenon	2021	35	9	2414â€2420		FariÃ±as, J and Mayo, X and Giraldez-GarcÃa, MA and Carballeira, E and Fernandez-Del-Olmo, M and Rial-Vazquez, J and Kingsley, JD and Iglesias-Soler, E	RAYYAN-INCLUSION: {"Vickie"=>"Excluded", "jsong6"=>"Excluded"} | RAYYAN-EXCLUSION-REASONS: irrelevant,wrong study design
rayyan-293004656	The effect of training of the non-dominant knee muscles on ipsi-and contralateral strength gains	2015	23	3	177â€182		Abazovic, E and Kovecevic, E and Kovec, S and Bradic, J	RAYYAN-INCLUSION: {"Vickie"=>"Excluded", "jsong6"=>"Excluded"} | RAYYAN-LABELS: Trained nondom | RAYYAN-EXCLUSION-REASONS: wrong study design,No dom vs. non-dom comparison
rayyan-293004657	Comparison of Cross-Education and Global Training Effects in Adults and Youth After Unilateral Strength Training	2020					Chaouachi, A and Ben Othman, A and Chaouachi, M and Hechmi, A and Farthing, JP and Granacher, U and Behm, DG	RAYYAN-INCLUSION: {"Vickie"=>"Excluded", "jsong6"=>"Excluded"} | RAYYAN-EXCLUSION-REASONS: wrong study design,No dom vs. non-dom comparison
rayyan-293004658	The effects of cross-education on loss of muscle strength and knee function after ACL reconstruction	2014					NTR4395,	RAYYAN-INCLUSION: {"Vickie"=>"Excluded", "jsong6"=>"Excluded"} | RAYYAN-EXCLUSION-REASONS: irrelevant
rayyan-293004659	The effects of whole-body vibration on the cross-transfer of strength	2012	2012			504837	Goodwill, AM and Kidgell, DJ	RAYYAN-INCLUSION: {"Vickie"=>"Excluded", "jsong6"=>"Excluded"} | RAYYAN-EXCLUSION-REASONS: irrelevant
rayyan-293004660	Cross-education does not improve early and late-phase rehabilitation outcomes after ACL reconstruction: a randomized controlled clinical trial	2019	27	2	478â€490		Zult, T and Gokeler, A and van Raay, JJAM and Brouwer, RW and Zijdewind, I and Farthing, JP and HortobÃ¡gyi, T	RAYYAN-INCLUSION: {"Vickie"=>"Excluded", "jsong6"=>"Excluded"} | RAYYAN-EXCLUSION-REASONS: irrelevant
rayyan-293004661	Mechanomyographic and electromyographic responses to unilateral isometric training	2002	16	2	192â€201		Ebersole, KT and Housh, TJ and Johnson, GO and Perry, SR and Bull, AJ and Cramer, JT	RAYYAN-INCLUSION: {"Vickie"=>"Excluded", "jsong6"=>"Excluded"} | RAYYAN-EXCLUSION-REASONS: irrelevant,no right and left limb training
rayyan-293004662	Effct of Rehabilitation in Stroke Recovery	2016					IRCT2016102430477N1,	RAYYAN-INCLUSION: {"Vickie"=>"Excluded", "jsong6"=>"Excluded"} | RAYYAN-EXCLUSION-REASONS: irrelevant
rayyan-293004663	Home-based exercises for older adults in social isolation -STAGE 1	2020					RBR-4gdzh2,	RAYYAN-INCLUSION: {"Vickie"=>"Excluded", "jsong6"=>"Excluded"} | RAYYAN-EXCLUSION-REASONS: irrelevant
rayyan-293004664	Blood flow restriction method for inducing cross-education phenomenon and muscle function reconstruction	2022	26	17			Wang, Y., Wang, B., Wei, Z., Bao, C., Liu, S., Meng, Q.	RAYYAN-INCLUSION: {"Vickie"=>"Excluded", "jsong6"=>"Excluded"} | RAYYAN-LABELS: Review and not study | RAYYAN-EXCLUSION-REASONS: irrelevant,foreign language
rayyan-293004665	Isokinetic force-power profile of the shoulder joint in males participating in CrossFit training and competing at different levels	2021	9				Torres-Banduc, M.A., Jerez-Mayorga, D., Moran, J., Keogh, J.W.L., RamÃrez-Campillo, R.	RAYYAN-INCLUSION: {"Vickie"=>"Excluded", "jsong6"=>"Excluded"} | RAYYAN-EXCLUSION-REASONS: irrelevant
rayyan-293004667	Ipsilateral lower-to-upper limb cross-transfer effect on muscle strength, mechanical power, and lean tissue mass after accentuated eccentric loading	2021	57	5			Magdi, H.R., Maroto-Izquierdo, S., de Paz, J.A.	RAYYAN-INCLUSION: {"Vickie"=>"Excluded", "jsong6"=>"Excluded"} | RAYYAN-EXCLUSION-REASONS: irrelevant,wrong study design
rayyan-293004668	Local and contralateral effects after the application of neuromuscular electrostimulation in lower limbs	2020	17	23			Benito-MartÃnez, E., Senovilla-Herguedas, D., de la Torre-Montero, J.C., MartÃnez-BeltrÃ¡n, M.J., Reguera-GarcÃa, M.M., Alonso-CortÃ©s, B.	RAYYAN-INCLUSION: {"Vickie"=>"Excluded", "jsong6"=>"Excluded"} | RAYYAN-EXCLUSION-REASONS: irrelevant
rayyan-293004669	Relations of executive function and physical performance in middle adulthood:A prospective investigation in African American and white adults	2020	75	6			Leibel, D.K., Williams, M.R., Katzel, L.I., Evans, M.K., Zonderman, A.B., Waldstein, S.R.	RAYYAN-INCLUSION: {"Vickie"=>"Excluded", "jsong6"=>"Excluded"} | RAYYAN-EXCLUSION-REASONS: irrelevant
rayyan-293004670	Investigation of the effects of leg dominance on cross-transfer of flexibility after a unilateral treatment with foam roller-a pilot study	2020	8	3			Daskalaki, K., Pafis, G., Gioftsidou, A., Beneka, A., Bebetsos, E., Malliou, P.	RAYYAN-INCLUSION: {"Vickie"=>"Excluded", "jsong6"=>"Excluded"} | RAYYAN-LABELS: no strength outcome | RAYYAN-EXCLUSION-REASONS: irrelevant,wrong study design
rayyan-293004671	Global Training Effects of Trained and Untrained Muscles With Youth Can be Maintained During 4 Weeks of Detraining	2019	33	10			Chaouachi, A., Ben Othman, A., Makhlouf, I., Young, J.D., Granacher, U., Behm, D.G.	RAYYAN-INCLUSION: {"Vickie"=>"Excluded", "jsong6"=>"Excluded"} | RAYYAN-EXCLUSION-REASONS: irrelevant,wrong study design
rayyan-293004673	Health effects of wrist-loading sports during youth: A systematic literature review	2018	15	9			Kox, L.S., Kuijer, P.P.F.M., Thijssen, D.A.J., Kerkhoffs, G.M.M.J., Van Rijn, R.R., Frings-Dresen, M.H.W., Maas, M.	RAYYAN-INCLUSION: {"Vickie"=>"Excluded", "jsong6"=>"Excluded"} | RAYYAN-LABELS: Review and not study | RAYYAN-EXCLUSION-REASONS: irrelevant
rayyan-293004675	Magnitude and associated factors of anemia among pregnant women in Dera District: A cross-sectional study in northwest Ethiopia	2017	10	1			Derso, T., Abera, Z., Tariku, A.	RAYYAN-INCLUSION: {"Vickie"=>"Excluded", "jsong6"=>"Excluded"} | RAYYAN-EXCLUSION-REASONS: irrelevant,wrong study design
rayyan-293004679	The comparison of cross-education effect in young and elderly females from unilateral training of the elbow flexors	2014	28	1			Ehsani, F., Nodehi-Moghadam, A., Ghandali, H., Ahmadizade, Z.	RAYYAN-INCLUSION: {"Vickie"=>"Excluded", "jsong6"=>"Excluded"} | RAYYAN-EXCLUSION-REASONS: irrelevant,no right and left limb training
rayyan-293004681	Cross-education of strength depends on limb dominance: Implications for theory and application	2009	37	4			Farthing, J.P.	RAYYAN-INCLUSION: {"Vickie"=>"Excluded", "jsong6"=>"Excluded"} | RAYYAN-LABELS: Review and not study | RAYYAN-EXCLUSION-REASONS: wrong study design
