# Supplementary material for: Is there evidence for the asymmetrical transfer of strength to an untrained limb?
Source: Eur J Appl Physiol. 2024 Apr 3;124(8):2503–10. doi: 10.1007/s00421-024-05472-9 (PMC11322193; doi:10.1007/s00421-024-05472-9)
Supplement: Supplementary file 2 — Supplementary file2 (PDF 105 KB) [file 421_2024_5472_MOESM2_ESM.pdf]

Supplementary Figure 1. Risk of Bias II (BMJ, 2019) of the methodological quality of the included studies for this meta-analysis.

Intention-to-treat	Unique ID	Study ID	Experimental	Comparator	Outcome	Weight	D1	D2	D3	D4	D5	Overall
	1	1	Dominant	Non-Dominant	Strength	1	+	+	+	+	+	+
	2	2	Dominant	Non-Dominant	Strength	1	+	+	+	+	+	+
	3	3	Dominant	Non-Dominant	Strength	1	+	+	+	+	+	+

+	Low risk
!	Some concerns
-	High risk

D1	Randomisation process
D2	Deviations from the intended interventions
D3	Missing outcome data
D4	Measurement of the outcome
D5	Selection of the reported result
